# LC‐MS and High‐Throughput Data Processing Solutions for Lipid Metabolic Tracing Using Bioorthogonal Click Chemistry

**DOI:** 10.1002/anie.202501884

**Published:** 2025-05-02

**Authors:** Palina Nepachalovich, Stefano Bonciarelli, Gabriele Lombardi Bendoula, Jenny Desantis, Michela Eleuteri, Christoph Thiele, Laura Goracci, Maria Fedorova

**Affiliations:** ^1^ Center of Membrane Biochemistry and Lipid Research Faculty of Medicine Carl Gustav Carus, Technical University Dresden Tatzberg 47–49 Dresden 01307 Germany; ^2^ Mass Analytica Av Cerdanyola 92–94 Sant Cugat del Vallés 08173 Spain; ^3^ Department of Chemistry, Biology and Biotechnology University of Perugia Via Elce di Sotto 8 Perugia 06123 Italy; ^4^ Life & Medical Sciences Institute, University of Bonn Carl‐Troll‐Straße 31 Bonn 53115 Germany

**Keywords:** Click chemistry, LC‐MS, Lipostar2, Sphingolipids, Tracing lipid metabolism

## Abstract

Tracing lipid metabolism in mammalian cells presents a significant technological challenge due to the vast structural diversity of lipids involved in multiple metabolic routes. Bioorthogonal approaches based on click chemistry have revolutionized analytical performance in lipid tracing. When adapted for mass spectrometry (MS), they enable highly specific and sensitive analyses of lipid transformations at the lipidome scale. Here, we advance this approach by integrating liquid chromatography (LC) prior to MS detection and developing a software‐assisted workflow for high‐throughput data processing. LC separation resolved labeled and unmodified lipids, enabling qualitative and quantitative analysis of both lipidome fractions, as well as isomeric lipid species. Using synthetic standards and endogenously produced alkyne lipids, we characterized LC‐MS behavior, including preferential adduct formation and the extent of in‐source fragmentation. Specific fragmentation rules, derived from tandem MS experiments for 23 lipid subclasses, were implemented in Lipostar2 software for high‐throughput annotation and quantification of labeled lipids. Applying this platform, we traced metabolic pathways of palmitic and oleic acid alkynes, revealing distinct lipid incorporation patterns and metabolic bottlenecks. Altogether, here we provide an integrated analytical and bioinformatics platform for high‐throughput tracing of lipid metabolism using LC‐MS workflow.

## Graphical abstract

This study introduces an integrated analytical and bioinformatics platform for high‐throughput tracing of lipid metabolism using bioorthogonal alkyne fatty acids and optimized LC‐MS workflow. Applied to human fibrosarcoma cells, the method traced fatty acid metabolism, revealing nuances in sphingolipid routing and metabolic bottlenecks, highlighting its potential for lipidomics research.

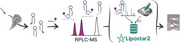

## Introduction

Lipids are critical to cellular homeostasis, functioning as structural components of membranes, energy reservoirs, and mediators of signalling pathways.^[^ ^1^ ^]^ Thus, studying lipid metabolism is essential for understanding health and disease processes in living organisms. Conventional lipidomics technologies can provide detailed snapshots of lipid species and their abundances but often lack temporal and pathway‐level resolution. However, understanding the dynamic nature of lipidomes is essential to uncover the role of lipid metabolism and its transformation in health and disease. ​Stable isotope flux analysis, commonly employing isotopically labeled precursors like ^13^C‐glucose or amino acids, is a prevalent method for tracing metabolic pathways.^[^ ^2^ ^]^ However, this approach has notable limitations. The overlap of the label with naturally occurring lipid isobars can complicate data interpretation, necessitating analysis by ultra‐high resolution mass spectrometry (MS). Even with advanced instrumentation, complex data processing is often required to accurately resolve these overlaps. Additionally, the dilution of the isotopic label across a vast array of metabolites, and the fact that stable isotope‐labeled species have the same ionization efficiency as unlabeled ones, result in limited analytical sensitivity. These challenges often confine stable isotope flux to targeted analysis, limiting its applicability to broader, untargeted metabolic investigations.

Recent progress in click chemistry has provided novel tools for tracing lipid metabolism with alkyne‐functionalized fatty acids (FA;Ys) emerging as versatile tracers due to their bioorthogonal properties.^[^ ^3^, ^4^, ^5^, ^6^, ^7^ ^]^ FA;Ys closely resemble native fatty acids (FAs) in terms of metabolic availability and reactivity, and several studies have demonstrated that FA;Ys are incorporated into lipids with the same class preferences as natural FAs, without altering the endogenous lipidome composition.^[^ ^3^, ^8^, ^9^ ^]^ This includes class‐specific incorporation, acyl chain remodeling, and turnover dynamics illustrated for different cell types. Similar results have also been reported for alkyne‐labeled polyunsaturated FAs, particularly in the context of non‐enzymatic reactions, though one should be careful while investigating pathways involving ω‐end hydroxylation.^[^ ^10^, ^11^ ^]^

**Figure 1:**
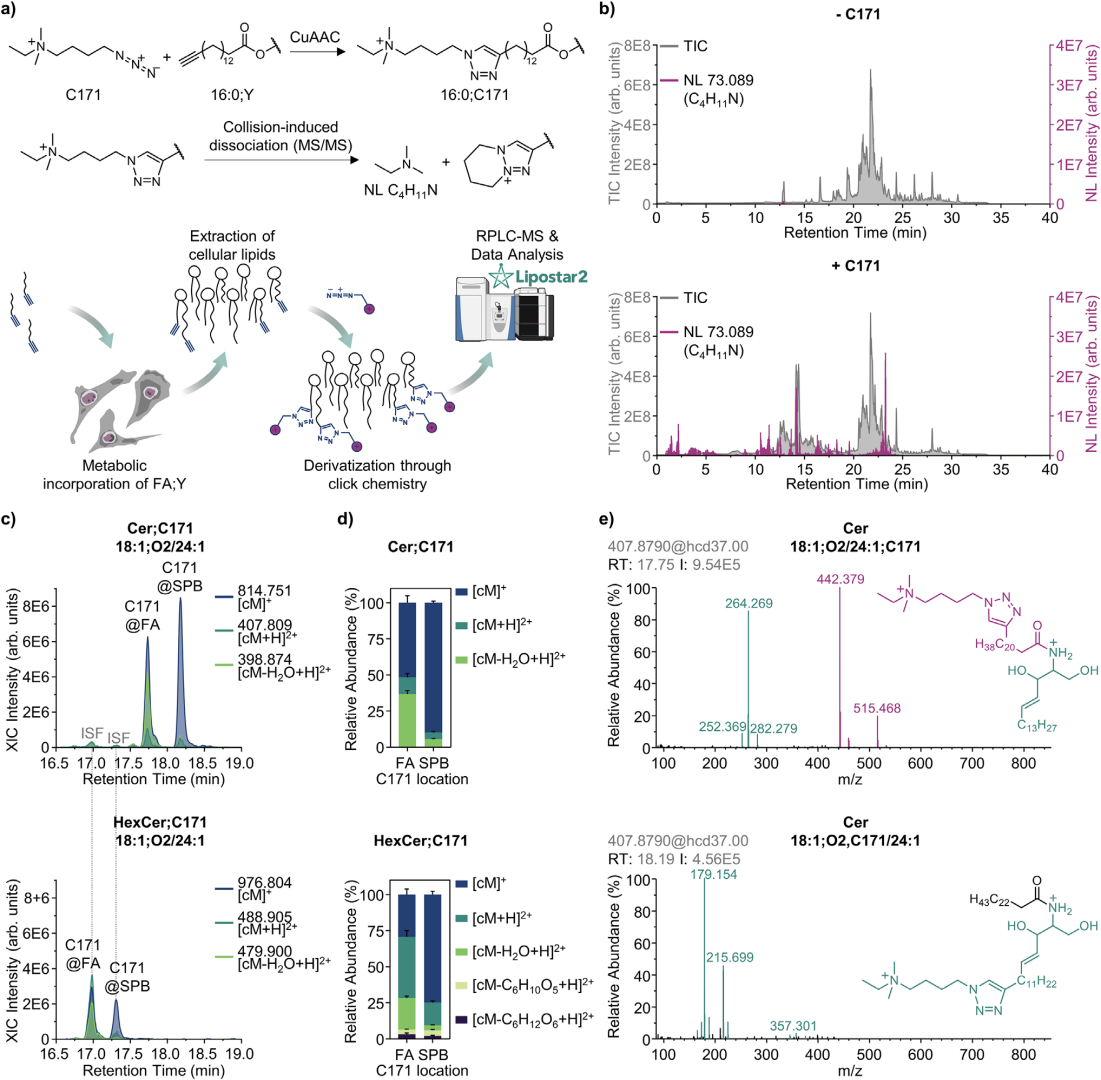
Key features of an LC‐MS method for analysis of C171‐clicked lipids. a) Schematic of the alkyne fatty acid (FA;Y) tracing experiment using copper‐catalyzed azide‐alkyne cycloaddition (CuAAC) derivatization. b) Total ion chromatograms (TIC; grey) along with C_4_H_11_N neutral loss (NL; magenta) profiles for lipid extracts from HT1080 cells treated with 100 µM palmitic acid alkyne (PA;Y) for 4 h, subjected to derivatization either in the absence (top) or presence (bottom) of C171BF_4_. c) Extracted ion chromatograms (XICs) for ceramide (Cer, top) and hexosylceramide (HexCer, bottom) 18:1;O2/24:1 with C171 being incorporated in fatty acyl (FA) or sphingoid base (SPB), illustrating chromatographic separation of isomeric pairs of endogenous and in‐source fragmentation (ISF)‐derived species. d) Relative ion abundances for Cer (top) and HexCer (bottom) with C171 being incorporated in FA or SPB. Values represent mean ± s.d., *n* = 18–24. e) MS2 spectra for Cer;C171 18:1;O2/24:1 isomers with C171 incorporated either in FA (top) or SPB (bottom) lipid moieties.

A method introduced by Thiele and colleagues^[^ ^8^, ^9^, ^12^ ^]^ further extends the application of FA;Y tracers to MS‐based lipidomics. Here, alkyne‐containing lipids are derivatized with azido‐quaternary ammonium (azido‐quat) reporters, such as C171 (Figure 1a). Resulting triazole derivatives present favorable MS properties including a constant positive charge to enhance ionization efficiency, and distinct fragmentation behavior upon collision‐induced dissociation, characterized by a specific neutral loss (NL) of dimethylethylamine (C_4_H_11_N). This approach provides a major advancement in MS‐based lipid tracing, enabling high‐throughput analysis of diverse lipid classes with enhanced sensitivity and specificity. However, direct injection MS used for the analysis of clicked lipid species might impose certain limitations, including difficulty in resolving isomeric species, vulnerability to matrix effects, and the risk of false‐positive identifications due to unaccounted in‐source fragmentation (ISF), which can hinder accurate quantification and data interpretation.^[^ ^13^ ^]^ To extend the analytical capacity of lipid metabolic tracing using the click chemistry approach, we developed a liquid chromatography‐MS (LC‐MS) platform complemented with tailored bioinformatics tools.

## Results and Discussion

Building upon the experimental setup developed by Thiele et al.,^[^ ^8^ ^]^ we incorporated reversed‐phase LC (RPLC) separation prior to MS detection of clicked lipidome (Figure 1a). The triazole moiety in derivatized lipids changed hydrophobicity of clicked compared to unmodified lipids, allowing them to be resolved chromatographically prior to MS analysis (Figure 1b). Due to LC separation, clicked and unmodified fractions of the lipidome could be analysed separately, preventing ion suppression. Additionally, when data‐dependent acquisition (DDA) is used, a large number of unique tandem mass spectra can be obtained both for clicked and unmodified lipids, supporting accurate and simultaneous annotation of both lipidome fractions. Importantly, RPLC was capable of separating even isomeric derivatized lipids (Figure 1c). For example, in cells treated with palmitic acid alkyne (FA 16:0;Y, or PA;Y), sphingolipids (SPs) contained isomeric species with the tracer incorporated either in the FA or sphingoid base (SPB) moieties.

As illustrated here for C171‐clicked ceramides (Cer;C171 18:1;O2/24:1) and hexosylceramides (HexCer;C171 18:1;O2/24:1, shorthand notation for clicked lipids is explained in Table S1), a baseline chromatographic resolution of isomeric pairs can be achieved using RPLC (Figure 1c). Furthermore, FA‐ and SPB‐clicked SPs displayed substantially different ion profiles (Figure 1d), which can be used as an additional confirmation for isomers annotation. RPLC separation further supported accurate lipid annotation and quantification by eliminating ISF artifacts through the use of retention time mapping. For instance, both HexCer;C171 18:1;O2/24:1 isomers upon ionization underwent ISF with the formation of the corresponding Cer;C171 18:1;O2/24:1 species (Figure 1c, top; signals at retention time 17.0 and 17.3 min) which, when not separated by LC, could not be distinguished from endogenous Cer. Information on the ionization preferences and ISF lability for other lipid classes including SP subclasses, glycerophospholipids, glycerolipids, and free FAs is provided in Figure S1.

Finally, base peak LC separation of isomers prior MS detection provided high quality MS2 spectra revealing their unique fragmentation patterns, exemplified here for Cer;C171 18:1;O2/24:1 isomers (Figure 1e). Using isotopically labeled alkyne standards (Tables S2–S4 and File S1) as well as lipids endogenously produced in cells supplemented with various FA;Ys (Table S5 and File S2), we comprehensively characterized MS2 fragmentation patterns of C171‐derivatized lipids representative of 15 classes and 23 subclasses reflecting the majority of mammalian lipids. In particular, we provided fragmentation patterns allowing unambiguous annotation of isomeric pairs including FA‐ versus SPB‐clicked SPs, diacylphosphatidylglycerols (PGs) versus diacylbis(monoacylglycero)phosphates (BMPs), FA‐ versus alkyl/alkenyl (FOH)‐clicked pairs for alkylacylglycerophosphocholines (PCs O‐) and alkenylacylglycerophosphoethanolamines (PEs P‐) (File S2 and Table S5). Interestingly, while analysing fragment ion patterns, we identified unique signatures influenced by the charge state of clicked lipid ions (File S1 and S2, Tables S4 and S5). Specifically, singly and doubly charged precursors may exhibit markedly different behaviors. For example, for [cM]^+^ ions of diacylglycerophosphocholine (PC) and PC O‐, we observed fragment ions at *m/z* 198.089 and 212.105 ([C_5_H_14_NO_4_P + CH_2 _+ H]^+^ and [C_5_H_14_NO_4_P + C_2_H_4 _+ H]^+^, respectively), which are consistent with a putative intramolecular S_N_2‐like rearrangement involving translocation of a methyl or ethyl group from the C171 moiety to the phosphocholine headgroup. Notably, these ions were absent in the MS2 spectra of the corresponding doubly charged [cM + H]^2+^ precursors, suggesting that protonation of the phosphate group in the doubly charged state may block such rearrangement. Furthermore, clicked PE P‐ species demonstrated rich fragmentation for [cM + H]^2+^ ions consistent with a charge‐driven rearrangement involving the plasmenyl moiety (FAlk). Depending on the site of C171 moiety, we reported ions corresponding to [cFAlk + C_2_H_6_O_3_NP]^+^ (for C171 at the plasmenyl site) or [FAlk + C_2_H_6_O_3_NP + H]⁺ (for C171 at the acyl site). These fragments likely arise through the intramolecular transfer of the cFAlk or FAlk to the ethanolamine headgroup observed only for [cM + H]^2+^ precursors, highlighting the effect of charge state and structural nuances on gas‐phase fragmentation behavior of clicked species.

**Figure 2:**
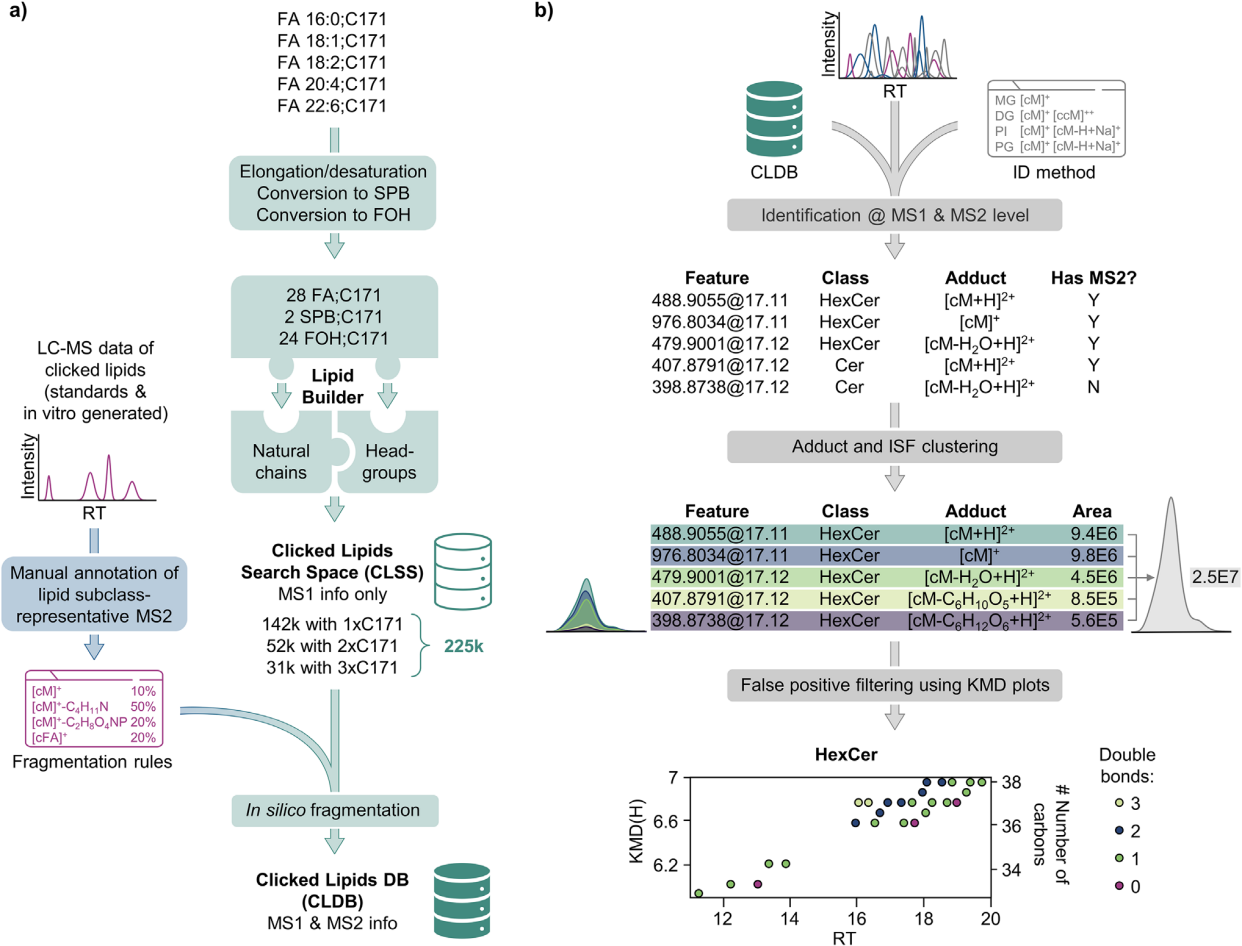
Workflow for identifying clicked lipids. a) The clicked lipid database (CLDB) construction is started by generating C171‐derivatized structures, incorporating biologically relevant fatty acid (FA) elongation, desaturation products, and FA transformation to sphingoid bases (SPBs) and fatty alcohols (FOHs). These structures are then combined with natural lipid chains and headgroups using the Lipid Builder tool in Lipostar2 DB Manager, forming a comprehensive Clicked Lipid Search Space (CLSS) that is fragmented in silico using experimentally derived MS2 fragmentation patterns for C171‐derivatized alkyne lipid standards and alkyne FAs metabolized in cells into complex alkyne lipids. b) Key steps in annotation and quantification of clicked lipids in Lipostar2 include MS1 and MS2 matching with the CLDB, adducts and in‐source fragmentation ions clustering, and Kendrick mass defect plots verification to enhance annotation accuracy and reduce false‐positive identifications.

On average, LC‐MS datasets from untargeted DDA analysis of C171‐derivatized lipidomes contained around 4000 MS2 spectra with a characteristic NL of C_4_H_11_N (73.089 for *z* = 1, 36.544 for *z *= 2, and/or 24.363 for *z *= 3 precursors). Although monitoring the NL of C_4_H_11_N to detect C171‐clicked species is valuable, it does not support automated annotation of derivatized lipids. Thus, using MS2 fragmentation patterns obtained above, a tailored approach based on the Lipostar2^[^ ^14^ ^]^ software was developed to assist high‐throughput annotation and quantification of clicked and unmodified species (Figure 2). Briefly, lipids annotation in Lipostar2 is based on comparing experimental MS2 spectra with database (DB) spectra computed using experimentally established fragmentation rules.^[^ ^14^ ^]^ To create a comprehensive knowledge‐based DB for C171‐derivatized lipids, we started with the triazole forms of FA;Ys typically used in lipid tracing studies: PA;Y, oleic (FA 18:1;Y, or OA;Y), linoleic (FA 18:2;Y), arachidonic (FA 20:4;Y), and docosahexaenoic (FA 22:6;Y) acid alkynes. Then, we expanded this set by considering metabolic pathways relevant to mammalian cells, including FA elongation, desaturation, and conversion to SPBs and FOHs, which resulted in 54 clicked chains (Table S6). Lipid Builder tool within Lipostar2 DB Manager was used to combine the structures of clicked chains with various headgroups and natural chains to obtain a structural DB of 224 514 lipids from 15 main classes and 23 subclasses (Clicked Lipid Search Space, CLSS).

Next, the manually curated interpretation of the MS2 fragmentation patterns described above (Files S1 and S2, Tables S4 and S5) was coded in the Lipostar2 format and used for the in silico fragmentation of the structural DB in Lipostar2 Lipid DB Manager.^[^ ^14^ ^]^ As a result, we obtained a fully functional Clicked Lipid DB (CLDB), which we used alongside the refined identification method (Table S7) for high‐throughput annotation in Lipostar2, taking advantage of built‐in software features, such as clustering of adducts and ISF ions, and retention time mapping using Kendrick mass defect plots to exclude false positive annotations. Importantly, Lipostar2 supports the simultaneous identification of clicked and natural lipids using a merged DB (CLDB and, for example, a LIPID MAPS Structure DB^[^ ^15^ ^]^ fragmented according to conventional rules) and the corresponding combined identification method.

To validate the efficiency of newly developed LC‐MS and Lipostar2 workflow for the analysis of lipidome dynamics, we traced the metabolic fates of PA;Y and OA;Y in mammalian cells under the condition of FA;Y overload. Indeed, exposure to elevated amounts of free FA is often associated with lipotoxicity, with adverse effects attributed to different lipid classes. Here, HT1080 fibrosarcoma cells were incubated with 100 µM PA;Y or OA;Y for 4 h, lipids were extracted, derivatized, and analysed using LC‐MS and Lipostar2 platform.

Isotopically labeled internal standards (ISTDs), including both native and alkyne‐tagged lipids (detailed in Table S2), enabled relative quantification of clicked and unmodified lipid fractions via one‐point calibration. The analytical validity of this quantification strategy was supported by ten‐point external calibration curves, which established sensitivity, limits of detection (LOD), and quantification (LOQ) across lipid classes represented by ISTDs (Tables S3, S8; Figure S2). The lowest LOD and LOQ values (0.01 and 0.02 pmol loaded onto column, respectively) were shown for dihydroCer;C171 and clicked diacylglycerols (DG;C171s), while C171‐derivatized diacylglycerophosphoethanolamine (PE;C171) showed the highest LOD and LOQ with 0.41 and 1.2 pmol per column, respectively. The signal for all ISTDs showed very low variability at concentration levels above the LOQ, with the highest coefficient of variation (9.7%) observed for the clicked diacylglycerophosphoserine (PS;C171) standard. Additionally, we quantified the sensitivity enhancement conferred by the introduction of a charged C171 moiety upon derivatization of alkyne lipids. Signal gain for C171‐labeled species ranged from 2.4‐ to 246‐fold higher compared to their underivatized alkyne counterparts, depending on the lipid subclass (Table S3). Finally, we assessed recovery of clicked, alkyne and non‐alkyne lipids using ISTDs spiked into the matrix (HT1080 cell lipid extract) before and after extraction (Figure S3). Although recovery varied across subclasses (from 19.4 ± 1.4% for clicked diacylglycerophosphoinositol (PI 17:0;C171_15:1‐[2]H8) to 83.7 ± 1.4% for DG 17:0;C171_15:1‐[2]H8), the use of class‐specific alkyne ISTDs spiked into cell pellets before lipid extraction allows to account for any losses during the multistep experimental procedure.

**Figure 3:**
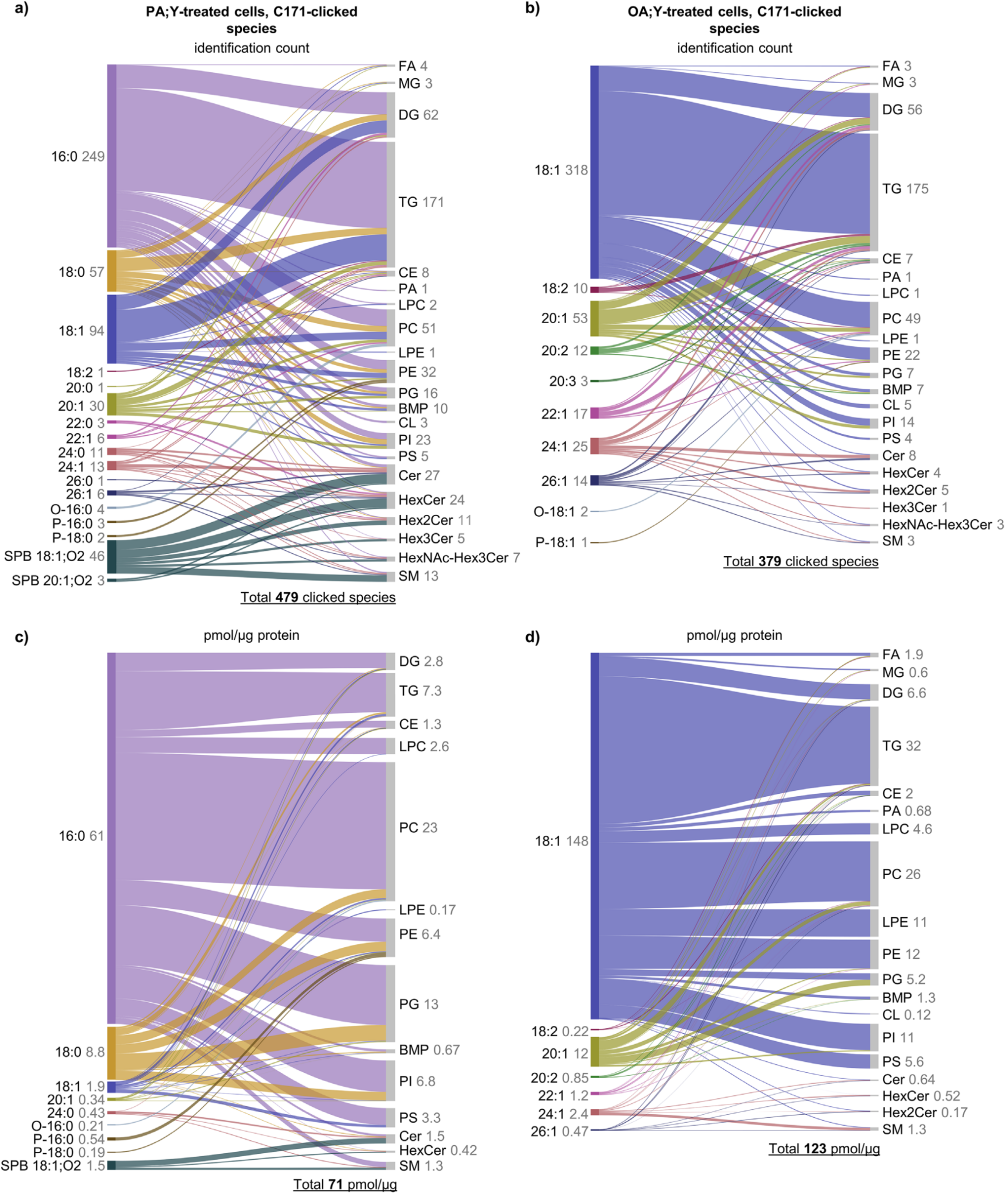
Anabolism of palmitic and oleic acid alkynes in HT1080 human fibrosarcoma cells. a) and b) Qualitative Sankey plots showing the number of annotated C171‐clicked chains detected in complex lipids in lipid extracts from HT1080 cells treated with 100 µM palmitic acid alkyne (PA;Y; a) or oleic acid alkyne (OA;Y; b) for 4 h. c) and d) Quantitative Sankey plots showing concentrations of C171‐clicked chains detected in complex lipids in PA;Y‐ (c) and OA;Y‐ d) treated cells; only species with an average total quantity >0.1 pmol µg^−1^ protein are shown. The number of identified species (a) and (b) and the concentrations (c) and (d) are shown in grey. Values in (c) and (d) represent mean, *n* = 3.

In the PA;Y‐treated cells, 479 clicked lipids were identified, including 428 singly (1C171), 50 doubly (2C171), and one triply labeled (3C171) species (Figure 3a). Upon OA;Y incubation, 379 clicked lipids were annotated, comprising 309 1C171, 64 2C171, and six 3C171 species (Figure 3b). 136 clicked lipids were identical between PA;Y and OA;Y samples (Table S9), reflecting the convergence of lipid metabolic pathways. For both FA;Y, the number of annotated clicked lipid species followed the trend triglycerides (TG) >> DG ≥ PC > PE with most of the identified species containing the original PA;Y or OA;Y chains or its CH_2_‐elongated species (Figure S4). However, when compared in quantitative terms, the FA;Ys showed rather distinct metabolic preferences (Figure 3c,d). OA;Y preferentially incorporated into TG, including doubly‐ and triply‐labeled forms. Conversely, PA;Y showed a stronger preference for PC, with labeled PC representing threefold higher enrichment than TG. Interestingly, the second most PA;Y‐enriched lipid class was phosphatidylglycerols (PG). Previously, it was demonstrated that PA‐induced lipotoxicity in MDA‐MB‐231 cells led to mitochondrial dysfunction, characterized by a rapid decrease in cardiolipin (CL) levels, cytochrome c release, and apoptosis.^[^ ^16^ ^]^ Using thin‐layer chromatography, authors also showed a significant build‐up of phosphatidic acid and/or PG in palmitate‐treated cells, suggesting impaired lipid remodeling toward CL. While they were not able to differentiate between phosphatidic acid and PG species, our tracing analysis provides direct evidence that PA;Y and its elongation/desaturation products are readily incorporated into PG lipids.

**Figure 4:**
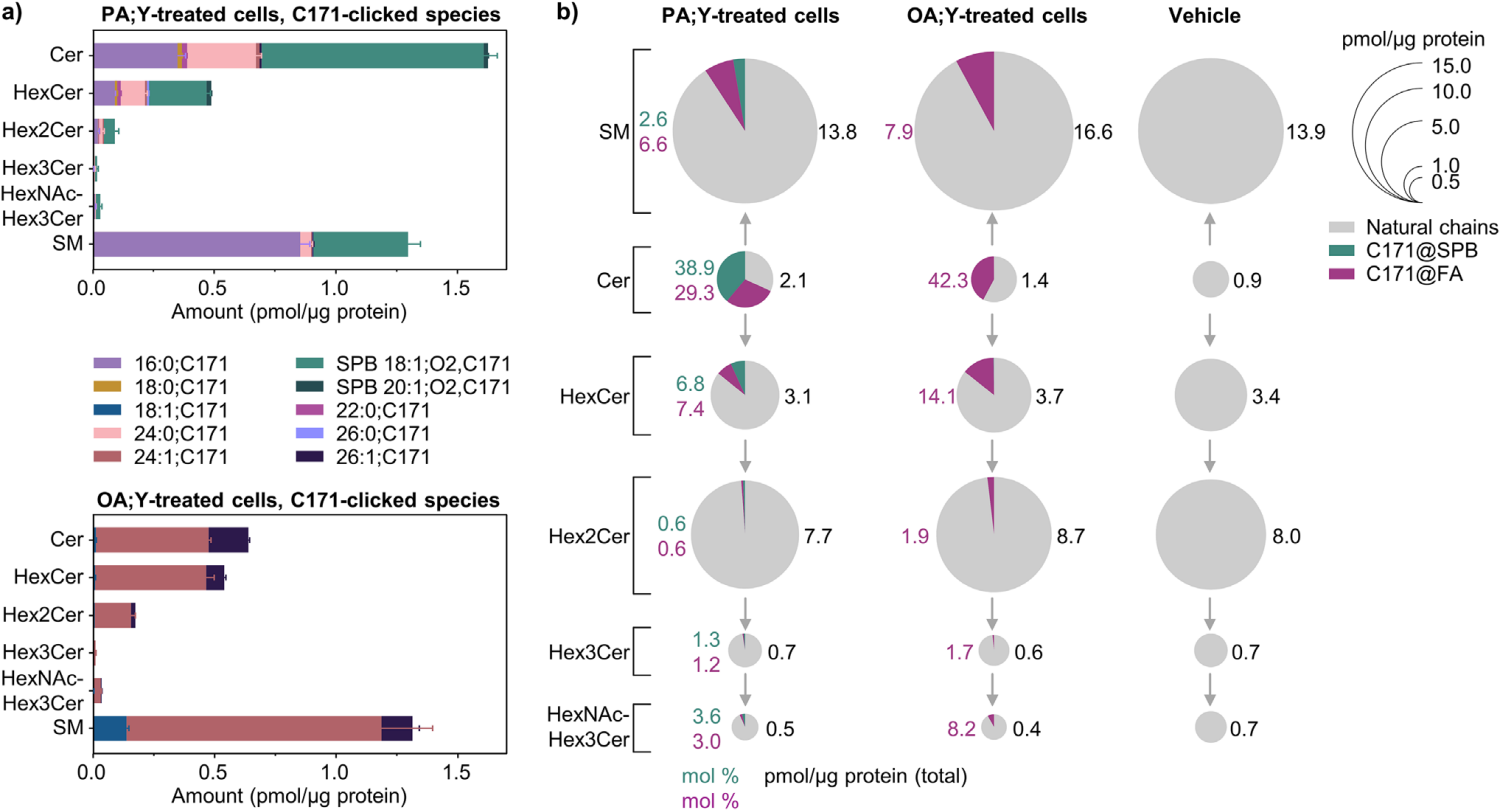
Sphingolipid (SP) profiling of HT1080 human fibrosarcoma cells treated with palmitic and oleic acid alkynes. a) Bar plots showing the concentration of C171‐clicked FA and SPB chains in various SP subclasses identified in PA;Y (top) or OA;Y (bottom) treated cells. b) Bubble pie charts illustrating the quantitative distribution of C171‐clicked and unlabeled SP in cells treated with PA;Y (left), OA;Y (middle), or vehicle (right). Bubble size corresponds to the total concentration of a SP subclass, with C171‐clicked FA (magenta) and SPB (green) abundances (mol % of total) shown as fractions. Values in (a) represent mean ± s.d., values in (b) represent mean, *n* = 3.

Among other lipid classes closely associated with lipotoxic effects upon FA overload, SPs are particularly interesting.^[^ ^17^, ^18^, ^19^ ^]^ Thus, we decided to address in more details the metabolic routing of PA;Y and OA;Y into SPs. PA;Y incorporation in SPs was notably higher (3.5 ± 0.3 pmol µg^−1^ protein) than OA;Y (2.6 ± 0.3 pmol µg^−1^ protein; Table S9, Figure 3c,d). Quantitative analysis of SP subclasses revealed distinct incorporation patterns (Figure 4a). PA;Y was preferentially incorporated into Cer and sphingomyelin (SM) in the form of SPB and labeled acyl chains in the form of FA 16:0;C171 or, to a smaller extent, its elongation product FA 24:0;C171 (Figure 4a). In contrast, OA;Y incorporated preferentially in SM in the form of elongated species such as FA 24:1;C171 and FA 26:1;C171, reflecting its efficient routing through elongation pathway. PA;Y‐derived Cer exhibited approximately double the amount compared to OA;Y‐derived Cer, attributed to PA;Y's dual role as a precursor of both SPB and FA chains. However, this distinction diminished in SM and HexCer, where the incorporation levels of PA;Y‐ and OA;Y‐derived alkyne chains were comparable.

Finally, given that the developed LC‐MS method is well‐suited for the simultaneous analysis of both clicked and natural fractions of the lipidome, we compared the contribution of labeled species to the overall amounts of SPs in a quantitative form (Figure 4b). The PA;Y treatment caused substantial Cer enrichment, with two‐thirds of the total Cer being labeled after 4 h of tracer supplementation. This Cer accumulation aligns well with the documented role of PA‐induced lipotoxicity, which has been linked to Cer‐mediated amplification of apoptotic pathways.^[^ ^20^ ^]^ In contrast, OA;Y treatment led to a broader distribution across SP classes (Figure S5 and Table S10), and, interestingly, to a significant increase in the overall amount of SM relative to both PA;Y‐ and vehicle‐treated cells (*p* = 0.0013 and *p* = 0.0017, respectively; one‐way ANOVA followed by multiple comparisons test). Importantly, the quantitative profile of SP acyl chain distribution (Figure S5) closely matched RNA sequencing (RNA‐seq) data for ceramide synthase (CerS) isoforms in HT1080 cells.^[^ ^21^ ^]^ Thus, CerS1 and CerS4, incorporating FA 18:0 and FA 20:0 in Cer, show negligible expression, whereas CerS5 and CerS6, generating Cer with FA 16:0, are moderately expressed. However, the most highly expressed isoform in HT1080 cells is CerS2, responsible for the synthesis of very long‐chain FA 22:0–26:0 Cer.^[^ ^22^ ^]^ Consistently, our lipidomics data reveal minimal presence of SPs with FA 18:0, FA 18:1, and FA 20:0 chains in both unlabeled and clicked sphingolipidomes (Figure S5). This alignment between RNA‐seq and lipid tracing data further highlights the high accuracy of the developed analytical and bioinformatics platform to capture lipid metabolic processes and reflects the functional role of CerS isoforms in SP biosynthesis.

## Conclusion

In summary, we present a comprehensive analytical pipeline and software solution specifically tailored for LC‐MS data processing in click chemistry experiments, enabling detailed mapping of FA anabolism. By using alkyne‐labeled FA analogues in combination with the azido‐quat reporter C171, we achieved substantial sensitivity gains – ranging from 2.4‐ to 246‐fold – compared to underivatized alkyne lipids. In addition to improved sensitivity, the derivatized lipids were chromatographically separated from their native counterparts and produced distinct MS2 fragmentation patterns, collectively enhancing the selectivity of metabolic tracing. This analytical strategy was complemented by a software‐assisted workflow embedded in Lipostar2, providing an automated and integrated solution for high‐throughput identification and relative quantification of clicked lipids. Furthermore, the comprehensive DB developed for the C171 reporter can be seamlessly adapted to the isotopologue family of MS reporters, enabling multiplexed pulse‐chase experiments in single LC‐MS runs.^[^ ^8^, ^9^ ^]^

By combining analytical precision with high‐throughput capabilities, this approach opens new avenues for discovering the intricacy of FA metabolism and its implications for health and disease. We applied this workflow to trace the incorporation of PA;Y and OA;Y analogues into the cellular lipidome. In HT1080 cells, detailed analysis of the SP fraction revealed distinct preferences of each FA for specific lipidome subfractions. The high resolving power of the method allowed us to quantify the differential incorporation of PA;Y into SPBs versus FAs of Cer, as well as their selective routing into SM versus HexCer. Simultaneous quantification of both labeled and unlabeled lipidome fractions confirmed that PA;Y overload stimulates Cer accumulation via *de novo* synthesis, a process often linked to the lipotoxic and pro‐apoptotic effects of saturated FA. Conversely, OA;Y supplementation resulted in significantly elevated SM levels, suggesting differential modulation of *de novo* synthesis and salvage pathways of SP metabolism by these FAs. Future pulse‐chase experiments, in combination with selective pathway inhibitors, will help to dissect FA‐specific mechanisms underlying lipotoxicity.

Despite its advantages, the method has certain limitations. The derivatization process significantly increases the polarity of analytes, making the analysis of highly polar clicked lipids challenging. Such species (e.g., derivatized acylcarnitines) require specialized extraction protocols and LC settings, which are not fully addressed by the current method. Additionally, quantification results for derivatized PG, BMP, CL, lysoPE, and complex Hex(n)Cer (n > 1) should be interpreted with caution due to the absence of subclass‐specific ISTDs, necessitating the use of analogues with similar LC‐MS behavior for estimation.

## Supporting Information

The authors have cited additional references within the Supporting Information.^[^ ^23^, ^24^, ^25^, ^26^ ^]^

## Conflict of Interests

The authors declare no conflict of interest.

## Supporting information



Supporting Information

Supporting Information

## Data Availability

The data that support the findings of this study are available in the supplementary material of this article
