# Supplementary material for: LC‐MS and High‐Throughput Data Processing Solutions for Lipid Metabolic Tracing Using Bioorthogonal Click Chemistry
Source: Angew Chem Int Ed Engl. 2025 May 2;64(27):e202501884. doi: 10.1002/anie.202501884 (PMC12207371; doi:10.1002/anie.202501884)
Supplement: Supplementary file 1 — Supporting Information [file ANIE-64-e202501884-s002.pdf]

Table of Contents

Table S1. Shorthand notation and ion nomenclature used for C171-derivatized lipids. 2

Table S3. Analytical performance characteristics of selected clicked lipid standards determined by external calibration approach. 3

Figure S1. Ionization preferences and in-source-fragmentation of C171-derivatized lipids. 4

Figure S2. External calibration of C171-derivatized, alkyne-, and non-alkyne lipid standards. 5

Figure S3. Recovery of C171-derivatized, alkyne-, and non-alkyne lipid standards from a simulated click reaction. 6

Figure S4. Anabolism of palmitic (PA;Y) and oleic (OA;Y) acid alkynes. 7

Figure S5. Integrated view of the natural and labelled sphingolipidome. 8

Experimental Section 9

References 23

Author Contributions 24

External Supporting Data

File S1. LC-MS description of C171-derivatized alkyne-containing lipids from Click Internal Standard Mix.

File S2. LC-MS description of C171-derivatized alkyne-containing lipids produced endogenously (one example per lipid subclass/type) from FA;Y.

Table S2. Composition of Master Internal Standard Mix.

Table S4. Generalized MS2 fragmentation patterns of C171-derivatized alkyne-containing lipids from Click Internal Standard Mix.

Table S5. Generalized MS2 fragmentation patterns of C171-derivatized alkyne-containing lipids produced endogenously from FA;Y.

Table S6. Overview of chains used to generate C171-derivatized lipid structures for Clicked Lipids Database.

Table S7. LC-MS and identification settings used to configure Lipostar2 for processing raw files for C171-derivatized lipid analysis.

Table S8. Univariate regression analysis and analytical performance parameters for C171-derivatized, alkyne-, and non-alkyne lipid standards.

Table S9. Identification and quantification of C171-derivatized lipids in HT1080 cells treated with PA;Y or OA;Y.

Table S10. Identification and quantification of native and C171-derivatized sphingolipids in HT1080 cells treated with PA;Y, OA;Y or Vehicle.

Table S11. Source Data for Figure 1c, Figures 3 and 4, Figures S1, S3-S5.

Table S1. Shorthand notation and ion nomenclature used for C171-derivatized lipids.

Sub-class	N C171	Shorthand notation and ion structures
LPE	1	LPE 18:1;C171<br>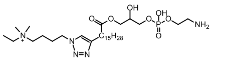 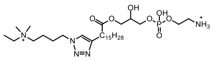<br>Chemical Formula: $C_{31}H_{61}N_5O_7P^+$ m/z: 646.4304 — Chemical Formula: $C_{31}H_{62}N_5O_7P^{2+}$ m/z: 323.7188<br>$[cM]^+$ — $[cM+H]^{2+}$
PE	1	PE 18:1;C171_18:1<br>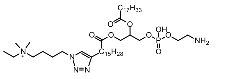 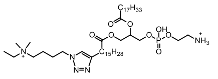<br>Chemical Formula: $C_{49}H_{93}N_5O_8P^+$ m/z: 910.6757 — Chemical Formula: $C_{49}H_{94}N_5O_8P^{2+}$ m/z: 455.8415<br>$[cM]^+$ — $[cM+H]^{2+}$
PE	2	PE 18:1;C171_18:1;C171<br>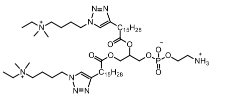 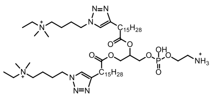<br>Chemical Formula: $C_{57}H_{108}N_9O_8P^{2+}$ m/z: 538.9024 — Chemical Formula: $C_{57}H_{109}N_9O_8P^{3+}$ m/z: 359.6040<br>$[ccM]^{2+}$ — $[ccM+H]^{3+}$
PE P-	1	PE P-16:0;C171_18:1<br>(PE P- C171@FOH)<br>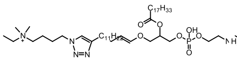 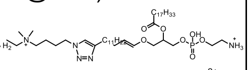<br>Chemical Formula: $C_{47}H_{91}N_5O_7P^+$ m/z: 868.6651 — Chemical Formula: $C_{47}H_{92}N_5O_7P^{2+}$ m/z: 434.8362<br>$[cM]^+$ — $[cM+H]^{2+}$
PE P-	1	PE P-16:0_18:1;C171<br>(PE P- C171@FA)<br>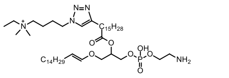 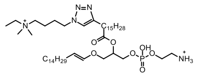<br>Chemical Formula: $C_{47}H_{91}N_5O_7P^+$ m/z: 868.6651 — Chemical Formula: $C_{47}H_{92}N_5O_7P^{2+}$ m/z: 434.8362<br>$[cM]^+$ — $[cM+H]^{2+}$
PE P-	2	PE P-16:0;C171_18:1;C171<br>(PE P- 2C171@FOH&FA)<br>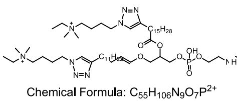 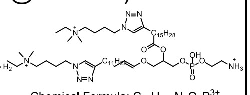<br>Chemical Formula: $C_{55}H_{106}N_9O_7P^{2+}$ m/z: 517.8971 — Chemical Formula: $C_{55}H_{107}N_9O_7P^{3+}$ m/z: 345.6005<br>$[ccM]^{2+}$ — $[ccM+H]^{3+}$
Cer	1	Cer 18:1;O2/24:1;C171<br>(Cer C171@FA)<br>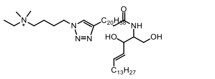 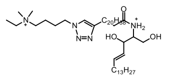<br>Chemical Formula: $C_{50}H_{96}N_5O_3^+$ m/z: 814.7508 — Chemical Formula: $C_{50}H_{97}N_5O_3^{2+}$ m/z: 407.8790<br>$[cM]^+$ — $[cM+H]^{2+}$
Cer	1	Cer 18:1;O2,C171/24:1<br>(Cer C171@SPB)<br>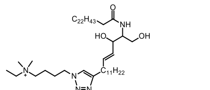 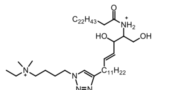<br>Chemical Formula: $C_{50}H_{96}N_5O_3^+$ m/z: 814.7508 — Chemical Formula: $C_{50}H_{97}N_5O_3^{2+}$ m/z: 407.8790<br>$[cM]^+$ — $[cM+H]^{2+}$
Cer	2	Cer 18:1;O2,C171/24:1;C171<br>(Cer 2C171@FA&SPB)<br>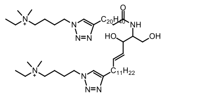 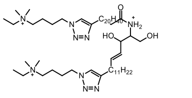<br>Chemical Formula: $C_{58}H_{113}N_9O_3^{2+}$ m/z: 491.9478 — Chemical Formula: $C_{58}H_{114}N_9O_3^{3+}$ m/z: 328.3010<br>$[ccM]^{2+}$ — $[ccM+H]^{3+}$

Sub-class	N C171	Shorthand notation and ion structures
FA	1	FA 18:1;C171<br>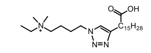<br>Chemical Formula: $C_{26}H_{49}N_4O_2^+$ m/z: 449.3851<br>$[cM]^+$
MG	1	MG 18:1;C171<br>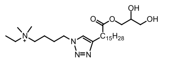<br>Chemical Formula: $C_{29}H_{55}N_4O_4^+$ m/z: 523.4218<br>$[cM]^+$
DG	1	DG 18:1;C171_18:1<br>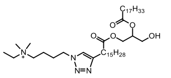<br>Chemical Formula: $C_{47}H_{87}N_4O_5^+$ m/z: 787.6671<br>$[cM]^+$
DG	2	DG 18:1;C171_18:1;C171<br>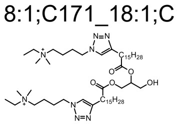<br>Chemical Formula: $C_{55}H_{102}N_8O_5^{2+}$ m/z: 477.3981<br>$[ccM]^{2+}$
TG	1	TG 18:1;C171_18:1_18:1<br>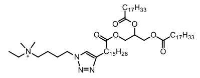<br>Chemical Formula: $C_{65}H_{119}N_4O_6^+$ m/z: 1051.9125<br>$[cM]^+$
TG	2	TG 18:1;C171_18:1;C171_18:1<br>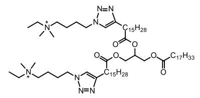<br>Chemical Formula: $C_{73}H_{134}N_8O_6^{2+}$ m/z: 609.5208<br>$[ccM]^{2+}$
TG	3	TG 18:1;C171_18:1;C171_18:1;C171<br>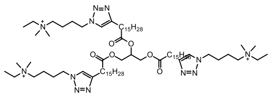<br>Chemical Formula: $C_{81}H_{149}N_{12}O_6^{3+}$ m/z: 462.0569<br>$[cccM]^{3+}$
CE	1	CE 18:1;C171<br>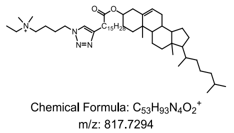<br>Chemical Formula: $C_{53}H_{93}N_4O_2^+$ m/z: 817.7294<br>$[cM]^+$

Table S3. Analytical performance characteristics of selected clicked lipid standards determined by external calibration approach.

ID[b]	R^2	Sensitivity ($pmol^{-1}$)	LOD (pmol)	LOQ (pmol)	Sensitivity gain (fold change)
CE-[2]H7 17:0;C171	0.9993	$4.1 \cdot 10^7$	0.02	0.05	130[a]
Cer 18:0;O2;C171/15:1-[2]H8	0.9997	$2.8 \cdot 10^7$	0.01	0.02	2.9
Cer 18:1;O2,C171/6:0	0.9948	$4.3 \cdot 10^7$	0.06	0.19	2.4
DG 17:0;C171_15:1-[2]H8	0.9996	$2.8 \cdot 10^7$	0.01	0.02	21.4
HexCer 18:1;O2/15:0;C171-[13]C2	0.9990	$5.2 \cdot 10^7$	0.05	0.16	9.6
MG-[13]C3 19:1;C171	0.9984	$5.3 \cdot 10^7$	0.06	0.17	246
PA 17:0;C171_15:1-[2]H8	0.9943	$1.2 \cdot 10^6$	0.1	0.3	5.9
PC 17:0;C171_15:1-[2]H8	0.9973	$2.2 \cdot 10^8$	0.37	1.1	14.9
PE 17:0;C171_15:1-[2]H8	0.9906	$2.7 \cdot 10^7$	0.41	1.2	28.5
PI 17:0;C171_15:1-[2]H8	0.9941	$5.1 \cdot 10^5$	0.27	0.82	3.3
PS 17:0;C171_15:1-[2]H8	0.9907	$4.4 \cdot 10^6$	0.32	0.97	28.3
SM 18:1;O2/15:0;C171-[13]C2	0.9971	$1.3 \cdot 10^8$	0.1	0.3	16.6
TG 17:0;C171_15:1-[2]H8_16:0	0.9995	$4.2 \cdot 10^7$	0.06	0.19	3

[a] Sensitivity gain for CE-2[H]7 17:0;C171 was calculated as the ratio to CE 18:1-2[H]7 standard from SPLASH LipidoMIX.

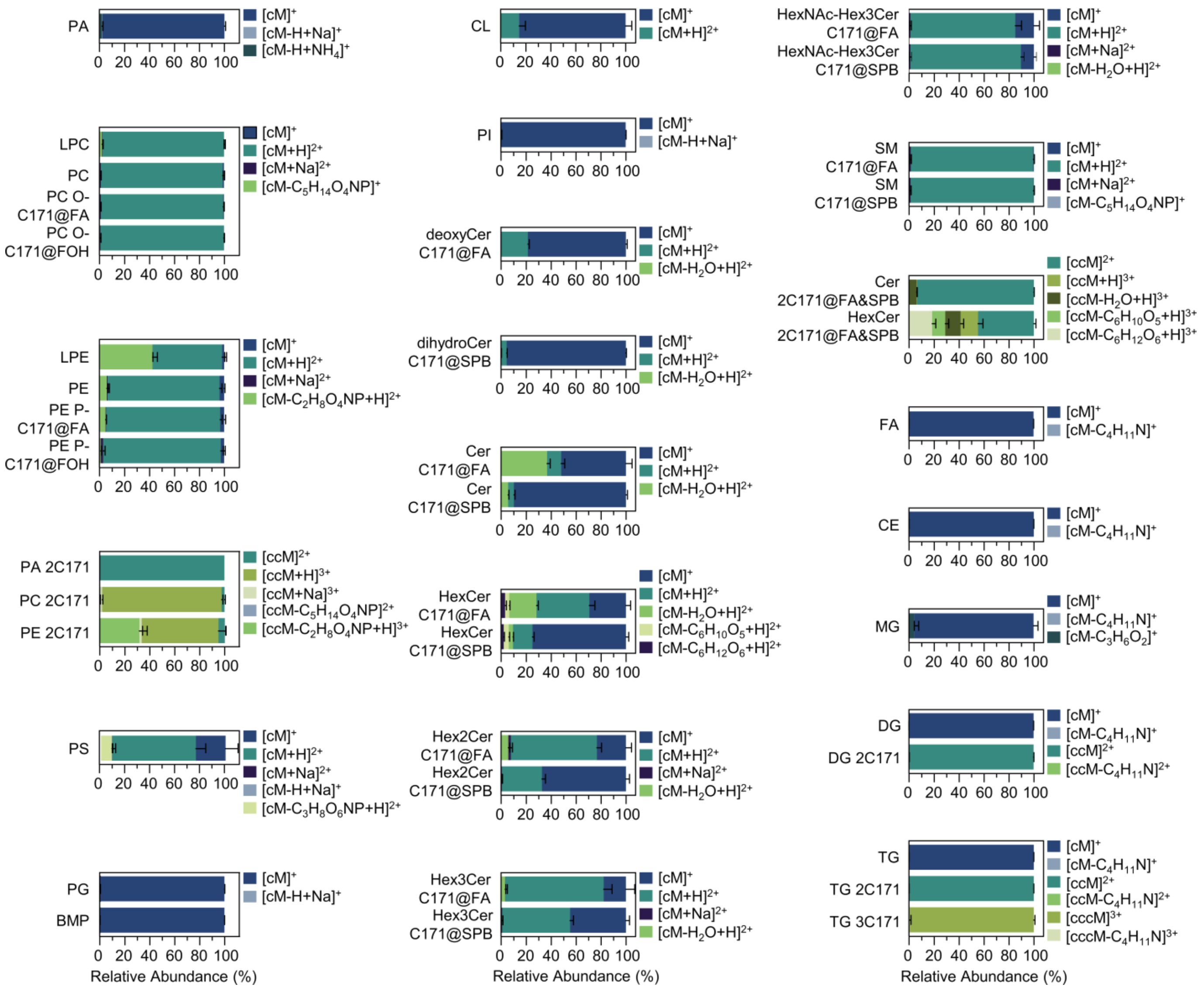

Figure S1. Ionization preferences and in-source-fragmentation of C171-derivatized lipids from different subclasses. The bar plots show relative abundances for quasi-molecular ions of C171-clicked species detected in positive, full MS ion mode. Values represent mean ± s.d., n = 3-45.

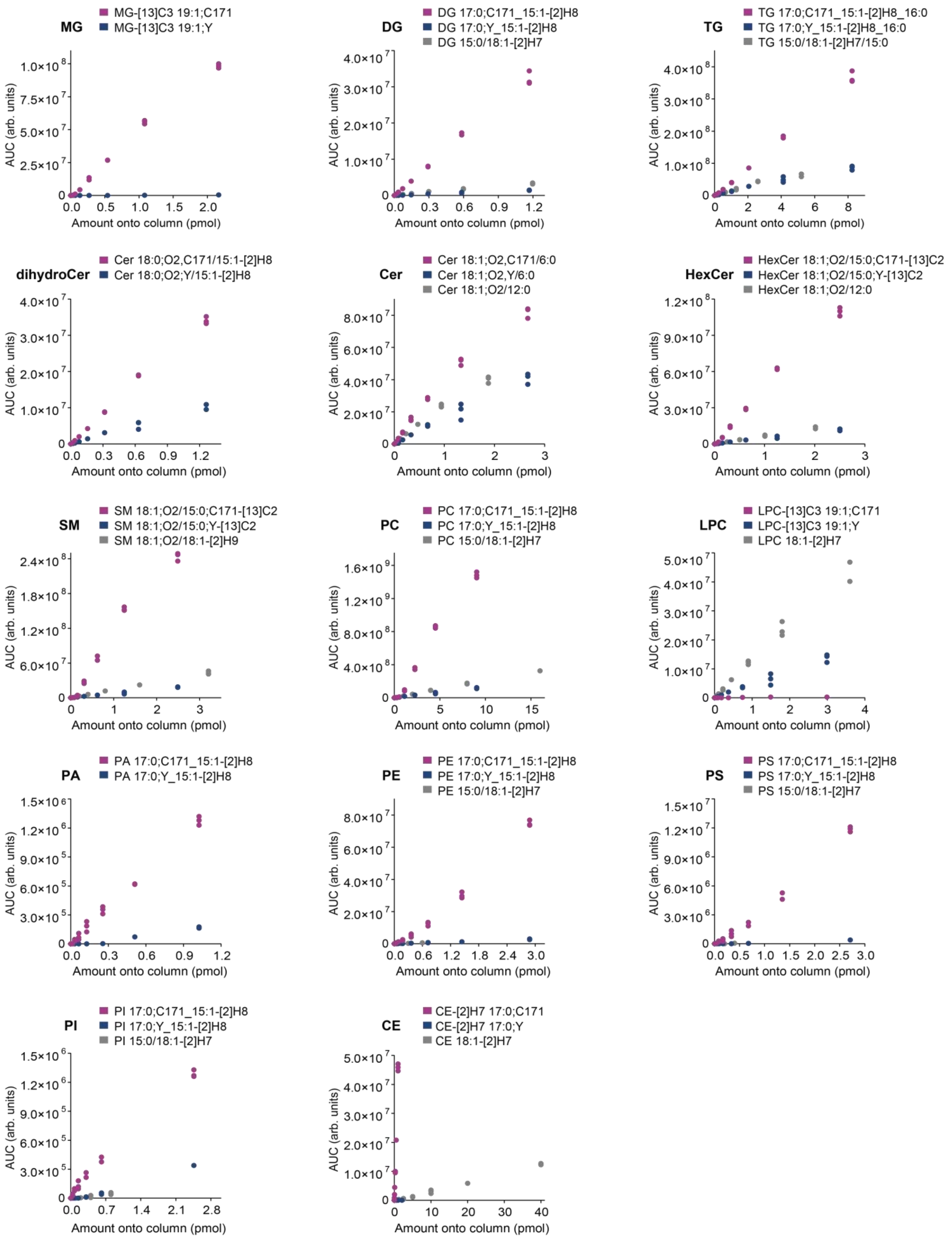

Figure S2. External calibration for C171-derivatized, alkyne-, and non-alkyne lipid standards. A Master Mix containing alkyne and non-alkyne lipid standards was spiked into HT1080 cell pellets and samples were processed according to the protocol detailed in the Experimental Section. Values represent individual area under the curve (AUC) for each concentration levels, n=3. Univariate linear regression fitting is detailed in Table S8.

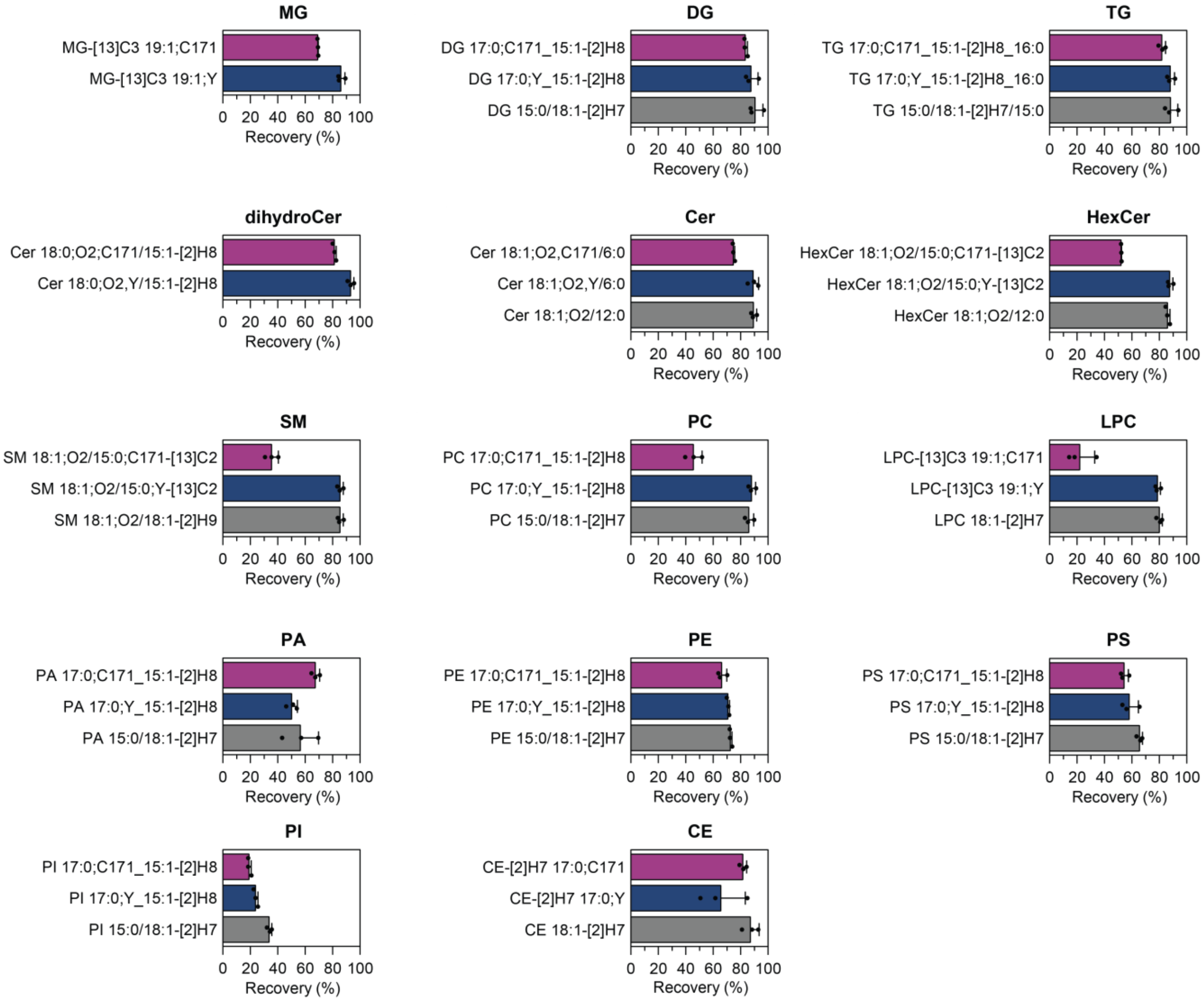

Figure S3. Recovery of C171-derivatized, alkyne-, and non-alkyne lipid standards from a simulated click reaction. A Master Mix containing pre-clicked lipid standards (magenta), their corresponding alkyne precursors (blue), and lipids from SPLASH LipidoMIX and Cer/Sph Mix I (grey) was spiked into HT1080 lipid extracts either before or after the derivatization step (performed in the absence of the C171 reagent), followed by the extraction with $CHCl_3$:H_2O (1:1, v/v). Recovery is expressed as the ratio of lipid peak areas (before/after extraction), shown as mean ± s.d., n = 3.

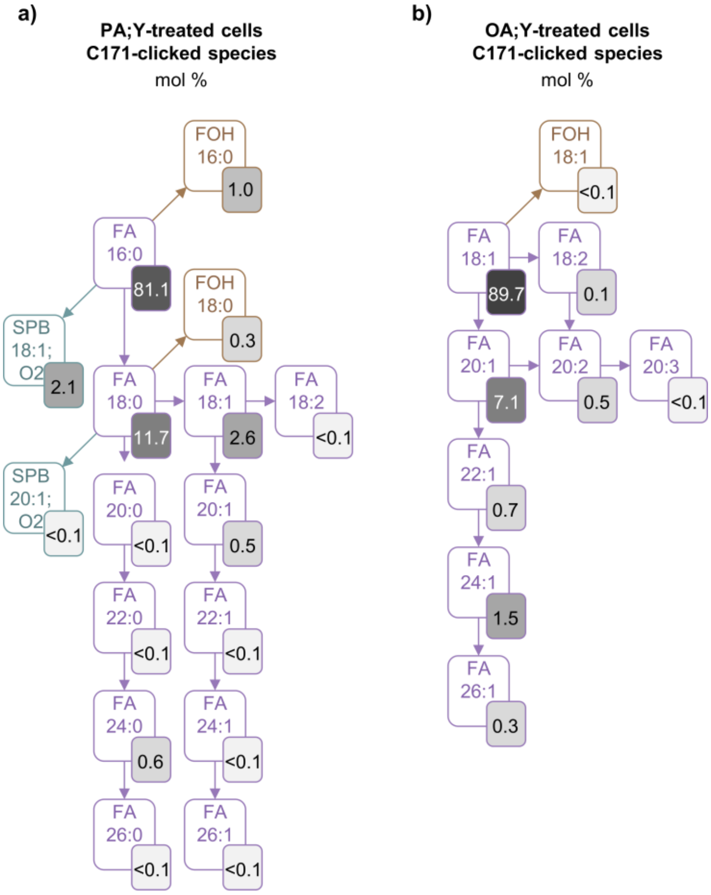

Figure S4. Anabolism of palmitic (PA;Y) and oleic (OA;Y) acid alkynes as determined by quantification in C171-derivatized complex lipids. Schematics illustrates elongation, desaturation, conversion to fatty alcohols (FOH), and sphingoid bases (SPB) for PA;Y (a) and OA;Y (b) treated cells, with values in rectangles showing relative amounts of the corresponding C171-clicked chains (mol % of total). Values represent mean, n = 3.

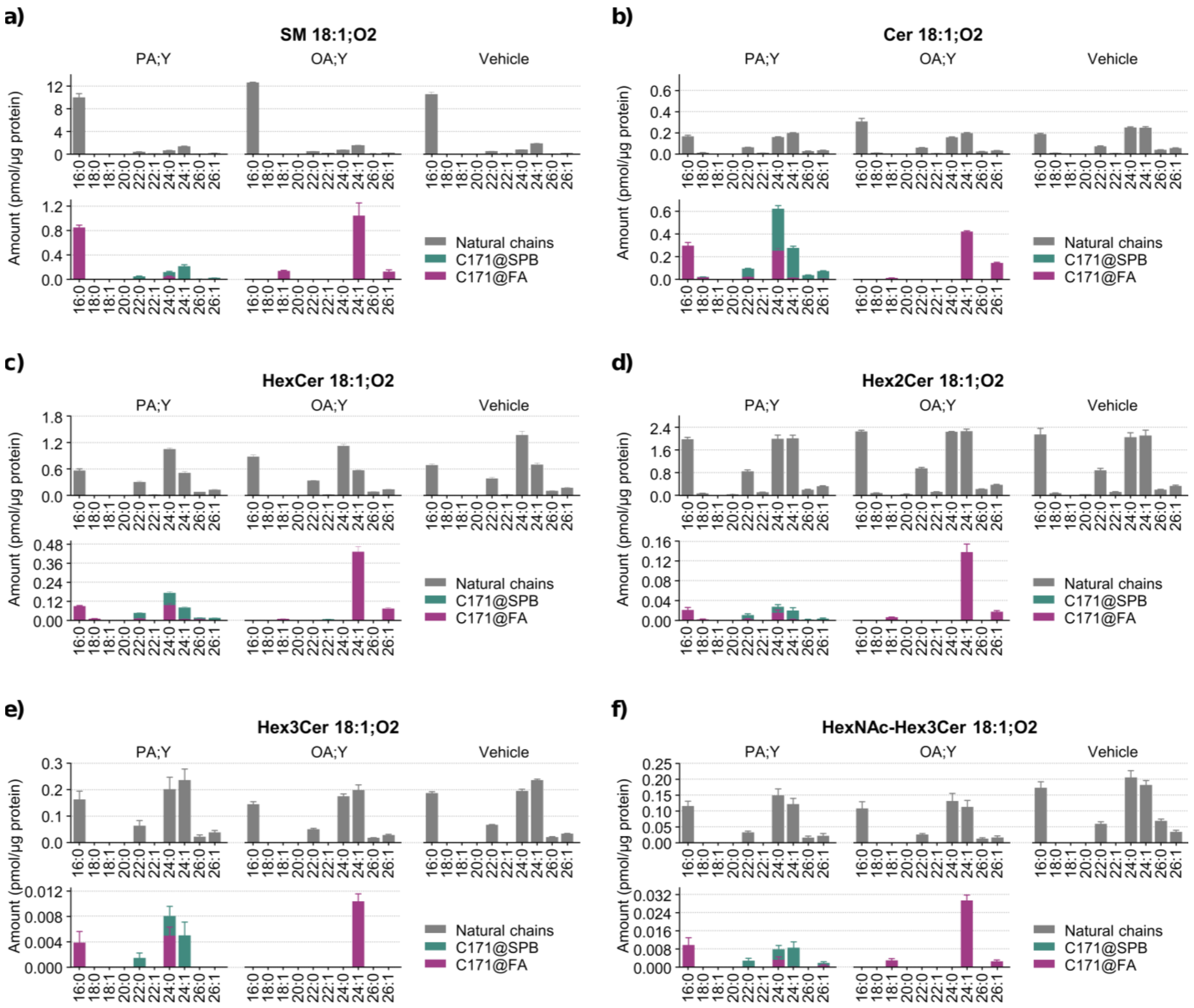

Figure S5. Integrated view of the natural and labelled sphingolipidome with chain lengths distribution in endogenous (top; grey) and C171-labelled (bottom; C171-clicked FA – purple; C171-clicked SPB - turquoise) sphingolipidome of HT1080 cells treated with PA;Y (left), OA;Y (middle) or Vehicle (right). Bar plots show quantitative fatty acyl profiles of SPB 18:1;O2-containing sphingomyelins (SM; a), ceramides (Cer; b), hexosylceramides (HexCer; c), dihexosylceramides (Hex2Cer; d), trihexosylceramides (Hex3Cer; e), and N-acetylhexosyl trihexosylceramides (HexNAc-Hex3Cer, f). Values represent mean ± s.d., n = 3.

Experimental Section

Chemicals

Chemical	Supplier	Catalog Number
1,4-Butanediol	TCI Europe	130680
27-alkyne cholesterol	Avanti Polar Lipids	00143P
2-Propanol (*i*-PrOH, ULC/MS-CC/SFC grade)	Biosolve B.V.	162641
3,4-Dihydro-2*H*-pyran	TCI Europe	D0555
Acetonitrile (CH_3CN, ULC/MS-CC/SFC grade)	Biosolve B.V.	012041
Acetic acid (AcOH)	Sigma-Aldrich	33209-M
Acetyl chloride	Fluorochem	094104
Aluminium chloride anhydrous ($AlCl_3$)	Fluorochem	044715
Amberlyst A26 hydroxide form	Sigma-Aldrich	542571
Ammonium acetate (CH_3COONH_4)	Sigma-Aldrich	A1542
Ammonium bicarbonate (NH_4HCO_3)	Carl Roth	7094
Ammonium formate ($HCOONH_4$, LiChropur)	Supelco	70221
Ammonium tetrafluoroborate (NH_4BF_4)	Sigma-Aldrich	223727
Arachidonic acid alkyne (AA;Y)	Cayman Chemical	10538
Bovine Serum Albumin (BSA)	Sigma-Aldrich	A7906
Butylhydroxytoluene (BHT)	SAFC	817074
C6 ceramide alkyne (Cer 18:1;O2,Y/6:0)	Cayman Chemical	24519
Cer/Sph Mixture I (Cer/Sph Mix I)	Avanti Polar Lipids	LM6002
Chloroform ($CHCl_3$, EMSURE ACS, ISO, Reag. Ph Eur)	Supelco	102445
Dichloromethane (DCM, ACS reagent, reag. ISO)	Sigma-Aldrich	32222
Dichloromethane anhydrous (DCM dry, Extra Dry over Molecular Sieve, Stabilized, AcroSeal™)	Thermo Fisher	348461000
Diethyl ether (DEE, ACS reagent, ISO, reag. Ph. Eur.)	Sigma-Aldrich	32203
Dulbecco's phosphate-buffered saline (DPBS)	Gibco	14190136
Ethanol (EtOH, LiChrosolv)	Supelco	111727
Ethyl acetate (ACS reagent, reag. ISO)	Sigma-Aldrich	33211
Fetal Bovine Serum (FBS)	Gibco	A5256701
Formic acid (HCOOH, ULC/MS-CC/SFC grade)	Biosolve B.V.	069141
Linoleic acid alkyne (LA;Y)	Cayman Chemical	10541
Methanol (MeOH, ULC/MS-CC/SFC grade)	Biosolve B.V.	136841
Methanol (MeOH, ACS reagent, reag. ISO)	Sigma-Aldrich	32213
Methanol anhydrous (MeOH dry, Extra Dry over Molecular Sieve, AcroSeal™)	Thermo Fisher	364391000
Methanesulfonyl chloride	Sigma-Aldrich	471259
Methyl iodide	Sigma-Aldrich	67692
N-Ethylmethylamine	TCI Europe	E0729
N,N-Dimethylformamide anhydrous (DMF dry)	Sigma-Aldrich	5.89565
Oleic acid alkyne (OA;Y)	Cayman Chemical	9002078
Palmitic acid alkyne (PA;Y)	Cayman Chemical	13266
PC alkyne (PC 16:0;Y/18:1)	Avanti Polar Lipids	900413P
PE alkyne (PE 16:0;Y/18:1)	Avanti Polar Lipids	900414P
Penicillin-Streptomycin	Gibco	15140122
Petroleum ether (PEE, ACS reagent, reag. ISO)	Sigma-Aldrich	32299
Plasmax™ cell culture medium	Ximbio	156371
SDS ultra pure	Carl Roth	2326
Sodium azide	Sigma-Aldrich	S2002
Sodium hydroxide	Sigma-Aldrich	30620
SPLASH LipidoMIX Mass Spec Standard (SPLASH)	Avanti Polar Lipids	330707
SYPRO™ Ruby Protein Blot Stain	Invitrogen	S11791
Tetrahydrofuran anhydrous (THF dry, inhibitor-free)	Sigma-Aldrich	5.89568
Tetrakis(acetonitrile)copper(I) tetrafluoroborate (Cu(I)TFB)	Thermo Scientific Chemicals	398760010
Triethylamine	TCI Europe	T0424
Tris ultrapure (Tris-HCl)	PanReac AppliChem ITW Reagents	A10861000
Trypsin-EDTA, 0.05%	Gibco	25300062
Urea	Carl Roth	2317
Water (H_2O, ULC/MS-CC/SFC grade)	Biosolve B.V.	232141

Cell Line

HT1080 human fibrosarcoma cells (ACC 315) were obtained from Leibniz Institute DSMZ. The cell line was maintained in Plasmax^{TM} supplemented with 10% FBS and 100 U(µg)/mL penicillin/streptomycin at 37°C with 5% CO_2.

Cell treatment and collection

$4.5 \cdot 10^4$ cells per well were seeded in 1.5 mL of growth medium in 12-well plates 21 h before the experiment. After 21 h, the medium was replaced with fresh medium containing either vehicle (0.4 vol % EtOH), PA;Y or OA;Y (100 µM in 0.4 vol % EtOH), and incubated for 4 h at 37°C, 5% CO_2. Cells were washed once with 1.5 mL DPBS (supplemented with 1 µg/mL BHT) and incubated with 0.1 mL Trypsin-EDTA for 2 min at 37°C, then 0.5 mL of the growth medium was added to stop trypsinization, the cell suspension was collected into a 2 mL Eppendorf tube, centrifuged at 500*g* for 15 min, washed once with 0.6 mL of ice-cold 155 mM CH_3COONH_4 (supplemented with 1 µg/mL BHT), and centrifuged at 500*g* for 15 min. The washed cell pellet was resuspended in 50 µL H_2O (supplemented with 1 µg/mL BHT) and stored overnight at -20°C prior lipid extraction.

To obtain LC-MS2 data for a wide range of alkyne-labeled complex lipids, similar treatment and collection were performed using all available FA;Y standards (25 µM, 4 h incubation).

Lipid extraction

20 µL of Master Internal Standard Mix (Master IS Mix) in $CHCl_3$:MeOH (1:1, v/v) was added to the sample in a 2 mL Eppendorf tube and left on ice for 15 min. For each sample, the amount of lipids in Master IS Mix was equivalent to 1.5 µL of 1X SPLASH, 1.5 µL of 1X Cer/Sph Mix I, 15 µL of 1X Click Internal Standard Mix (Click IS Mix[1]), and 50 pmol of Cer d18:1;Y/6:0. A complete description of the Master IS Mix is provided in Table S2.

Lipids were extracted following Folch's solvent formulation[2]. All solvents used for lipid extraction contained 1 µg/mL BHT. 365 µL ice-cold MeOH was added, the sample was vortexed for 10 s, then 740 µL of ice-cold $CHCl_3$ was added, vortexed for 10 s, and the sample was incubated for 1 h in a rotary shaker (40 rpm) at 4°C. Phase separation was induced by adding 225 µL of H_2O, the sample was vortexed for 10 s and centrifuged at 10000*g* for 5 min. 770 µL of the lower phase was transferred to a 1.5 mL Eppendorf tube and dried in a vacuum concentrator at 40 mbar and 20°C. Meanwhile, lipids from the remaining upper phase were re-extracted by adding 400 µL of $CHCl_3$:MeOH (2:1, v/v), vortexed for 10 s, 100 µL H_2O was added to induce phase separation, vortexed for 10 s, centrifuged at 10000*g* for 5 min, and 380 µL of the lower phase was transferred to the 1.5 mL Eppendorf tube containing the first extract portion and the sample was continued to dry in the vacuum concentrator. The dried lipid extract was stored at -80°C until derivatization. The upper phase and interphase remained after extraction were dried in the vacuum concentrator at 20 mbar and 20°C and used for total protein quantification.

Click reaction, lipid extraction and sample preparation for LC-MS

All solvents used for the click reaction and subsequent extraction contained 1 µg/mL BHT. $CHCl_3$ (8 µL) was added and the tubes sonicated on a water bath for 30 s. Working solution of Click Mix was prepared by mixing 10 µL of 48.5 mM $C171^+BF_4^-$ (C171; stored as aliquots in MeOH:H_2O (1:1, v/v) at -80°C), 48.5 µL of 10 mM Cu(I)TFB (stored as N_2-deaerated aliquots in CH_3CN at -80°C) and 265 µL of N_2-purged EtOH. 40 µL of working solution of Click Mix was added (1.5 mM $C171^+BF_4^-$, 1.5 mM Cu(I)TFB), followed by sonication for 30 s and incubation at 40°C for 16 h. The reaction tubes were cooled to room temperature, then 200 µL of ice-cold $CHCl_3$ and 200 µL of H_2O were added, the samples vortexed for 30 s and centrifuged at 20000*g* for 5 min at room temperature. 200 µL of the lower phase was transferred to a 0.5 mL Eppendorf tube and dried in the vacuum concentrator at 40 mbar and 20°C. The resulting lipid extract was reconstituted in 60 µL of *i*-PrOH (without BHT), vortexed for 30 s, centrifuged at 10000*g* for 5 min, and 50 µL was transferred to a glass insert for LC-MS analysis. Individual alkyne lipid standards (40 nmol per click reaction) were derivatized in a similar manner.

LC-MS

Reversed-phase ultra-high-performance LC (RP-UHPLC) was performed on a Vanquish Horizon (Thermo Fisher Scientific) equipped with an Accucore C30 column (150 × 2.1 mm, 2.6 µm, 150 Å, Thermo Fisher Scientific). 6 µL (positive mode) or 12 µL (negative mode) of lipid sample injected onto the column was separated by gradient elution with solvent A (CH_3CN:H_2O, 1:1, v/v) and B (*i*-PrOH:CH_3CN:H_2O, 85:10:5, v/v/v) both containing 5 mM $HCOONH_4$ and 0.1 vol % HCOOH. Separation was performed at 50°C at a flow rate of 0.3 mL/min using the following gradient: 0-5 min — 10-12.5% B, 5-20 min — 12.5-80% B, 20-24 min — 80–95% B, 24-27 min — 95-100% B, 27-32 min — 100% B (isocratic), 32-32.1 min —100-10% B, 32.1-40.0 min — 10% B (isocratic, column re-equilibration).

Mass spectrometry was performed on Thermo Scientific Orbitrap Exploris 240 (Thermo Fisher Scientific) equipped with a heated electrospray ionization (HESI) source with an EASY-IC unit for lock mass correction and operated with the following global HESI parameters: sheath gas 40 arbitrary units, auxiliary gas 10 arbitrary units, sweep gas 1 arbitrary units, spray voltage 3.5 kV (positive mode) or 2.5 kV (negative mode), ion transfer tube temperature 300°C, vaporizer temperature 370°C, S-lens RF level 35%, EASY-IC lock mass correction was set to RunStart.

<u>For annotation of clicked and non-derivatized lipids</u>, MS data were recorded in positive and negative ionization modes with the instrument operating in data dependent acquisition (DDA) mode with a cycle time of 1.3 s. Full scans (MS1) had the following settings: Orbitrap resolution 120000 at *m/z* 200, scan range *m/z* 200-1200 (positive mode) or 200-1000 (negative mode) within 0-20 min, and 600-1700

(positive mode) or 500-1600 (negative mode) within 20-40 min, absolute AGC value 1·10^6, maximum injection time 100 ms, 1 microscan. DDA MS2 (ddMS2) scans had the following settings: 1.2 *m/z* precursor selection isolation window, Orbitrap resolution 15000 at *m/z* 200, stepped higher-energy collisional dissociation (HCD) at normalized collision energies of 27-37-47% (positive mode) or 17-27-37% (negative mode) within 0-20 min, and 30-40-50% (positive mode) or 17-27-37% (negative mode) within 20-40 min, absolute AGC value 1·10^5, maximum injection time 60 ms, 1 microscan. The following filters were applied prior to ddMS2 scans: dynamic exclusion after 4 (positive mode) or 3 (negative mode) times if occurring within 6 s, exclusion duration 6 s, mass tolerance ±5 ppm, isotope exclusion; allowed charge state 1-3 (positive mode) or 1 (negative mode); targeted mass exclusion using the mass list of contaminants detected in blank samples (positive mode only), retention time window-assigned, exclusion mass tolerance ±5 ppm. Data were acquired in profile mode. To obtain MS2 spectra and confirm the annotation of derivatized glycolipids whose single-charged ions had *m/z* greater than 1700, an additional DDA experiment was performed in positive mode. All settings were the same as described above, but the *m/z* range was 200-1000 (LC-MS run 1) and 1000-1800 (LC-MS run 2).

For lipid quantification, full scan MS acquisition was performed in positive ionization mode with the following instrument settings: Orbitrap resolution 90000 at *m/z* 200, scan range *m/z* 250-1550 within 0-40 min, absolute AGC value 1·10^6, maximum injection time set to auto, 1 microscan. Data were acquired in profile mode.

Total protein quantification

Total protein content was determined following a SYPRO™ Ruby Protein Blot Stain assay[3]. Briefly, dried aqueous- and interphase after lipid extraction was dissolved in 70 µL of Lysis Buffer (7 M urea, 2% SDS, in 50 mM Tris-HCl, pH 7.6), vortexed and sonicated for 30 s. 20 µL of the solution was mixed with 20 µL of freshly prepared 3 mM NH_4HCO_3 and 1 µL of this solution was spotted in triplicate onto a 0.2 µm Amersham Protran nitrocellulose membrane. A calibration curve was prepared using BSA dissolved in Lysis Buffer and diluted with 3 mM NH_4HCO_3 (1:1, v/v) to final concentrations of 0.125-1.5 µg/µL (7-point calibration). 1 µL of each calibration standard was spotted in triplicate on the same membrane. The membrane was air-dried for 10 minutes before staining, then placed in a glass Petri dish and immersed in 7% (v/v) AcOH in MeOH:H_2O 1:9 (v/v) for 15 min at room temperature with gentle agitation. It was then washed three times with water (10 min each) and incubated for 40 min in the dark in a SYPRO Ruby blot stain reagent under gentle agitation at room temperature. After staining, the membrane was rinsed three times with water (5 min each rinse). Fluorescence signal was imaged using a ChemiDoc MP Imaging System (Bio-Rad) with excitation at 520–545 nm and emission at 605 nm, and spot intensities were quantified using Image Lab (Bio-Rad Laboratories, version 6.0.1b34). Protein concentrations in the samples were calculated relative to the BSA calibration curve. The calibration by BSA and determined total protein quantities including with standard error of mean (s.d.) and coefficient of variation (CV, %) are presented below.

Calibration by BSA with linear regression fit

c(BSA), µg/µL	Replicate	0.0625	0.125	0.25	0.50	0.75		R^2	Slope	Intercept
Intensity, arb. units	1	4.51E7	9.29E7	1.44E8	2.83E8	4.27E8				
	2	4.70E7	9.05E7	1.60E8	2.82E8	4.10E8		0.9978	5.38E8	1.62E7
	3	4.10E7	8.19E7	7.94E7	2.87E8	4.10E8				

		**PA;Y-treated cells**			**OA;Y-treated cells**			**Vehicle-treated cells**		
		sample 1	sample 2	sample 3	sample 1	sample 2	sample 3	sample 1	sample 2	Sample 3
Intensity, arb. units	1	2.69E8	2.26E8	2.52E8	2.43E8	2.96E8	3.02E8	3.41E8	3.48E8	3.15E8
	2	2.77E8	2.29E8	2.55E8	2.24E8	2.97E8	2.96E8	3.38E8	3.46E8	3.26E8
	3	2.93E8	2.39E8	2.86E8	2.35E8	2.97E8	3.08E8	3.44E8	3.49E8	3.31E8
c(Protein), µg/µL	1	0.47	0.39	0.44	0.42	0.52	0.54	0.61	0.62	0.56
	2	0.49	0.40	0.45	0.39	0.52	0.52	0.60	0.62	0.58
	3	0.52	0.42	0.51	0.41	0.53	0.55	0.61	0.62	0.59
Total protein amount, µg	1	49.7	41.3	46.3	44.5	55.0	56.3	63.9	65.2	58.6
	2	51.3	41.9	47.0	40.7	55.1	55.0	63.3	64.9	60.9
	3	54.4	43.7	53.1	42.9	55.2	57.2	64.4	65.5	61.9
	Mean	51.8	42.3	48.8	42.7	55.1	56.2	63.9	65.2	60.5
	s.d.	1.69	0.96	3.12	1.20	0.04	1.11	0.54	0.30	0.75
	CV, %	3.26	2.27	6.40	2.81	0.08	1.97	0.85	0.46	1.24

Database construction in Lipostar2 Database Manager

The Lipid Builder tool in Lipostar2 was modified to work with clicked chains. First, an intervention in lipid nomenclature was required to easily distinguish labeled chains from natural ones. To this end, the prefix "click-C171-" was used for C171-labeled chains so that additional MS reporter structures could be used in the future. The database was constructed based on the combinations described in Table S6. In general, complete conversion to label-saturated lipid species (e.g., triacylglycerols (TGs) bearing three C171 chains, or diacylglycerophosphocholines (PCs) endowed with two C171 chains) was allowed. Constraints were imposed on TGs and cardiolipins (CLs) to reduce the number of possible combinations (described in Table S6). This resulted in a database of 224514 structures (Clicked

Lipid Search Space), including isotopically labelled standards from Click IS Mix. A tailored nomenclature was adapted for labelled species where C171-clicked lipids carry a constant positive charge are denoted as "cM" (Table S1).
The fragmentation rules obtained by manually interpreting the MS2 spectra representing each subclass of C171-derivatized lipids (Files S1 and S2, Tables S4 and S5) were converted to the proprietary Lipostar2 format and used for in silico fragmentation of the structural database, resulting in a complete Clicked Lipids Database to be used for the MS2-based identification of clicked lipids.

Identification of C171-derivatized lipids in Lipostar2 using DDA acquisition

Raw files were uploaded to Lipostar2 (Mass Analytica, version 2.1.8b13) and processed using the LC-MS settings specified in Table S7. The data matrices were filtered using a retention time (RT) filter (0.8-34 min) and a Super Sample filter (only lipids with isotopic pattern were retained). Lipids were identified using adducts and RT range information specified in Table S7. Unidentified features were filtered out, and the remaining features with 4 and 3 stars were automatically approved, including related adducts and in-source fragmentation (ISF) ions. The bulk assessment of automatic approvals was performed using Kendrick mass defect (KMD) plots for each subclass and adduct type, and outliers from the RT trends were disapproved. After manual curation of the remaining automatic approvals, adduct clustering was performed with an RT tolerance of 0.2 min, including ISF search.

Relative lipid quantification strategy using full scan MS acquisition

Identification results from Lipostar2 were exported as a .csv file and used to generate an ion list for quantification in full scan MS-acquired raw files using Skyline (MacCoss Lab, version 24.1.0.199). Monoisotopic *m/z* values, including the complete ion sets (different adducts and in-source fragmentation-derived ions, as detailed in Table S7), along with corresponding RTs, were imported into Skyline as a "transition list". Transition settings were configured as follows: ion type – *p* (precursor), min *m/z* 250, max *m/z* 1550, method match tolerance 0.005 *m/z*, MS1 filtering by isotope peaks (count, 1 peak), precursor mass analyzer – centroided, mass accuracy 5 ppm, isotope labelling enrichment – default; MS2 filtering – none, no retention time filtering (all matching scans included). Raw data files were then imported, and extracted ion chromatogram peaks were integrated. The resulting data matrix was exported as a .csv file and further processed in Microsoft Excel. For each lipid, areas under the curve (AUC) of all detected ions were summed.
Quantification was performed using a one-per-class internal standard (ISTD) approach. The summed AUC for each lipid was divided by the summed AUC of the corresponding class-specific internal standard (ISTDs used for the quantification are specified in Tables S9 and S10) and multiplied by the ISTD amount. Final values were normalized to total protein content and expressed in pmol/µg protein.

Assessment of lipid recovery after extraction from the click reaction mixture

Bulk quantities of C171-derivatized lipid standards were generated by performing the click reaction on alkyne lipid standards in the absence of cellular lipid matrix, following the protocol described above. Specifically, 200 µL of 1X Click IS Mix (equivalent to 19.3 nmol of clickable alkyne groups) and 1.34 nmol of Cer 18:1;O2,Y/6:0 were used for the reaction. Half of the resulting derivatized product (equivalent to 100 µL of 1X Click IS Mix) was combined with an equimolar amount of the alkyne (non-clicked) version of the Click IS Mix (100 µL), 1.34 nmol of Cer 18:1;O2,Y/6:0, 10 µL of 1X SPLASH, and 10 µL of Cer/Sph Mix I. The mixture was dried in the vacuum concentrator and reconstituted in 200 µL of $CHCl_3$:MeOH (1:1, v/v), forming a Master Mix that contained C171-derivatized, alkyne, and non-alkyne lipid standards for the recovery experiment (a complete description with quantities is provided in Table S2). To prepare the cell lipid matrix, lipids were extracted from ~$3 \cdot 10^6$ HT1080 cells using the Folch method as described above. The extract was dried in the vacuum concentrator and reconstituted in 500 µL of $CHCl_3$:MeOH (1:1, v/v). For each sample, 50 µL of the lipid extract was transferred to a 1.5 mL Eppendorf tube.
For the "spiked before extraction" and "spiked after extraction" groups, 20 µL of the Master Mix or 20 µL of $CHCl_3$:MeOH (1:1, v/v) was added to the cell lipid matrix, respectively. All samples were dried under a vacuum, derivatization step was performed as described above but in the absence of the C171 reagent. Samples were extracted using $CHCl_3$:H_2O (1:1; v/v), and 20 µL of solvent ($CHCl_3$:MeOH, 1:1, v/v) or 20 µL of the Master Mix, were added to the "spiked before extraction" and "spiked after extraction" samples, respectively.
All samples were dried under vacuum, reconstituted in 50 µL of *i*-PrOH, vortexed for 30 s, centrifuged at 10,000*g* for 5 min, 45 µL was transferred to glass inserts for LC-MS analysis, of which 2.5 µL was used for the analysis. Full scan MS data (positive ionization mode) were acquired using the LC-MS method described before. The experiment was conducted in triplicates.
Lipid recovery was calculated according to the following formula:

$$Recovery\ (\%) = \frac{\sum_i I^{norm}_{spiked\ before\ extration}}{\sum_i I^{norm}_{spiked\ after\ extraction}} \cdot 100\%,$$

where:
$\sum_i I$ is the sum of monoisotopic peak areas for all detected adducts of a given lipid, and

$$\sum_i I^{norm} = \frac{\sum_i I}{NF} \text{ with } NF = \frac{\sum_i I(PC\ 16{:}0\_18{:}1)}{\max\left(\sum_i I\ (PC\ 16{:}0\_18{:}1)\right)}.$$

The normalization factor (NF) is based on the endogenous PC 16:0_18:1, compensating for slight variability of organic phase collection during extraction across replicates.

Analytical method validation through multipoint external calibration

A Master Mix containing alkyne and non-alkyne lipid standards was prepared in $CHCl_3$:MeOH (1:1, v/v) by combining 600 µL of 1X Click IS Mix, 2 nmol of Cer 18:1;O2,Y/6:0, 60 µL of 1X SPLASH, and 60 µL of Cer/Sph Mix I. The mixture was dried in a vacuum concentrator and reconstituted in 300 µL of $CHCl_3$:MeOH (1:1, v/v). This stock solution served as the highest concentration level in a 10-point dilution

series (including just solvent, 0 point), prepared by sequential 1:1 (v/v) dilution with $CHCl_3$:MeOH (1:1, v/v), except for the final dilution step, which was performed at 1:2.5 ratio (detailed in Table S2).
For each concentration level, 20 µL of Master Mix was spiked into HT1080 cell pellets (~$0.3 \cdot 10^6$ cells/sample) in 2 mL Eppendorf tubes (50 µL aliquots of cell suspension in H_2O was aliquoted from a single stock). Each concentration was processed in two ways (in triplicates): three samples underwent the full derivatization protocol with C171 reagent (C-set, 30 samples total), and three samples followed the same workflow without the C171 reagent (Y-set, 30 samples total).
All samples were extracted using the Folch protocol described above. The C-set was subjected to derivatization with C171, while the Y-set underwent the same protocol without the reagent. After the click reaction, lipids were re-extracted from the click reaction mixture, dried, reconstituted in 50 µL of *i*-PrOH, vortexed for 30 s, centrifuged at 10000*g* for 5 min, 45 µL was transferred to glass LC-MS inserts, and 1 µL was used for the analysis. Full scan MS data were acquired using the LC-MS method described above. Samples were injected in ascending concentration order, with *i*-PrOH blanks run in between to monitor potential carryover.
The normalized signal (obtained in the same manner as described for the lipid recovery experiment) was plotted as a function of the absolute amount (in pmol) loaded onto the LC column. A univariate linear regression $y = ax + b$ (where y is the normalized signal and x is the analyte amount, a is the regression slope and b is the y intercept) was fitted to calibration data points using the widest concentration range in which the regression coefficient of determination (R^2) exceeded 0.99. The zero-concentration point was not included in the model fitting. All regression analyses were conducted at a 95% confidence level, following IUPAC guidelines for figures of merit in univariate calibration[1].
Sensitivity was defined as the slope (a) of the linear regression, representing the change in analytical signal per unit analyte amount.
Limit of detection (LOD) was estimated according to the IUPAC-recommended formula:

$$LOD = \frac{3.3 \cdot s_x}{a} \cdot \sqrt{1 + h_0},$$

where s_x is the standard error of regression, a is the slope of the calibration line, and h_0 is the leverage at zero concentration, calculated as:

$$h_0 = \frac{1}{C} + \frac{\bar{x}^2}{\sum(x_i - \bar{x})^2},$$

with C being the number of unique calibration levels, $\bar{x}$ the mean of calibration concentrations, and x_i the individual calibration levels.
Limit of quantification (LOQ) was derived as:

$$LOQ = 3 \cdot LOD.$$

Sensitivity gain was calculated to assess the analytical advantage of C171 derivatization, using the ratio of slopes between the C171-labelled (a_{C171}) and unlabelled (parent alkyne, a_Y) lipid standards:

$$Sensitivity\ gain = \frac{a_{C171}}{a_Y}.$$

All calculations and regression diagnostics were performed in Microsoft Excel using Data Analysis Add-in (detailed in Table S8).

Statistical analysis
Statistical testing was performed using GraphPad Prism (GraphPad Software, version 10.4.2.633). For comparison of lipid abundances across experimental groups, one-way ANOVA was used followed by post hoc testing. Normality and homogeneity of variances were assessed using the Brown–Forsythe test. Multiple comparisons between groups were evaluated using the two-stage linear step-up procedure of Benjamini, Krieger, and Yekutieli to control the false discovery rate (FDR, Q = 5%). Individual p-values were calculated without assuming equal standard deviations. Differences were considered statistically significant at $p < 0.05$.

General synthetic chemistry methods
Unless otherwise noted, starting materials, reagents, and solvents were purchased from commercial suppliers and used without further purification. All air- and/or moisture-sensitive reactions were performed under a nitrogen atmosphere using commercially available anhydrous solvents (Thermo Fisher, Sigma-Aldrich) and standard Schlenk techniques. The glassware used for these reactions was flame-dried and cooled under a nitrogen flow before use.
Reactions were routinely monitored by thin-layer chromatography (TLC) on silica gel 60 F254 (layer 0.2 mm) pre-coated aluminium foil with a fluorescent indicator UV254 (Merck). Developed plates were air-dried and visualized under UV light (λ: 254/365 nm) and/or by staining and warming with potassium permanganate or ninhydrin solutions.
Automated flash chromatography was performed using Biotage® Selekt equipped with Sfär Silica HC Duo 5g or 10g cartridges.
^1H NMR and ^{13}C NMR spectra were recorded at room temperature at 400 and 101 MHz, respectively, on a Bruker Avance III HD 400 spectrometer using residual solvent peak as internal standards ($CDCl_3$: δ = 7.26 ppm ^1H NMR, δ = 77.16 ppm ^{13}C NMR; DMSO-d_6: δ = 2.50 ppm ^1H NMR, δ = 39.5 ppm ^{13}C NMR). Chemical shifts are reported in ppm (δ) and the coupling constants (*J*) are given in Hertz (Hz). Peak multiplicities are abbreviated as follows: s (singlet), bs (broad singlet), d (doublet), dd (double doublet), t (triplet), dt (double triplet), q (quartet), p (pentet), and m (multiplet).
LC-MS analysis was carried out on an Agilent 1290 HPLC system coupled to an Agilent Technologies 6540 UHD Accurate-Mass QTOF mass spectrometer (Agilent). Chromatographic conditions were as follows: Luna® Omega Polar C18 column (100 x 2.1 mm, 1.6 µm, Phenomenex); injection volume 5.00 µL; flow rate 0.6 mL/min; acquisition time 10 min; column temperature 40°C; eluent A: H_2O, eluent B: CH_3CN; gradient: 0-8 min — 0.5-95% B, 8-10 min — 95% B (isocratic), post-run 2 min.

ESI-MS conditions: *m/z* range 105-1700; scan rate 3 spectra/s; gas temperature 350°C; gas flow 9 mL/min; nebulizer pressure 35 psi; sheath gas temperature 400°C; sheath gas flow 9 mL/min; VCap 4000 V; nozzle voltage 0 V; fragmentor voltage 120 V; skimmer voltage 65 V; octupole RF peak voltage 750 V. UV detection was averaged across wavelengths from 190 nm to 640 nm.

Synthesis of C171 reagent

The MS reporter compound **C171** was synthesized using a slightly modified procedure based on the originally reported method[4], as depicted in Scheme S1. The optimized synthetic route provided the **C171** compound with an overall yield of 25%.

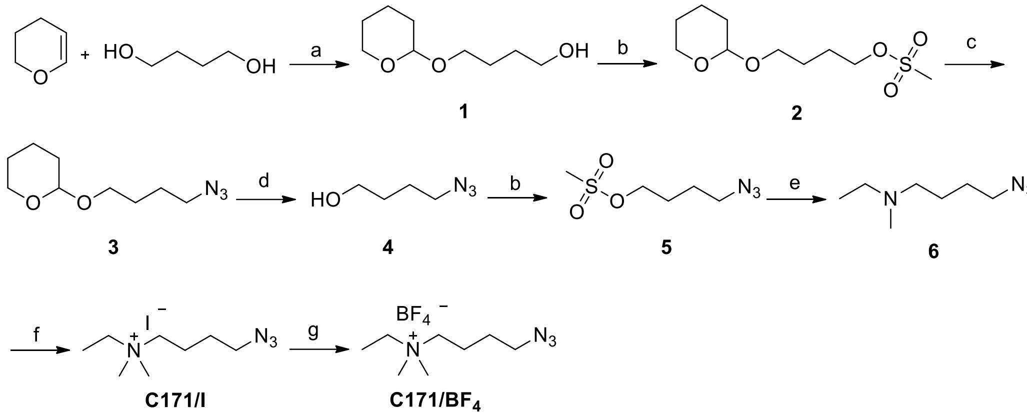

Scheme S1. Reagents and conditions: a) anhydrous $AlCl_3$, 30°C, 30 min; b) methanesulfonyl chloride, Et_3N, dry DCM, rt, 2-4 h; c) NaN_3, dry DMF, 60°C, 16 h; d) acetyl chloride, dry MeOH, rt, 30 min; e) *N*-methylethanamine, dry THF, 60°C, 6 h; f) methyl iodide, dry DCM, rt, 6 h; g) anion exchange by Amberlyst A26 hydroxide form, NH_4BF_4, MeOH/H_2O 50%.

Commercially available butane-1,4-diol was first monoprotected by reaction with 3,4-dihydro-2*H*-pyran in the presence of aluminium trichloride ($AlCl_3$), as previously described[5], to afford intermediate **1**. The subsequent reaction with methanesulfonyl chloride in the presence of triethylamine (Et_3N) yielded mesylate derivative **2**, which was then reacted with sodium azide (NaN_3) to give the azido derivative **3**. Deprotection of **3** with acetyl chloride furnished intermediate **4**, which, after reaction with methanesulfonyl chloride, produced mesylate derivative **5**. Substitution of the mesylate group in **5** with *N*-ethylmethylamine led to intermediate **6**, and its reaction with methyl iodide yielded **C171**, as iodide salt (**C171/I**). Finally, the iodide anion was exchanged with tetrafluoroborate (BF_4^-) by Amberlyst A26 hydroxide resin to give the final compound **C171/BF$_4$**, which was used in CuAAC reactions.

*Synthesis of 4-((Tetrahydro-2H-pyran-2-yl)oxy)butyl methanesulfonate (Intermediate **2**)*

Under a nitrogen atmosphere, to a solution of **1**[5] (0.162 g, 0.929 mmol) in dry DCM (4.6 mL) cooled to 0°C, Et_3N (0.19 mL, 1.395 mmol) and a solution of methanesulfonyl chloride (0.079 mL, 1.022 mmol) in dry DCM (0.4 mL) were added. The reaction was stirred at room temperature for 2 h. The reaction mixture was quenched with 5% citric acid solution (0.5 mL), and the organic phase was washed with water (1 × 5 mL), brine (1 × 5 mL), dried over Na_2SO_4, filtered, and evaporated to dryness to afford the titled compound as a viscous colourless oil (0.185 g, 79% yield). ^1H NMR (400 MHz, $CDCl_3$) δ 4.61 – 4.53 (m, 1H), 4.28 (t, *J* = 6.5 Hz, 2H), 3.89 – 3.74 (m, 2H), 3.54 – 3.39 (m, 2H), 3.00 (s, 3H), 1.93 – 1.58 (m, 8H), 1.55 – 1.51 (m, 2H). ^{13}C NMR (101 MHz, $CDCl_3$) δ 98.99, 70.01, 66.64, 62.47, 37.36, 30.68, 26.32, 25.70, 25.39, 19.61.

*Synthesis of 2-(4-Azidobutoxy)tetrahydro-2H-pyran (Intermediate **3**)*

Under a nitrogen atmosphere, to a solution of **2** (0.180 g, 0.713 mmol) in dry DMF (1.35 mL), NaN_3 (0.134 g, 2.069 mmol) was added, and the reaction was stirred at 60°C for 16 h. After cooling to room temperature, the reaction mixture was diluted with water (30 mL) and extracted with EA (3 × 15 mL). The combined organic phases were washed with water (3 × 20 mL), brine (20 mL), dried over Na_2SO_4, filtered, and evaporated to dryness to afford the titled compound as a colourless oil (0.128 g, 90% yield). ^1H NMR (400 MHz, $CDCl_3$) δ 4.63 – 4.53 (m, 1H), 3.91 – 3.73 (m, 2H), 3.58 – 3.38 (m, 2H), 3.37 – 3.27 (m, 2H), 1.88 – 1.66 (m, 6H), 1.61 – 1.50 (m, 4H). ^{13}C NMR (101 MHz, $CDCl_3$) δ 98.90, 66.80, 62.38, 51.35, 30.72, 26.93, 25.96, 25.46, 19.63.

*Synthesis of 4-Azidobutan-1-ol (Intermediate **4**)*

Under a nitrogen atmosphere, to a solution of **3** (0.120 g, 0.602 mmol) in dry MeOH (4.0 mL) cooled to 0°C, acetyl chloride (0.024 mL, 0.337 mmol) was added dropwise, and the reaction was stirred at room temperature for 30 min. The reaction mixture was evaporated to dryness and then purified by automated flash chromatography on a SiO_2 cartridge (eluent: PET/EA, 90:10 to 50:50) to afford the titled compound as a colourless oil (0.038 g, 55% yield). ^1H NMR (400 MHz, $CDCl_3$) δ 3.69 (t, *J* = 6.1 Hz, 2H), 3.33 (t, *J* = 6.4 Hz, 2H), 1.76 – 1.60 (m, 4H). ^{13}C NMR (101 MHz, $CDCl_3$) δ 62.25, 51.31, 29.80, 25.42.

*Synthesis of 4-Azidobutyl methanesulfonate (Intermediate **5**)*

Under a nitrogen atmosphere, to a solution of **4** (0.032 g, 0.278 mmol) in dry DCM (1.3 mL) cooled to 0°C, Et_3N (0.058 mL, 0.417 mmol) and methanesulfonyl chloride (0.024 mL, 0.306 mmol) were added. The reaction was stirred at room temperature for 4 h. The mixture was quenched with 5% citric acid solution (0.2 mL), diluted with DCM (2.0 mL), washed with water (1 × 5 mL), brine (1 × 4 mL), dried over Na_2SO_4, filtered, and evaporated to dryness to afford the titled compound as a colourless oil (0.053 g, 99% yield). ^1H NMR (400 MHz, $CDCl_3$) δ 4.26 (t, *J* = 6.2 Hz, 2H), 3.36 (t, *J* = 6.5 Hz, 2H), 3.02 (s, 3H), 1.94 – 1.80 (m, 2H), 1.79 – 1.67 (m, 2H). ^{13}C NMR (101 MHz, $CDCl_3$) δ 69.05, 50.74, 37.48, 26.49, 25.10.

*Synthesis of 4-Azido-N-ethyl-N-methylbutan-1-amine (Intermediate **6**)*

Under a nitrogen atmosphere, in a 4 mL pressure tube, to a solution of **5** (0.100 g, 0.517 mmol) in dry THF (0.4 mL), *N*-ethylmethylamine (0.150 mL, 1.759 mmol) was added. The reaction was stirred at 60°C for 12 h. After cooling to room temperature, the reaction mixture was diluted with DCM (2.0 mL) and 1 N NaOH solution (1.0 mL), then extracted with DCM (3 × 2 mL). The combined organic phases were washed with water (2 × 5 mL), brine (4 mL), dried over Na_2SO_4, filtered, and evaporated under a gentle nitrogen flow at room temperature to afford the titled compound as a clear yellow oil (0.055 g, 69% yield). ^1H NMR (400 MHz, $CDCl_3$) δ 3.29 (t, *J* = 6.7 Hz, 2H), 2.45 – 2.30 (m, 4H), 2.20 (s, 3H), 1.68 – 1.48 (m, 4H), 1.05 (t, *J* = 7.2 Hz, 3H).

*Synthesis of 4-Azido-N-ethyl-N,N-dimethylbutan-1-aminium iodide (Intermediate **C171/I**)*

In a 2 mL vial, to a solution of **6** (0.0607 g, 0.388 mmol) in dry DCM (0.107 mL), methyl iodide (0.029 mL, 0.466 mmol) was added, and the reaction was stirred at room temperature for 6 h. The reaction mixture was evaporated under a gentle nitrogen flow at room temperature. Diethyl ether (DEE, 3 × 0.3 mL) was added to the crude residue; the upper phase was discarded after each wash. The remaining residue was dried under nitrogen to afford the titled compound as a clear yellow oil (0.115 g, 100% yield). ^1H NMR (400 MHz, $CDCl_3$) δ 3.67 (m, 4H), 3.48 (t, *J* = 6.4 Hz, 2H), 3.35 (s, 6H), 1.94 – 1.82 (m, 2H), 1.78 – 1.71 (m, 2H), 1.43 (t, *J* = 7.0 Hz, 3H). ^{13}C NMR (101 MHz, $CDCl_3$) δ 63.23, 60.12, 51.00 (2C), 50.57, 25.57, 20.19, 8.79. HRMS (ESI) m/z $[M]^+$ calculated for $C_8H_{19}N_4$ 171.16042, found 171.16030.

*Synthesis of 4-Azido-N-ethyl-N,N-dimethylbutan-1-aminium tetrafluoroborate (Intermediate **C171/BF$_4$**)*

For use in the click reaction labelling protocol, the iodide anion in **C171/I** was exchanged for BF_4^-. The anion exchange was performed as follows: compound **C171/I** (110 mg) was dissolved in 1.9 mL of a solution of MeOH/H_2O (50:50, v/v). The resulting solution was loaded onto a 5 mL column of Amberlyst A26 hydroxide resin (Sigma-Aldrich) that had been washed sequentially with 8 volumes each of MeOH/H_2O (50:50, v/v), 1 M NaOH, H_2O, 1 M NH_4BF_4, H_2O, and MeOH/H_2O (50:50, v/v). The column was eluted with MeOH/H_2O (50:50, v/v). The first 1.5 mL of flow-through were discarded, and the following 7.6 mL effluent were collected to obtain a 48 mM colourless solution of **C171/BF$_4$.**

LC-MS analysis of compound **C171/I**

Compound Table

Compound Label	RT	Mass	Abund	Formula	Tgt Mass	Diff (ppm)	MFG Formula	DB Formula
Cpd 1: C8 H19 N4	1.468	171.16091	563218	C8 H19 N4	171.16097	-0.34	C8 H19 N4	C8 H19 N4

Compound Label	*m/z*	RT	Algorithm	Mass
Cpd 1: C8 H19 N4	171.1603	1.468	Find By Formula	171.16091

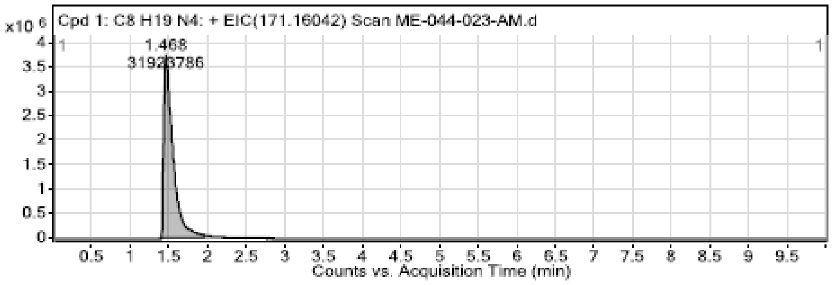

MS Spectrum

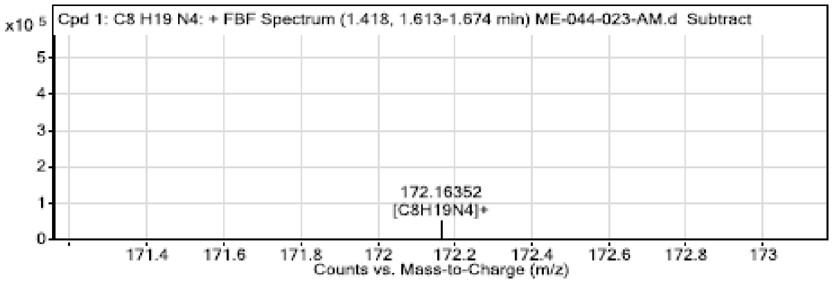

MS Zoomed Spectrum

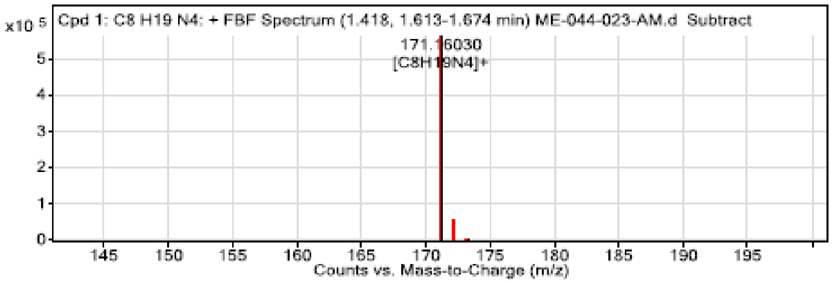

MS Spectrum Peak List

m/z	z	Abund	Formula	Ion
171.1603	1	563218.06	C8H19N4	M+
172.16352	1	51990.25	C8H19N4	M+
173.16635	1	2115.62	C8H19N4	M+

^{1}H NMR (400 MHz, $CDCl_3$) spectrum of compound **2**

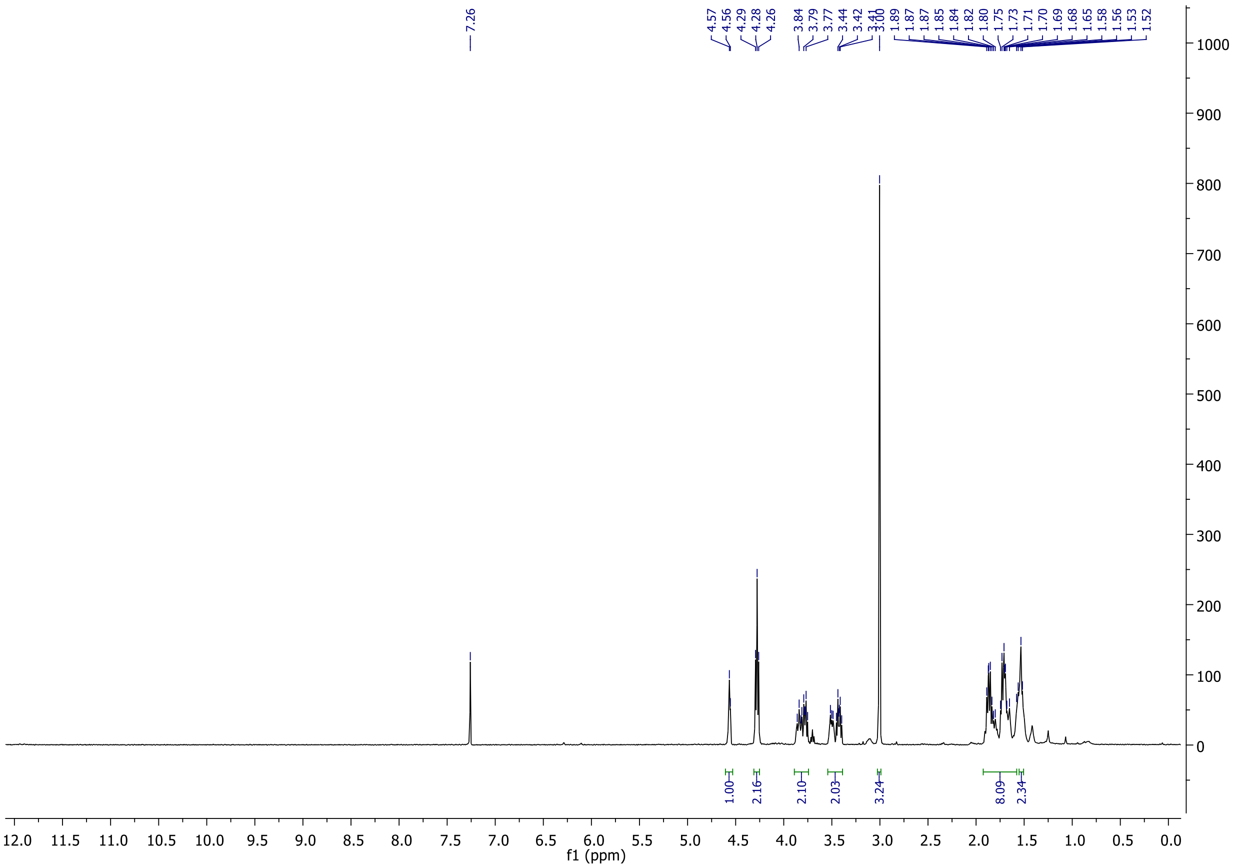

^{13}C NMR (101 MHz, $CDCl_3$) spectrum of compound **2**

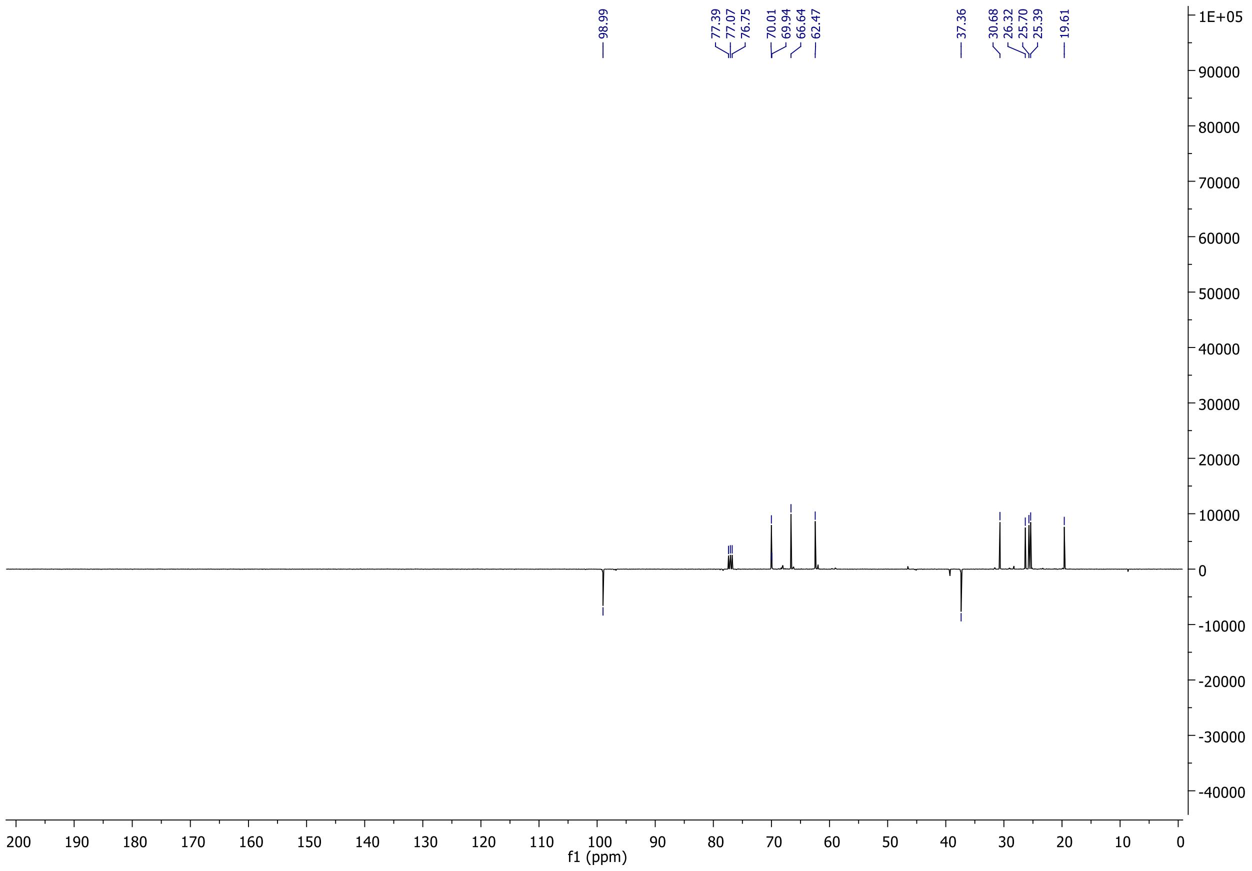

^{1}H NMR (400 MHz, $CDCl_3$) spectrum of compound **3**

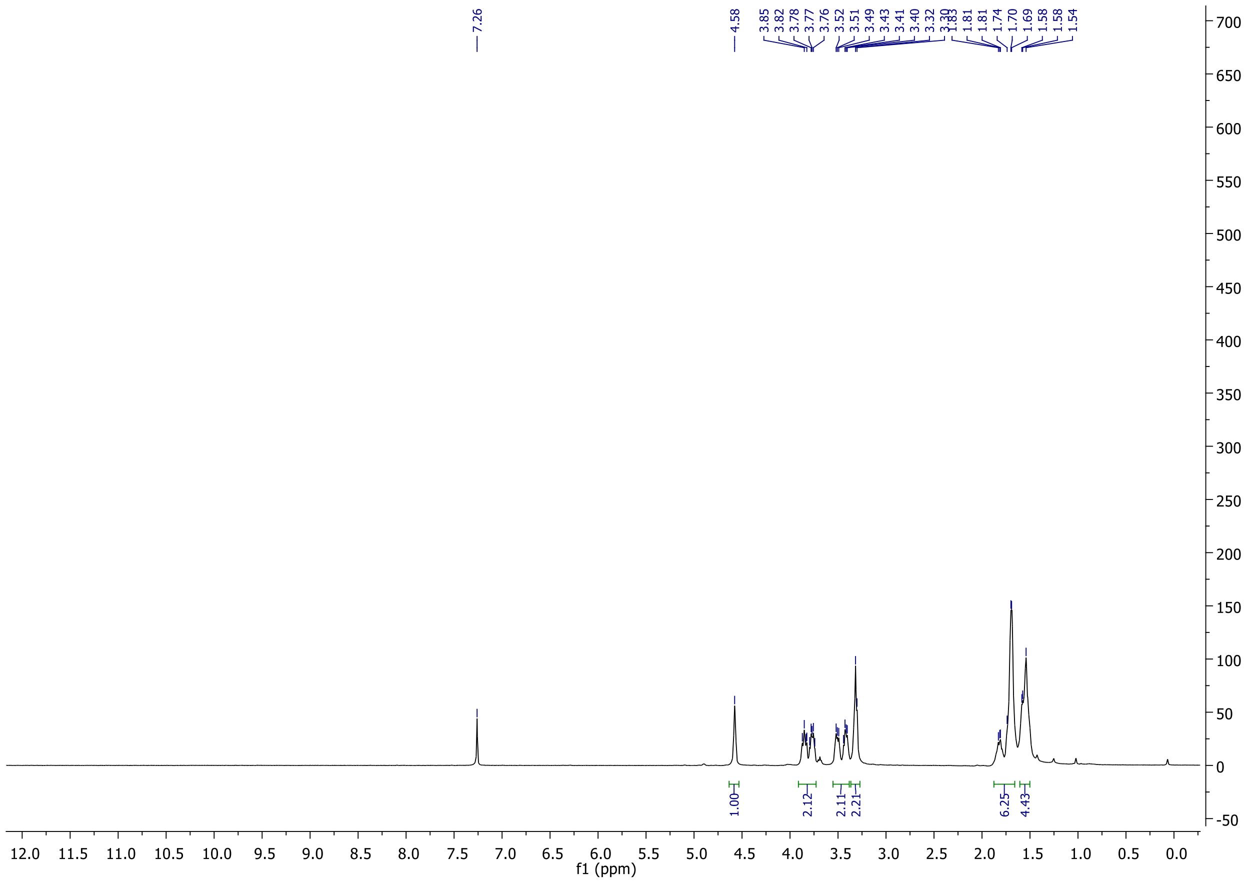

^{13}C NMR (101 MHz, $CDCl_3$) spectrum of compound **3**

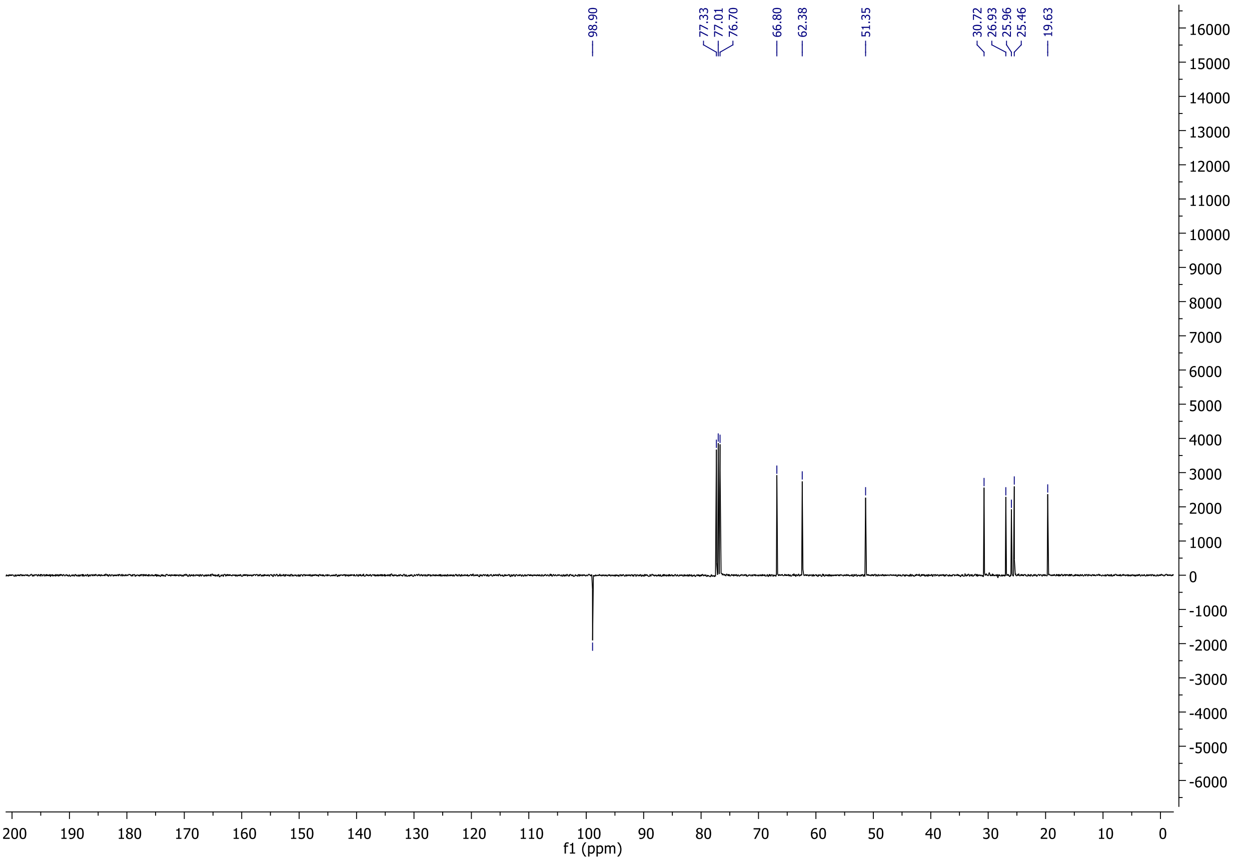

^{1}H NMR (400 MHz, $CDCl_3$) spectrum of compound **4**

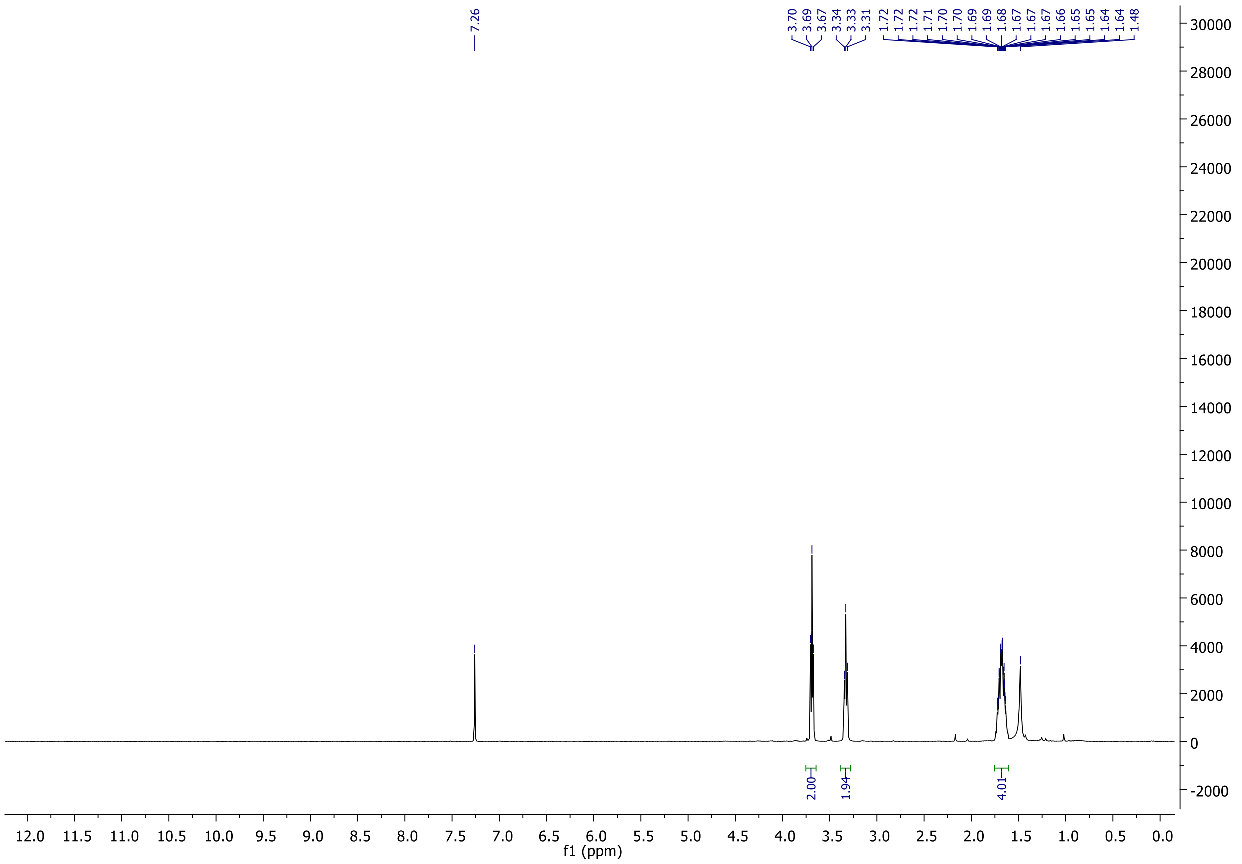

^{13}C NMR (101 MHz, $CDCl_3$) spectrum of compound **4**

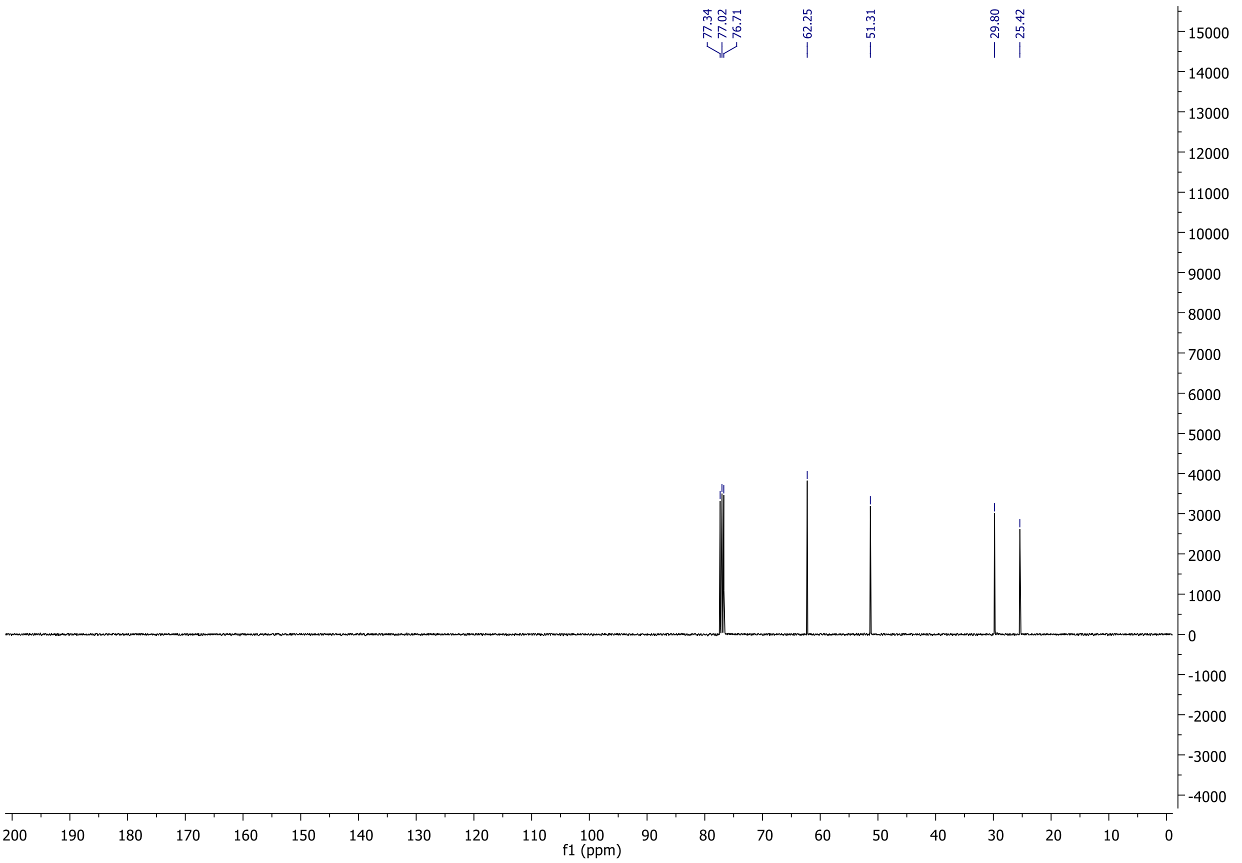

^{1}H NMR (400 MHz, $CDCl_3$) spectrum of compound **5**

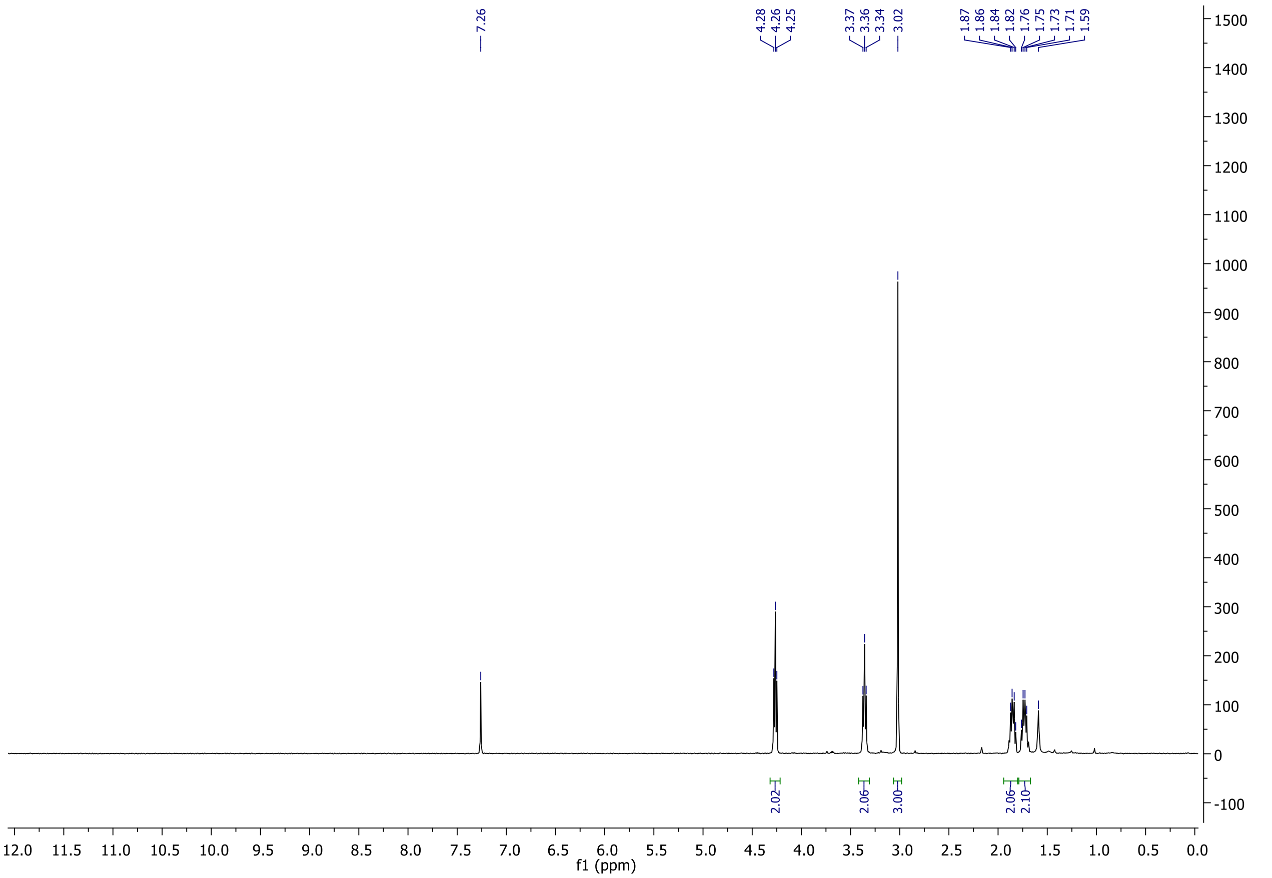

^{13}C NMR (101 MHz, $CDCl_3$) spectrum of compound **5**

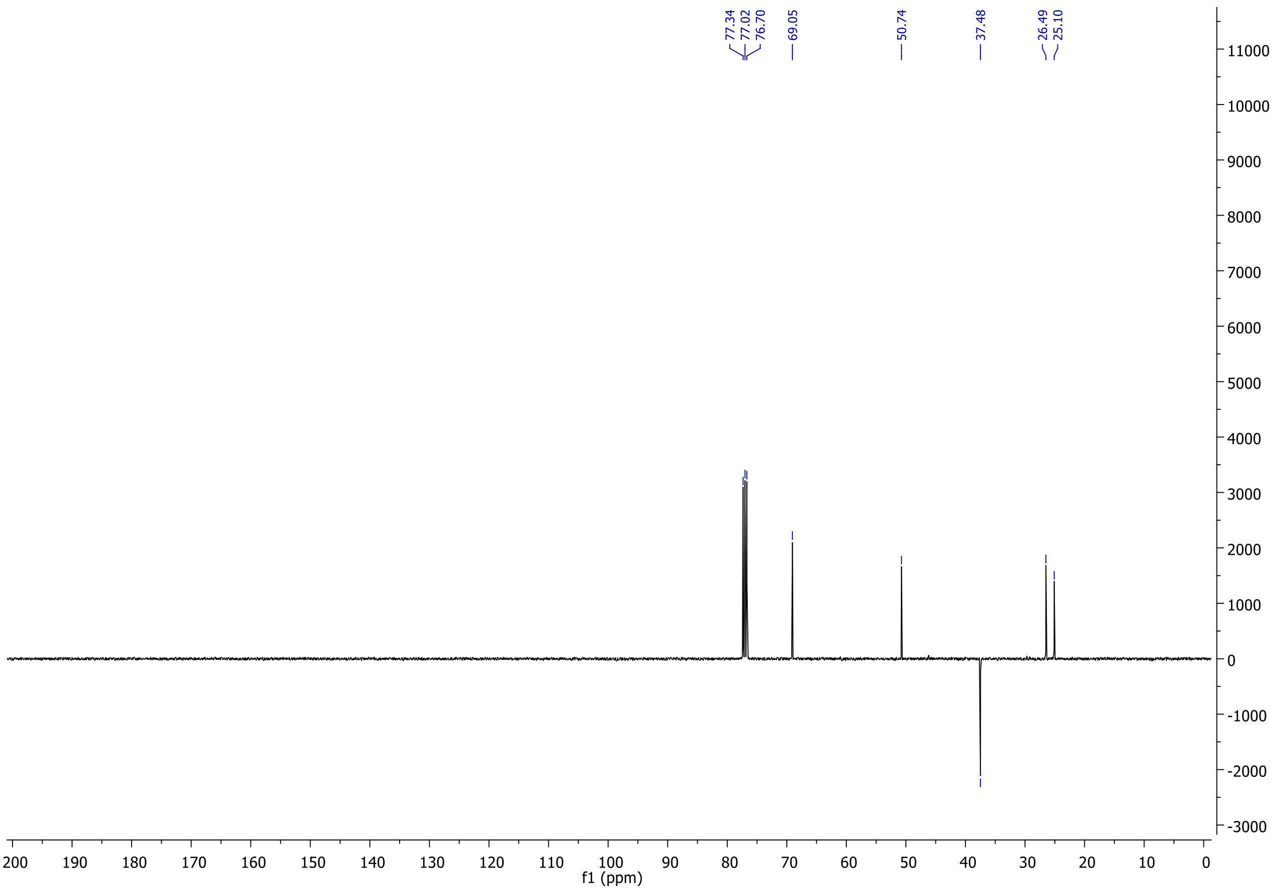

^1H NMR (400 MHz, $CDCl_3$) spectrum of compound **6**

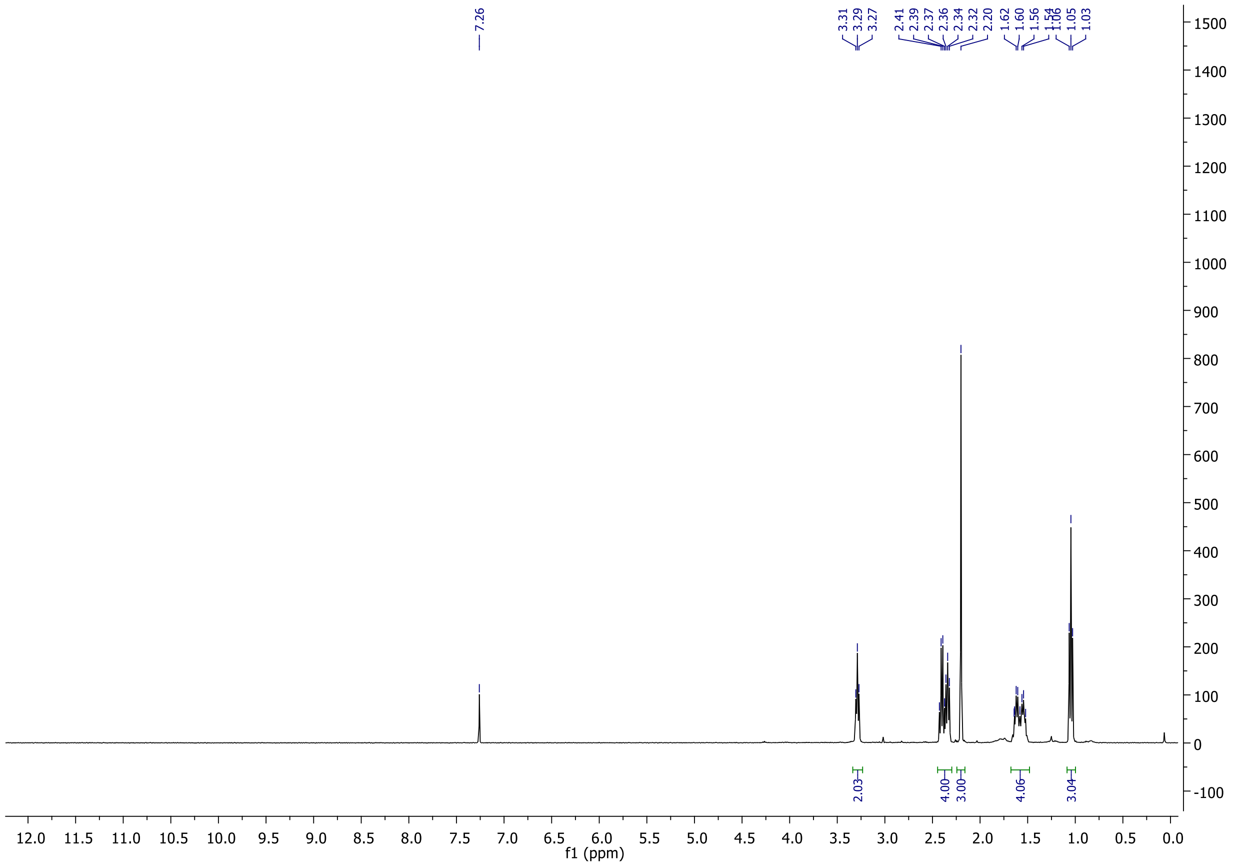

^{1}H NMR (400 MHz, $CDCl_3$) spectrum of compound **C171/I**

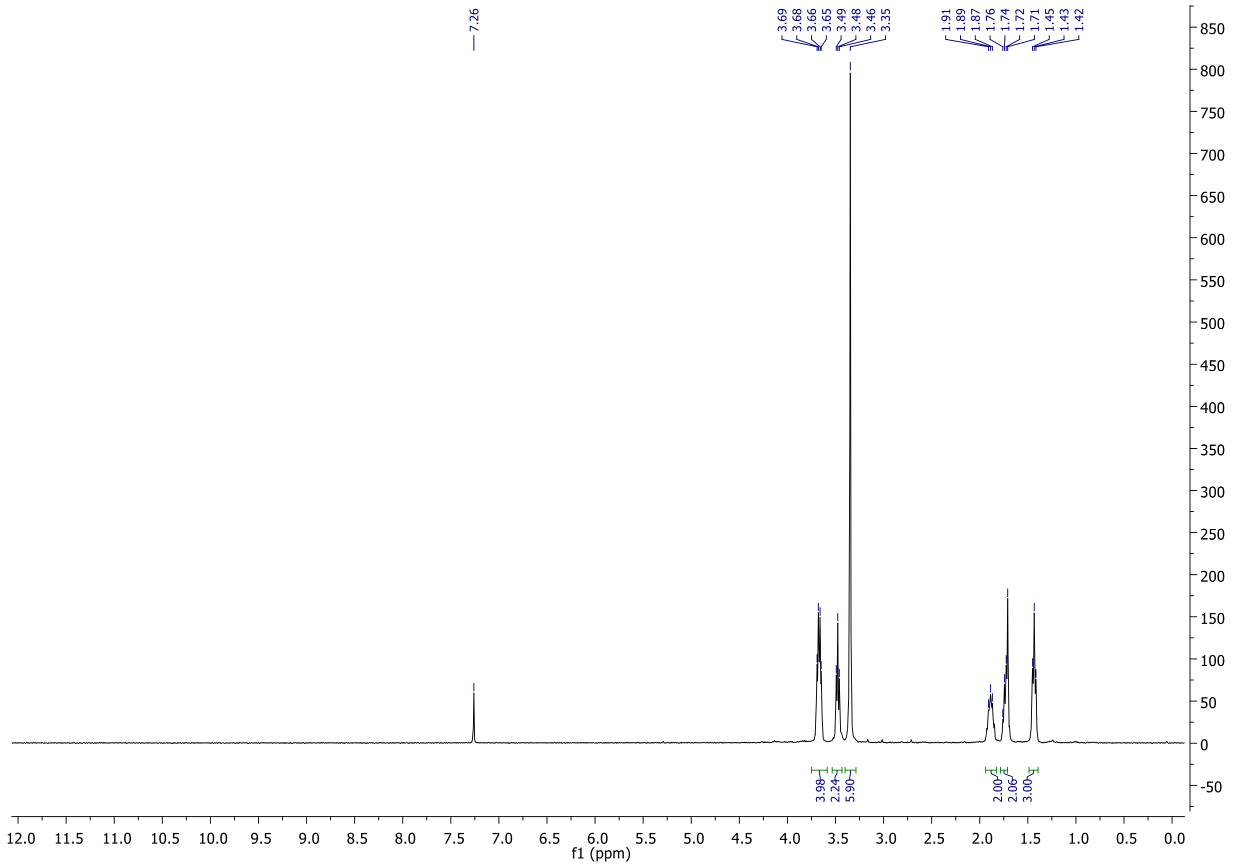

^{13}C NMR (101 MHz, $CDCl_3$) spectrum of compound **C171/I**

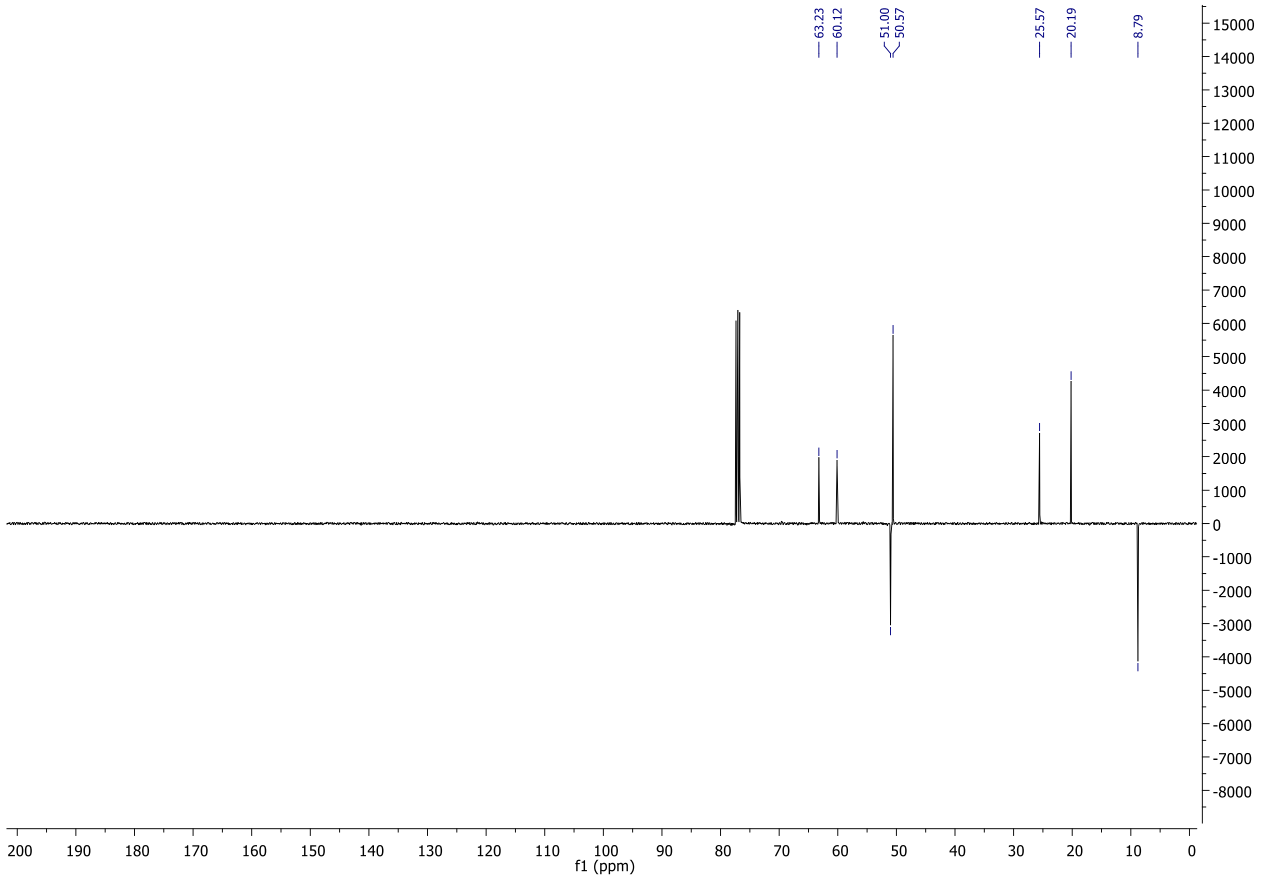

References

[1] F. Allegrini, A. C. Olivieri, in *Comprehensive Chemometrics*, Elsevier, **2020**, pp. 441–463.

[2] L. F. Eggers, D. Schwudke, in *Encyclopedia of Lipidomics* (Ed.: M.R. Wenk), Springer Netherlands, Dordrecht, **2016**, pp. 1–6.

[3] K. Berggren, T. H. Steinberg, W. M. Lauber, J. A. Carroll, M. F. Lopez, E. Chernokalskaya, L. Zieske, Z. Diwu, R. P. Haugland, W. F. Patton, *Analytical Biochemistry* **1999**, *276*, 129–143.

[4] C. Thiele, K. Wunderling, P. Leyendecker, *Nat Methods* **2019**, *16*, 1123–1130.

[5] M. M. Littleson, C. M. Baker, A. J. Dalençon, E. C. Frye, C. Jamieson, A. R. Kennedy, K. B. Ling, M. M. McLachlan, M. G. Montgomery, C. J. Russell, A. J. B. Watson, *Nat Commun* **2018**, *9*, 1105.

Author Contributions

M.F. and L.G. conceived the project. J.D and M.E. synthesized the C171 MS reporter. C.T. provided the Click Internal Standard Mix. P.N. conducted cell culture experiments, derivatization and LC-MS analysis. P.N. and G.L.B. performed manual MS2 spectra annotation. S.B., G.L.B., and L.G. constructed the Click Lipid Database. P.N., S.B., G.L.B., and L.G. analyzed LC-MS datasets. P.N. prepared figures and wrote the initial manuscript draft. M.F. and L.G. contributed to the final manuscript version and supervised the project. All authors read and agreed on the content of the paper.
