# Supplementary material for: LC‐MS and High‐Throughput Data Processing Solutions for Lipid Metabolic Tracing Using Bioorthogonal Click Chemistry
Source: Angew Chem Int Ed Engl. 2025 May 2;64(27):e202501884. doi: 10.1002/anie.202501884 (PMC12207371; doi:10.1002/anie.202501884)
Supplement: Supplementary file 2 — Supporting Information [file ANIE-64-e202501884-s001.zip › anie202501884-supp-0002-SuppMat/File S1.pdf]

File S1. LC-MS description of C171-derivatized alkyne-containing lipids from Click Internal Standard Mix.

For each species, chromatographic profile, MS/MS spectra of $[cM]^{+}$ and/or $[cM+H]^{2+}$ (one C171 unit per structure), $[ccM]^{2+}$ and/or $[ccM+H]^{3+}$ (two C171 units per structure), $[cccM]^{3+}$ (three C171 units per structure), and putative gas-phase fragmentation schemes are shown.

Notes:

The sn position cannot be determined from MS^2 data, in most cases structures are drawn with a C171 chain in sn-1 only for convenience in drawing schemes.

All fragment ion structures are only conjectures.

The color-coding of fragment abundances in the proposed fragmentation schemes corresponds to the averaged signal intensities obtained by integrating "precursor → fragment" transitions in n = 10-20 raw files (see Supplementary Data Table 1 for complete lists of fragment ions and their relative abundances).

m/z 226.300 is the noise signal present in the week of acquisition of certain MS/MS spectra.

Content (hyperlink):

1. LPC-[13]C3 19:1;C171
2. PC 17:0;C171_15:1-[2]H8
3. PC-[13]C3 19:1;C171_19:1;C171
4. PE 17:0;C171_15:1-[2]H8
5. PE-[13]C3 19:1;C171_19:1;C171
6. PA 17:0;C171_15:1-[2]H8
7. PA-[13]C3 19:1;C171_19:1;C171
8. PI 17:0;C171_15:1-[2]H8
9. PS 17:0;C171_15:1-[2]H8
10. Cer 18:0;O2;C171/15:1-[2]H8
11. HexCer 18:1;O2/15:0;C171-[13]C2
12. SM 18:1;O2/15:0;C171-[13]C2
13. MG-[13]C3 19:1;C171
14. DG 17:0;C171_15:1-[2]H8
15. DG-[13]C3 19:1;C171_19:1;C171
16. TG 17:0;C171_15:1-[2]H8_16:0
17. TG 17:0;C171_17:0;C171_15:1-[2]H8
18. TG-[13]C3 19:1;C171_19:1;C171_19:1;C171
19. CE-[2]H7 17:0;C171

LPC-$^{13}C_3$ 19:1;C171 353.2551 – $C_{32}{}^{13}C_3H_{70}N_5O_7P^{2+}$

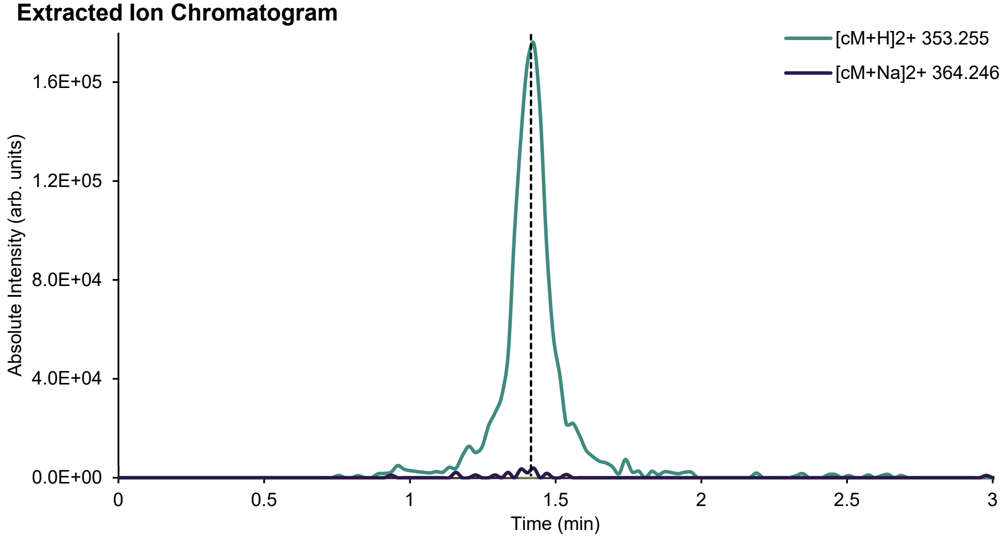

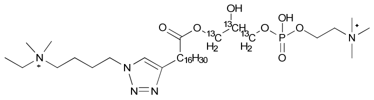

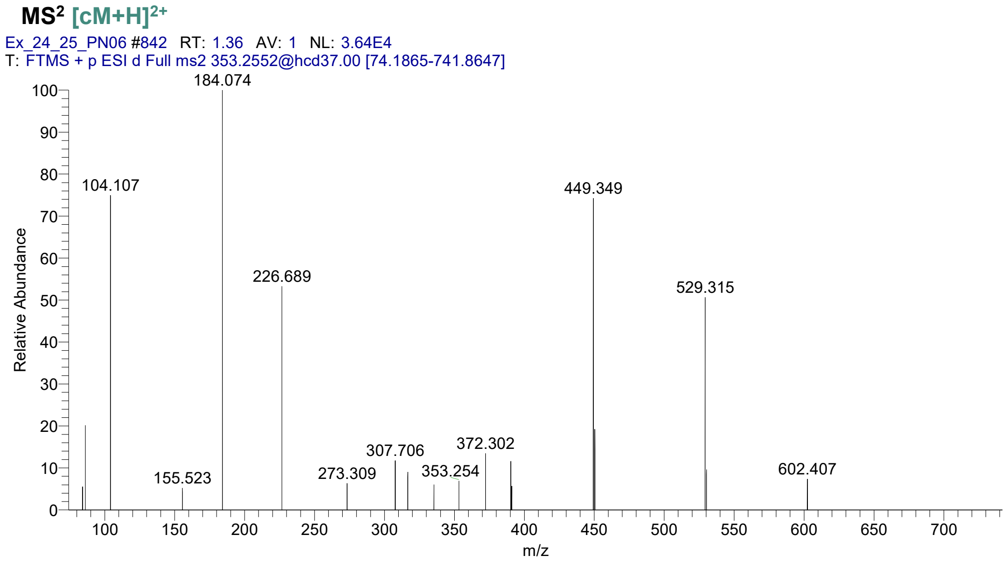

◂ Back to Content

LPC-$^{13}C_3$ 19:1;C171 353.2551 – $C_{32}{}^{13}C_3H_{70}N_5O_7P^{2+}$

proposed fragmentation **scheme**

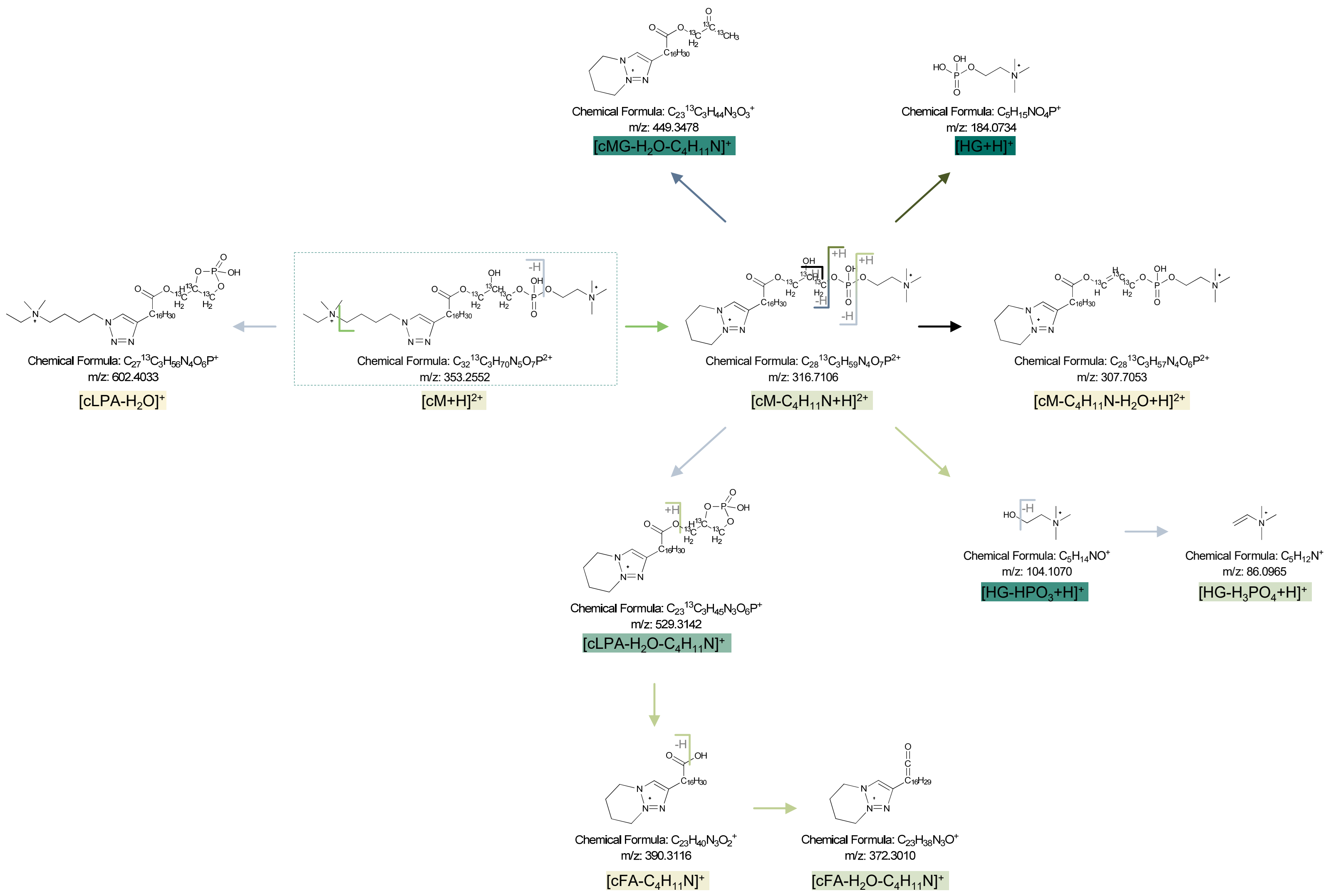

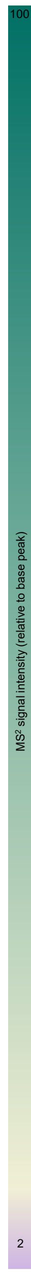

◂ Back to Content

PC 17:0;C171_15:1-d$_8$ 906.7259 – $C_{48}H_{85}D_8N_5O_8P^{+}$ / 453.8666 – $C_{48}H_{86}D_8N_5O_8P^{2+}$

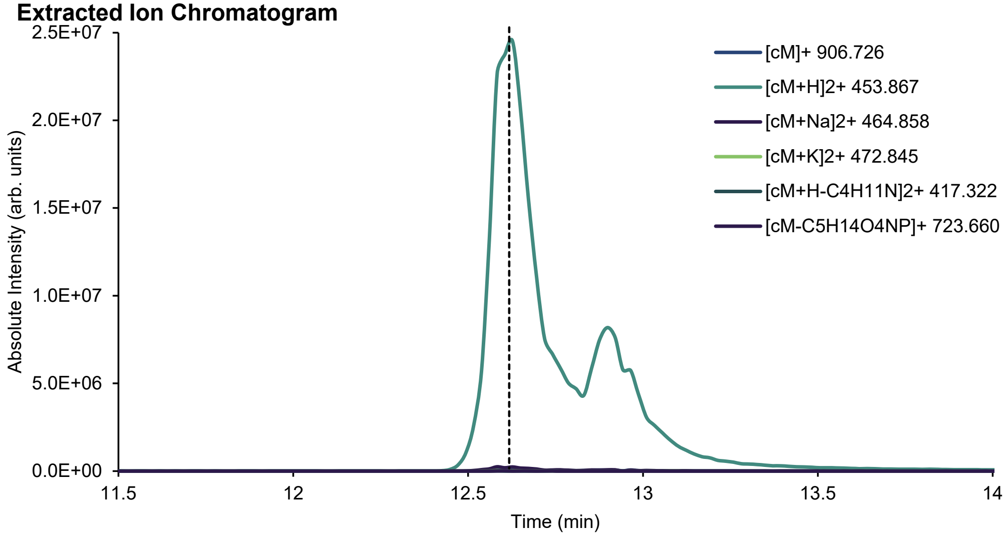

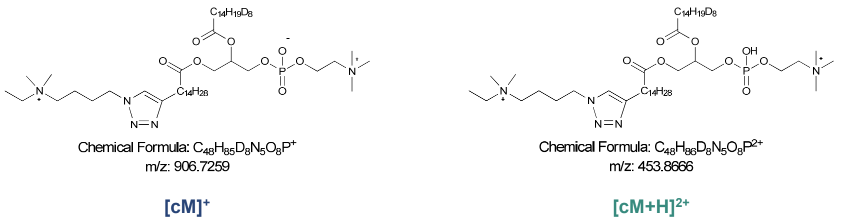

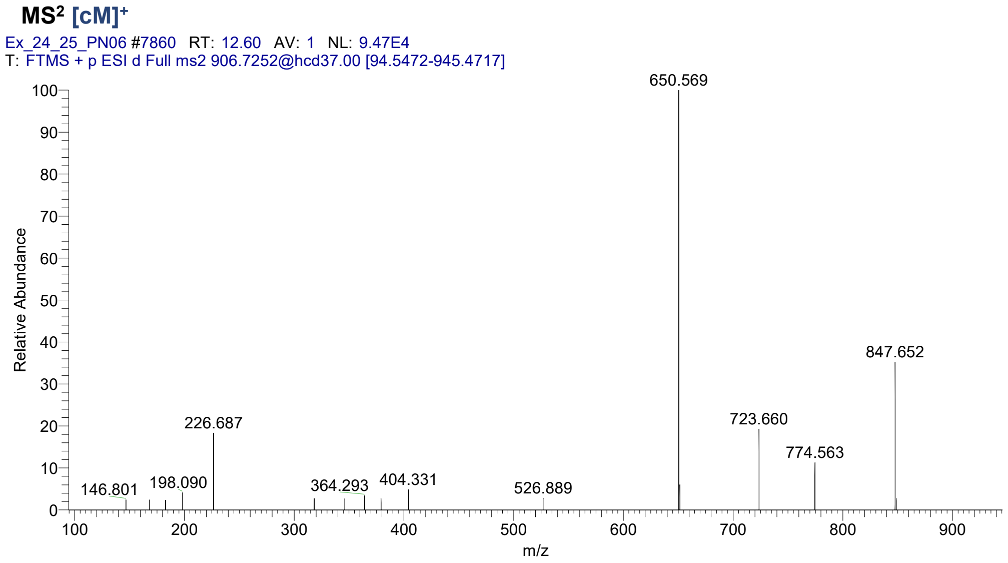

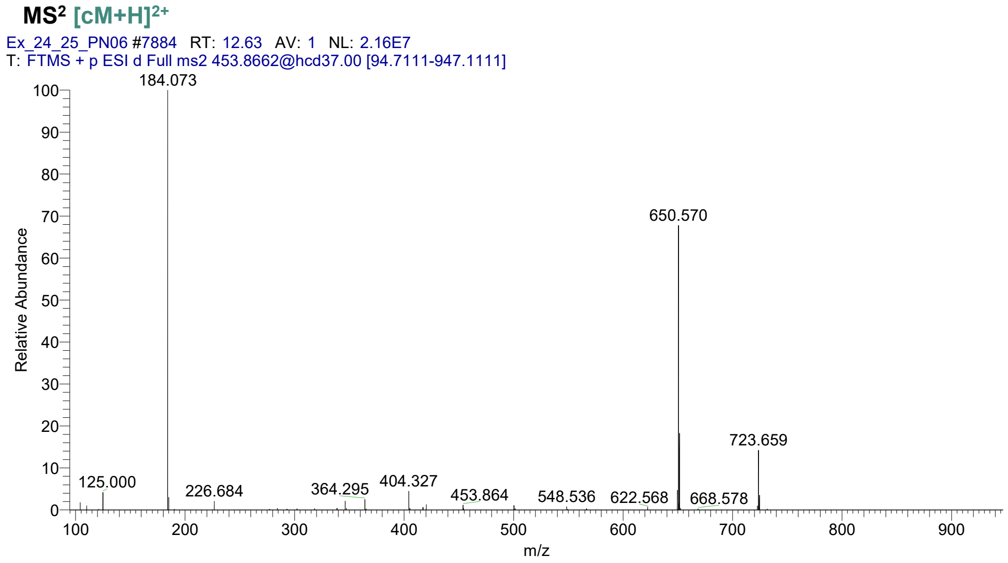

◂ Back to Content

PC 17:0;C171_15:1-d_8 906.7259 – $C_{48}H_{85}D_8N_5O_8P^+$ proposed fragmentation scheme

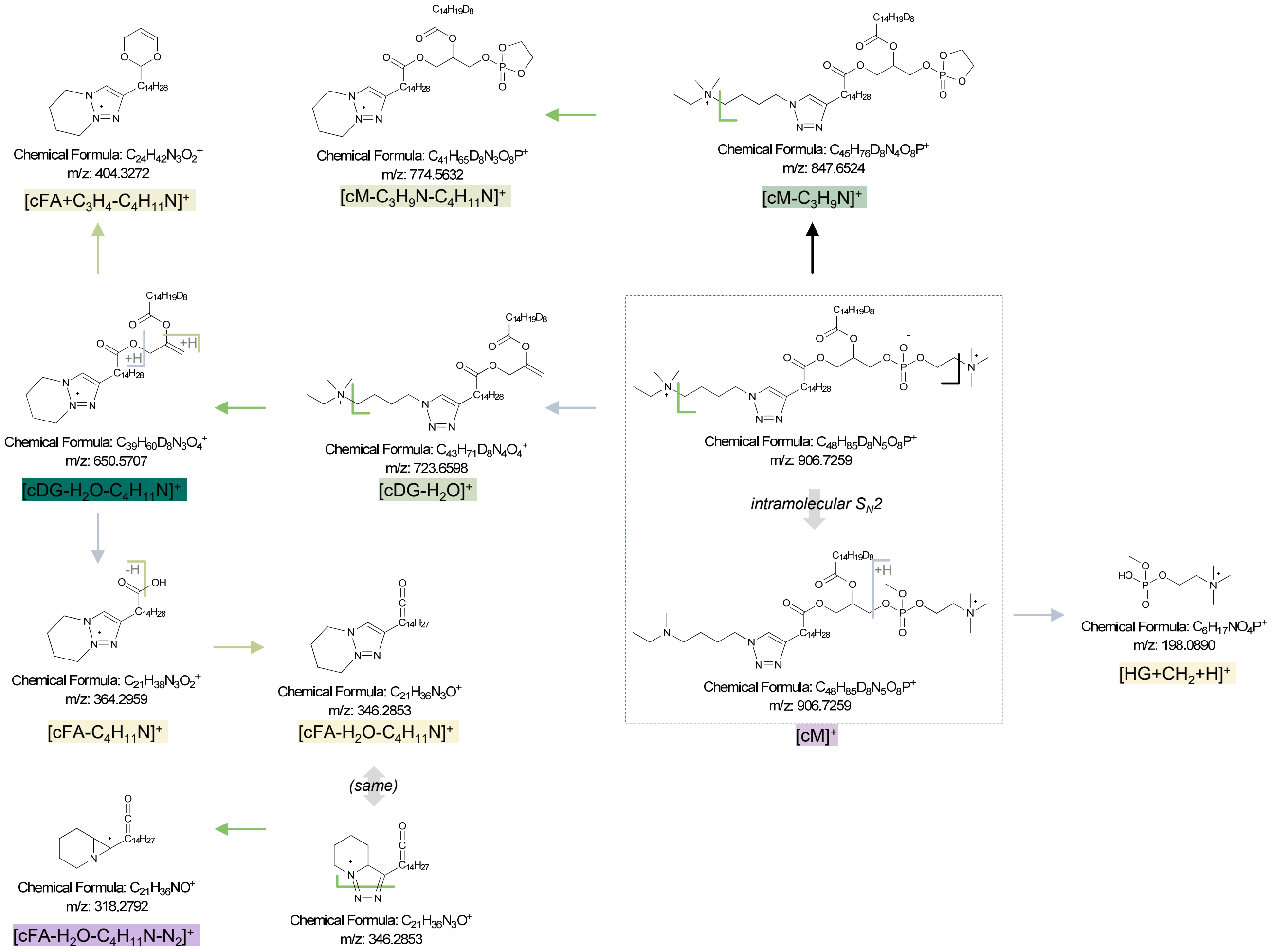

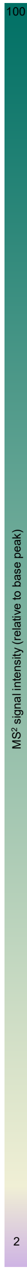

◂ Back to Content

PC 17:0;C171_15:1-d_8 453.8666 – $C_{48}H_{86}D_8N_5O_8P^{2+}$ proposed fragmentation scheme

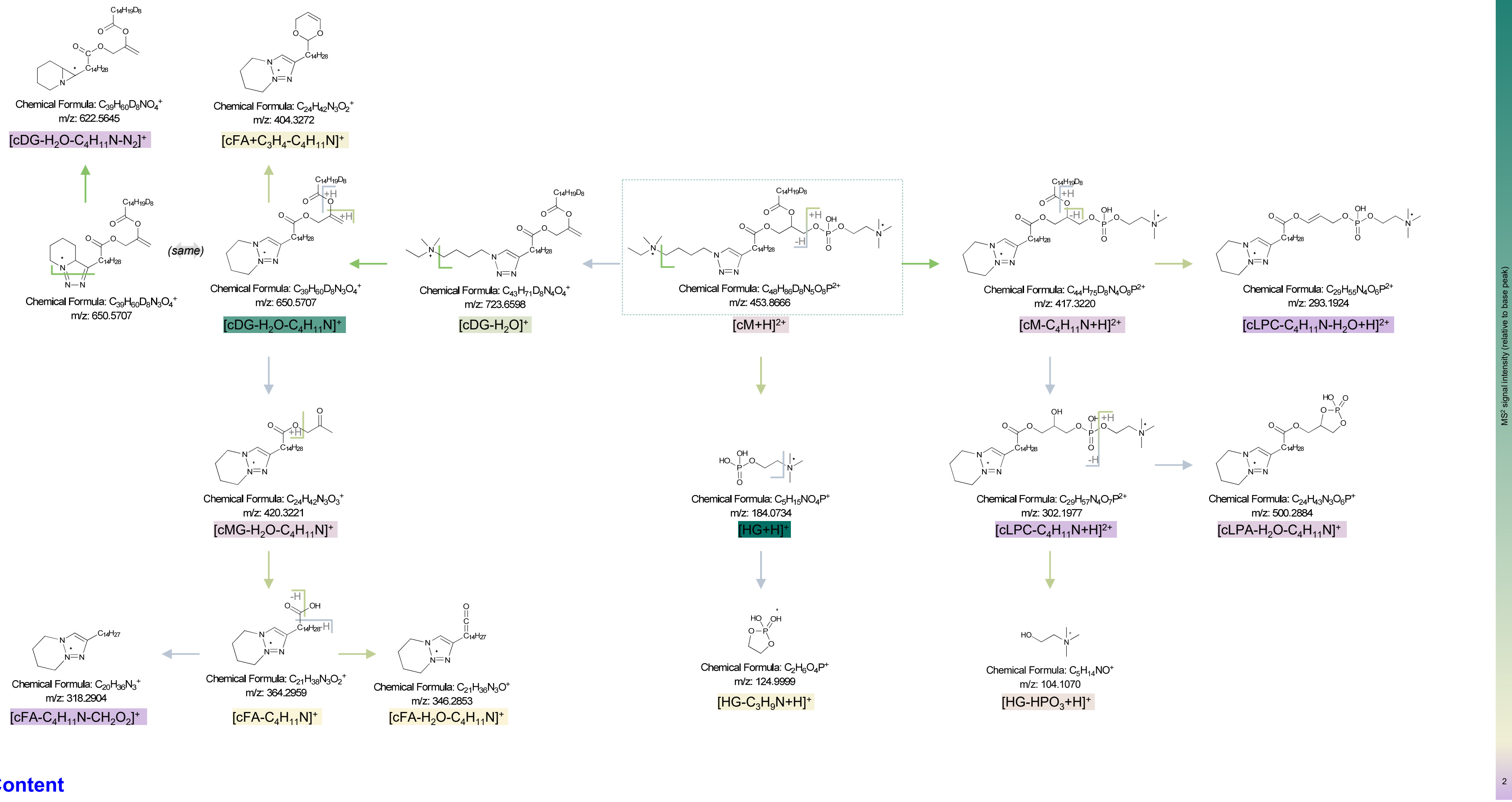

◂ Back to Content

PC-$^{13}C_3$ 19:1;C171_19:1;C171 575.4466 – $C_{59}{}^{13}C_3H_{118}N_9O_8P^{2+}$ / 383.9668 – $C_{59}{}^{13}C_3H_{119}N_9O_8P^{3+}$

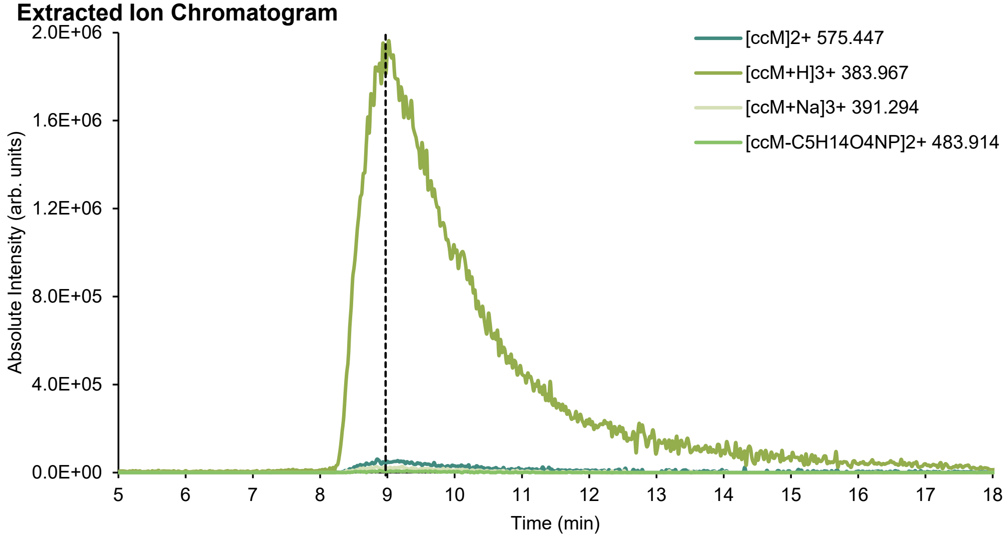

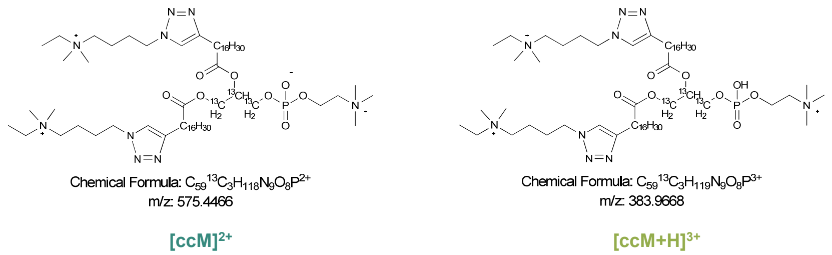

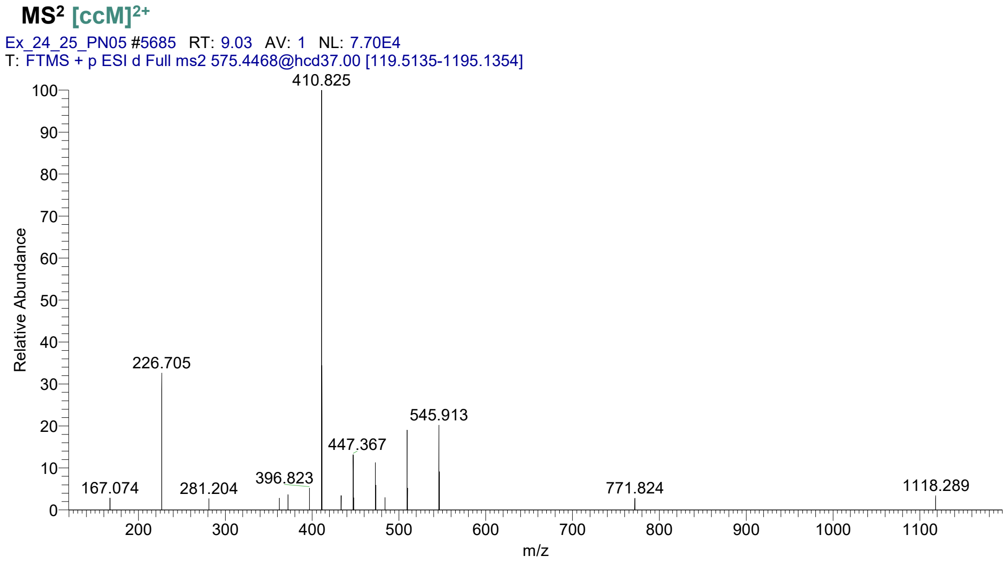

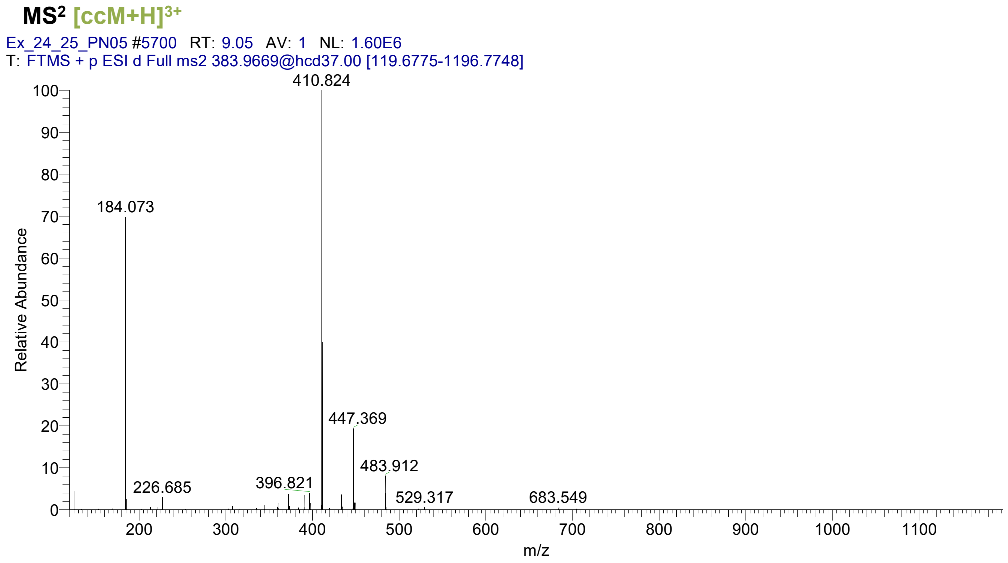

◂ Back to Content

PC-$^{13}C_3$ 19:1;C171_19:1;C171 575.4466 – $C_{59}{}^{13}C_3H_{118}N_9O_8P^{2+}$ proposed fragmentation scheme

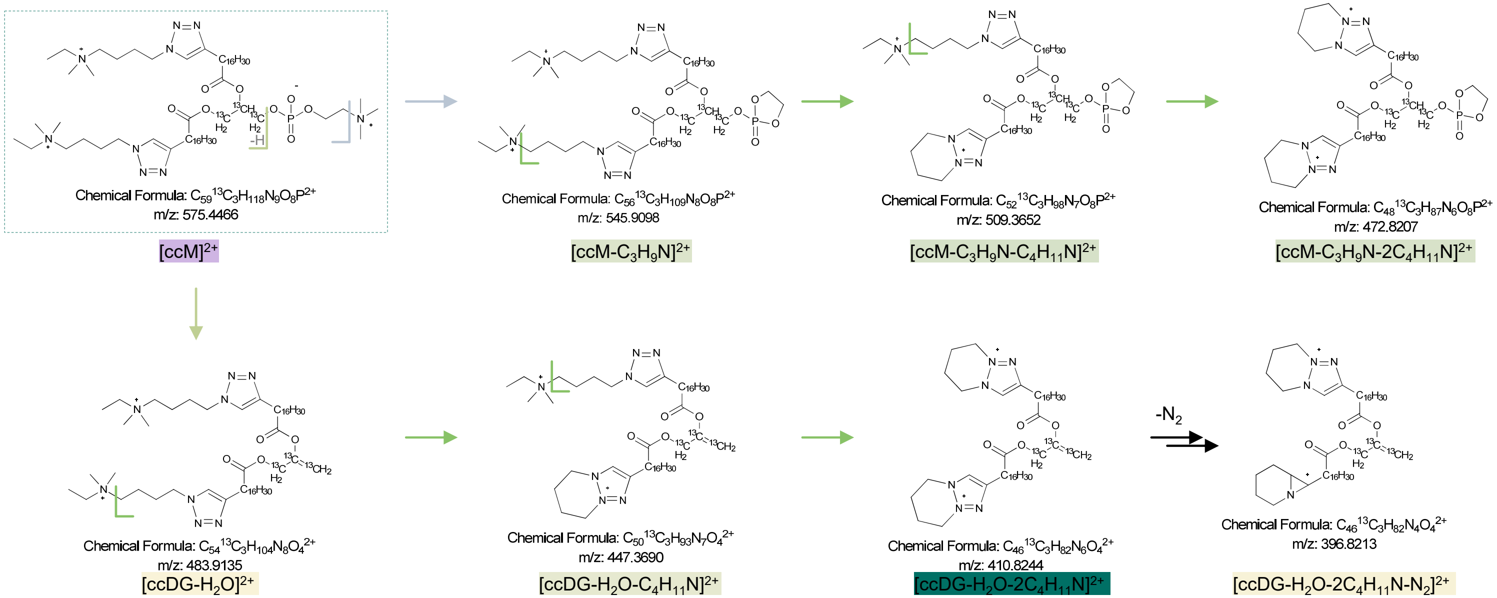

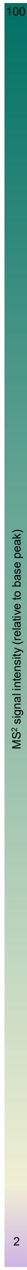

◂ Back to Content

PC-$^{13}C_3$ 19:1;C171_19:1;C171 383.9668 – $C_{59}{}^{13}C_3H_{119}N_9O_8P^{3+}$ proposed fragmentation scheme

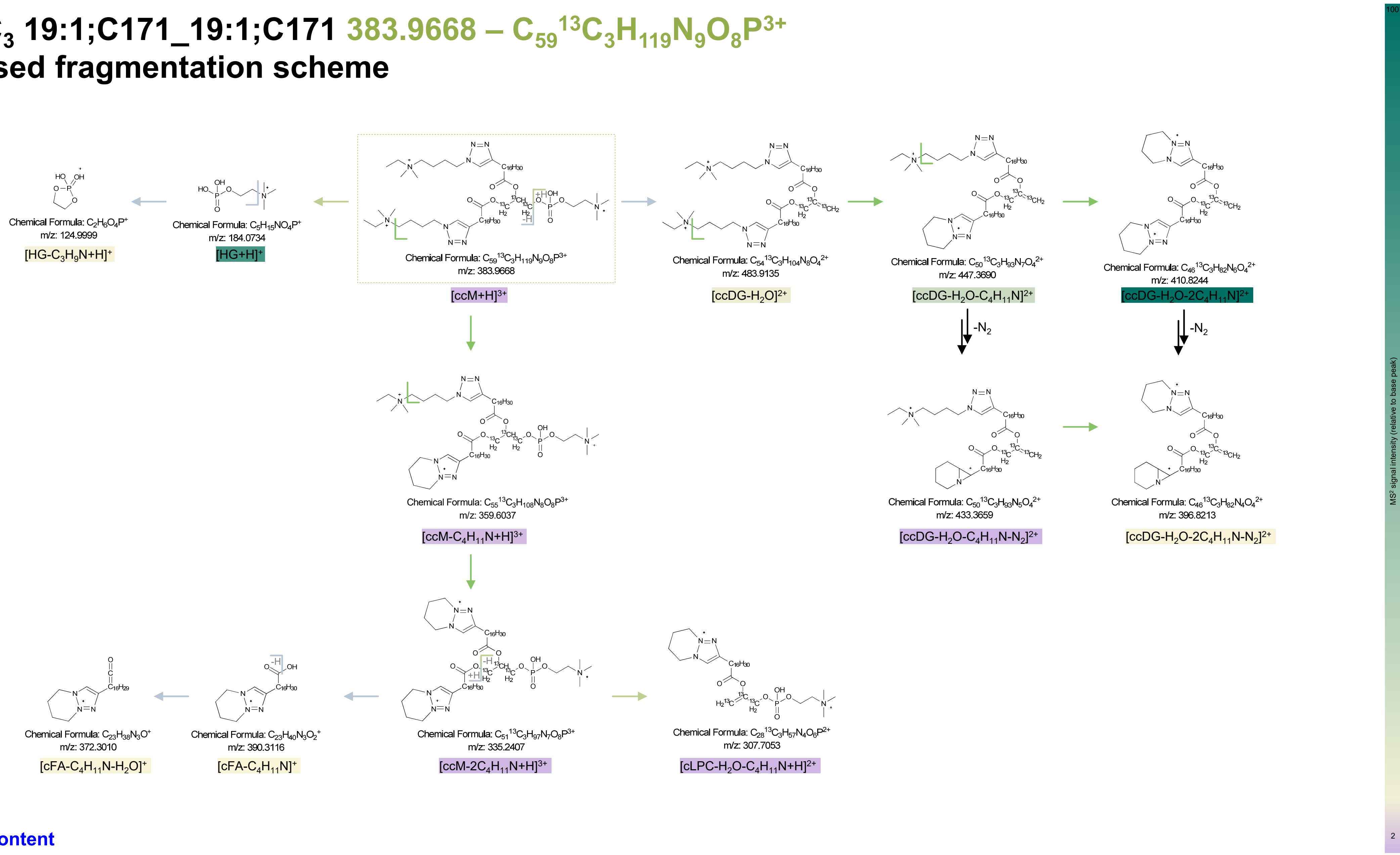

◂ Back to Content

PE 17:0;C171_15:1-d8 864.6789 – $C_{45}H_{79}D_8N_5O_8P^+$ / 432.8431 - $C_{45}H_{80}D_8N_5O_8P^{2+}$

Extracted Ion Chromatogram

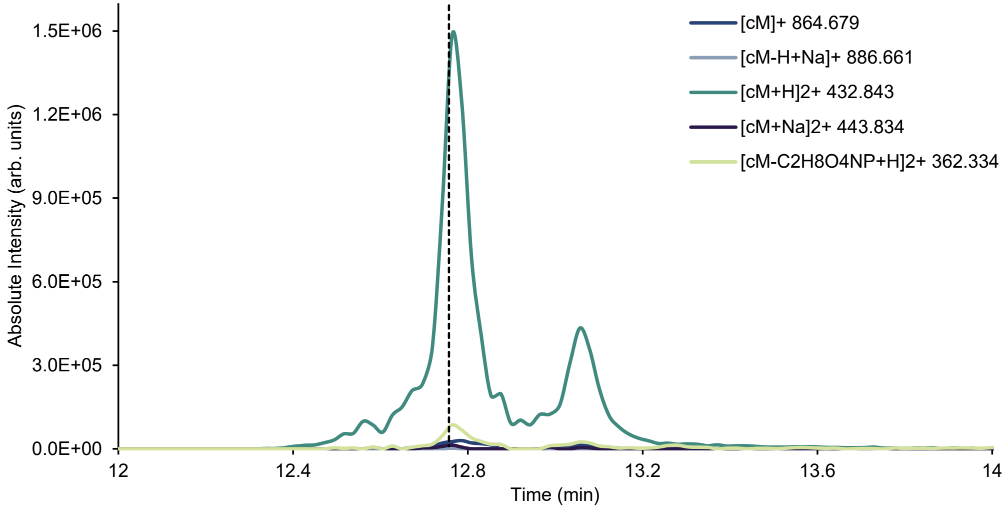

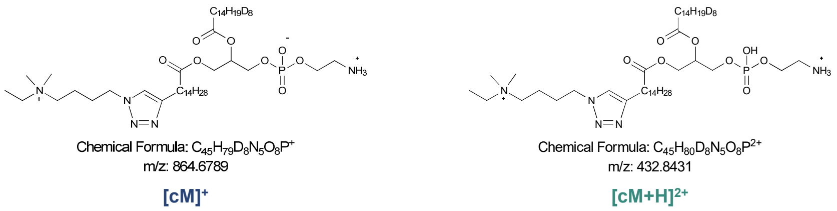

MS² [cM]⁺

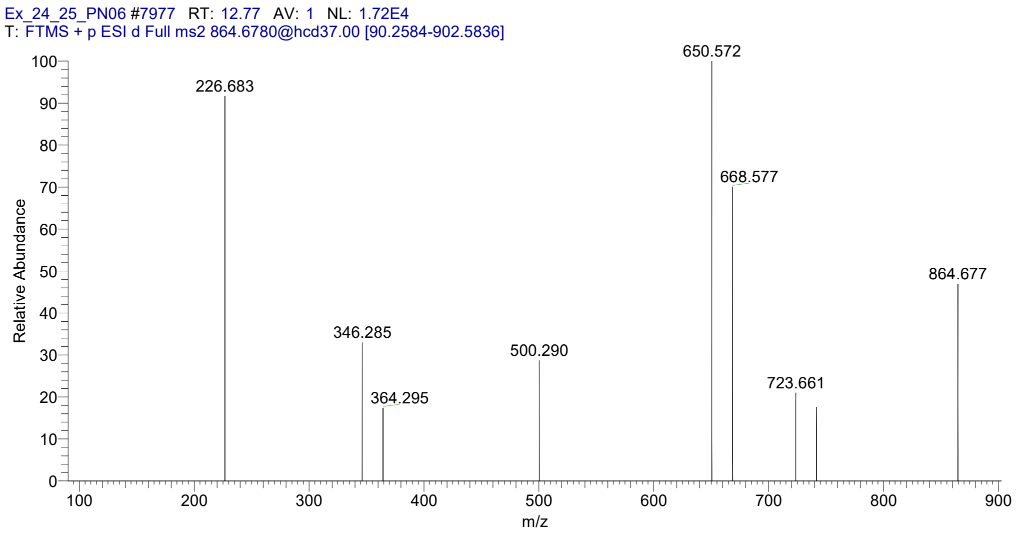

MS² [cM+H]²⁺

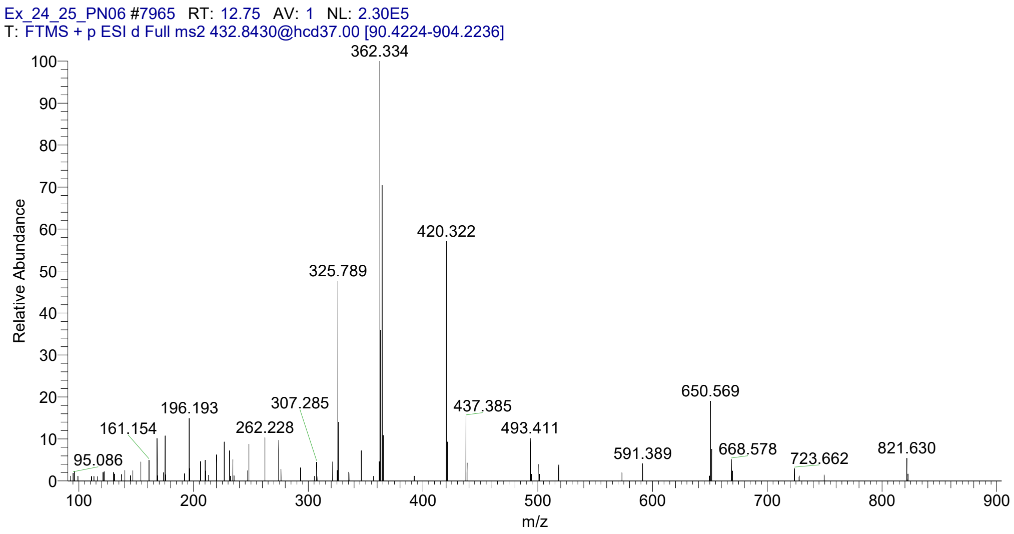

◂ Back to Content

PE 17:0;C171_15:1-d_8 864.6789 – $C_{45}H_{79}D_8N_5O_8P^+$
proposed fragmentation scheme

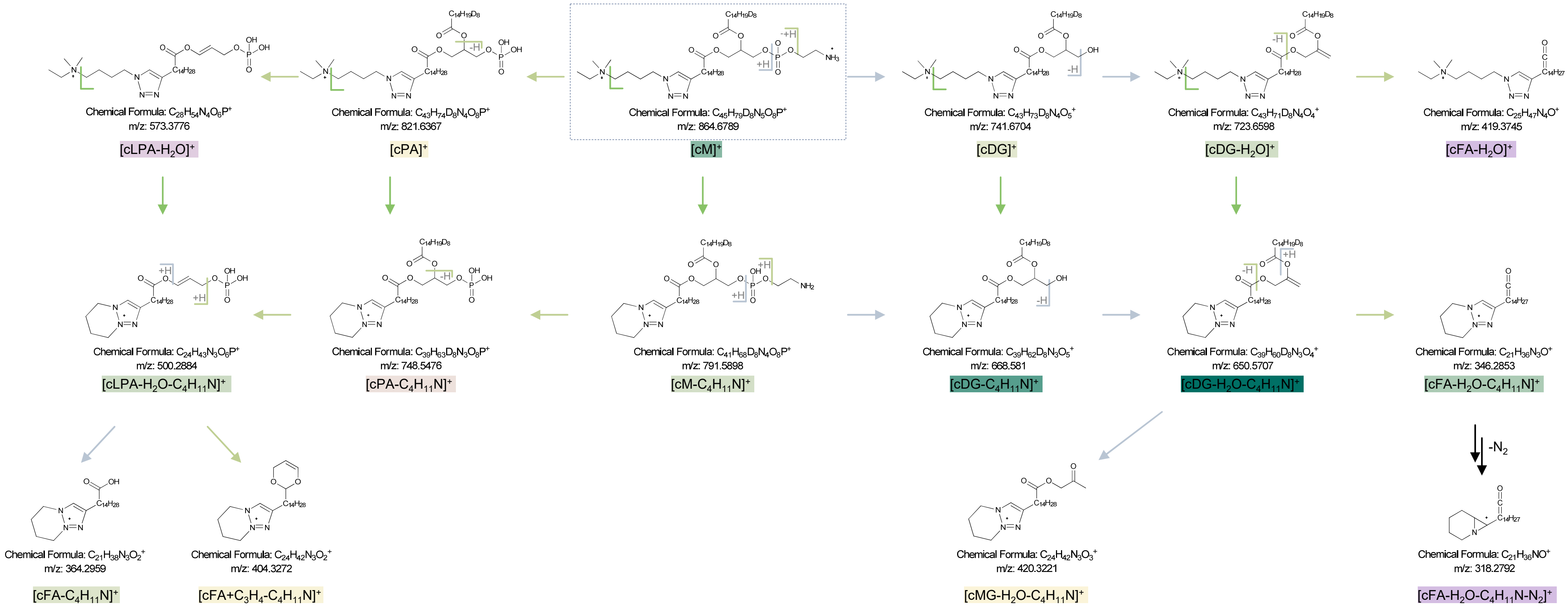

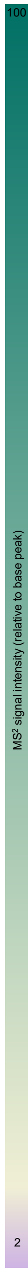

◂ Back to Content

PE 17:0;C171_15:1-d$_8$ 432.8431 - $C_{45}H_{80}D_8N_5O_8P^{2+}$ proposed fragmentation scheme

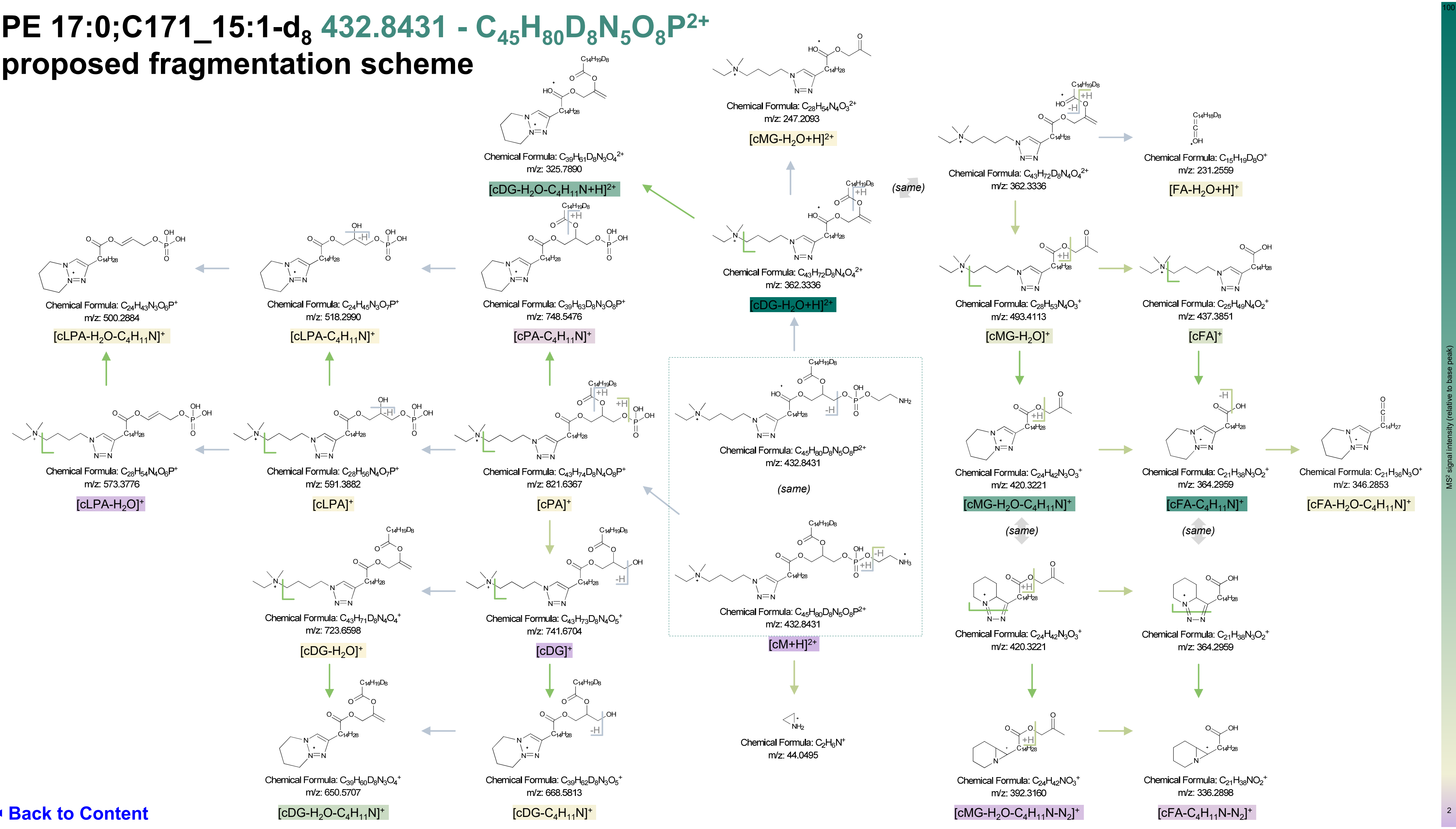

◂ Back to Content

PE-$^{13}C_3$ 19:1;C171_19:1;C171 554.4231 – $C_{56}{}^{13}C_3H_{112}N_9O_8P^{2+}$ / 369.9511 – $C_{56}{}^{13}C_3H_{113}N_9O_8P^{3+}$

Extracted Ion Chromatogram

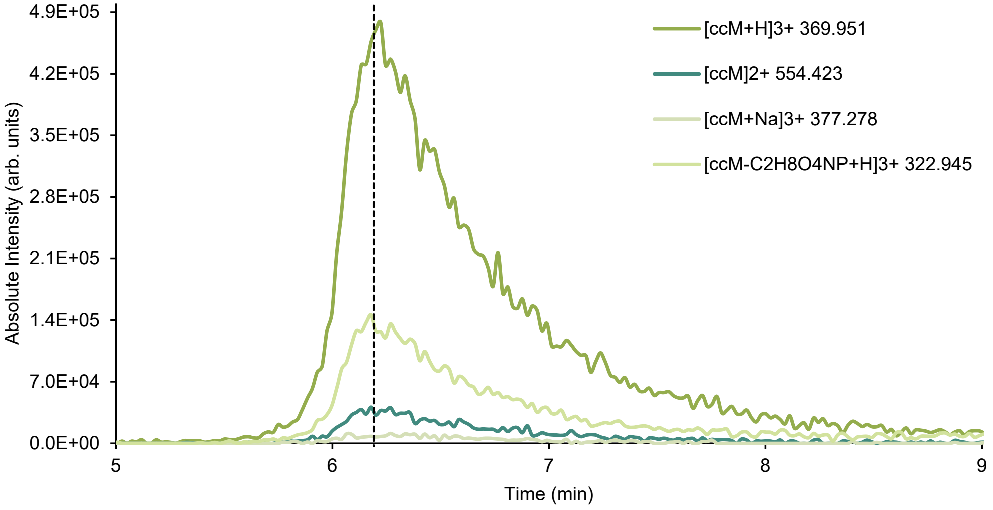

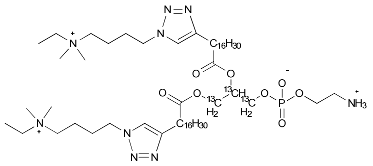

Chemical Formula: $C_{56}{}^{13}C_3H_{112}N_9O_8P^{2+}$
m/z: 554.4231

$[ccM]^{2+}$

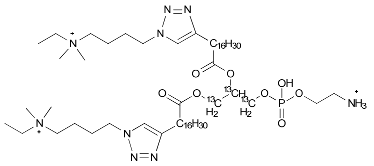

Chemical Formula: $C_{56}{}^{13}C_3H_{113}N_9O_8P^{3+}$
m/z: 369.9511

$[ccM+H]^{3+}$

MS^2 $[ccM]^{2+}$

Ex_24_27_PN05 #4180 RT: 6.70 AV: 1 NL: 2.88E4
T: FTMS + p ESI d Full ms2 554.4229@hcd37.00 [115.2247-1152.2466]

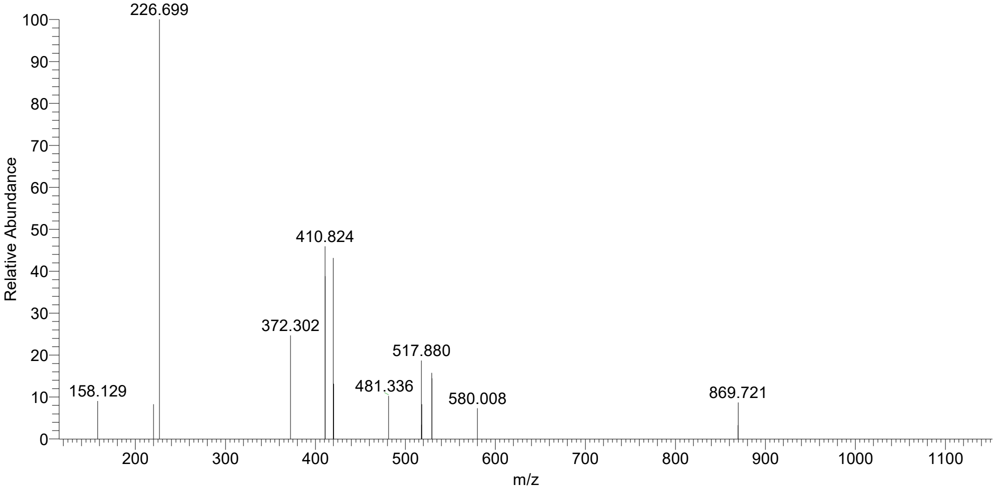

MS^2 $[ccM+H]^{3+}$

Ex_24_27_PN05 #4189 RT: 6.72 AV: 1 NL: 5.18E4
T: FTMS + p ESI d Full ms2 369.9511@hcd37.00 [115.3886-1153.8865]

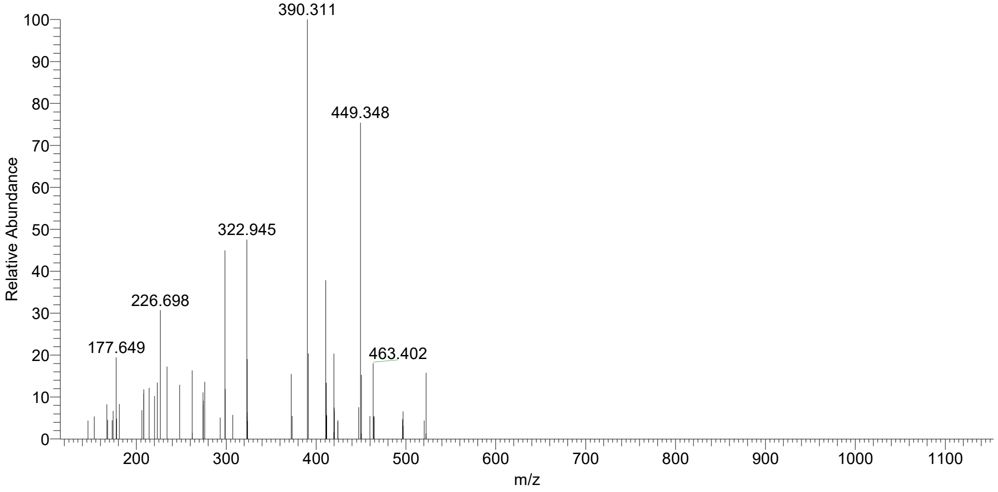

◂ Back to Content

PE-$^{13}C_3$ 19:1;C171_19:1;C171 554.4231 – $C_{56}{}^{13}C_3H_{112}N_9O_8P^{2+}$ proposed fragmentation scheme

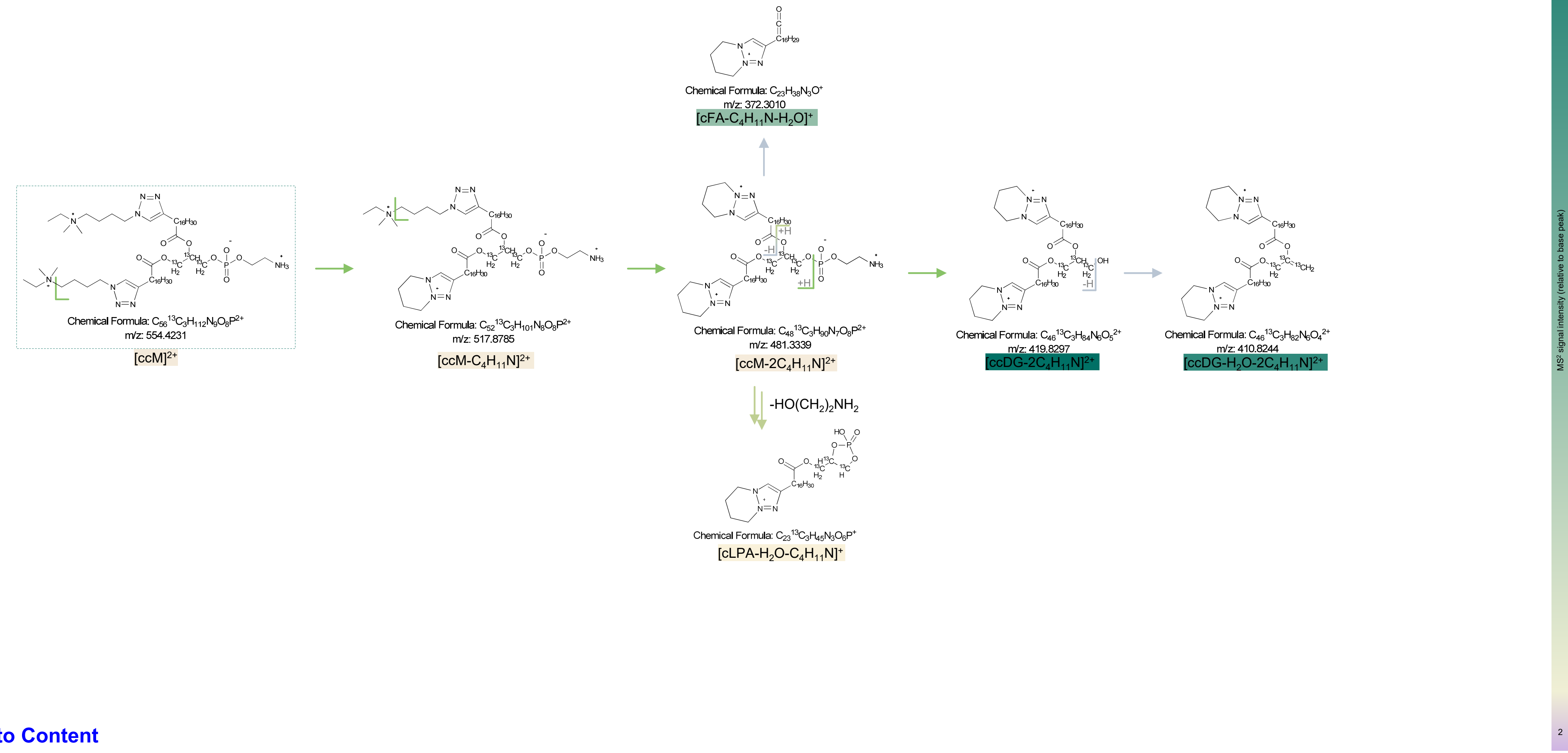

◂ Back to Content

PE-$^{13}C_3$ 19:1;C171_19:1;C171 369.9511 – $C_{56}{}^{13}C_3H_{113}N_9O_8P^{3+}$ proposed fragmentation scheme

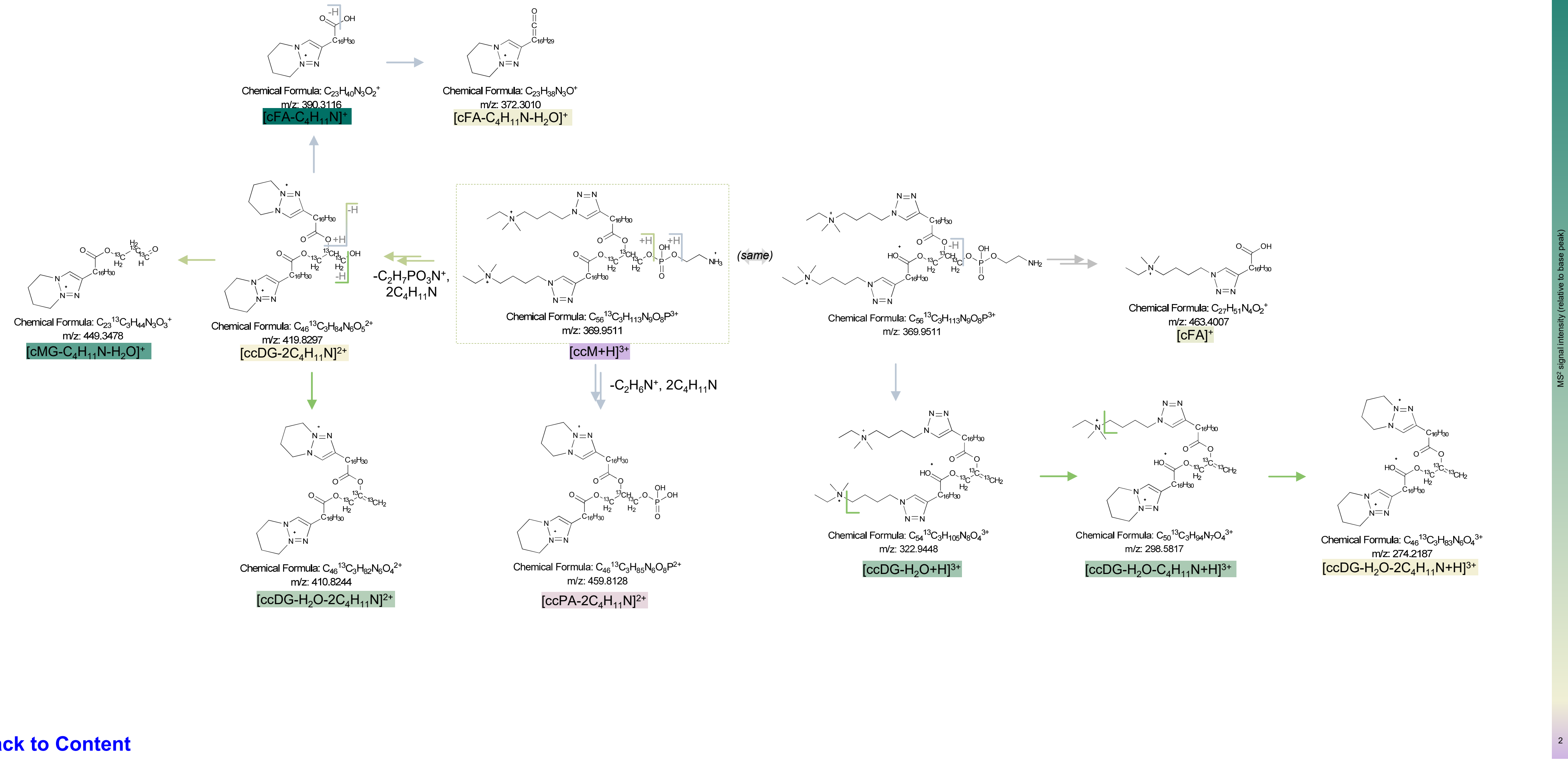

◂ Back to Content

PA 17:0;C171_15:1-d$_8$ 821.6367 – $C_{43}H_{74}D_8N_4O_8P^+$

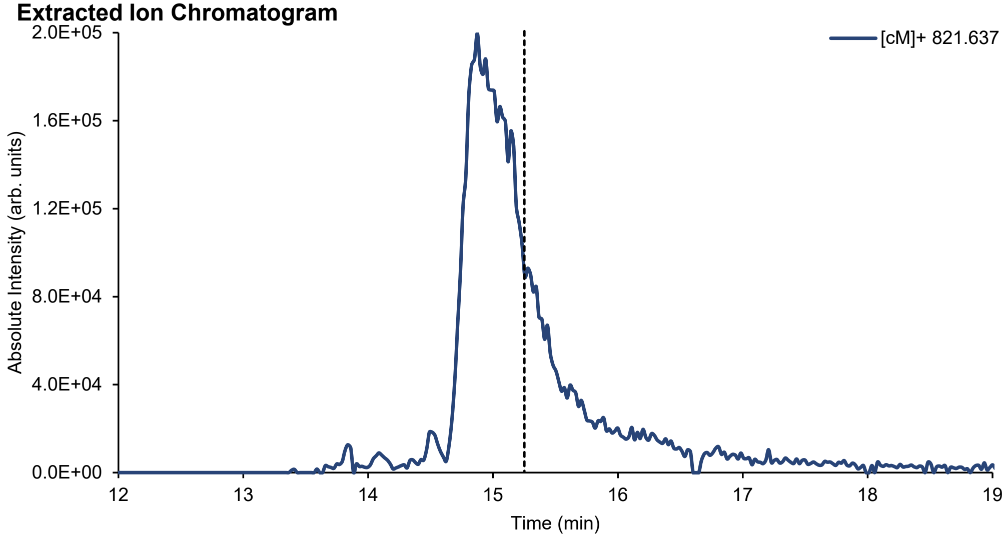

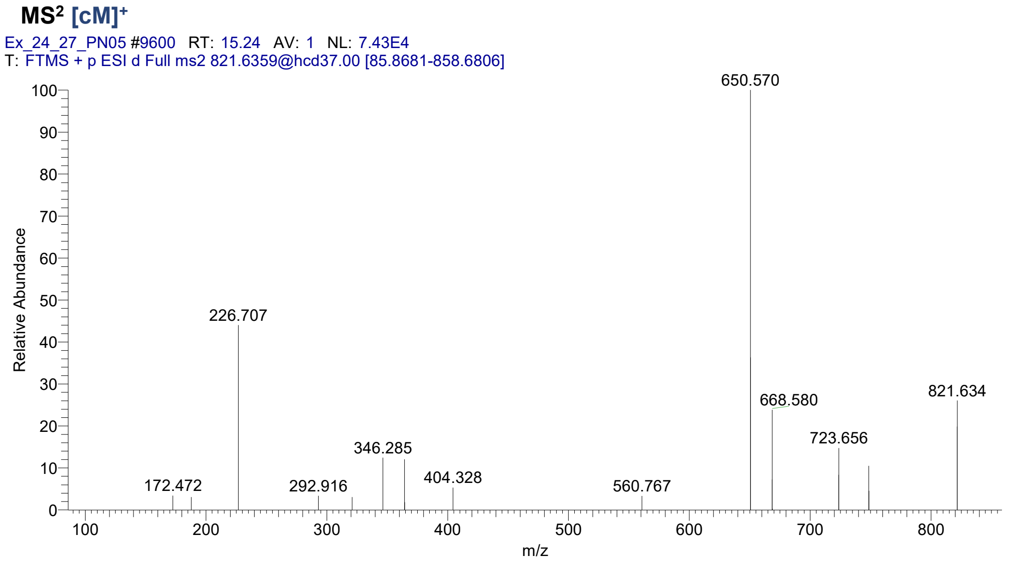

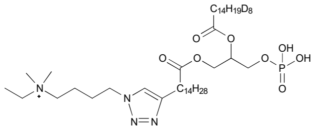

Chemical Formula: $C_{43}H_{74}D_8N_4O_8P^+$
m/z: 821.6367

[cM]$^+$

◂ Back to Content

PA 17:0;C171_15:1-d_8 821.6367 – $C_{43}H_{74}D_8N_4O_8P^+$
proposed fragmentation scheme

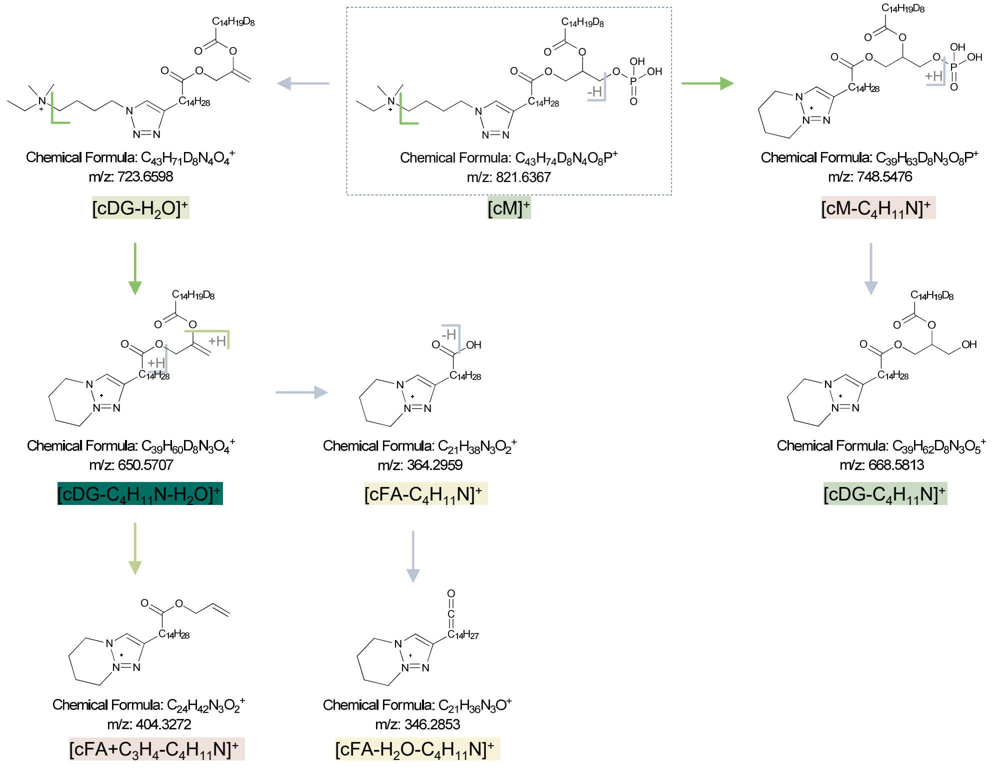

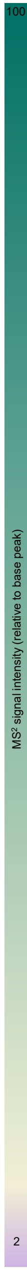

◂ Back to Content

PA-$^{13}C_3$ 19:1;C171_19:1;C171 532.9020 – $C_{54}{}^{13}C_3H_{107}N_8O_8P^{2+}$

Extracted Ion Chromatogram

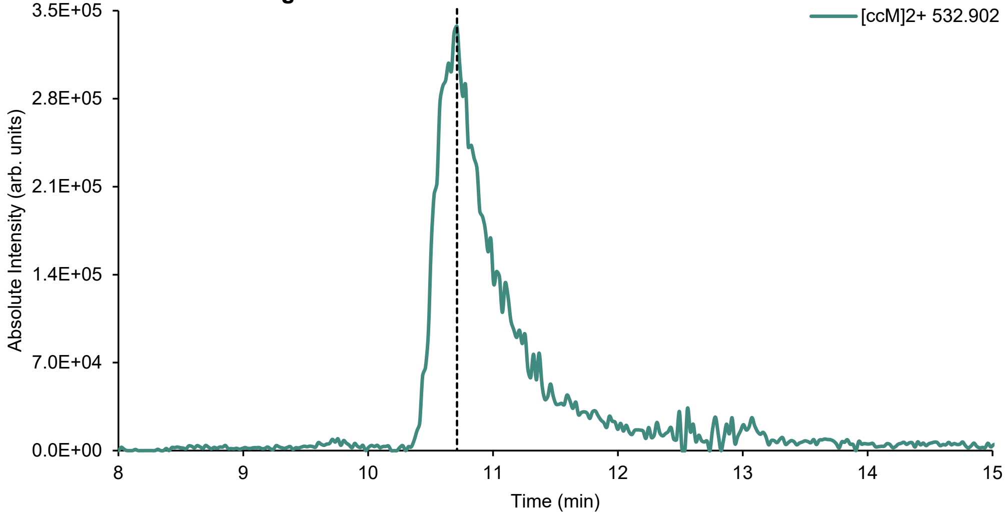

MS^2 [ccM]$^{2+}$

Ex_24_27_PN05 #6776 RT: 10.83 AV: 1 NL: 2.45E5
T: FTMS + p ESI d Full ms2 532.9015@hcd37.00 [110.8343-1108.3430]

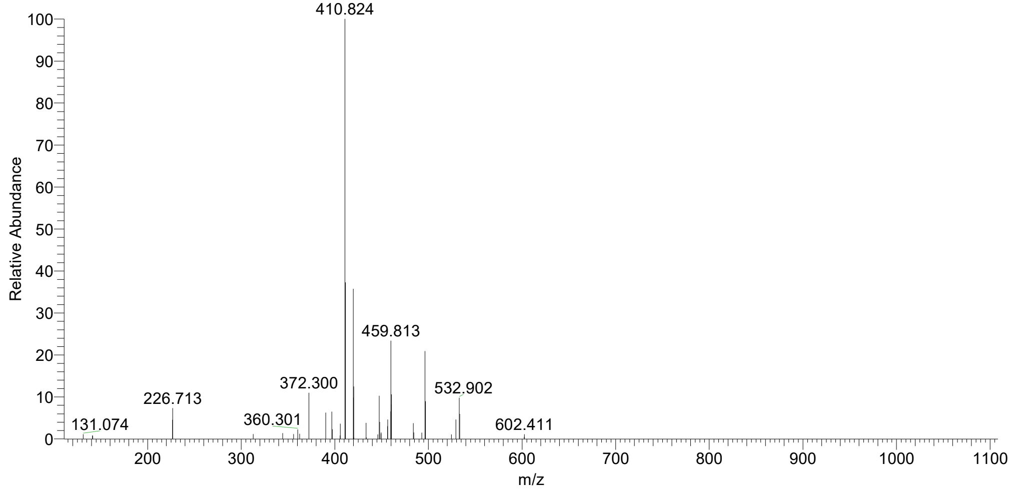

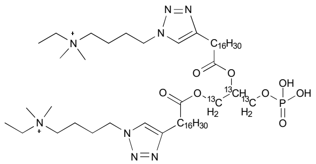

Chemical Formula: $C_{54}{}^{13}C_3H_{107}N_8O_8P^{2+}$
m/z: 532.9020

[ccM]$^{2+}$

◂ Back to Content

PA-$^{13}C_3$ 19:1;C171_19:1;C171 532.9020 – $C_{54}{}^{13}C_3H_{107}N_8O_8P^{2+}$ proposed fragmentation scheme

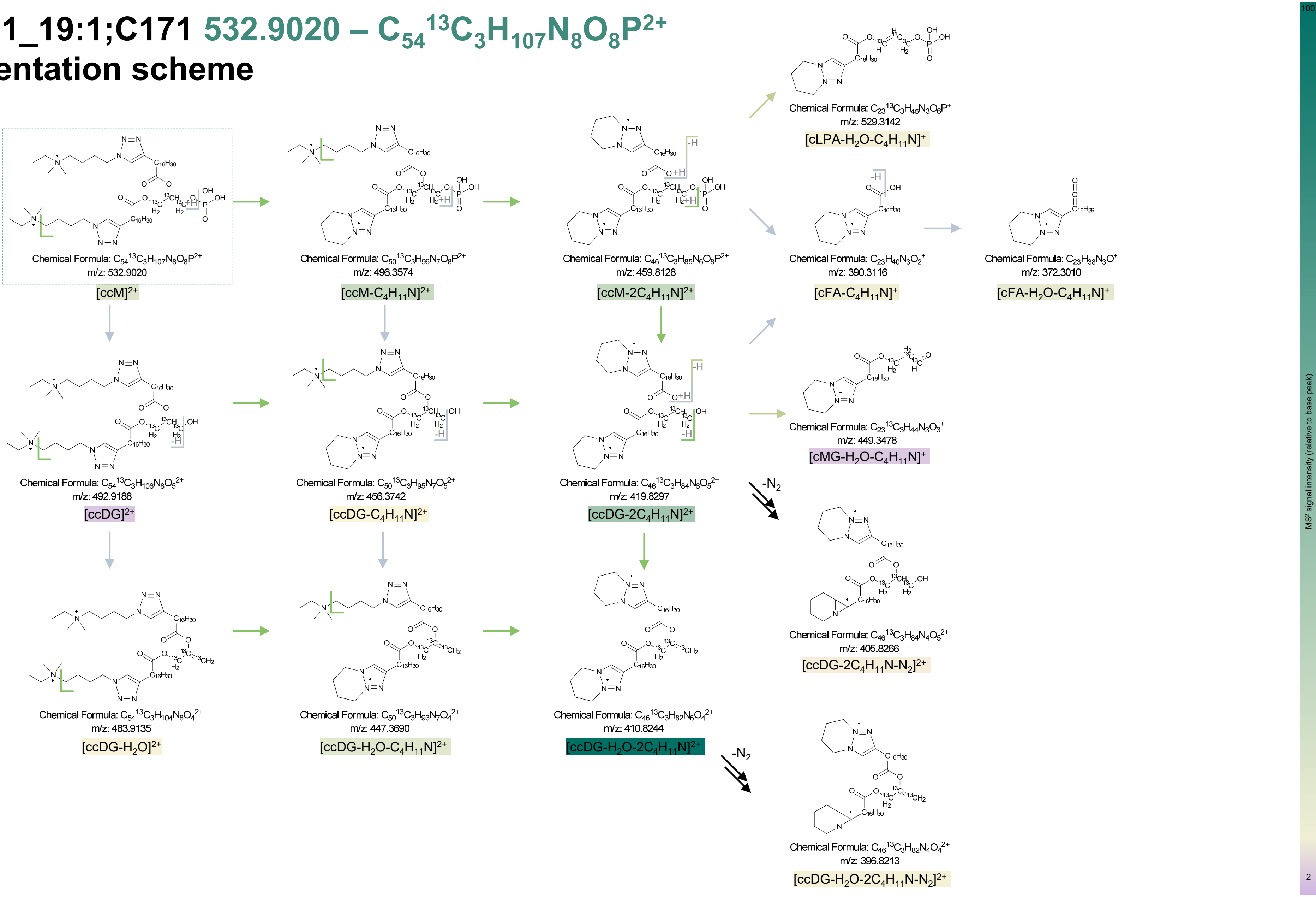

◂ Back to Content

PI 17:0;C171_15:1-d_8 983.6896 – $C_{49}H_{84}D_8N_4O_{13}P^+$

Extracted Ion Chromatogram

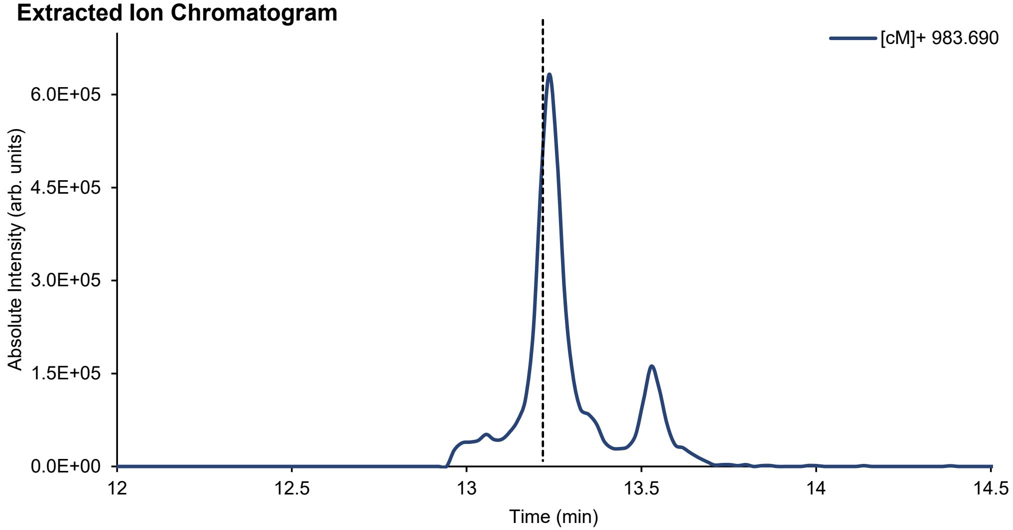

MS² [cM]⁺

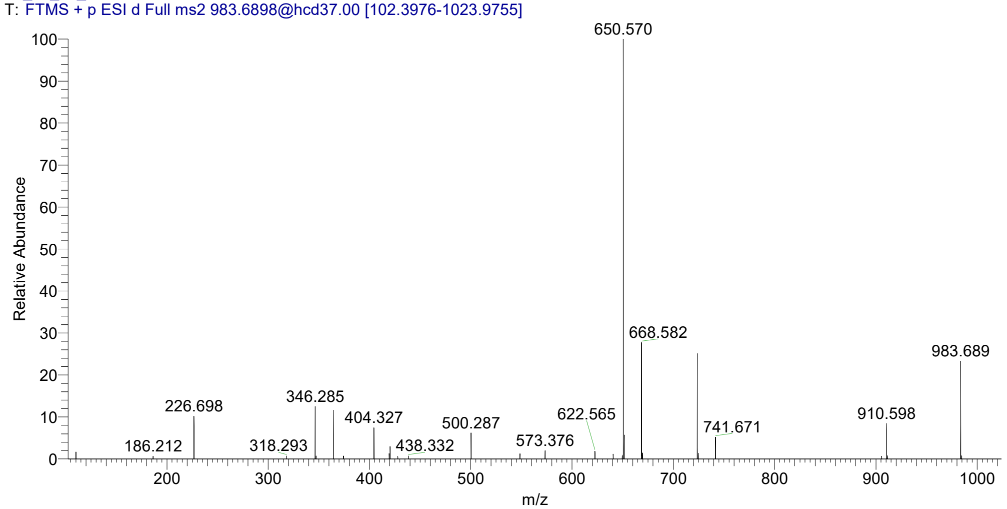

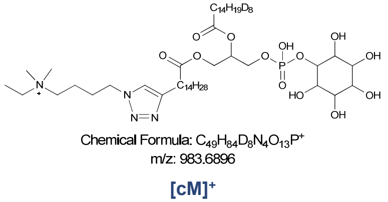

◂ Back to Content

PI 17:0;C171_15:1-d$_8$ 983.6896 – $C_{49}H_{84}D_8N_4O_{13}P^+$
proposed fragmentation scheme

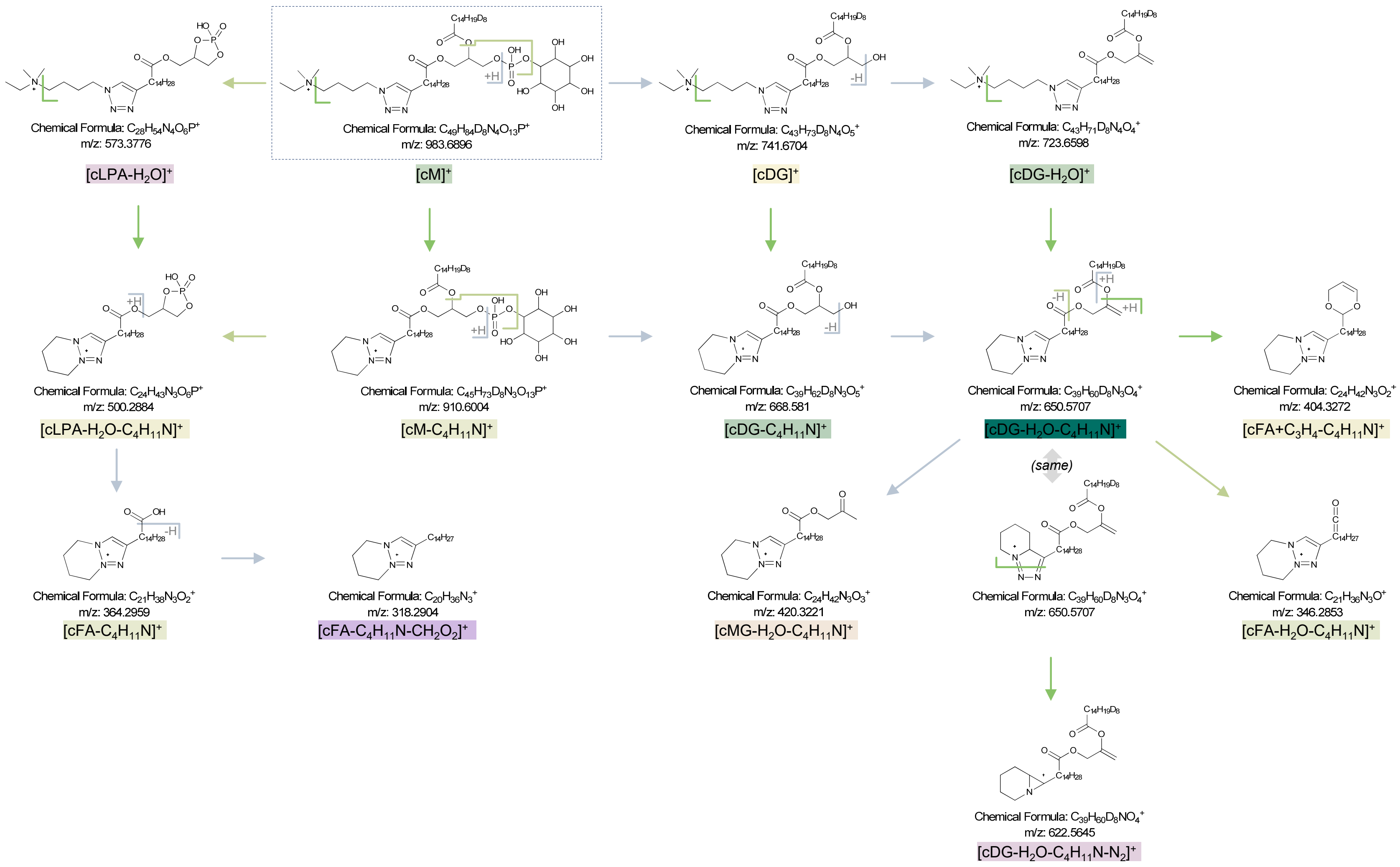

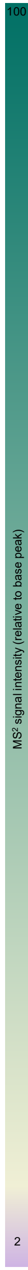

PS 17:0;C171_15:1-d_8 908.6688 – $C_{46}H_{79}D_8N_5O_{10}P^+$ / 454.8380 – $C_{46}H_{80}D_8N_5O_{10}P^{2+}$

Extracted Ion Chromatogram

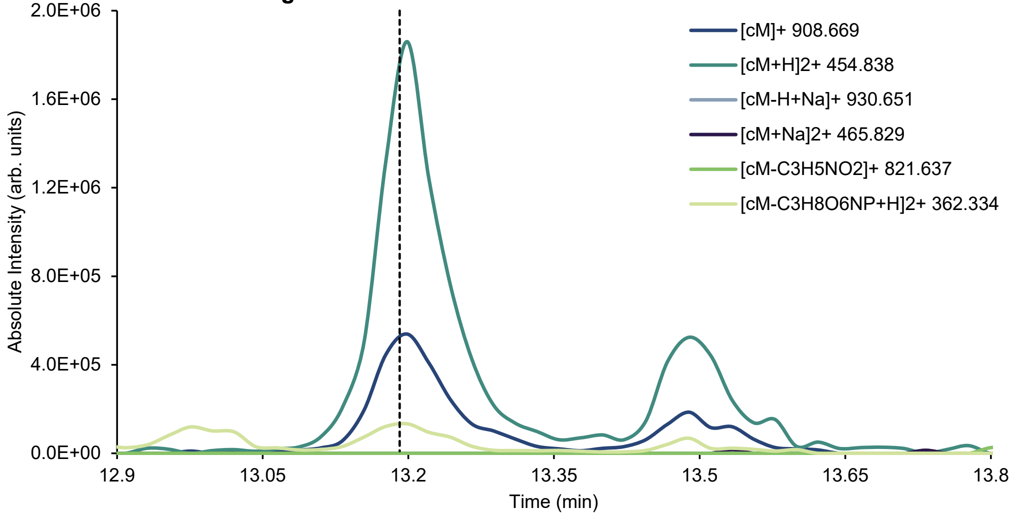

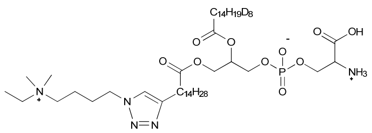

Chemical Formula: $C_{46}H_{79}D_8N_5O_{10}P^+$
m/z: 908.6688

[cM]⁺

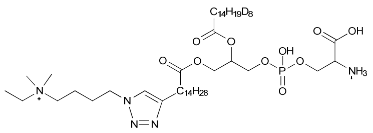

Chemical Formula: $C_{46}H_{80}D_8N_5O_{10}P^{2+}$
m/z: 454.8380

[cM+H]²⁺

MS² [cM]⁺

Ex_24_27_PN12 #8457 RT: 13.18 AV: 1 NL: 3.21E5
T: FTMS + p ESI d Full ms2 908.6680@hcd37.00 [94.7453-947.4533]

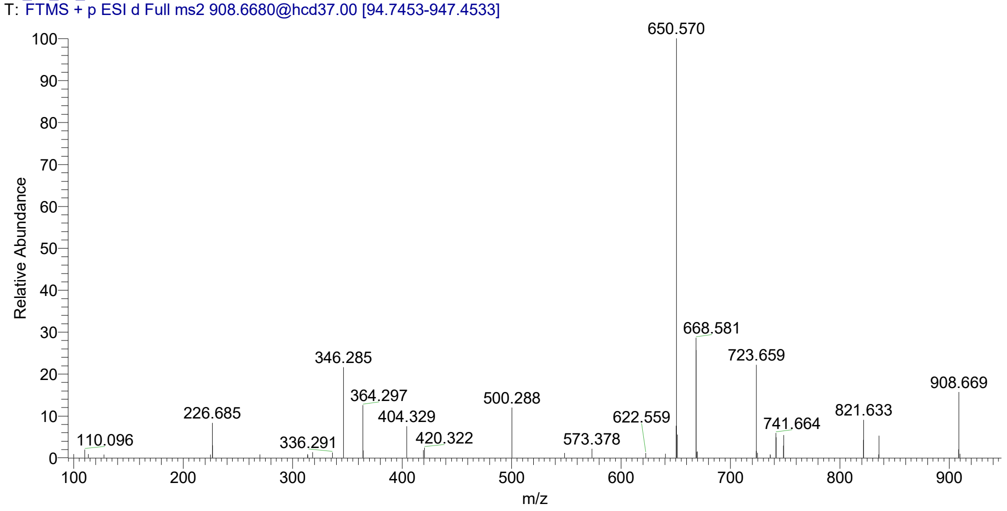

◂ Back to Content

PS 17:0;C171_15:1-d$_8$ 908.6688 – $C_{46}H_{79}D_8N_5O_{10}P^+$
proposed fragmentation scheme

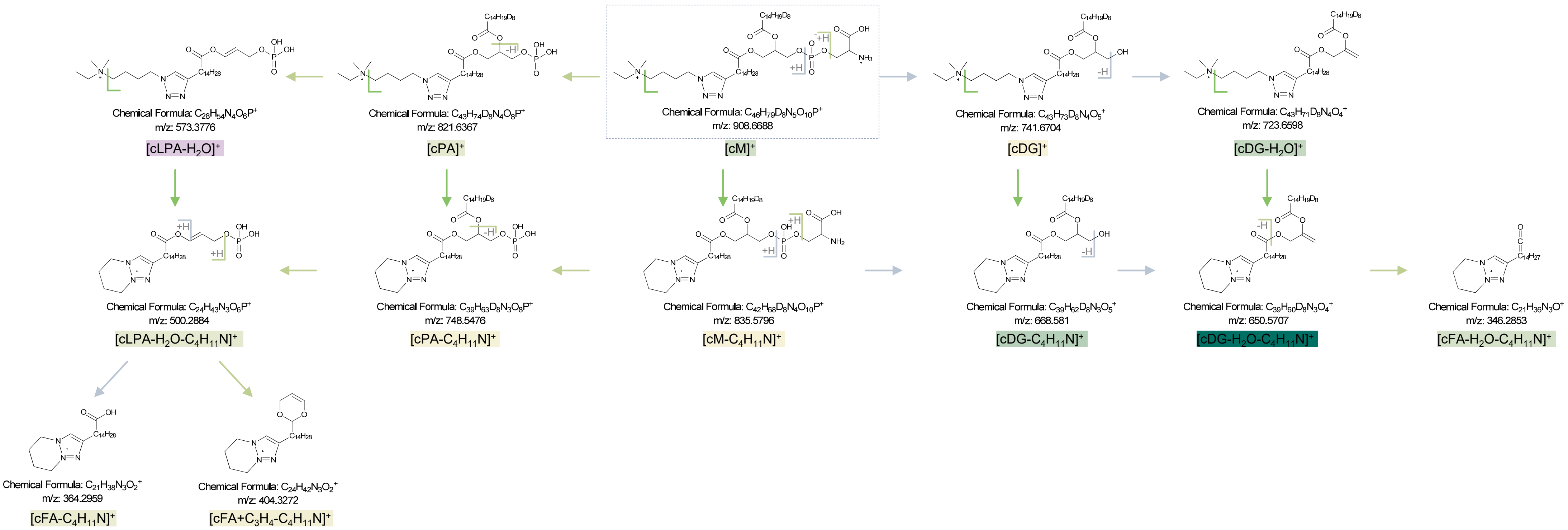

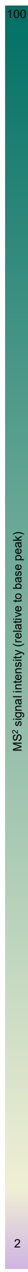

◂ Back to Content

Cer 18:0;O2,C171/15:1-d_8 698.6758 – $C_{41}H_{72}D_8O_3N_5^+$ / 349.8416 – $C_{41}H_{73}D_8O_3N_5^{2+}$

Extracted Ion Chromatogram

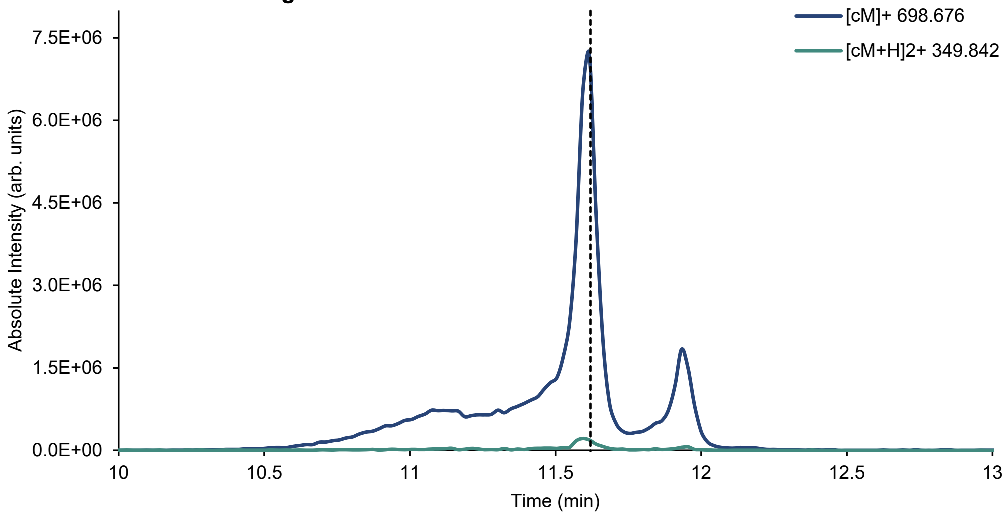

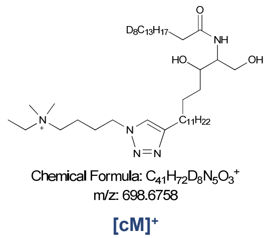

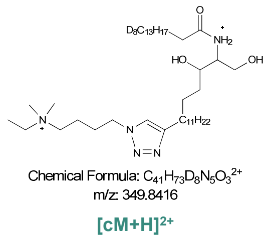

MS² [cM]⁺

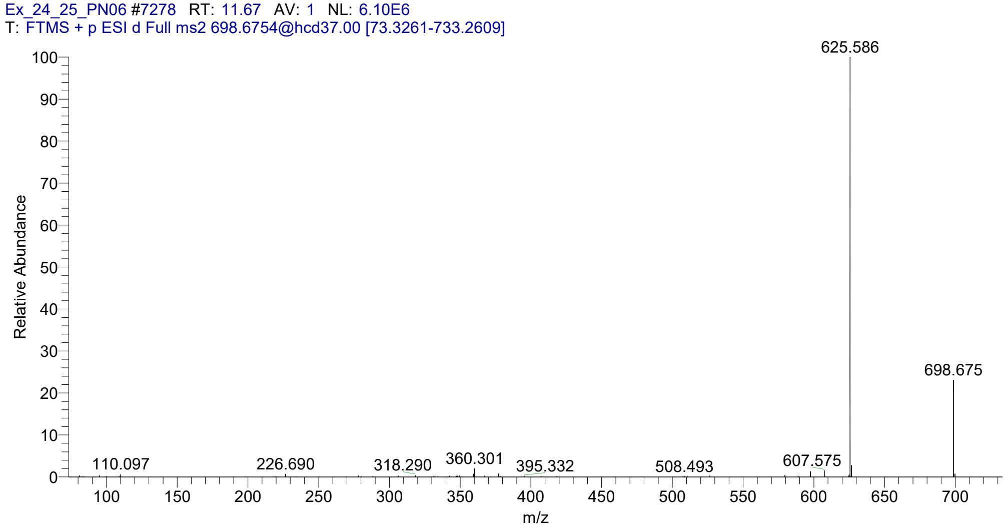

MS² [cM+H]²⁺

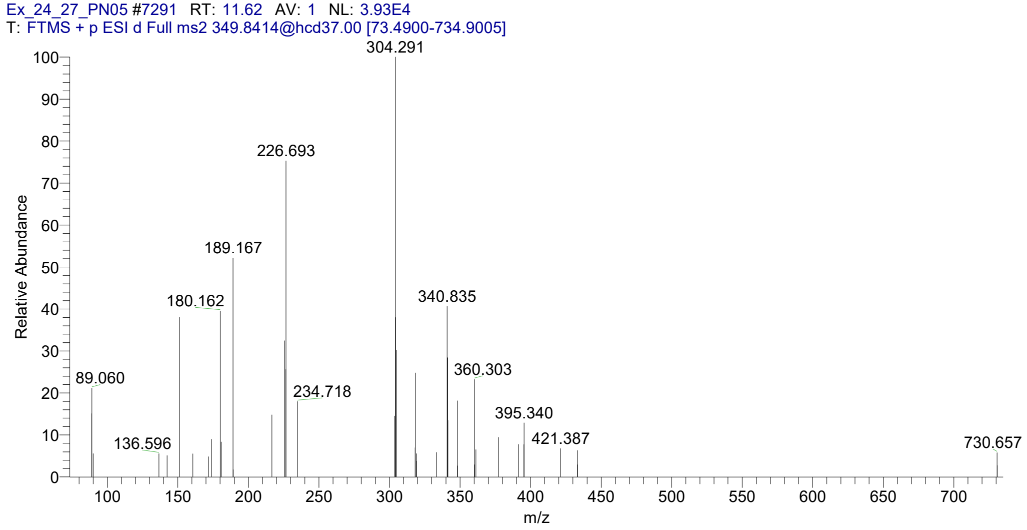

◂ Back to Content

Cer 18:0;O2,C171/15:1-d$_8$ 698.6758 – $C_{41}H_{72}D_8O_3N_5^+$
proposed fragmentation scheme

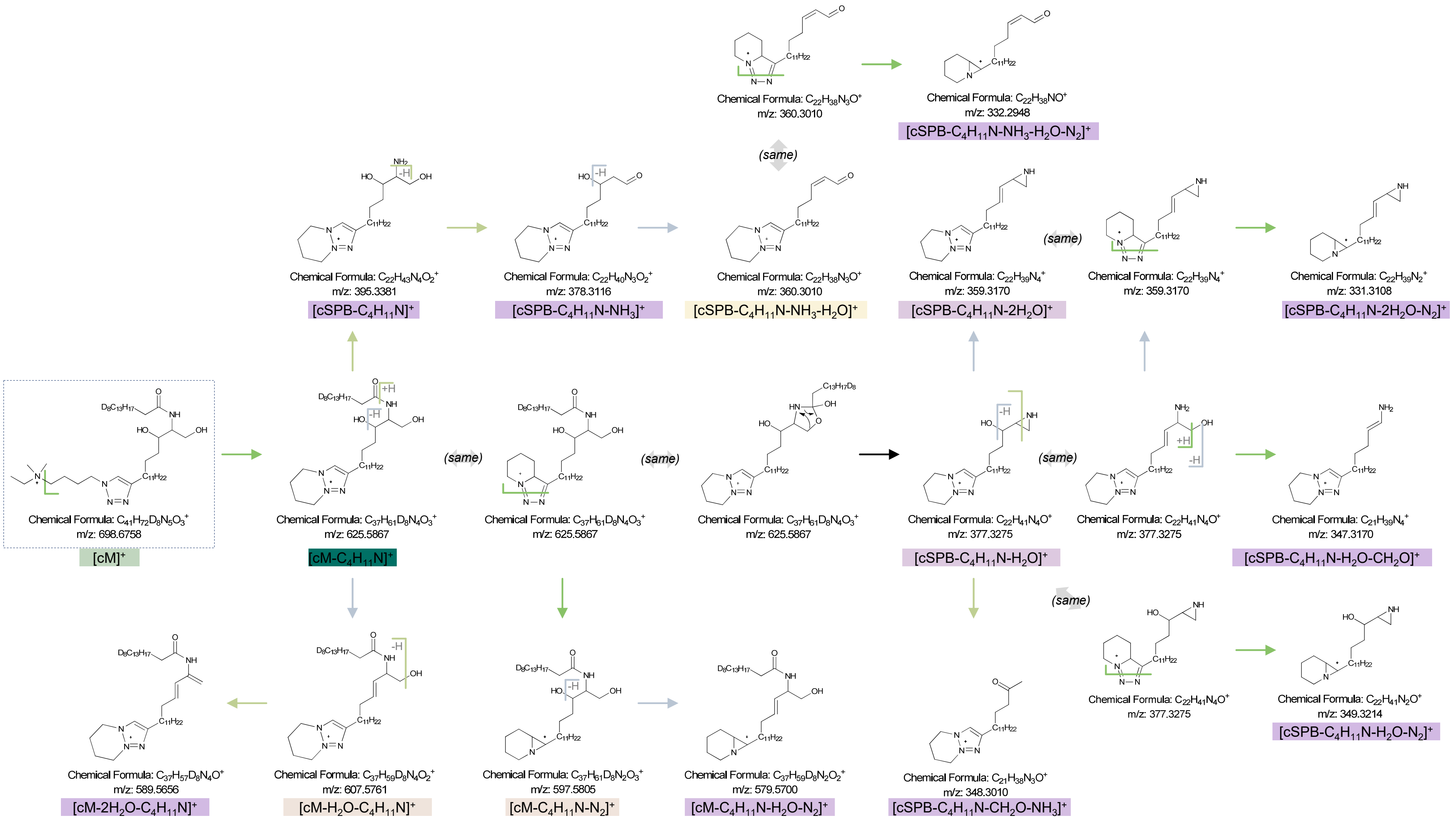

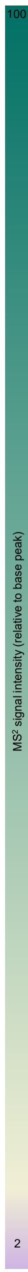

◂ Back to Content

Cer 18:0;O2,C171/15:1-d$_8$ 349.8416 – $C_{41}H_{73}D_8O_3N_5^{2+}$
proposed fragmentation scheme

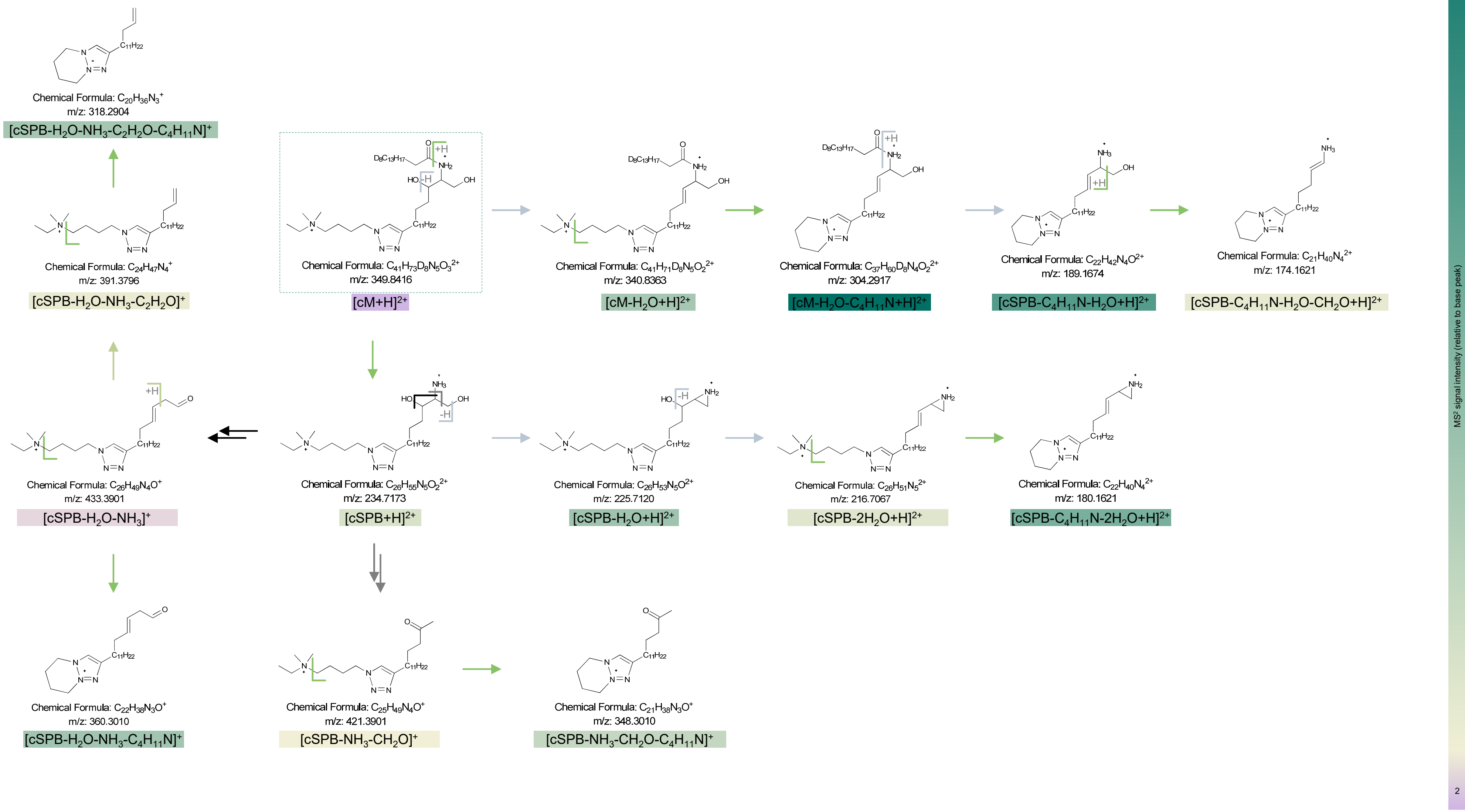

◂ Back to Content

HexCer 18:1;O2/15:0;C171-$^{13}C_2$ 854.6851 – $C_{45}{}^{13}C_2H_{90}O_8N_5{}^{+}$ / 427.8462 – $C_{45}{}^{13}C_2H_{91}O_8N_5{}^{2+}$

Extracted Ion Chromatogram

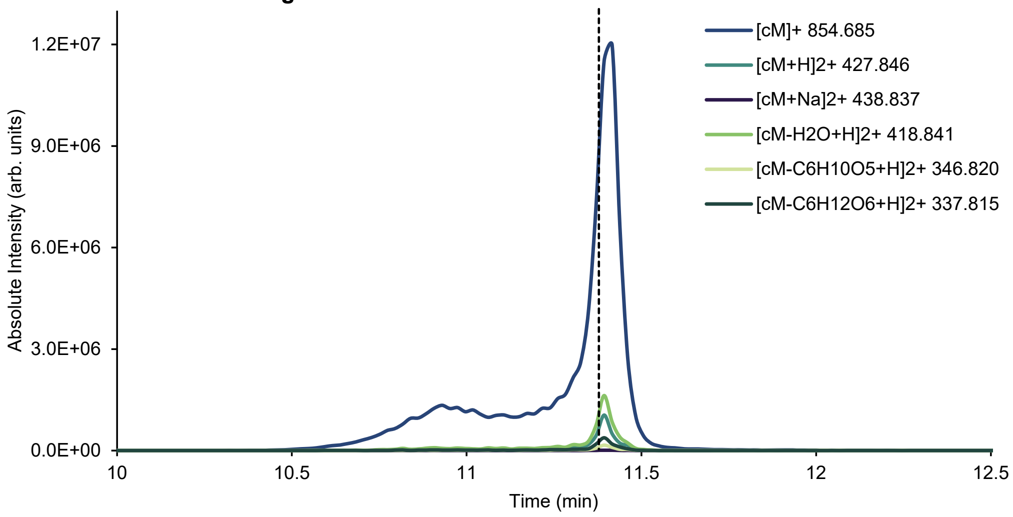

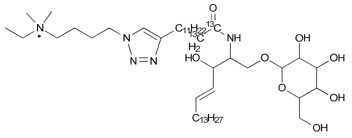

Chemical Formula: $C_{45}{}^{13}C_2H_{90}N_5O_8{}^{+}$
m/z: 854.6851

[cM]$^{+}$

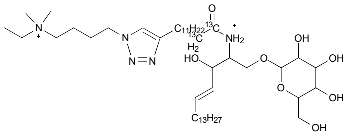

Chemical Formula: $C_{45}{}^{13}C_2H_{91}N_5O_8{}^{2+}$
m/z: 427.8462

[cM+H]$^{2+}$

MS^2 [cM]$^{+}$

Ex_24_25_PN06 #7117 RT: 11.42 AV: 1 NL: 9.29E6
T: FTMS + p ESI d Full ms2 854.6837@hcd37.00 [89.2389-892.3894]

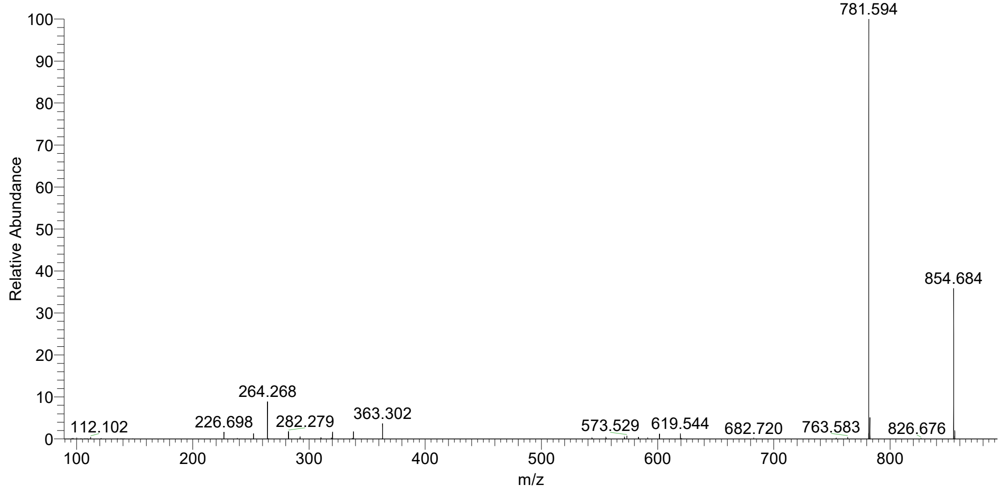

MS^2 [cM+H]$^{2+}$

Ex_24_25_PN06 #7118 RT: 11.42 AV: 1 NL: 3.08E5
T: FTMS + p ESI d Full ms2 427.8458@hcd37.00 [89.4029-894.0294]

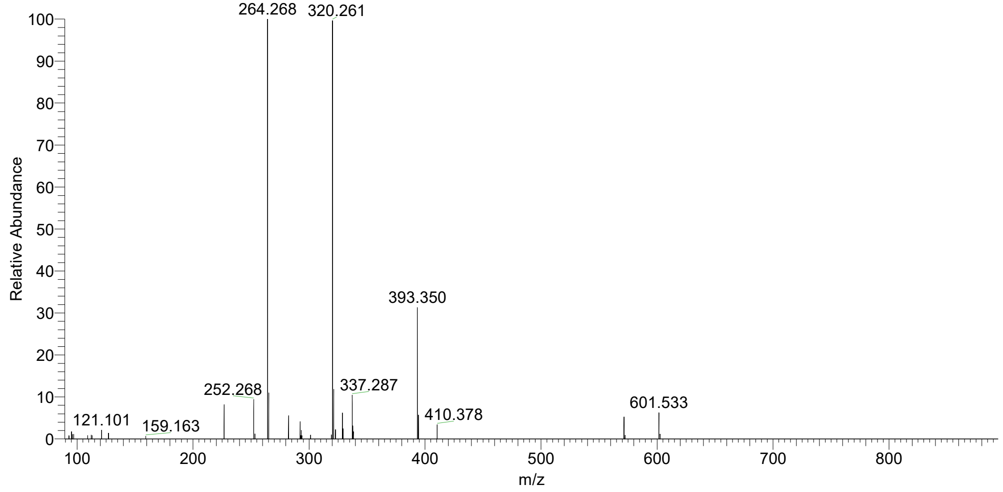

◂ Back to Content

HexCer 18:1;O2/15:0;C171-$^{13}C_2$ 854.6851 – $C_{45}{}^{13}C_2H_{90}O_8N_5{}^+$ proposed fragmentation scheme

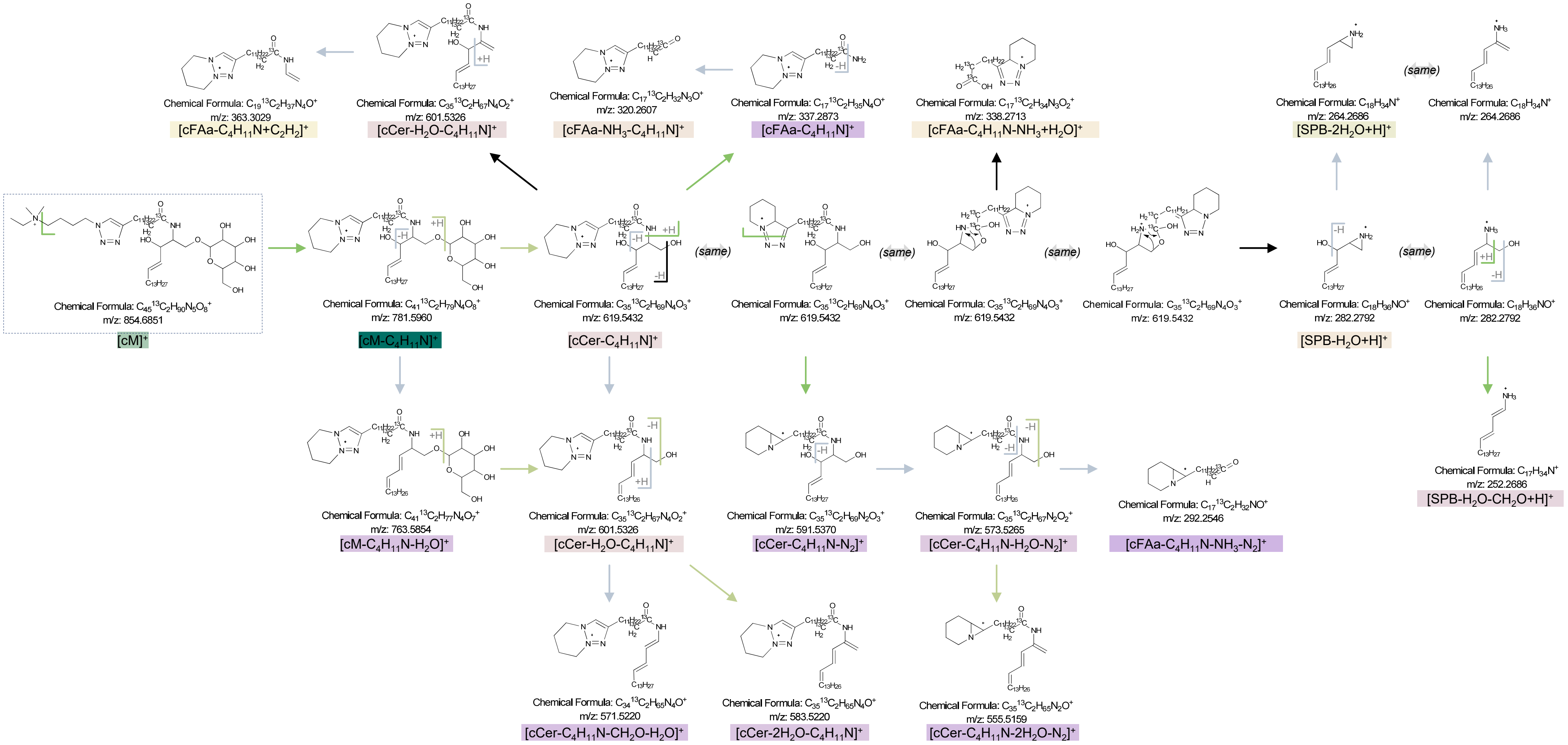

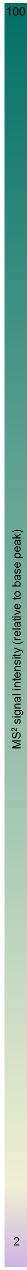

◂ Back to Content

HexCer 18:1;O2/15:0;C171-$^{13}C_2$ 427.8462 – $C_{45}{}^{13}C_2H_{91}O_8N_5{}^{2+}$
proposed fragmentation scheme

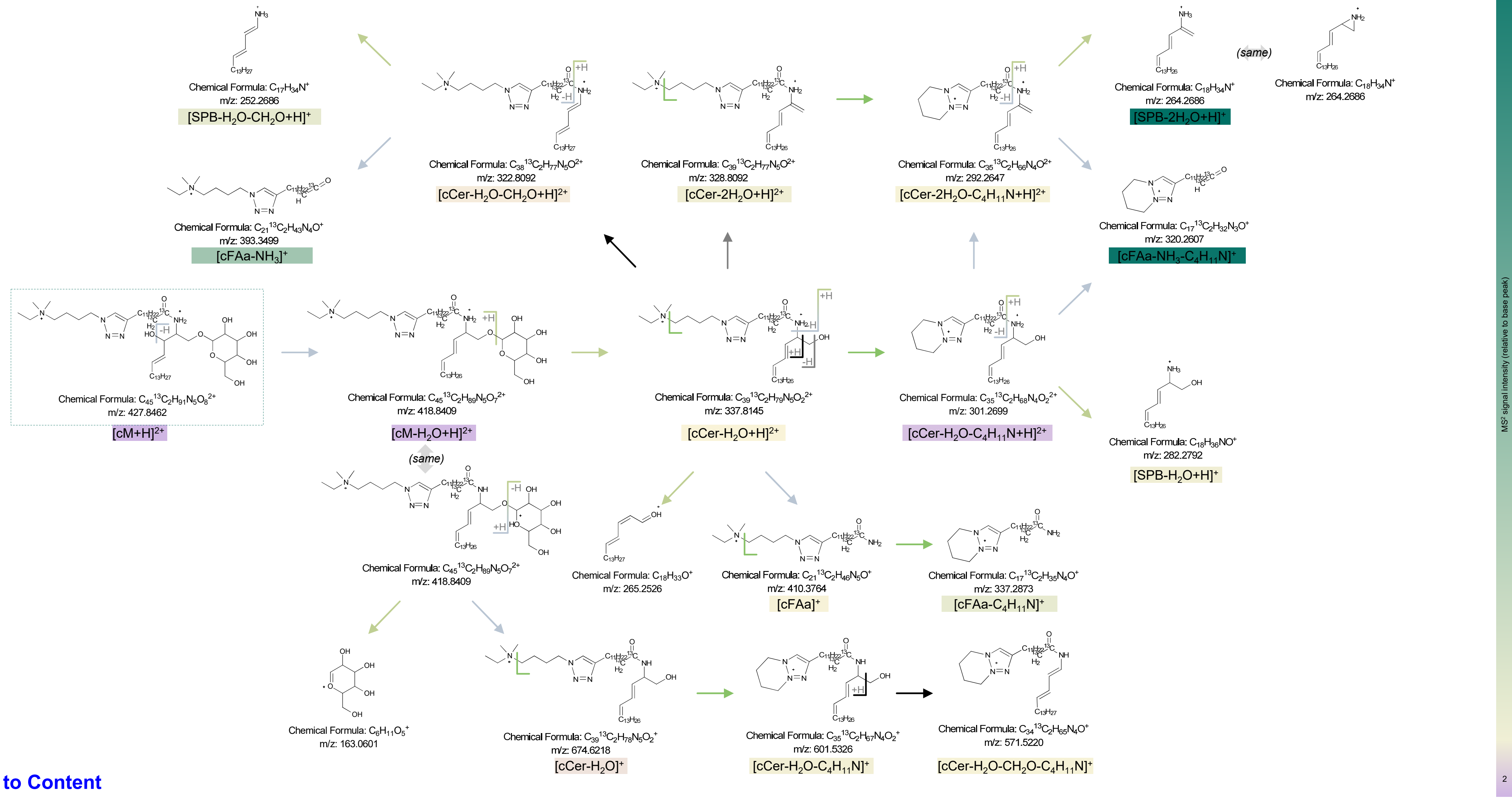

◂ Back to Content

SM 18:1;O2/15:0;C171-$^{13}C_2$ 857.6878 – $C_{44}{}^{13}C_2H_{92}O_6N_6P^+$ / 429.3475 – $C_{44}{}^{13}C_2H_{93}O_6N_6P^{2+}$

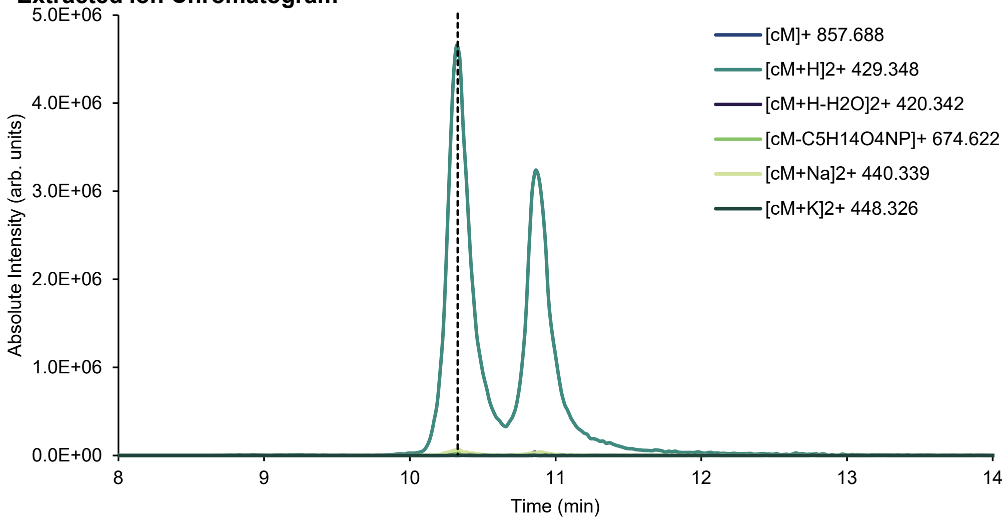

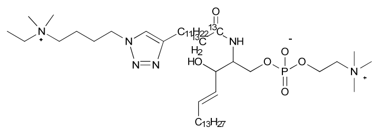
Chemical Formula: $C_{44}{}^{13}C_2H_{92}N_6O_6P^+$
m/z: 857.6878
[cM]$^+$

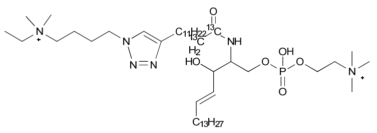
Chemical Formula: $C_{44}{}^{13}C_2H_{93}N_6O_6P^{2+}$
m/z: 429.3475
[cM+H]$^{2+}$

MS^2 [cM]$^+$

Ex_24_27_PN30_S25_V #6388 RT: 10.21 AV: 1 NL: 2.65E4
T: FTMS + p ESI d Full ms2 857.6870@hcd37.00 [89.5453-895.4527]

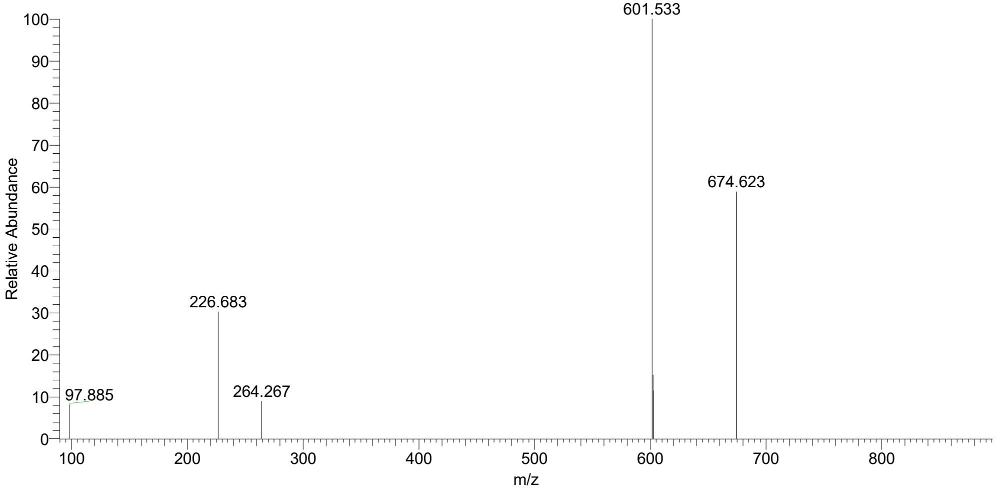

MS^2 [cM+H]$^{2+}$

Ex_24_27_PN30_S25_V #6381 RT: 10.20 AV: 1 NL: 5.23E6
T: FTMS + p ESI d Full ms2 429.3472@hcd37.00 [89.7092-897.0923]

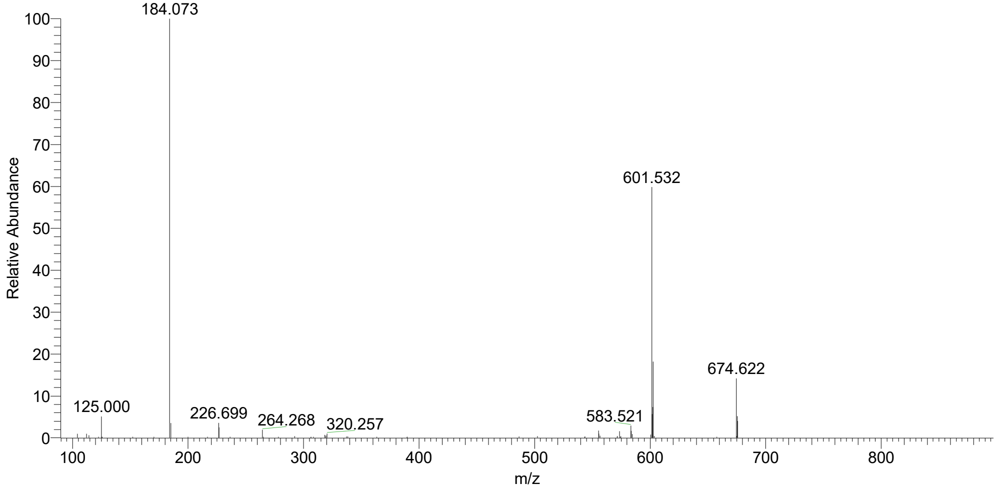

◂ Back to Content

SM 18:1;O2/15:0;C171-$^{13}C_2$ 857.6878 – $C_{44}{}^{13}C_2H_{92}O_6N_6P^+$ proposed fragmentation scheme

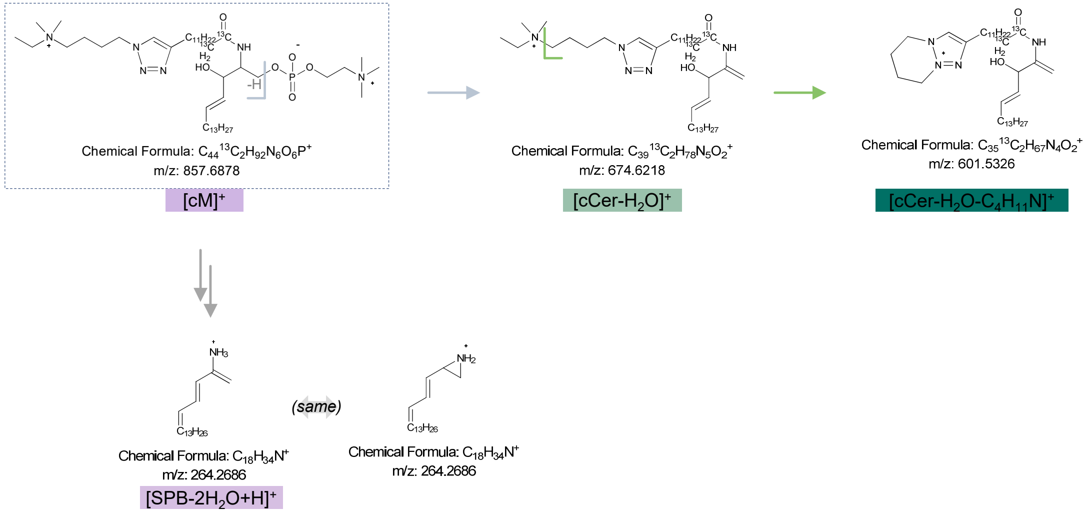

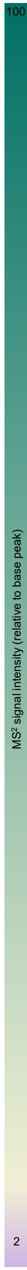

◂ Back to Content

SM 18:1;O2/15:0;C171-$^{13}C_2$ 429.3475 – $C_{44}{}^{13}C_2H_{93}O_6N_6P^{2+}$ proposed fragmentation scheme

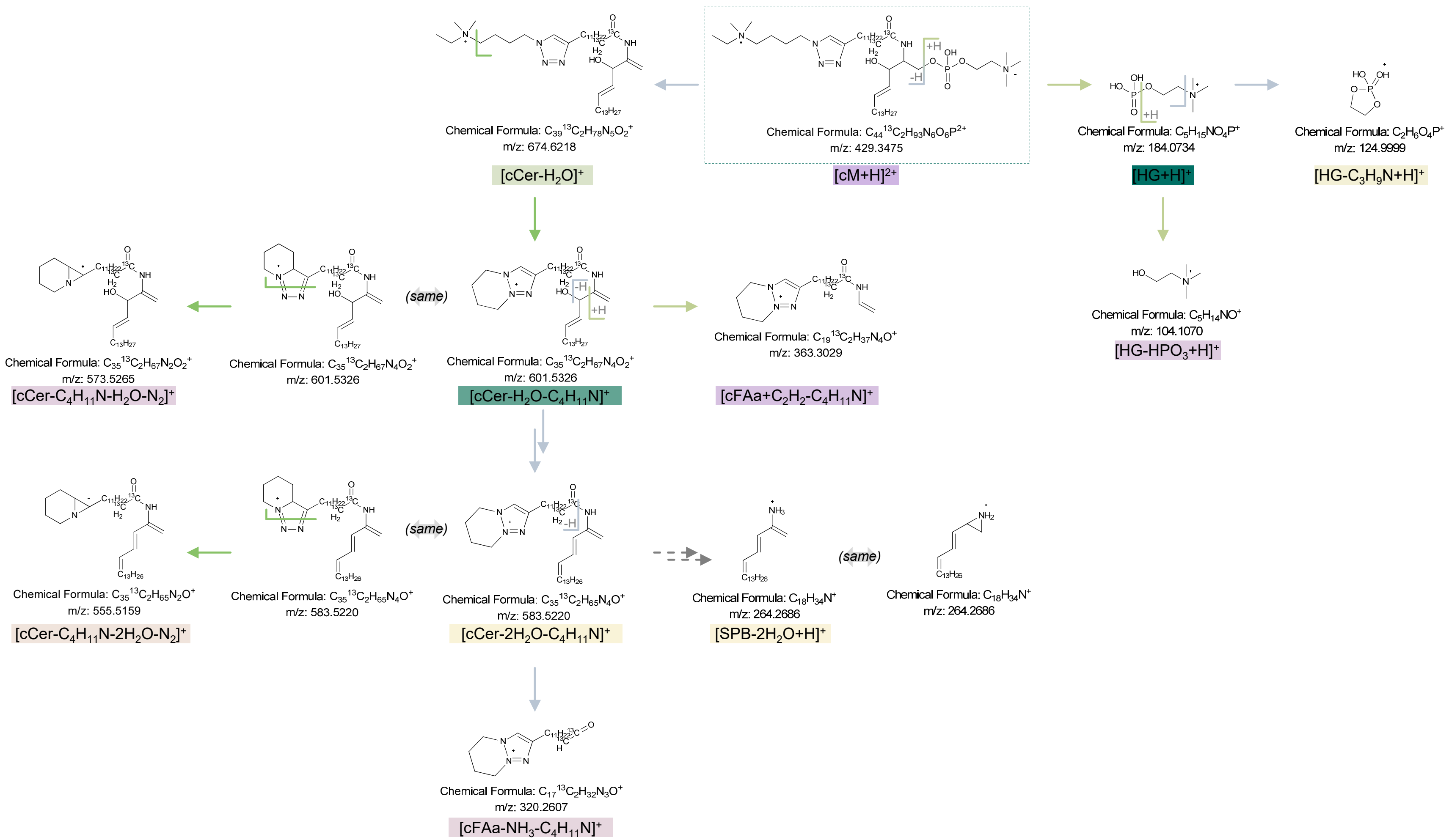

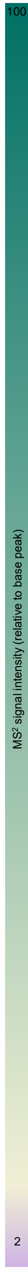

◂ Back to Content

MG-$^{13}C_3$ 19:1;C171 540.4475 – $C_{27}{}^{13}C_3H_{57}N_4O_4^+$

Extracted Ion Chromatogram

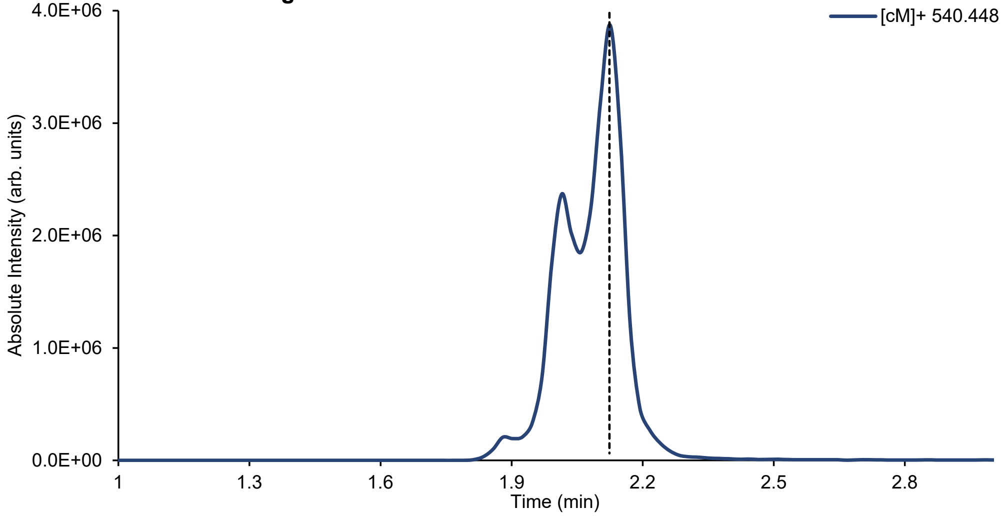

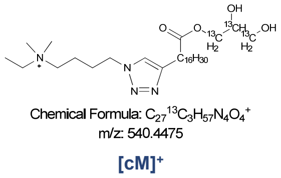

MS² [cM]⁺

Ex_24_27_PN01 #1301 RT: 2.11 AV: 1 NL: 4.31E6
T: FTMS + p ESI d Full ms2 540.4471@hcd37.00 [57.1868-571.8680]

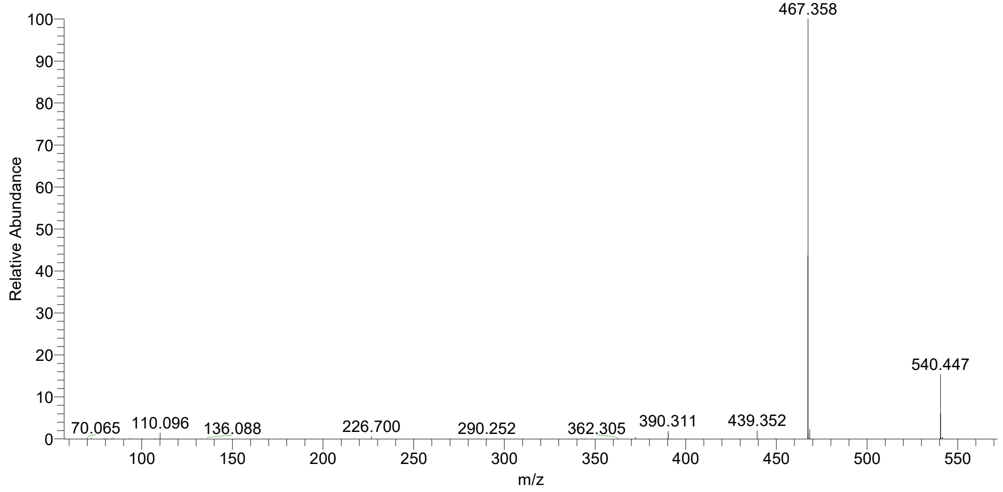

◂ Back to Content

MG-$^{13}C_3$ 19:1;C171 540.4475 – $C_{27}{}^{13}C_3H_{57}N_4O_4^+$
proposed fragmentation scheme

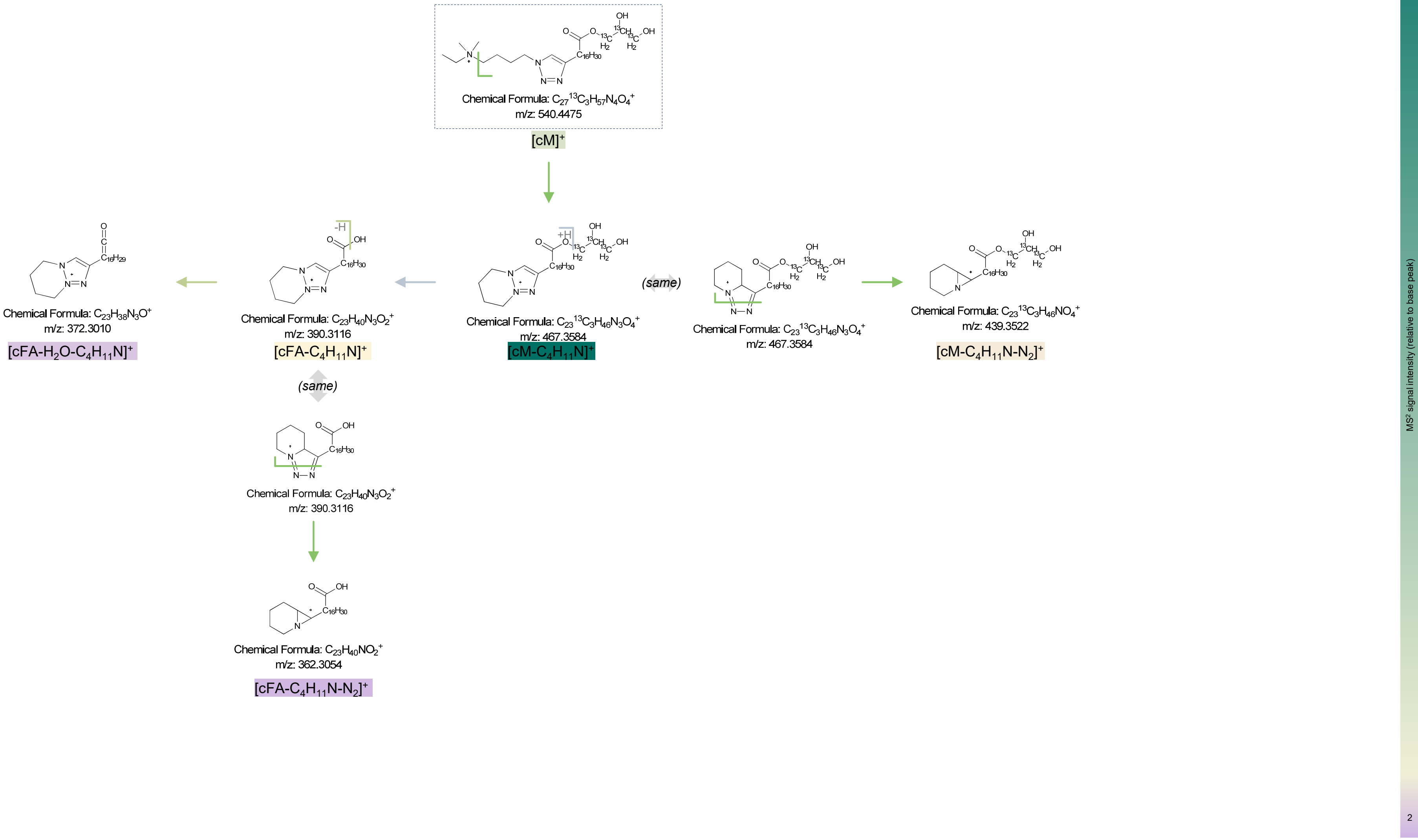

◂ Back to Content

DG 17:0;C171_15:1-d_8 741.6704 – $C_{43}H_{73}D_8N_4O_5^+$

Extracted Ion Chromatogram

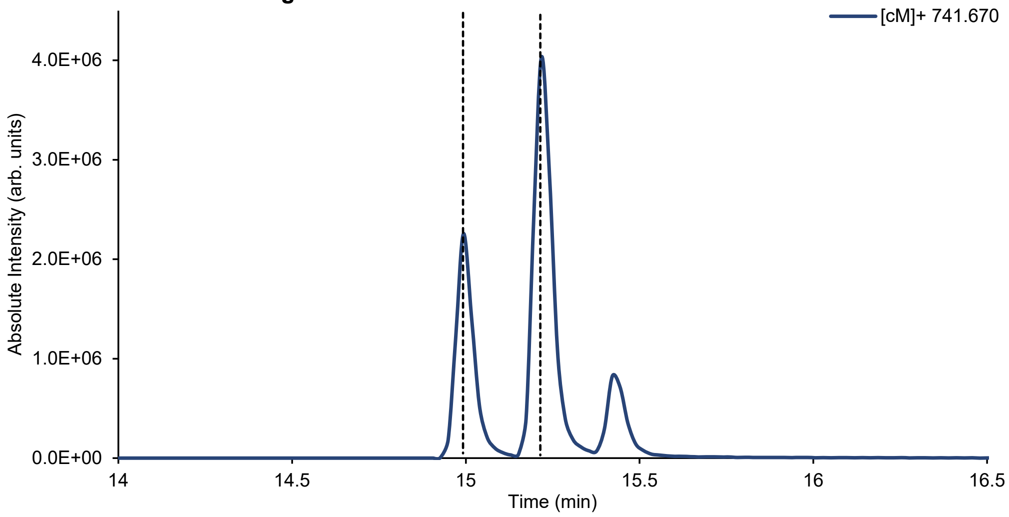

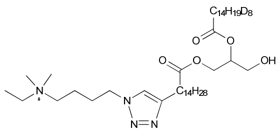

Chemical Formula: $C_{43}H_{73}D_8N_4O_5^+$
m/z: 741.6704

[cM]$^+$

MS^2 [cM]$^+$ RT 15.0 min

Ex_24_27_PN01 #9335 RT: 14.98 AV: 1 NL: 1.85E6
T: FTMS + p ESI d Full ms2 741.6703@hcd37.00 [77.7116-777.1157]

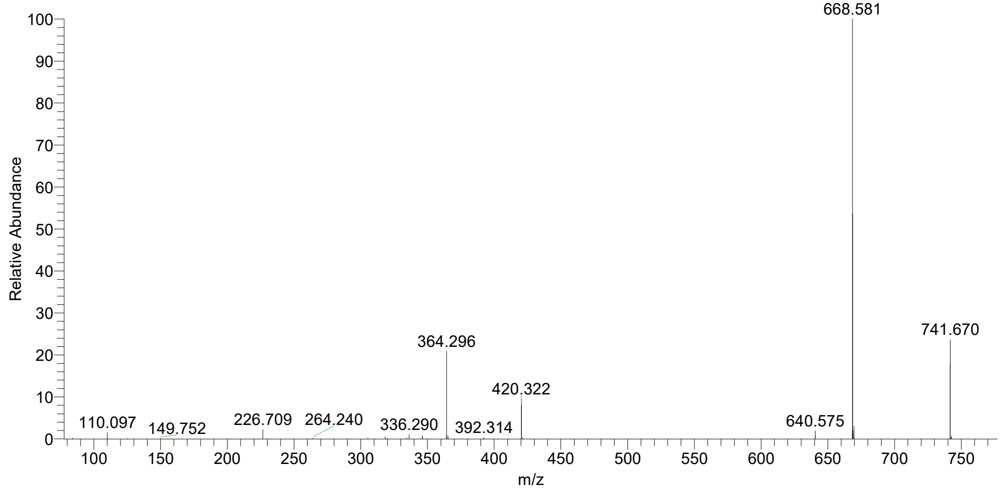

MS^2 [cM]$^+$ RT 15.2 min

Ex_24_27_PN01 #9475 RT: 15.20 AV: 1 NL: 3.95E6
T: FTMS + p ESI d Full ms2 741.6701@hcd37.00 [77.7115-777.1155]

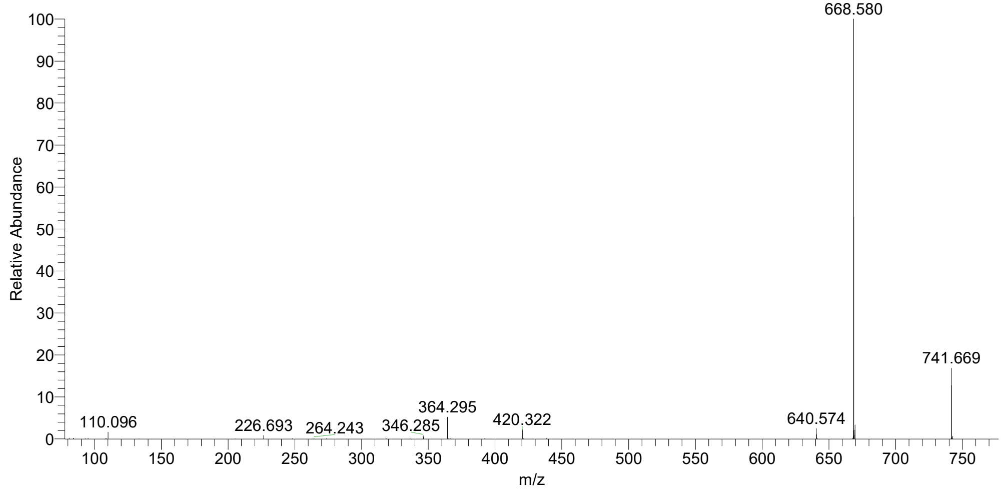

◂ Back to Content

DG 17:0;C171_15:1-d$_8$ 741.6704 – $C_{43}H_{73}D_8N_4O_5^+$
proposed fragmentation scheme

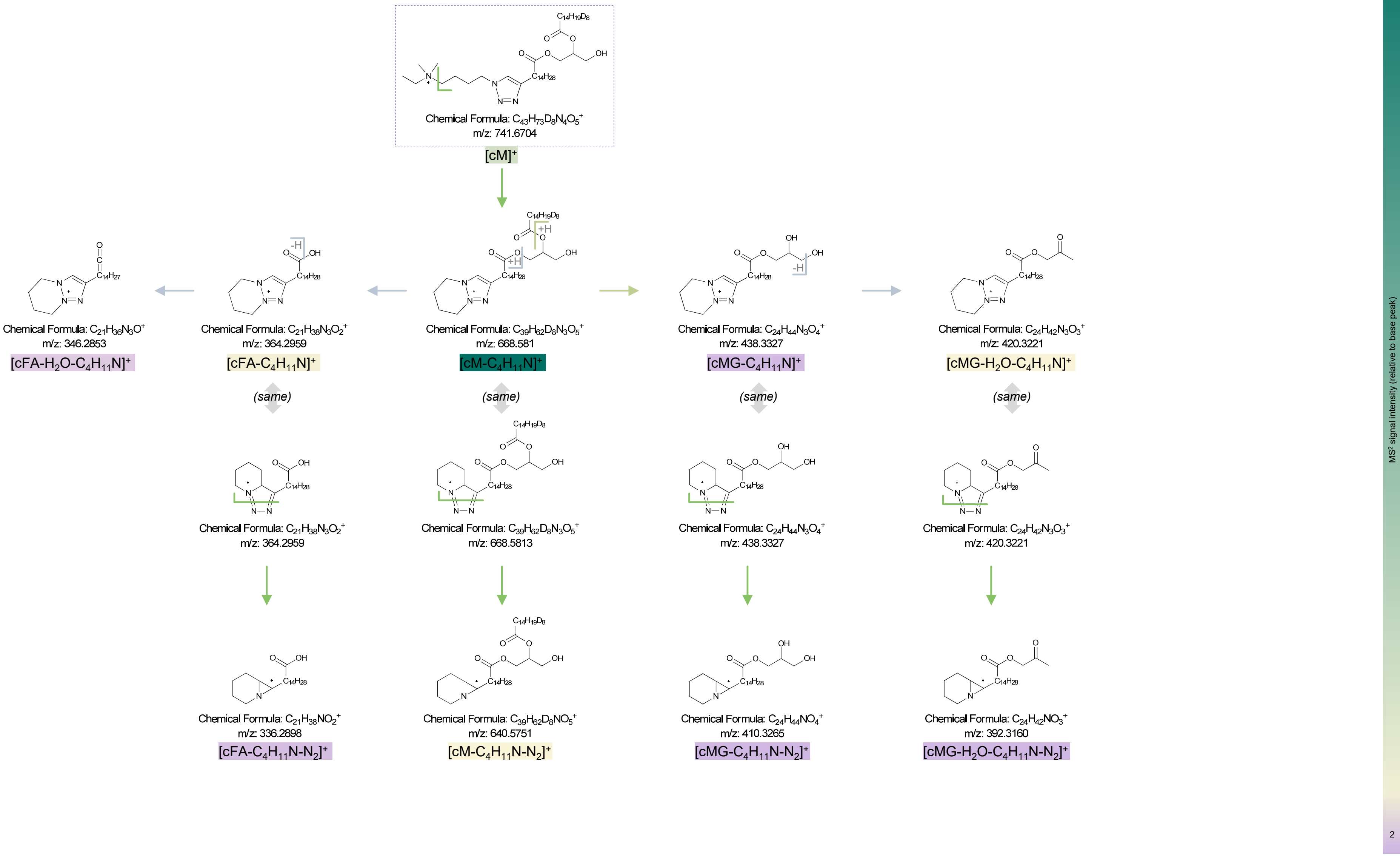

◂ Back to Content

DG-$^{13}C_3$ 19:1;C171_19:1;C171 492.9188 – $C_{54}{}^{13}C_3H_{106}N_8O_5{}^{2+}$

Extracted Ion Chromatogram

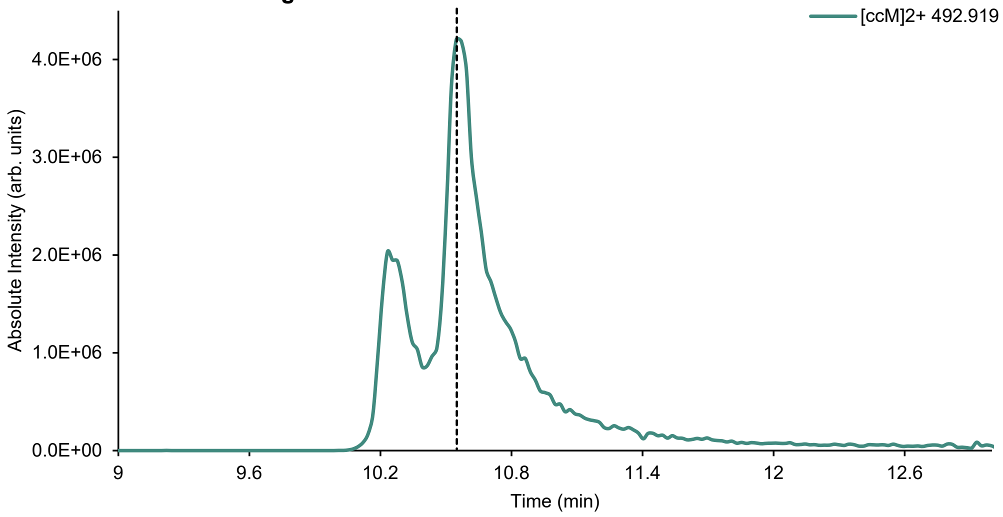

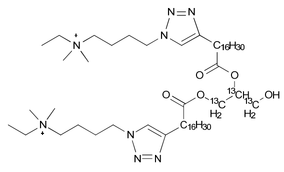

MS² [ccM]²⁺

Ex_24_25_PN08 #6552 RT: 10.53 AV: 1 NL: 7.08E6
T: FTMS + p ESI d Full ms2 492.9187@hcd37.00 [102.6778-1026.7782]

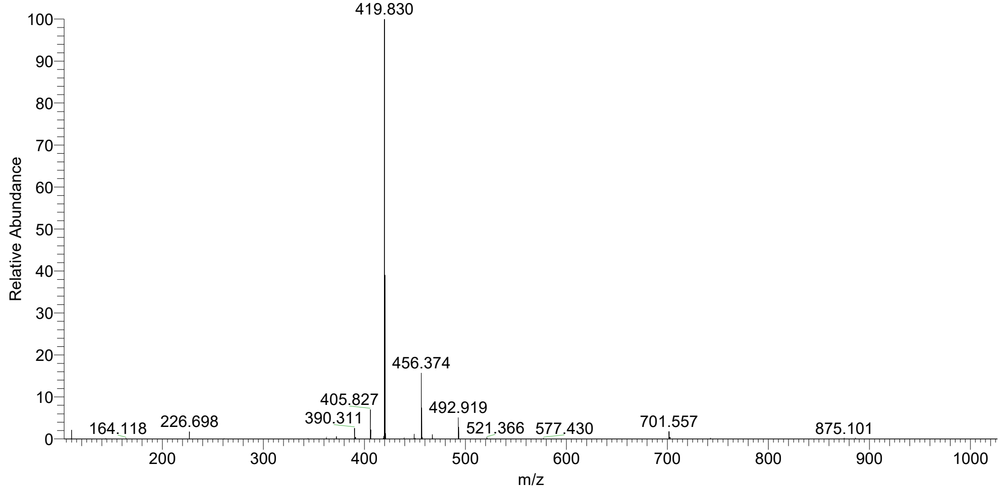

◂ Back to Content

DG-$^{13}C_3$ 19:1;C171_19:1;C171 492.9188 – $C_{54}{}^{13}C_3H_{106}N_8O_5{}^{2+}$ proposed fragmentation scheme

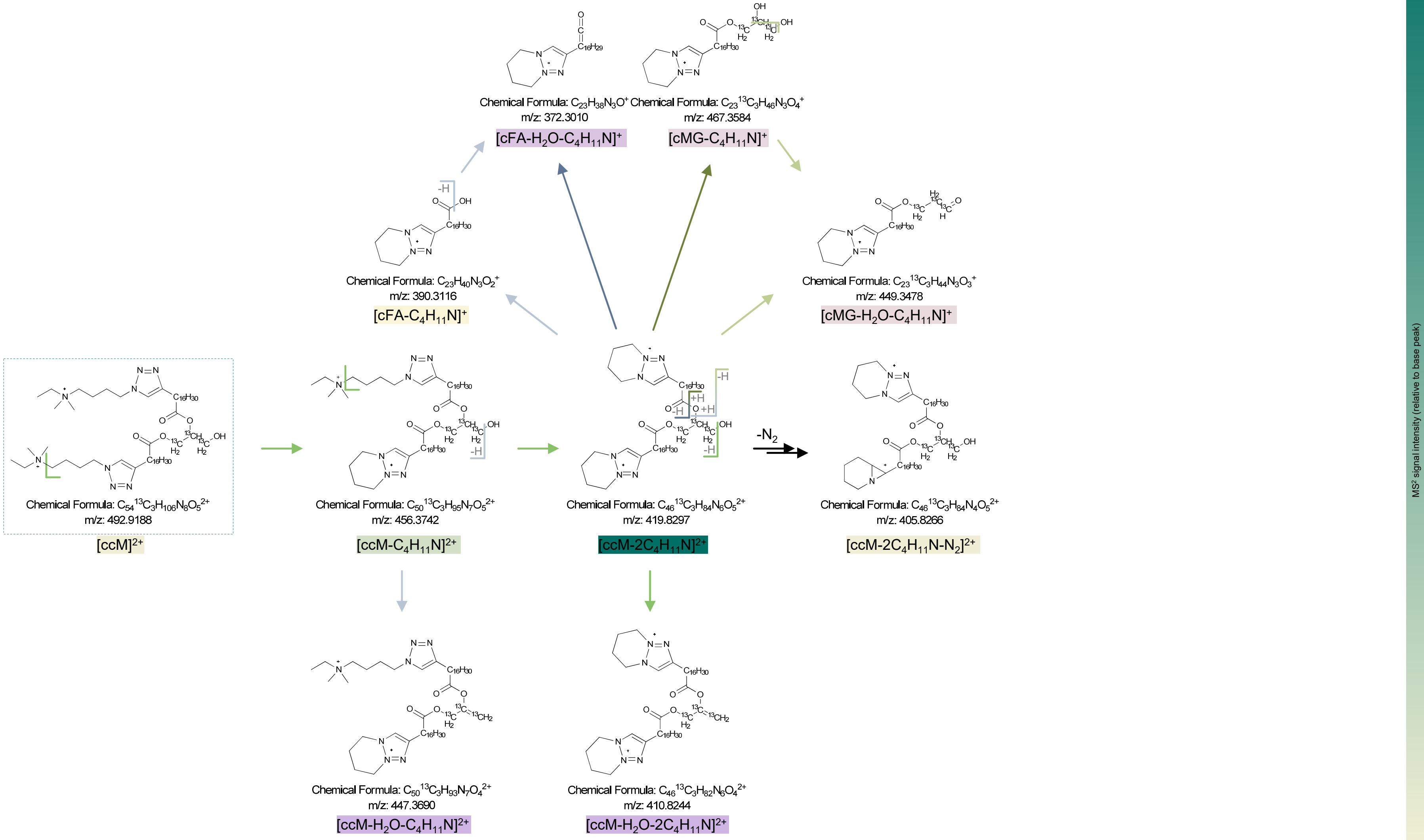

◂ Back to Content

TG 17:0;C171_15:1-d_8_16:0 979.9001 – $C_{59}H_{103}D_8N_4O_6^+$

Extracted Ion Chromatogram

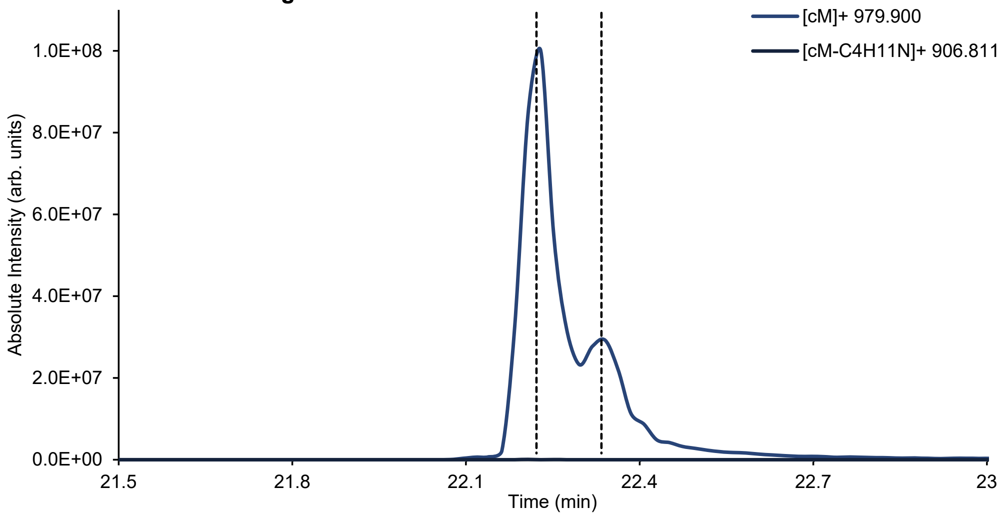

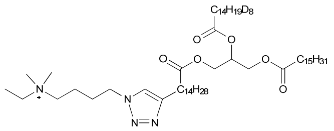

MS² [cM]⁺ RT 22.2 min

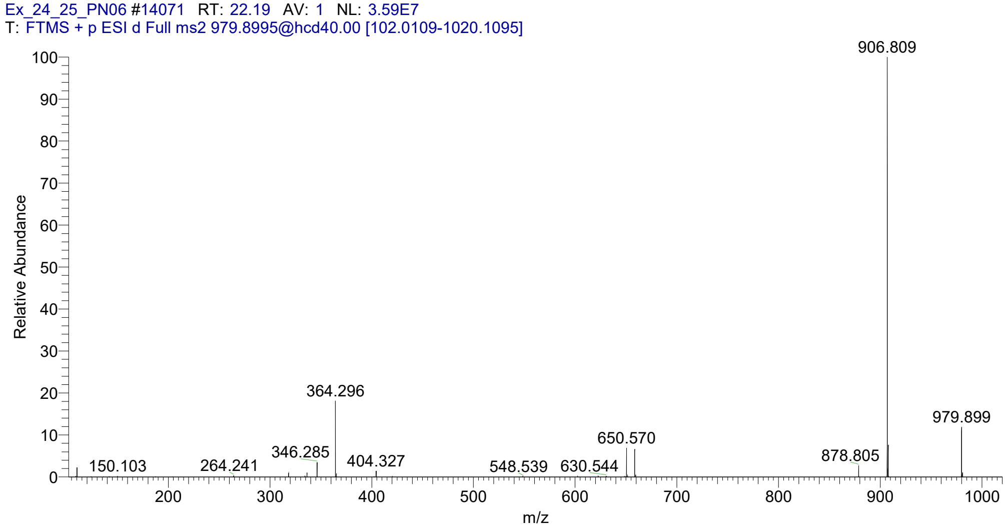

MS² [cM]⁺ RT 22.4 min

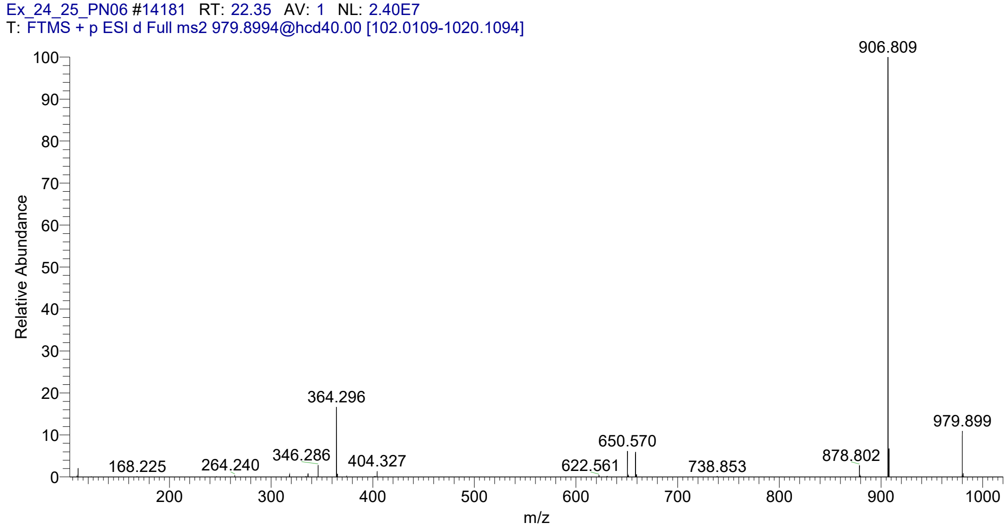

◂ Back to Content

TG 17:0;C171_15:1-d_8_16:0 979.9001 – $C_{59}H_{103}D_8N_4O_6^+$
proposed fragmentation scheme

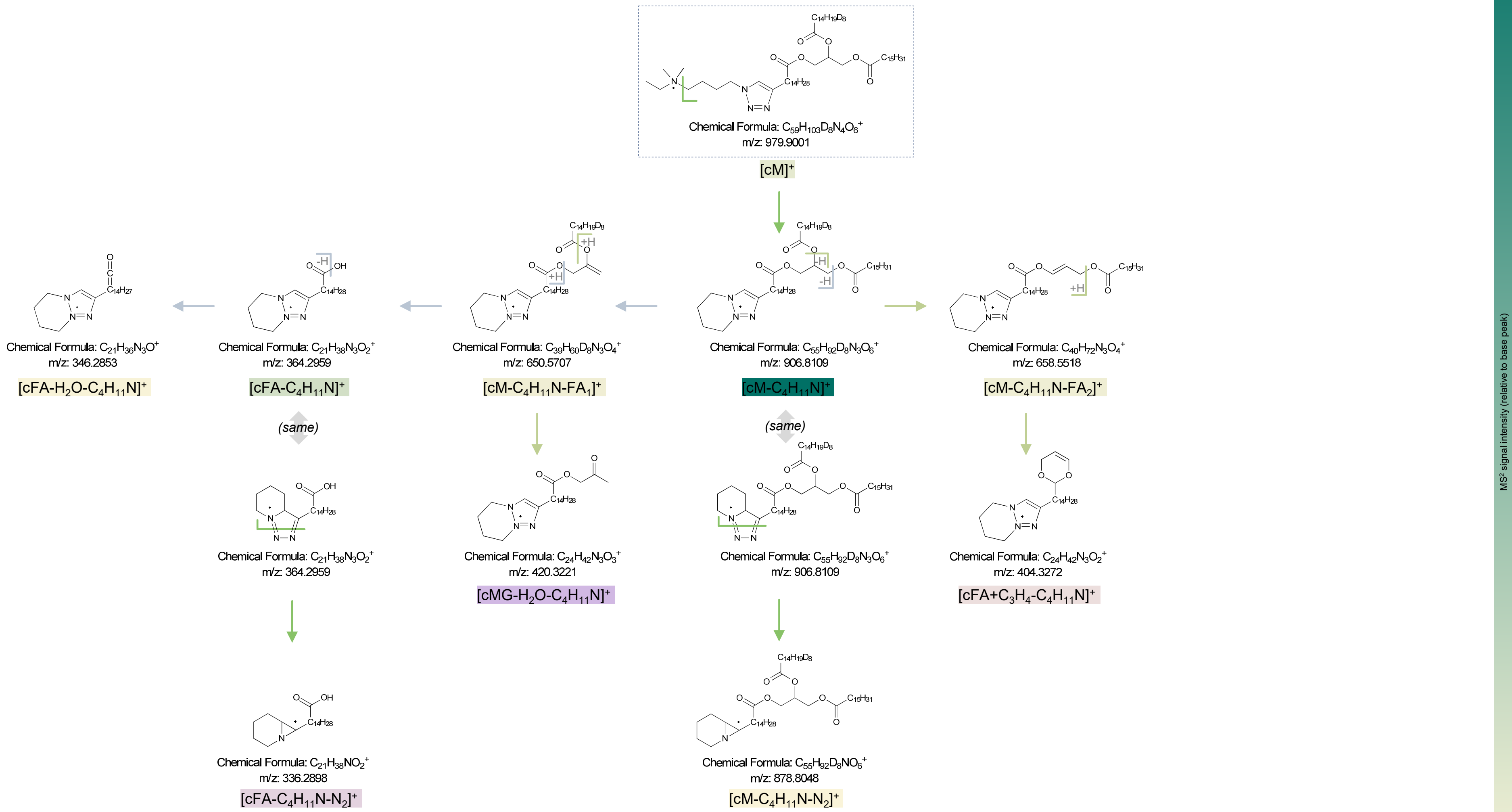

◂ Back to Content

TG 17:0;C171_17:0;C171_15:1-d_8 580.5224 – $C_{68}H_{120}D_8N_8O_6^{2+}$

Extracted Ion Chromatogram

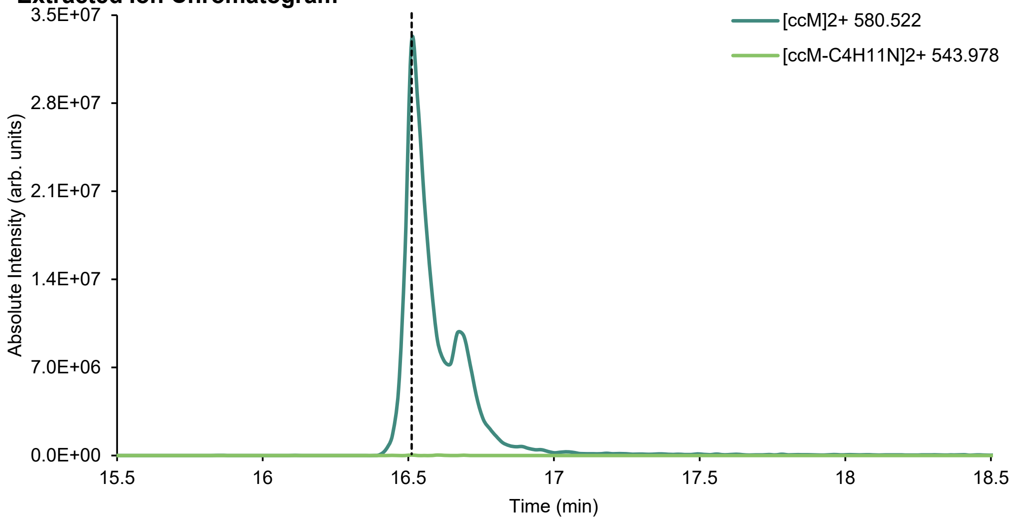

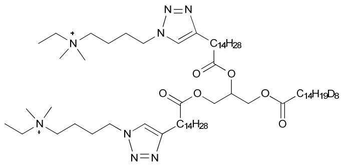

Chemical Formula: $C_{68}H_{120}D_8N_8O_6^{2+}$
m/z: 580.5224

[ccM]$^{2+}$

MS² [ccM]$^{2+}$

Ex_24_27_PN04 #10705 RT: 16.58 AV: 1 NL: 1.80E7
T: FTMS + p ESI d Full ms2 580.5222@hcd37.00 [120.5489-1205.4894]

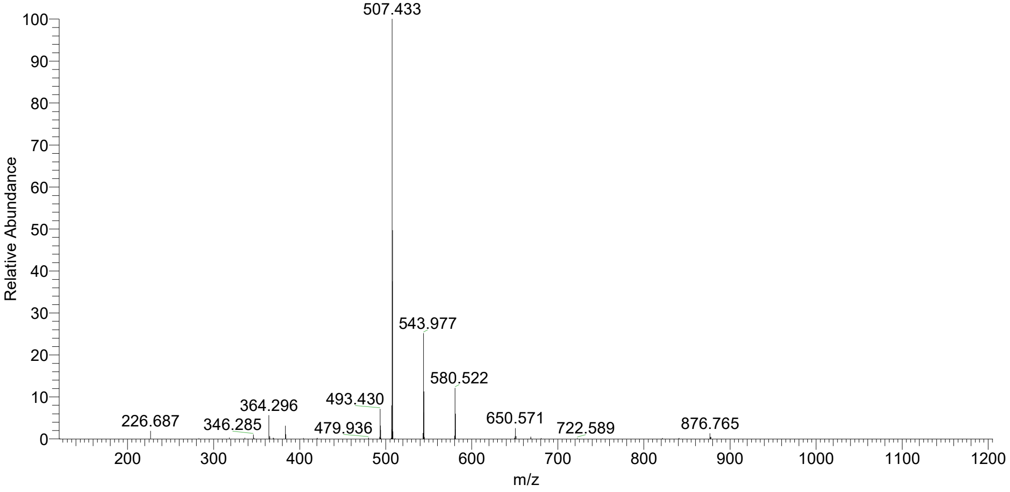

◂ Back to Content

TG 17:0;C171_17:0;C171_15:1-d_8 580.5224 – $C_{68}H_{120}D_8N_8O_6^{2+}$
proposed fragmentation scheme

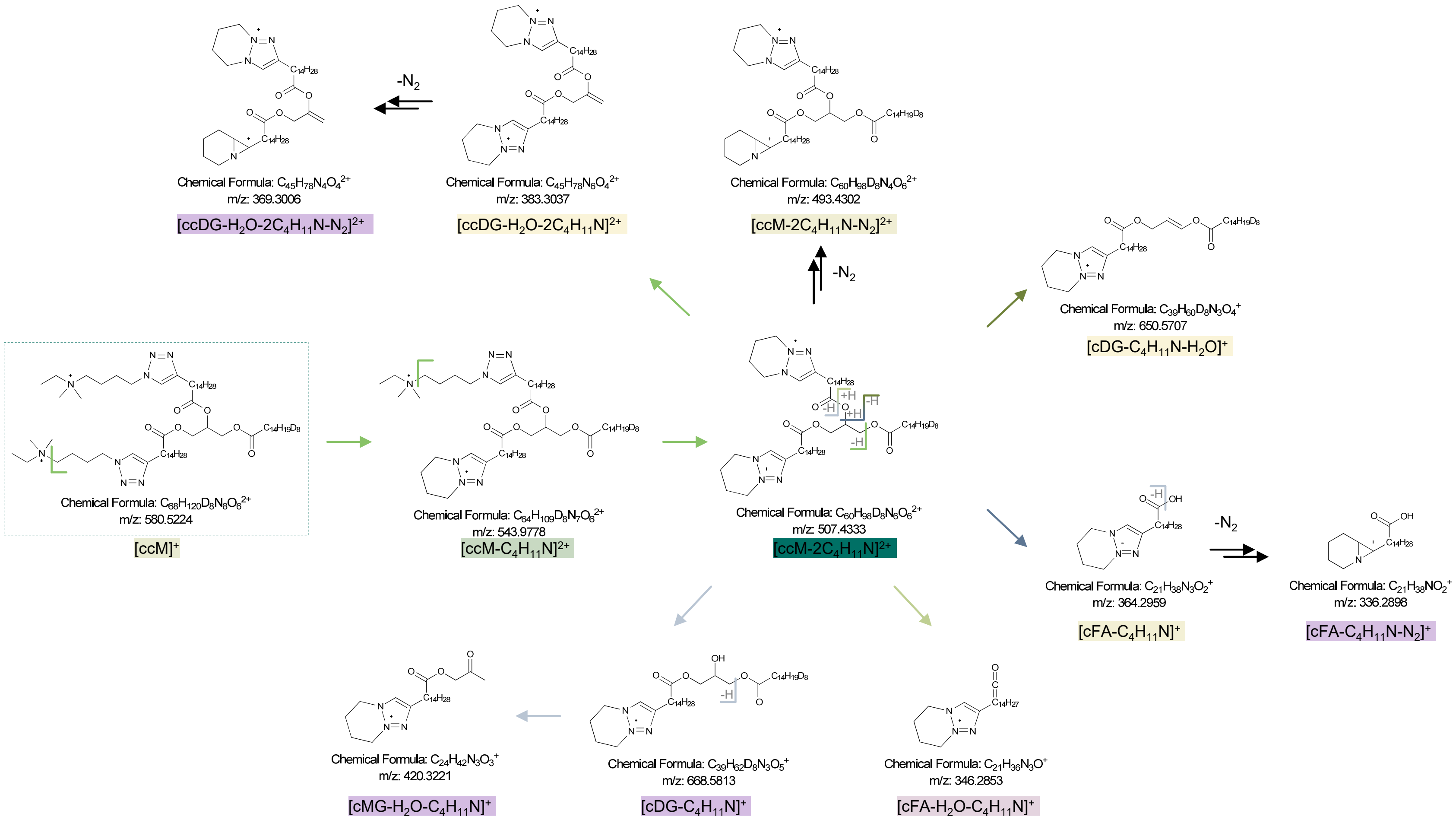

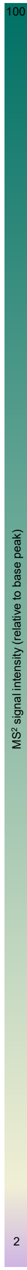

◂ Back to Content

TG-$^{13}C_3$ 19:1;C171_19:1;C171_19:1;C171 477.0759 – $C_{81}{}^{13}C_3H_{155}N_{12}O_6{}^{3+}$

Extracted Ion Chromatogram

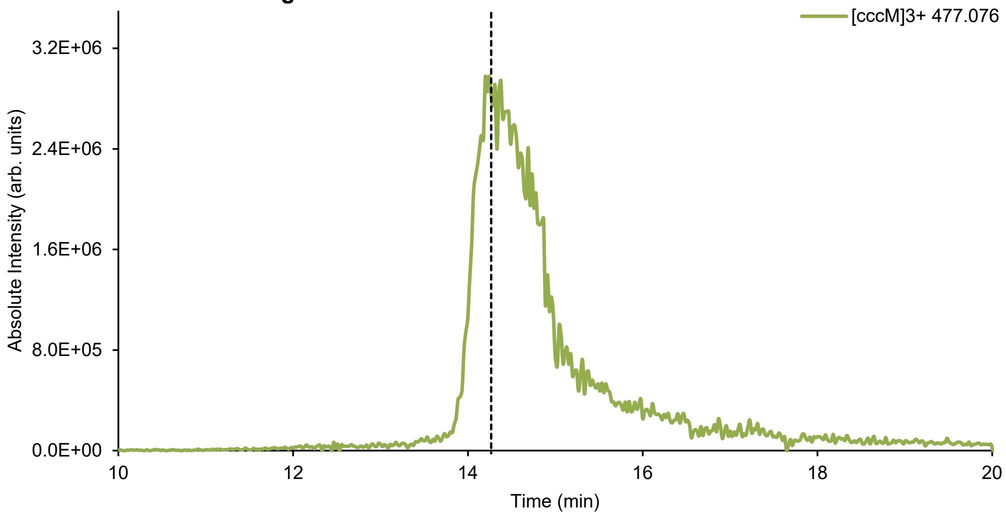

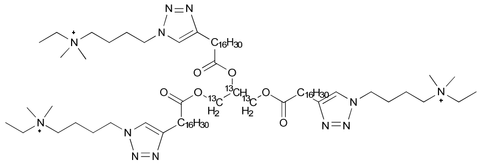

Chemical Formula: $C_{81}{}^{13}C_3H_{155}N_{12}O_6{}^{3+}$
m/z: 477.0759

[cccM]$^{3+}$

MS^2 [cccM]$^{3+}$

Ex_24_27_PN12 #9164 RT: 14.23 AV: 1 NL: 3.83E6
T: FTMS + p ESI d Full ms2 477.0758@hcd37.00 [148.1688-1481.6879]

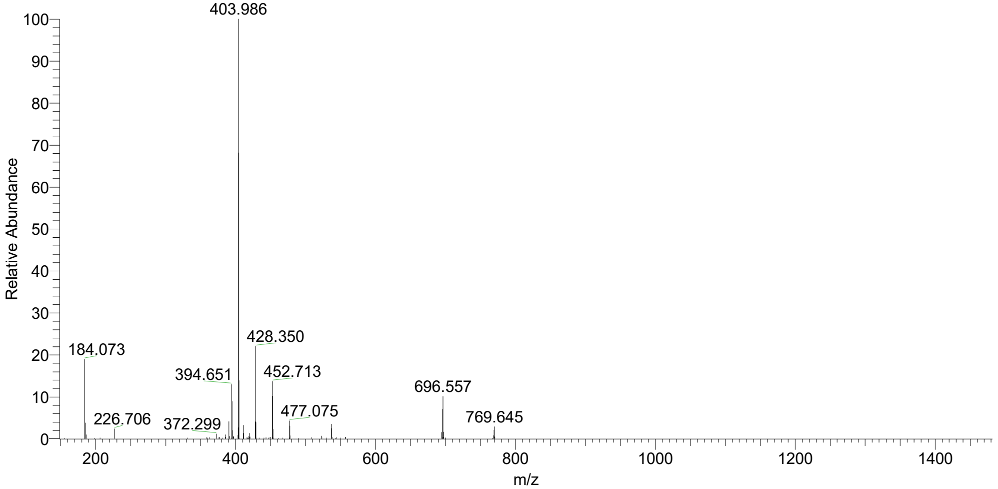

◂ Back to Content

TG-$^{13}C_3$ 19:1;C171_19:1;C171_19:1;C171 477.0759 – $C_{81}{}^{13}C_3H_{155}N_{12}O_6{}^{3+}$ proposed fragmentation scheme

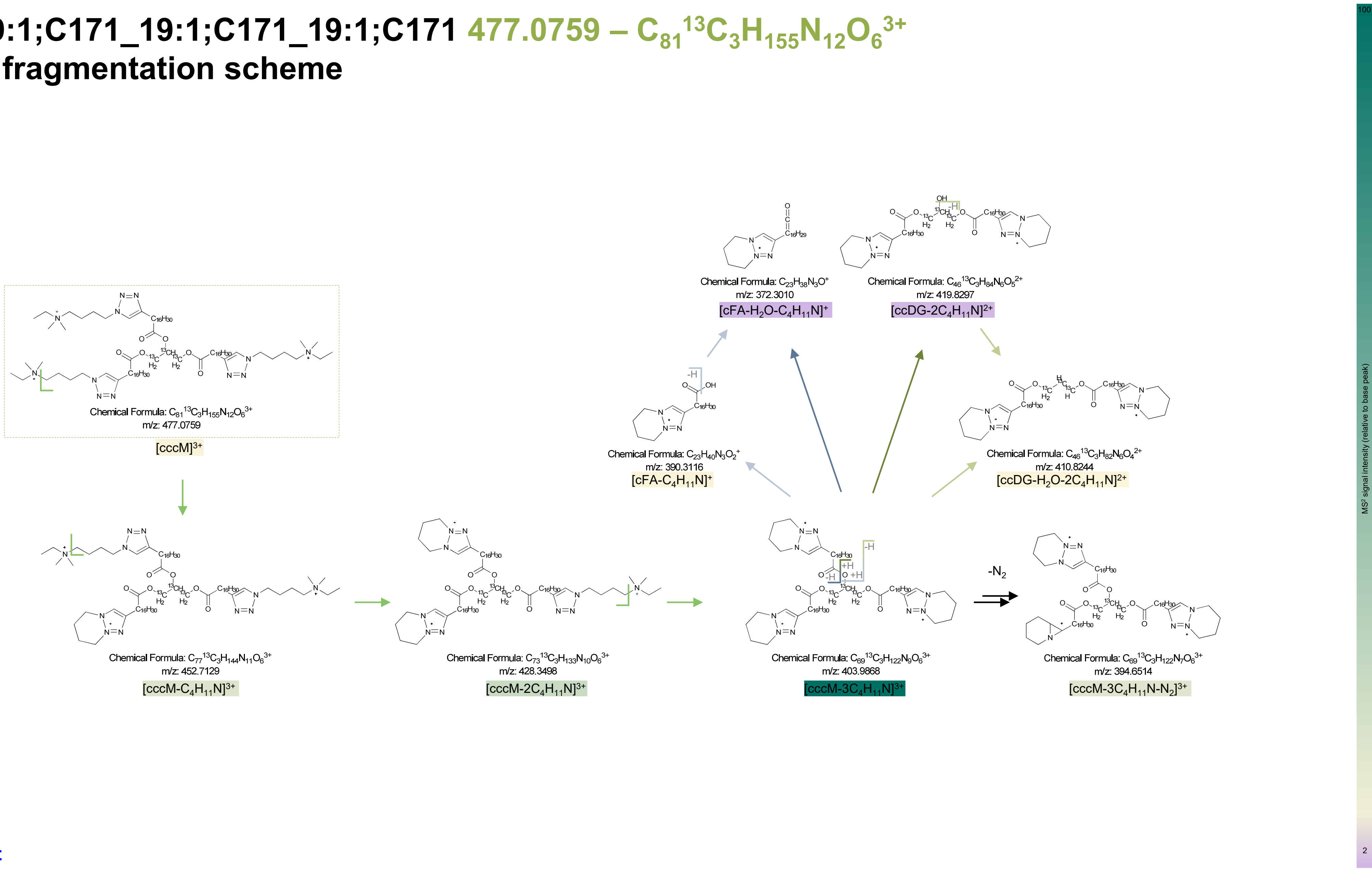

◂ Back to Content

CE-d7 17:0;C171 812.7733 – $C_{52}H_{86}D_7N_4O_2^+$

Extracted Ion Chromatogram

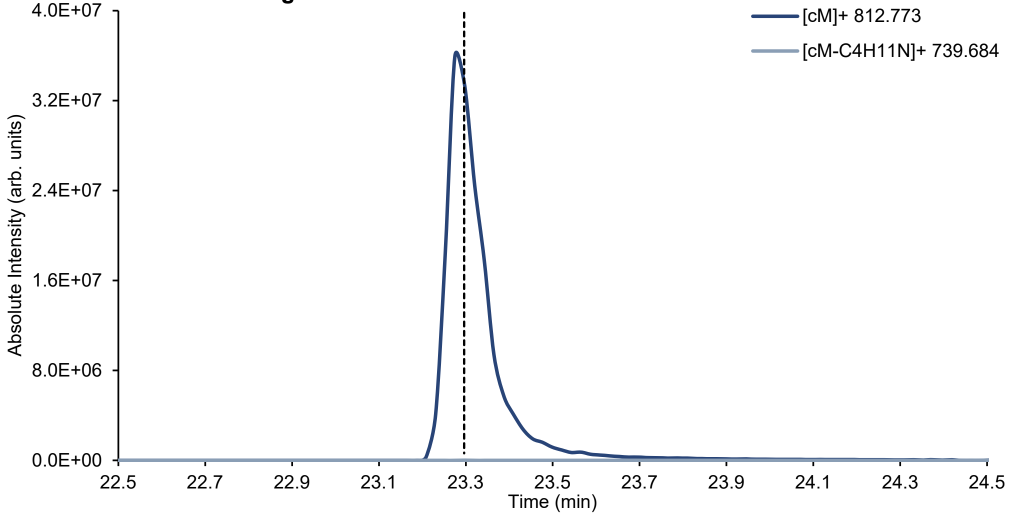

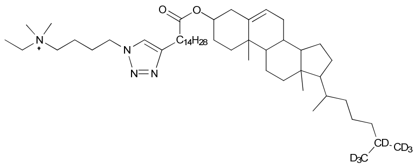

Chemical Formula: $C_{52}H_{86}D_7N_4O_2^+$
m/z: 812.7733
[cM]+

MS² [cM]⁺

Ex_24_25_PN06 #14847 RT: 23.30 AV: 1 NL: 2.16E7
T: FTMS + p ESI d Full ms2 812.7728@hcd40.00 [84.9640-849.6402]

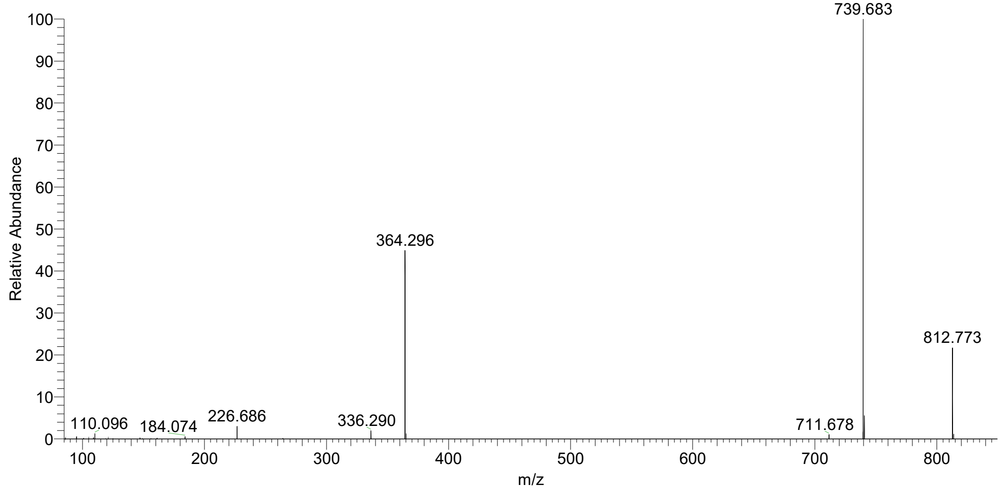

◂ Back to Content

CE-d$_7$ 17:0;C171 812.7733 – $C_{52}H_{86}D_7N_4O_2^+$ proposed fragmentation scheme

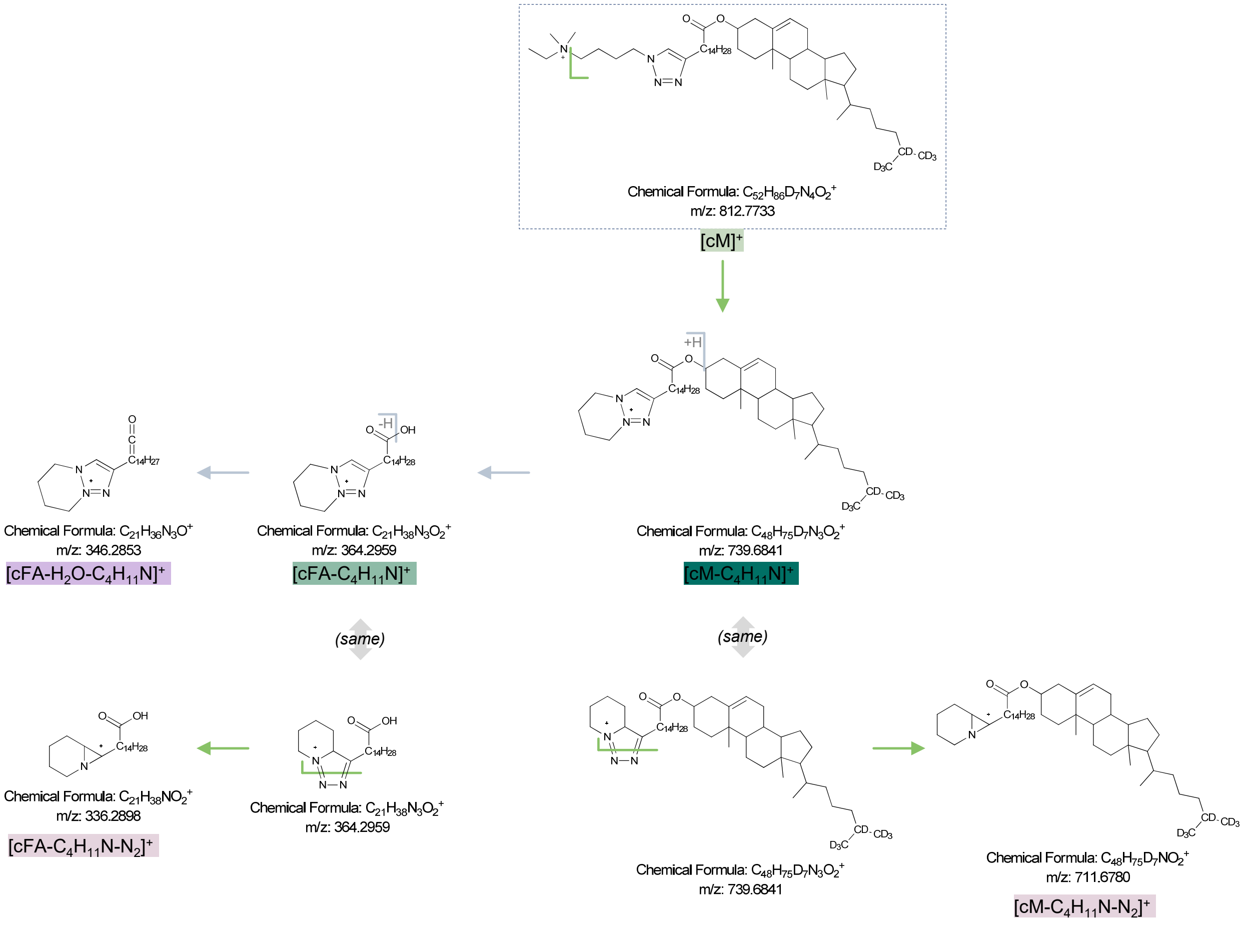

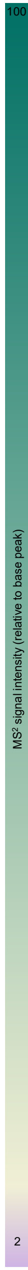

◂ Back to Content
