# Supplementary material for: LC‐MS and High‐Throughput Data Processing Solutions for Lipid Metabolic Tracing Using Bioorthogonal Click Chemistry
Source: Angew Chem Int Ed Engl. 2025 May 2;64(27):e202501884. doi: 10.1002/anie.202501884 (PMC12207371; doi:10.1002/anie.202501884)
Supplement: Supplementary file 2 — Supporting Information [file ANIE-64-e202501884-s001.zip › anie202501884-supp-0002-SuppMat/File S2.pdf]

File S2. LC-MS description of alkyne-containing lipids (one example per lipid class) formed endogenously, which were then derivatized by the C171 MS reporter.

For each species, chromatographic profile, MS/MS spectra of $[cM]^{+}$ and/or $[cM+H]^{2+}$ (one C171 unit per structure), $[ccM]^{2+}$ (two C171 units per structure), and putative gas-phase fragmentation schemes are shown.

Notes:

The sn position cannot be determined from MS^2 data, in most cases structures are drawn with a C171 chain in sn-1 only for convenience in drawing schemes.

All fragment ion structures are only conjectures.

The color-coding of fragment abundances in the proposed fragmentation schemes corresponds to the averaged signal intensities obtained by integrating "precursor → fragment" transitions in n = 10-20 raw files (see Supplementary Data Table 2 for complete lists of fragment ions and their relative abundances).

Content (hyperlink):

1. LPC;C171
2. PC;C171
3. PC O- C171@FOH
4. PC O- C171@FA
5. LPE;C171
6. PE;C171
7. PE P- C171@FOH
8. PE P- C171@FA
9. PI;C171
10. PS;C171
11. PG;C171
12. BMP;C171
13. CL;C171
14. dhCer C171@FA
15. Cer C171@FA
16. Cer C171@SPB
17. Cer 2C171@FA&SPB
18. HexCer C171@FA
19. HexCer C171@SPB
20. HexCer 2C171@FA&SPB
21. Hex2Cer C171@FA
22. Hex2Cer C171@SPB
23. Hex3Cer C171@FA
24. Hex3Cer C171@SPB
25. HexNAc-Hex3Cer C171@FA
26. HexNAc-Hex3Cer C171@SPB
27. SM C171@FA
28. SM C171@SPB
29. FA;C171
30. MG;C171
31. DG;C171
32. TG;C171
33. CE;C171

LPC 18:1;C171 688.4773 – $C_{34}H_{67}N_5O_7P^{+}$ / 344.7423 – $C_{34}H_{68}N_5O_7P^{2+}$

Extracted Ion Chromatogram

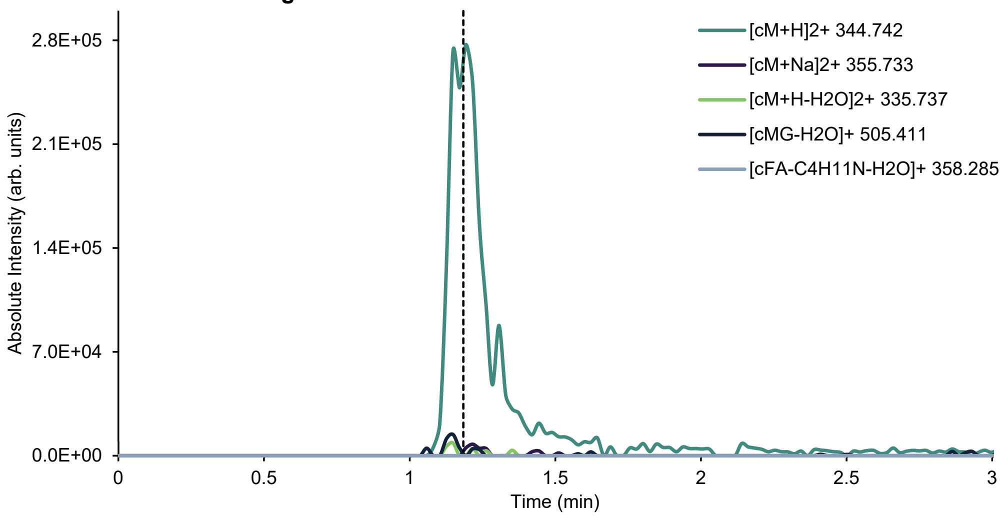

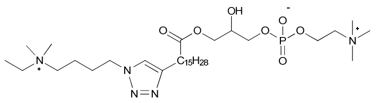

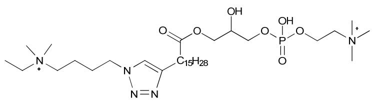

MS² [cM+H]²⁺

Ex_23_50_PN01 #750 RT: 1.20 AV: 1 NL: 8.45E4
T: FTMS + p ESI d Full ms2 344.7420@hcd35.00 [72.4498-724.4977]

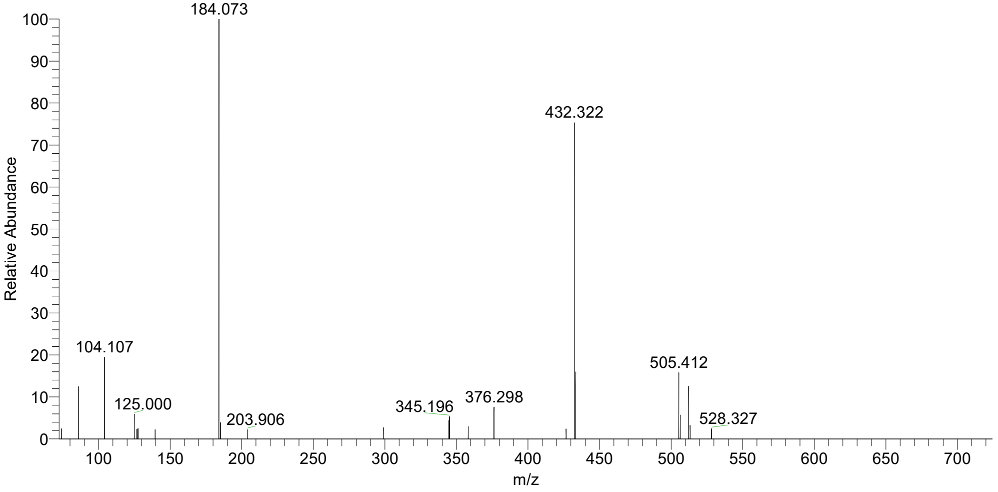

◂ Back to Content

LPC 18:1;C171 344.7423 – $C_{34}H_{68}N_5O_7P^{2+}$ proposed fragmentation scheme

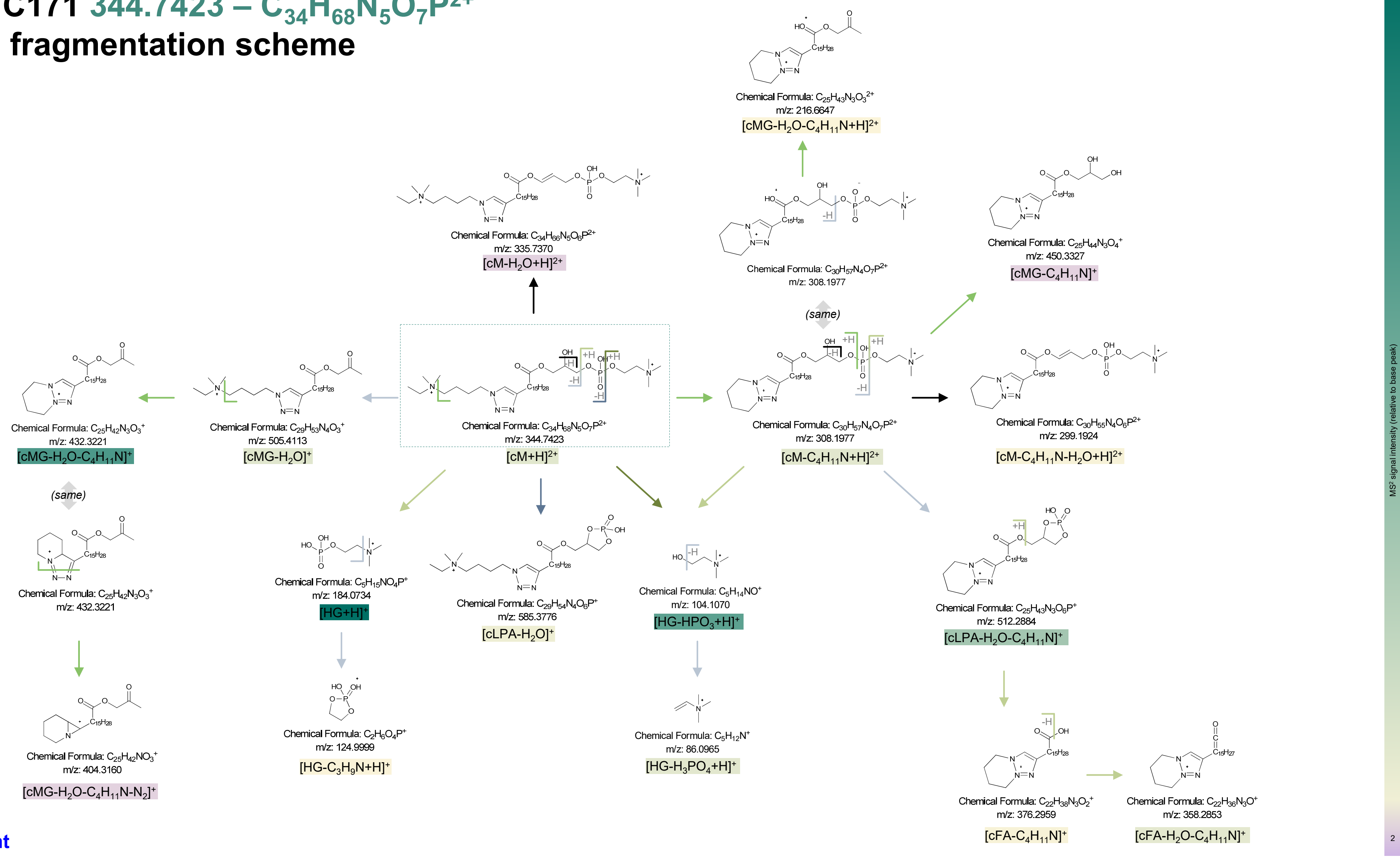

◂ Back to Content

PC 18:1;C171_18:0 954.7382 – $C_{52}H_{101}N_5O_8P^{+}$ / 477.8728 – $C_{52}H_{102}N_5O_8P^{2+}$

Extracted Ion Chromatogram

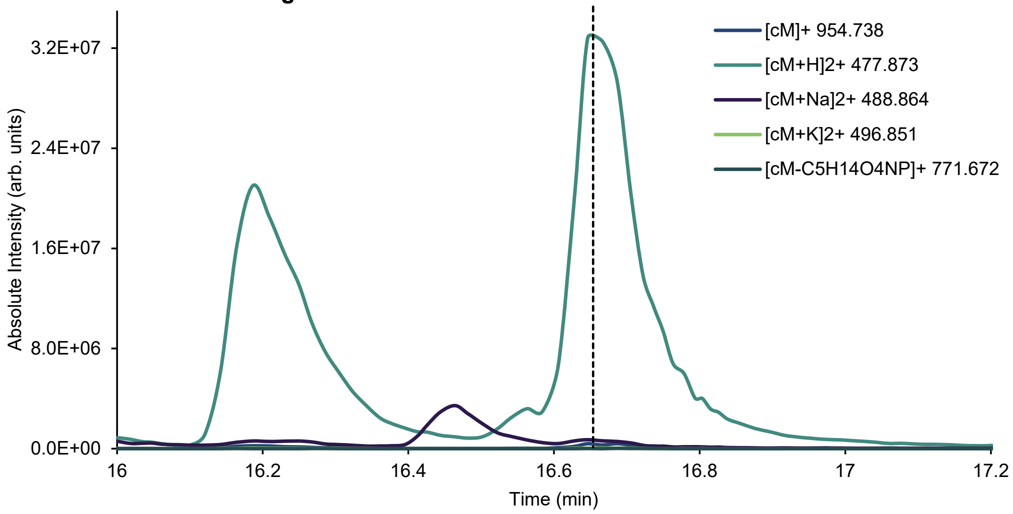

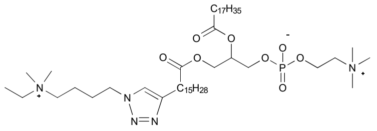

Chemical Formula: $C_{52}H_{101}N_5O_8P^{+}$
m/z: 954.7383
$[cM]^{+}$

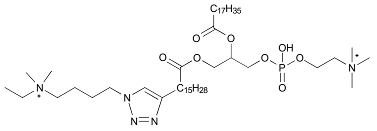

Chemical Formula: $C_{52}H_{102}N_5O_8P^{2+}$
m/z: 477.8728
$[cM+H]^{2+}$

MS^2 $[cM]^{+}$

25_35_60 #5313 RT: 16.67 AV: 1 NL: 1.83E5
T: FTMS + p ESI d Full ms2 954.7373@hcd40.00 [99.4444-994.4440]

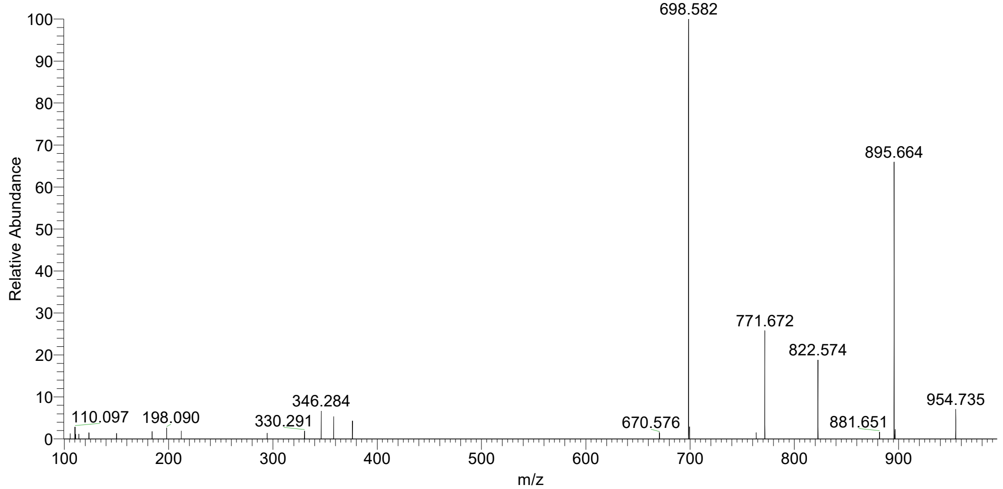

MS^2 $[cM+H]^{2+}$

25_35_60 #5319 RT: 16.68 AV: 1 NL: 2.61E7
T: FTMS + p ESI d Full ms2 477.8721@hcd40.00 [99.6083-996.0832]

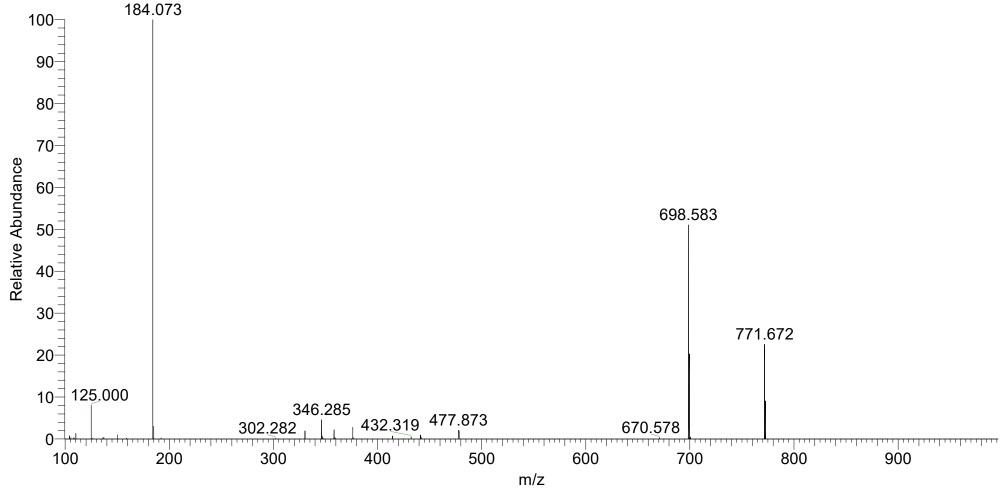

◂ Back to Content

PC 18:1;C171_18:0 954.7382 – $C_{52}H_{101}N_5O_8P^+$
proposed fragmentation scheme

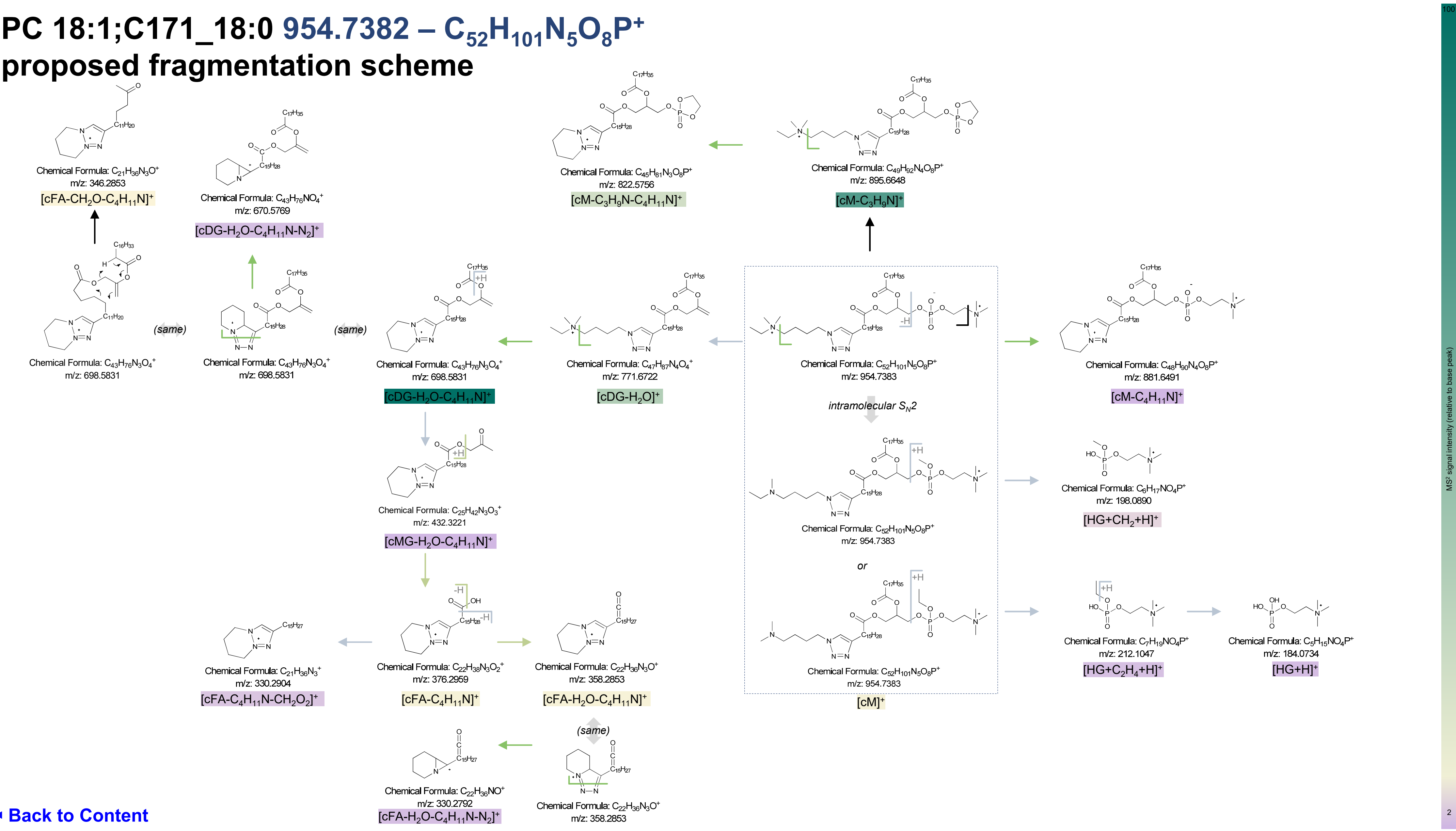

◂ Back to Content

PC 18:1;C171_18:0 477.8728 – $C_{52}H_{102}N_5O_8P^{2+}$ proposed fragmentation scheme

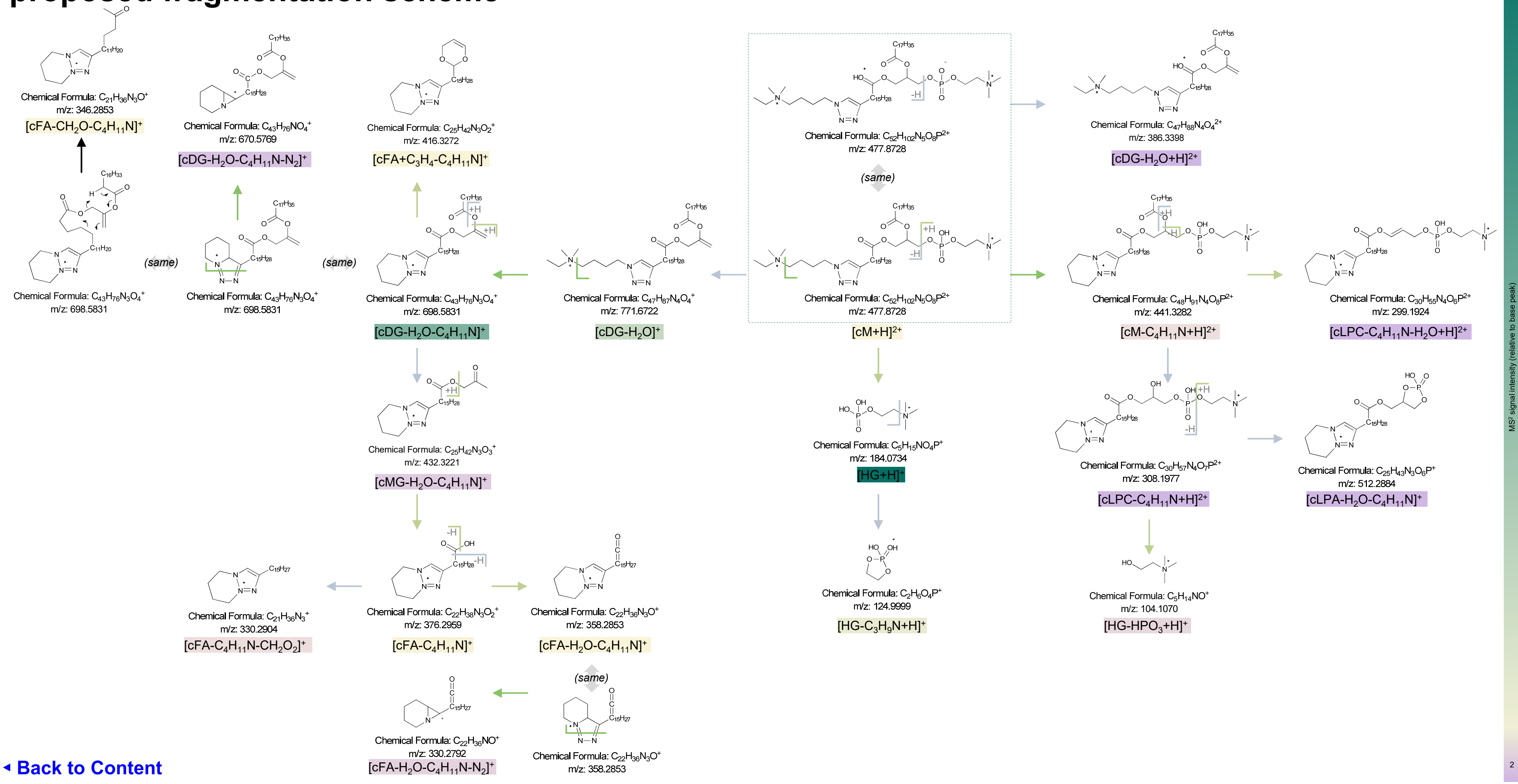

◂ Back to Content

PC O-16:0;C171_16:0 886.7120 – $C_{48}H_{97}N_5O_7P^{+}$ / 443.8600 – $C_{48}H_{98}N_5O_7P^{2+}$

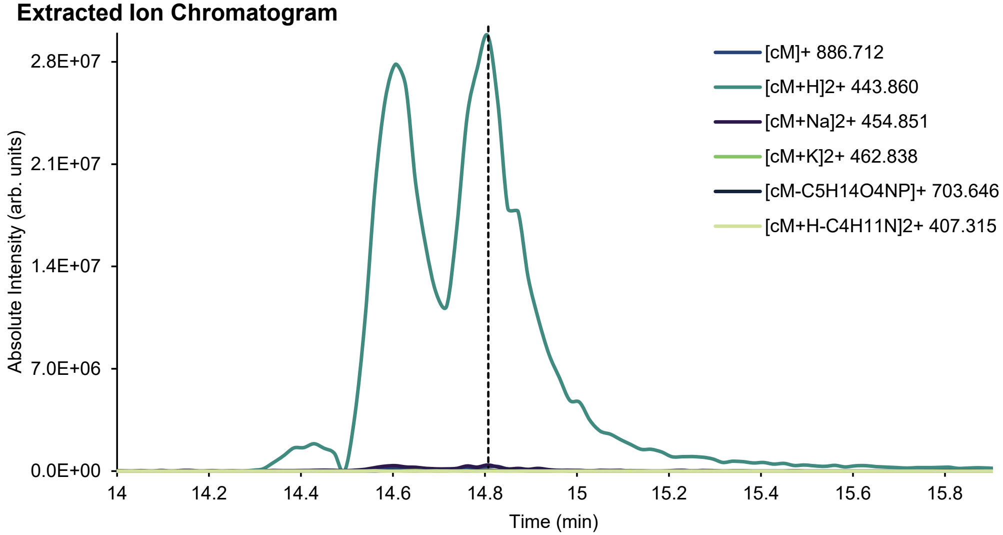

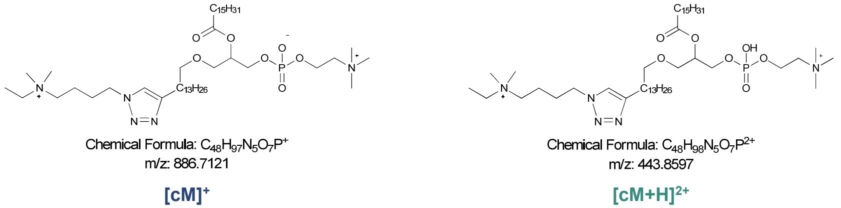

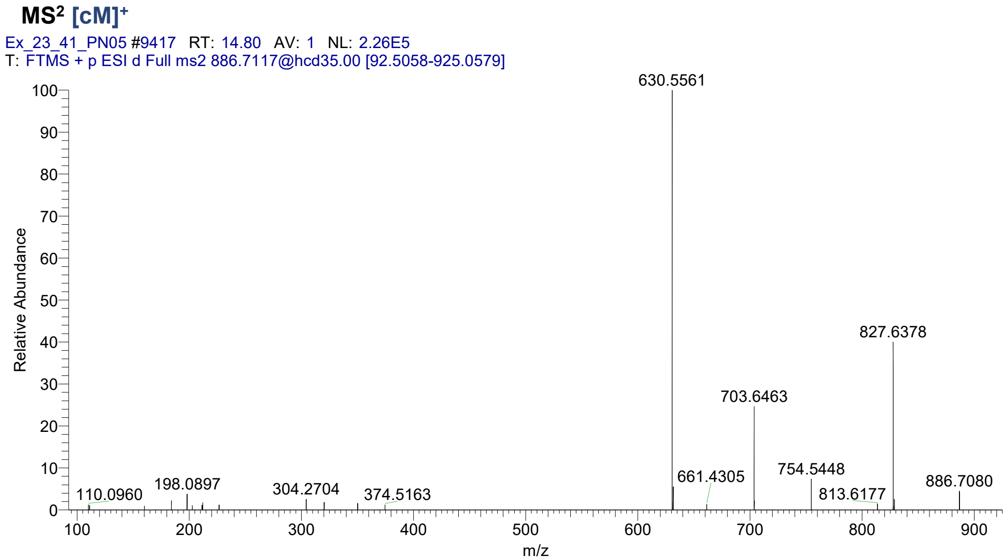

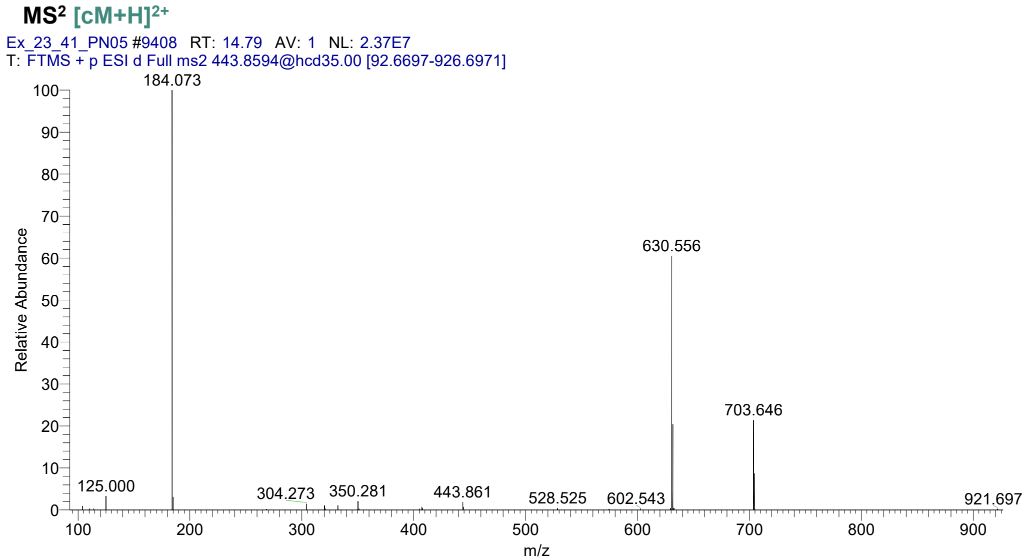

◂ Back to Content

PC O-16:0;C171_16:0 886.7120 – $C_{48}H_{97}N_5O_7P^+$ proposed fragmentation scheme

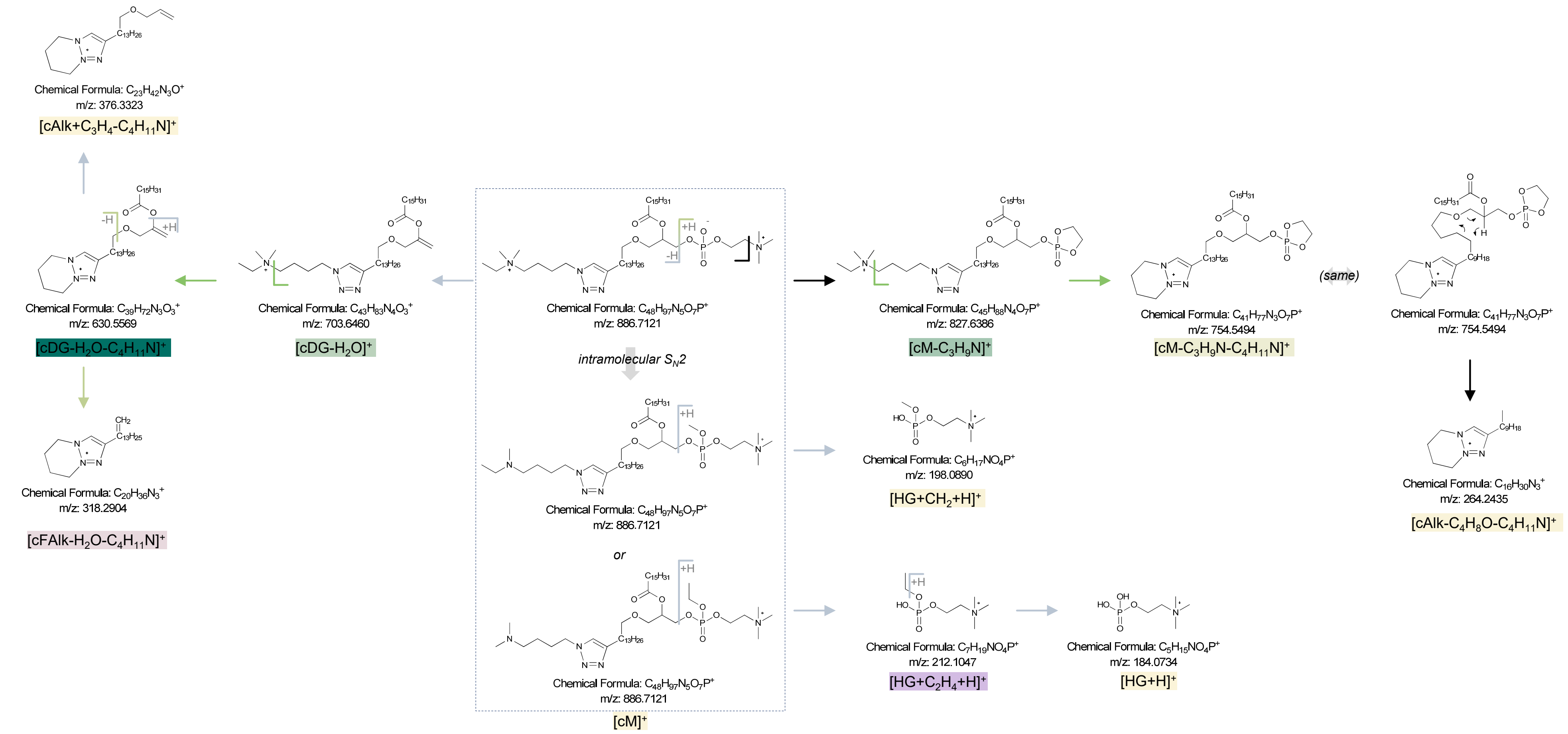

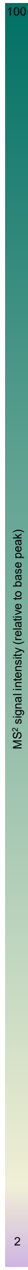

Also like [cFAlk-H2O-C4H11N+FA-CH2O2]+, $C_{25}H_{66}N_3$ m/z 528.5251

◂ Back to Content

PC O-16:0;C171_16:0 443.8600 – $C_{48}H_{98}N_5O_7P^{2+}$
proposed fragmentation scheme

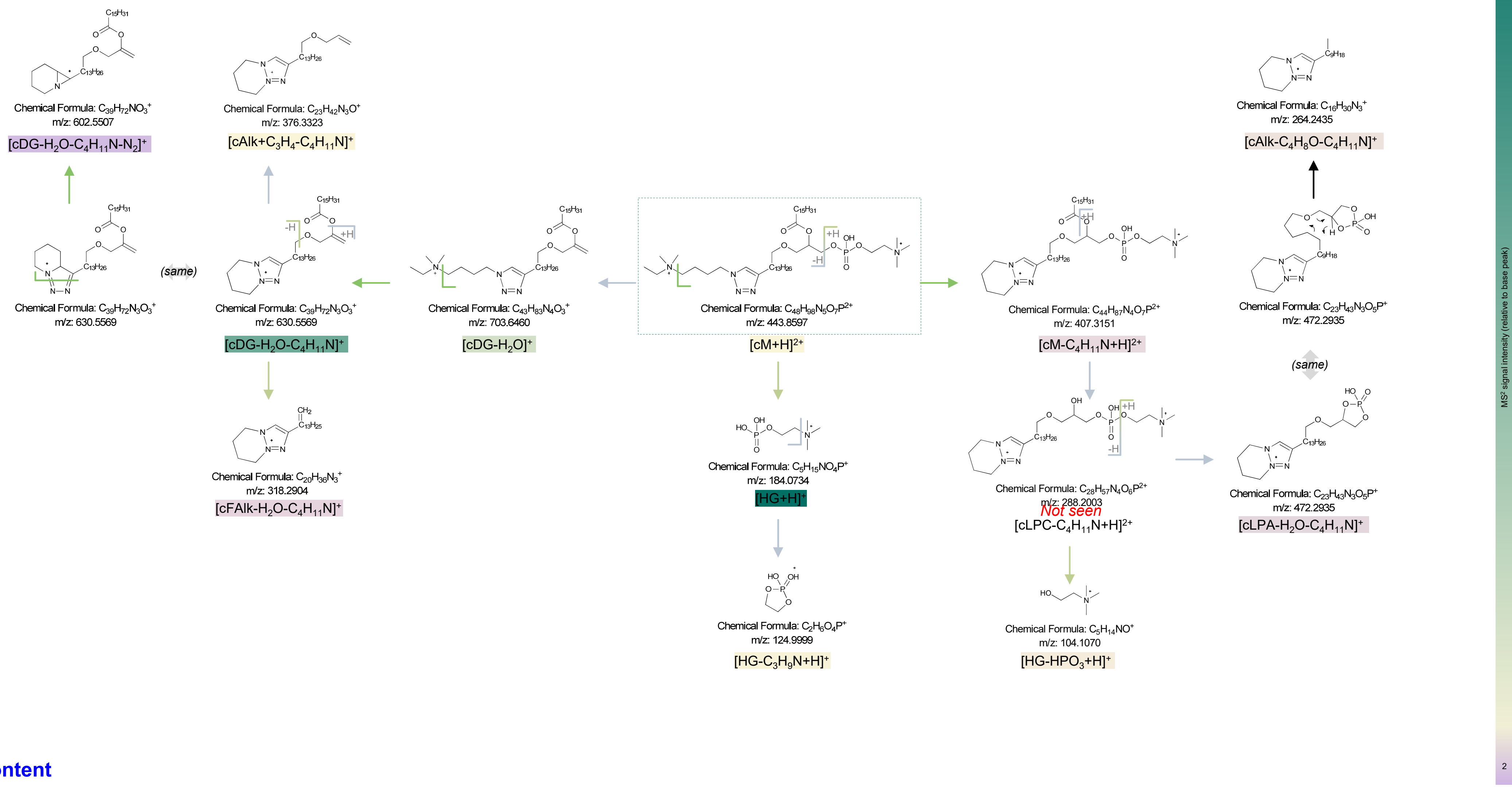

◂ Back to Content

PC 18:1;C171_O-16:0 912.7277 – $C_{50}H_{99}N_5O_7P^+$ / 456.8675 – $C_{50}H_{100}N_5O_7P^{2+}$

Extracted Ion Chromatogram

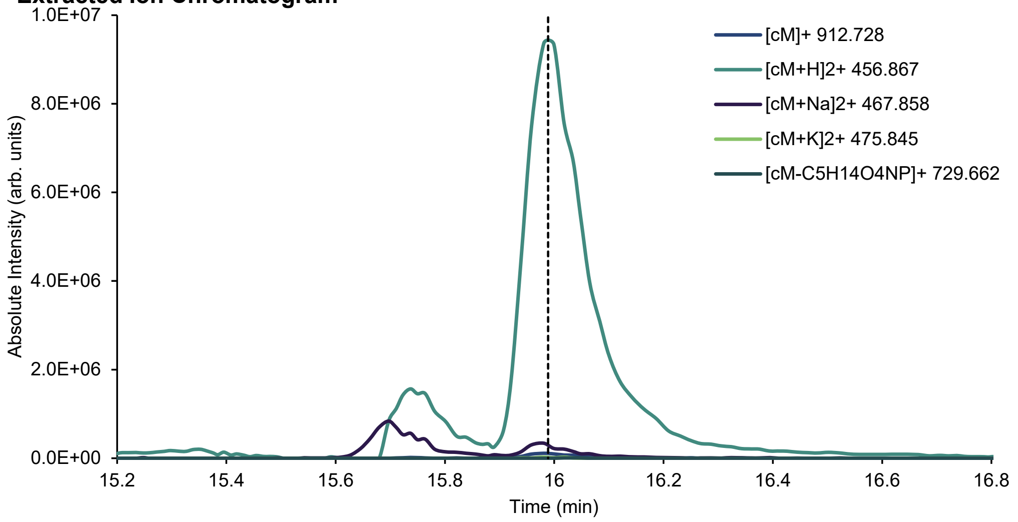

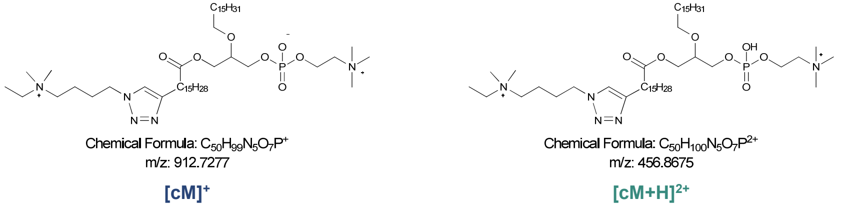

MS² [cM]⁺

25_35_60 #4837 RT: 15.97 AV: 1 NL: 6.80E4
T: FTMS + p ESI d Full ms2 912.7266@hcd40.00 [95.1593-951.5931]

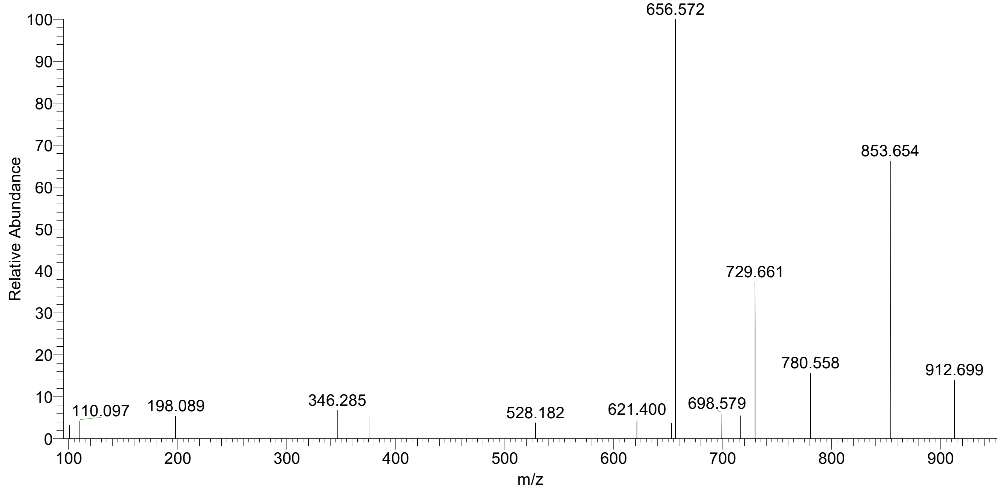

MS² [cM+H]²⁺

25_40_45 #4853 RT: 15.97 AV: 1 NL: 7.80E6
T: FTMS + p ESI d Full ms2 456.8670@hcd36.67 [95.3233-953.2327]

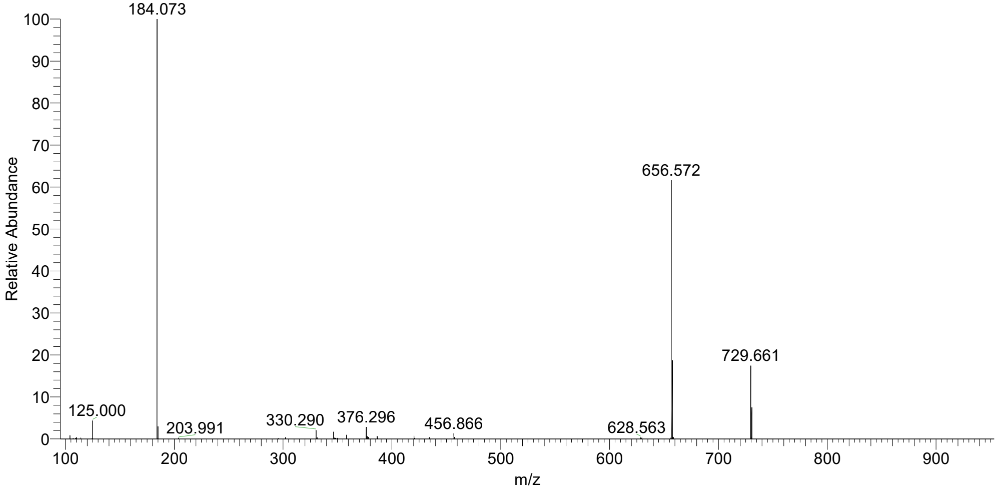

◂ Back to Content

PC 18:1;C171_O-16:0 912.7277 – $C_{50}H_{99}N_5O_7P^+$ proposed fragmentation scheme

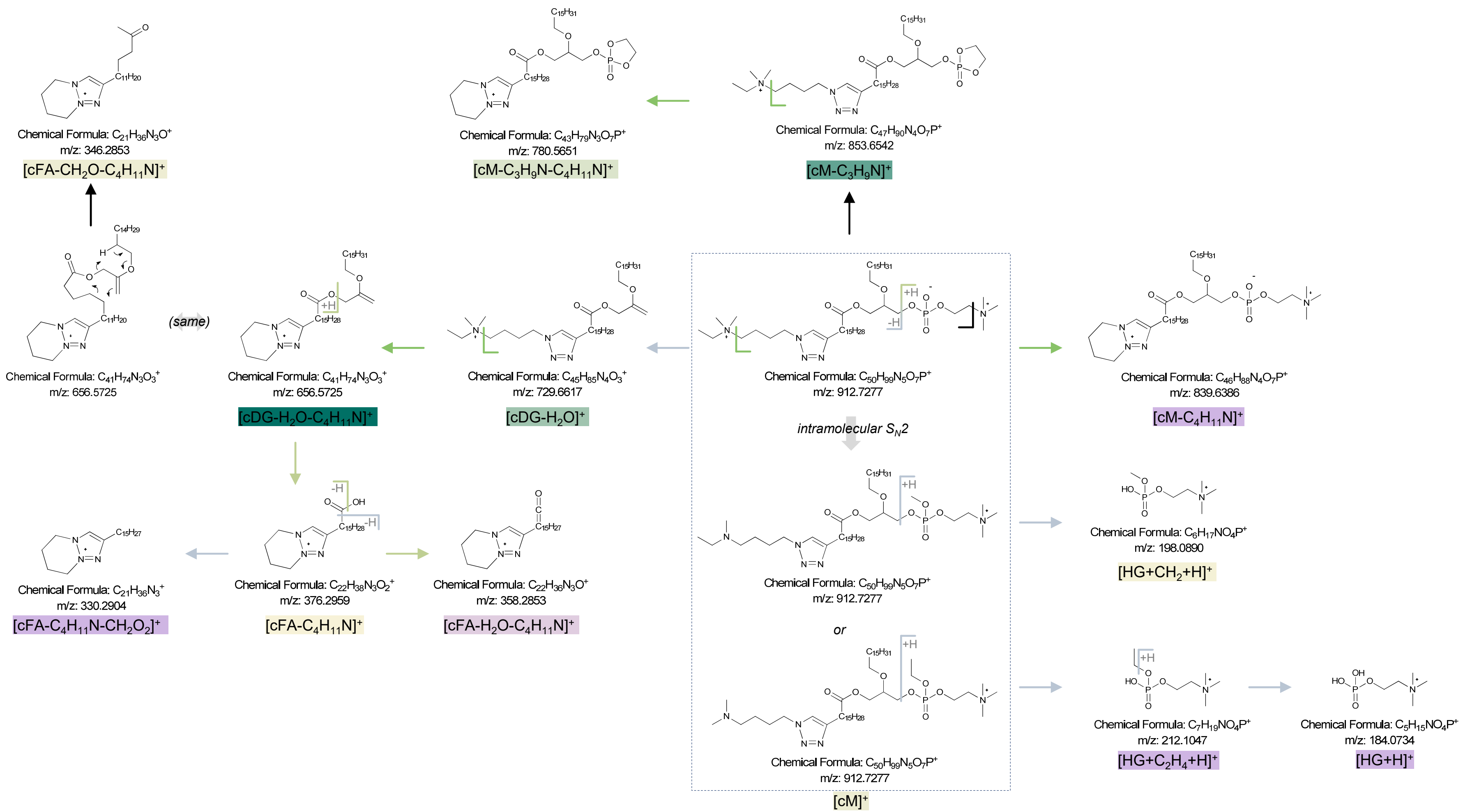

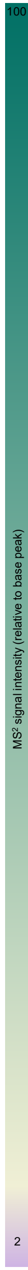

◂ Back to Content

PC 18:1;C171_O-16:0 456.8675 – $C_{50}H_{100}N_5O_7P^{2+}$ proposed fragmentation scheme

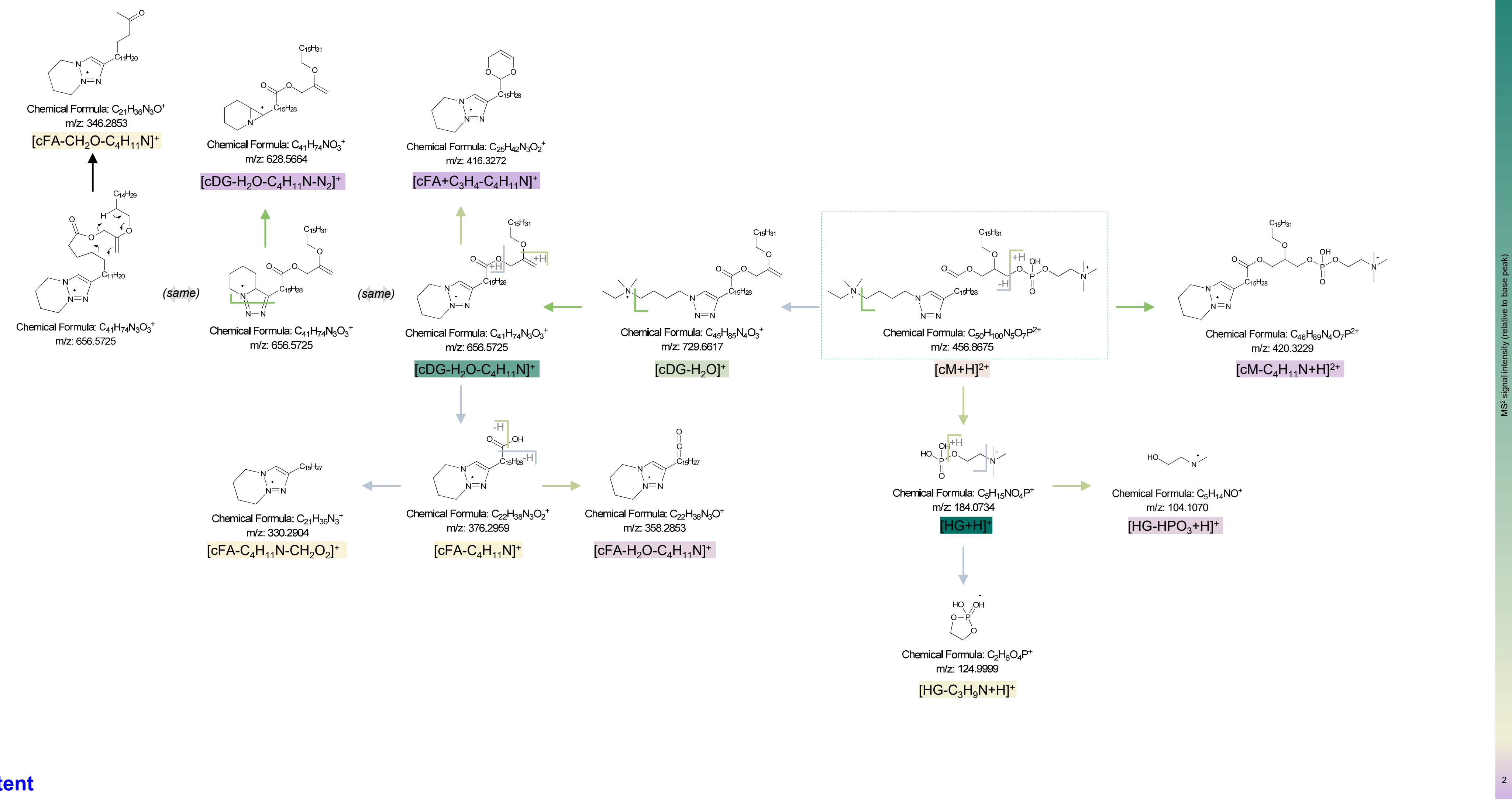

◂ Back to Content

LPE 18:1;C171 646.4304 – $C_{31}H_{61}N_5O_7P^+$ / 323.7186 – $C_{31}H_{62}N_5O_7P^{2+}$

Extracted Ion Chromatogram

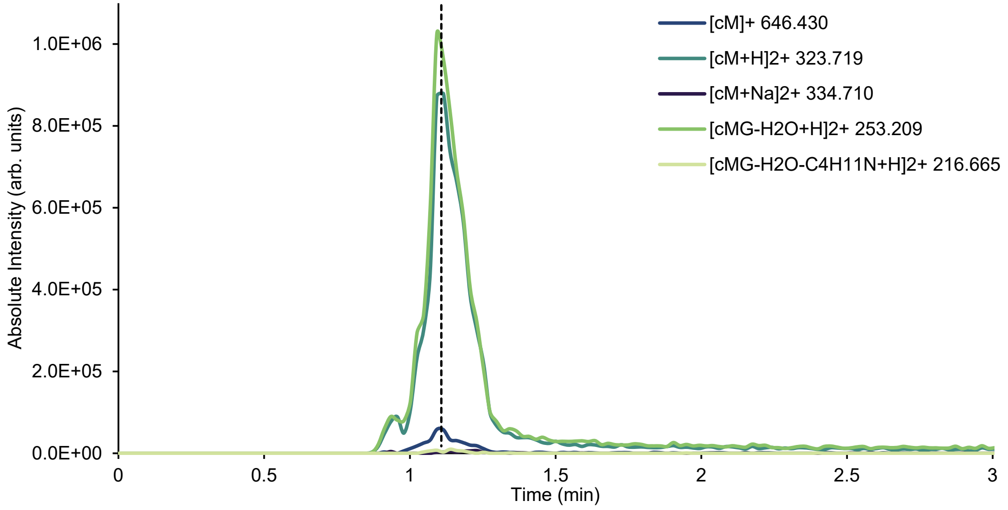

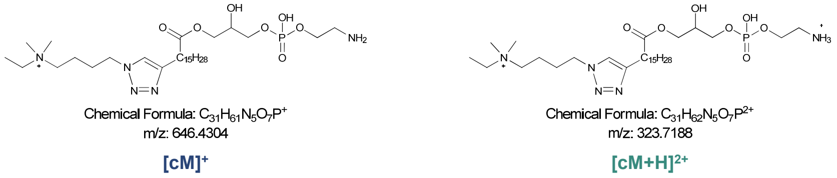

MS² [cM]⁺

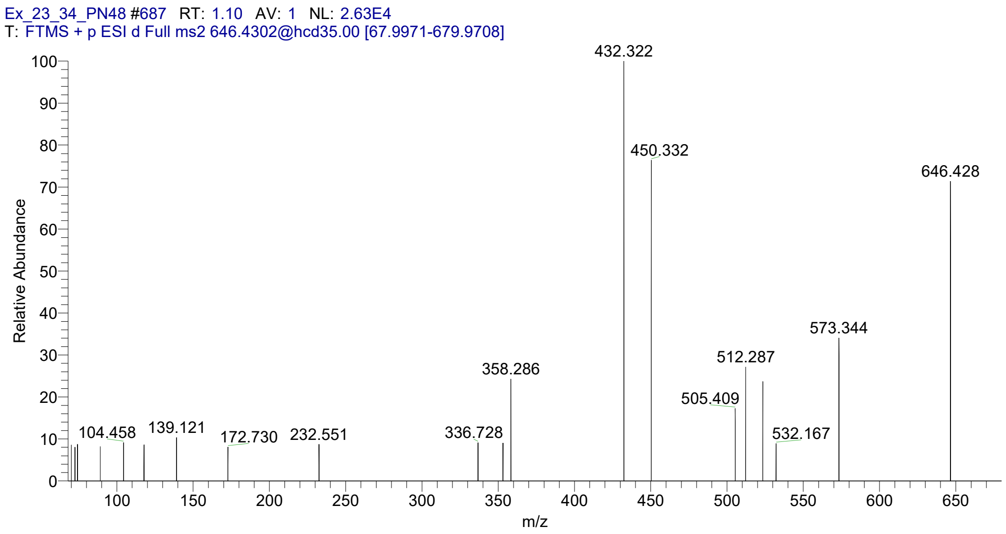

MS² [cM+H]²⁺

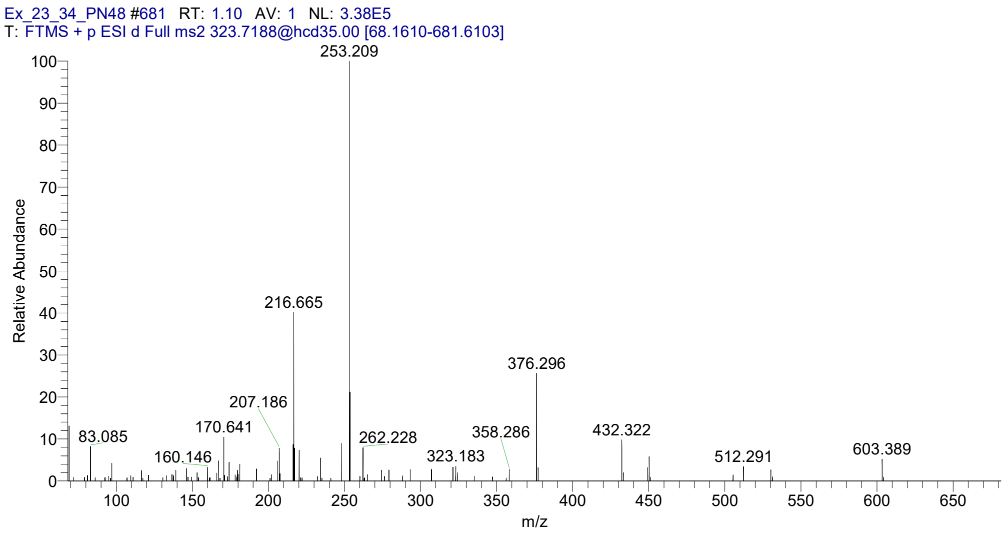

◂ Back to Content

LPE 18:1;C171 646.4304 – $C_{31}H_{61}N_5O_7P^+$
proposed fragmentation scheme

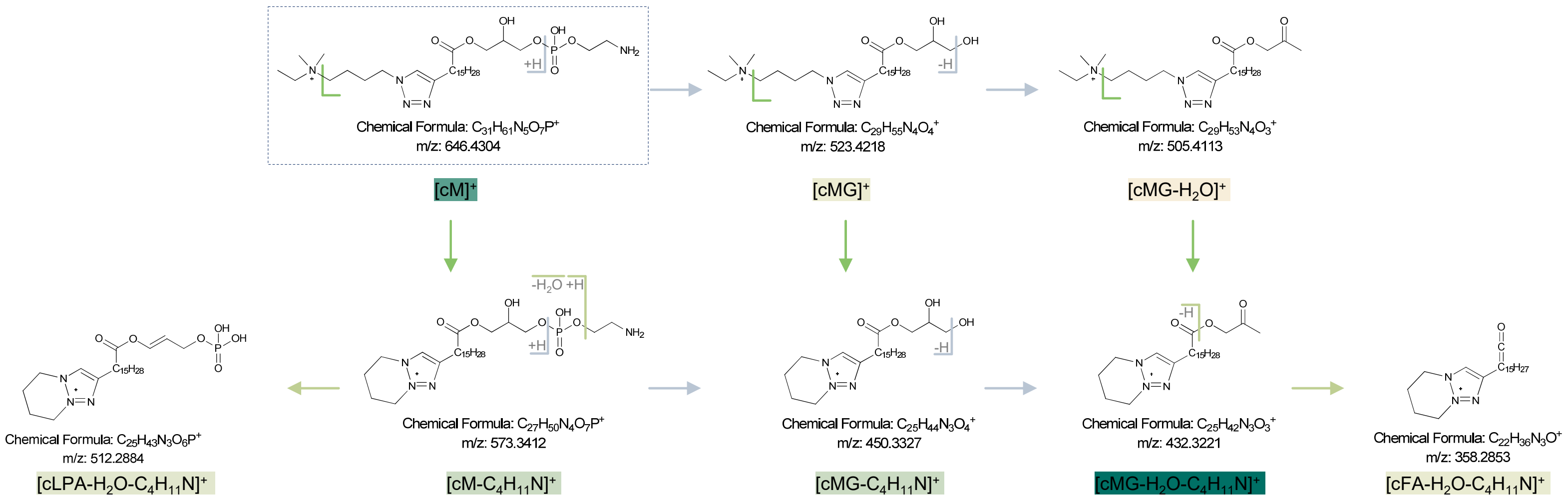

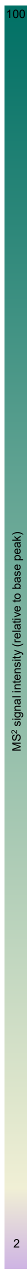

◂ Back to Content

LPE 18:1;C171 323.7186 – $C_{31}H_{62}N_5O_7P^{2+}$
proposed fragmentation scheme

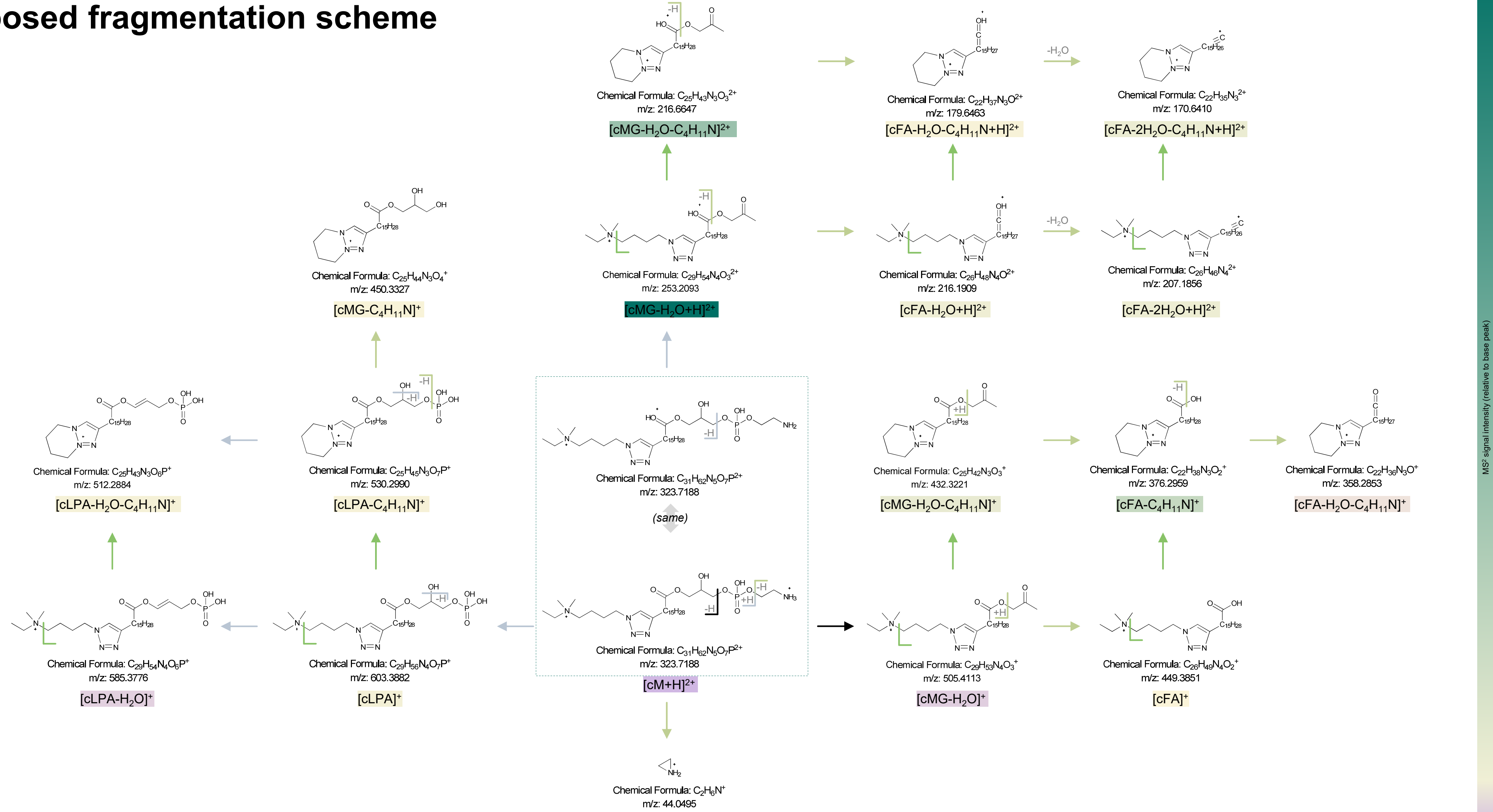

◂ Back to Content

PE 18:1;C171_18:0 912.6913 – $C_{49}H_{95}N_5O_8P^+$ / 456.8492 – $C_{49}H_{96}N_5O_8P^{2+}$

Extracted Ion Chromatogram

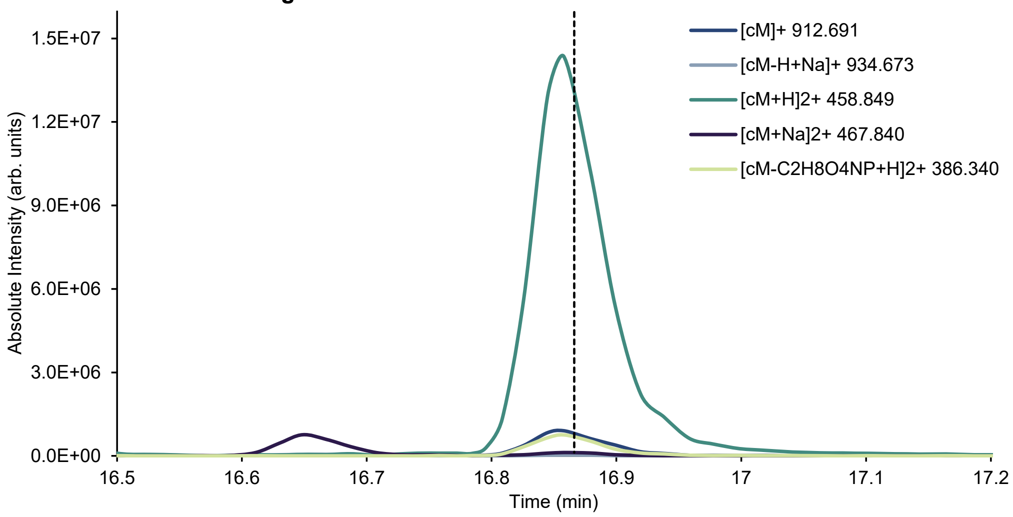

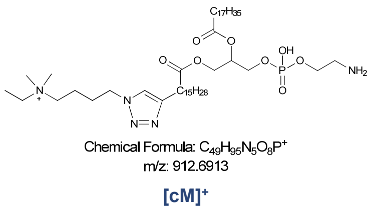

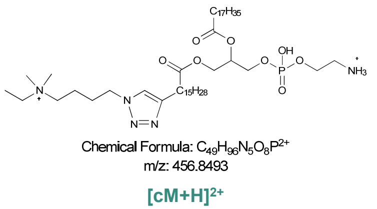

MS² [cM]⁺

30_40_50 #5471 RT: 16.86 AV: 1 NL: 3.42E5
T: FTMS + p ESI d Full ms2 912.6906@hcd40.00 [95.1556-951.5565]

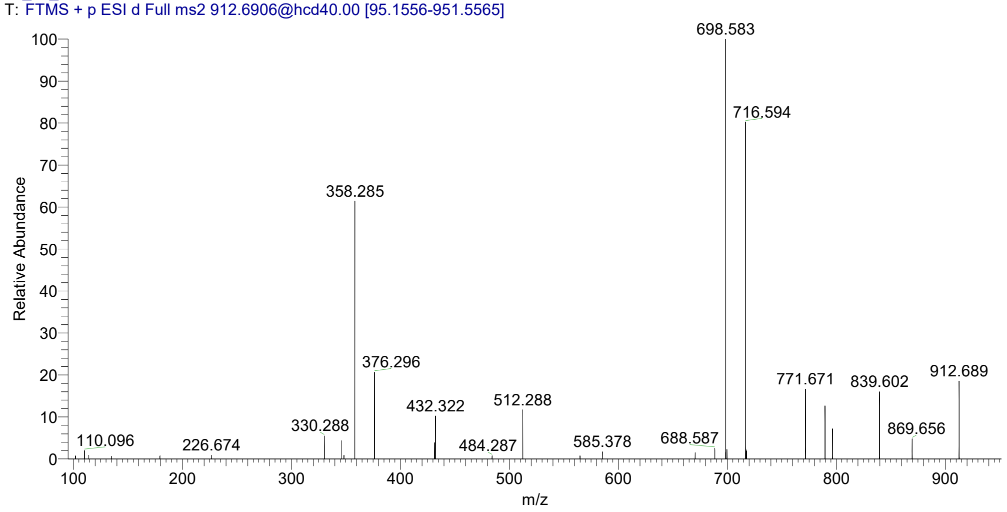

MS² [cM+H]²⁺

25_35_45 #5441 RT: 16.86 AV: 1 NL: 4.20E6
T: FTMS + p ESI d Full ms2 456.8486@hcd35.00 [95.3195-953.1952]

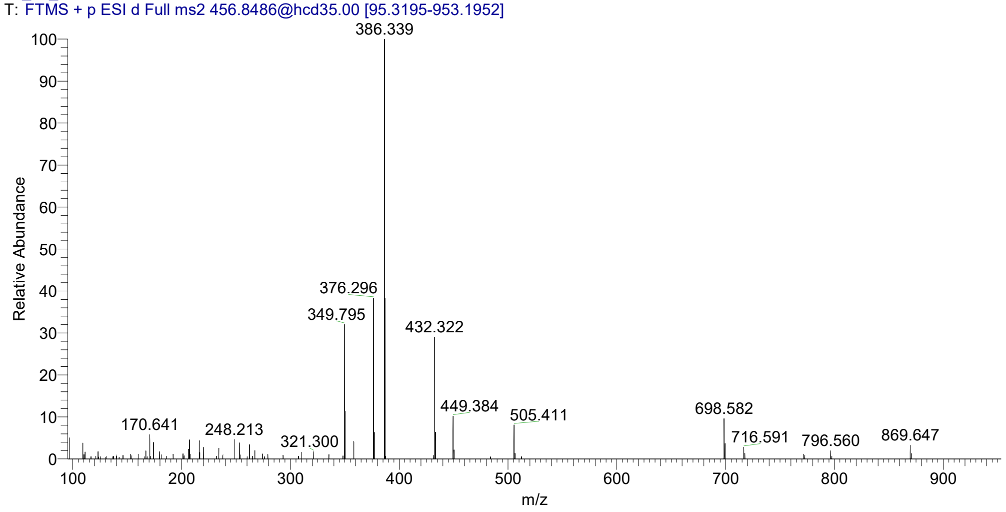

◂ Back to Content

PE 18:1;C171_18:0 912.6913 – $C_{49}H_{95}N_5O_8P^+$
proposed fragmentation scheme

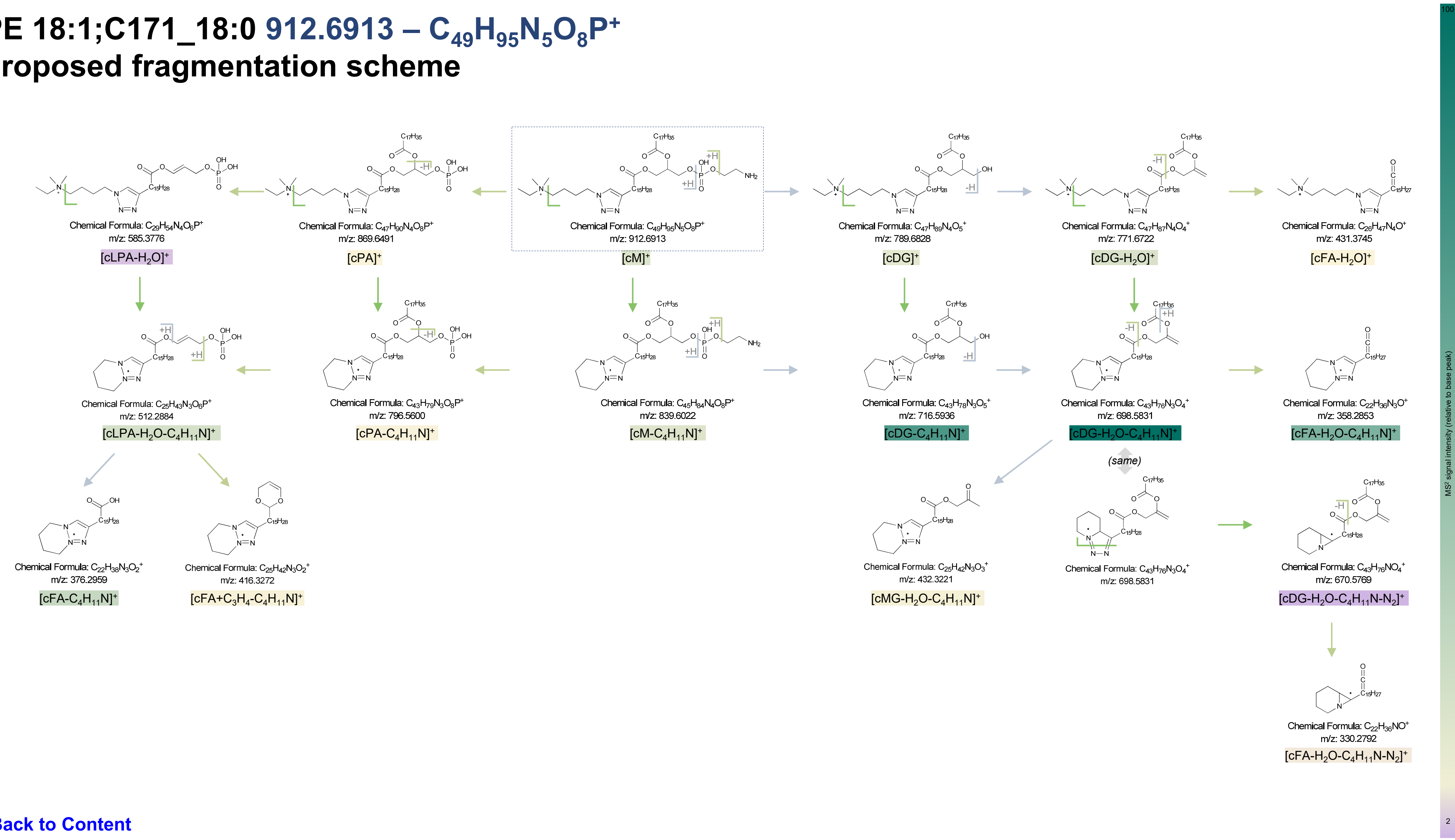

◂ Back to Content

PE 18:1;C171_18:0 456.8492 – $C_{49}H_{96}N_5O_8P^{2+}$ proposed fragmentation scheme

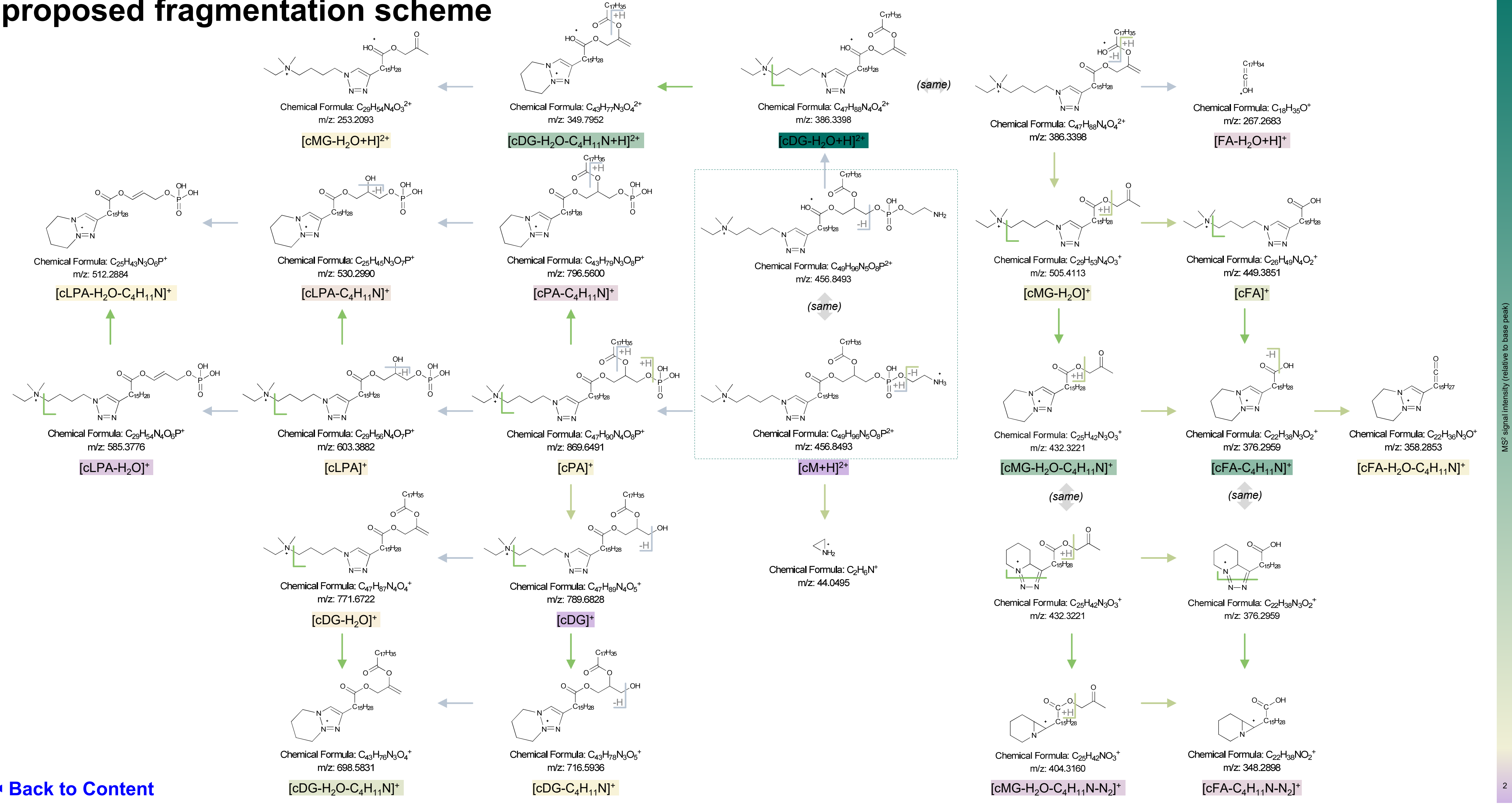

◂ Back to Content

PE P-16:0;C171_22:4 918.6808 – $C_{51}H_{93}N_5O_7P^+$ / 459.8440 – $C_{51}H_{94}N_5O_7P^{2+}$

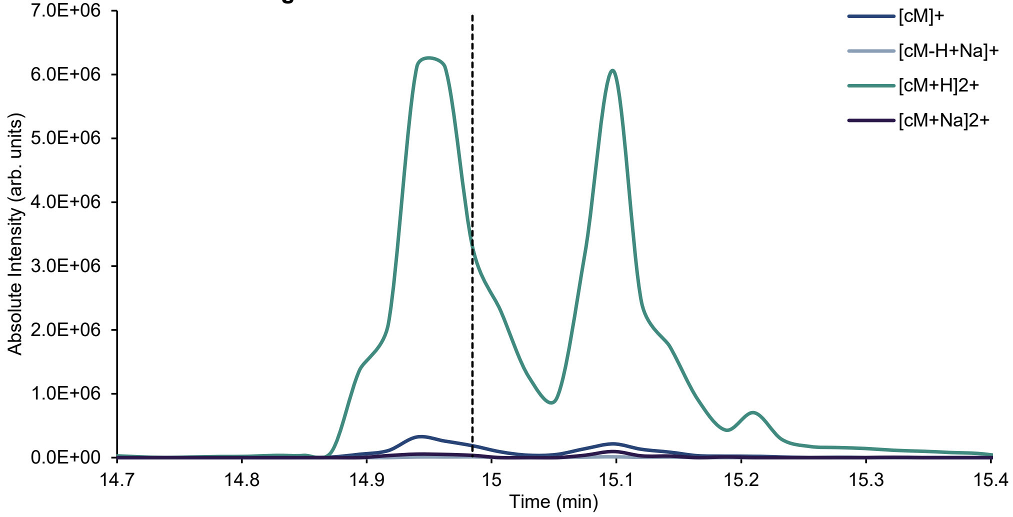

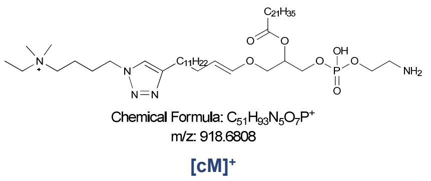

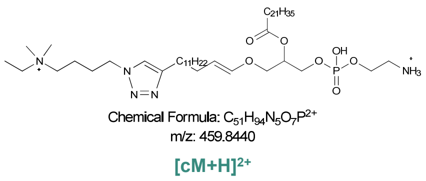

MS² [cM]⁺

Ex_23_34_PN23 #9536 RT: 14.95 AV: 1 NL: 1.37E5
T: FTMS + p ESI d Full ms2 918.6806@hcd35.00 [95.7666-957.6662]

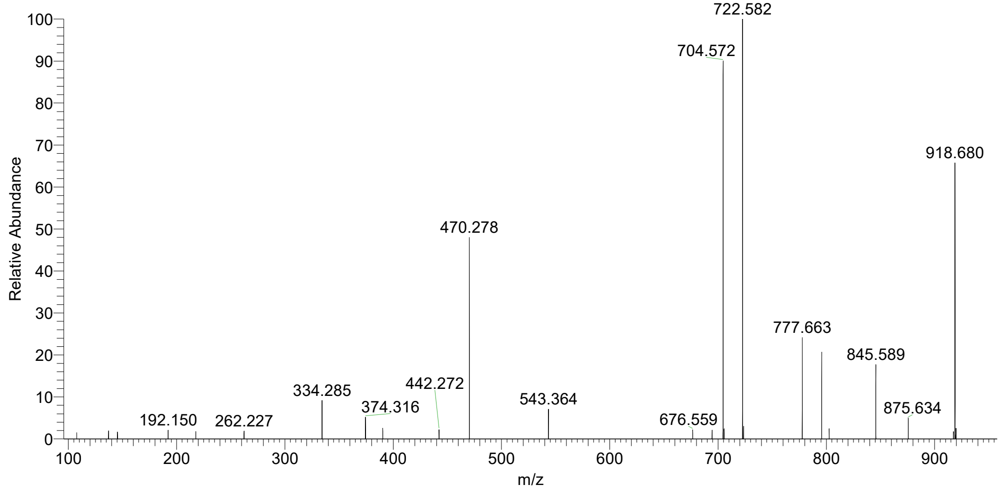

MS² [cM+H]²⁺

Ex_23_34_PN23 #9546 RT: 14.97 AV: 1 NL: 2.27E5
T: FTMS + p ESI d Full ms2 459.8435@hcd35.00 [95.9305-959.3047]

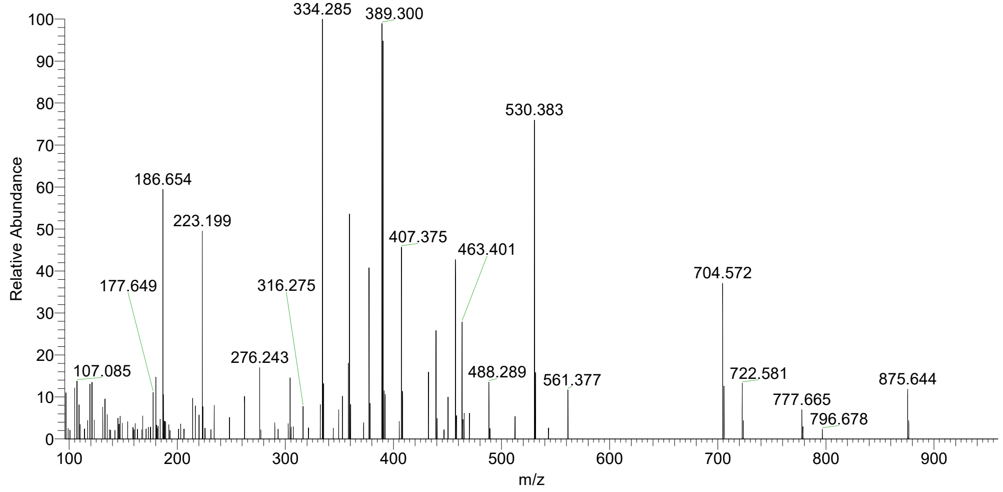

◂ Back to Content

PE P-16:0;C171_22:4 818.6808 – $C_{51}H_{93}N_5O_7P^+$
proposed fragmentation scheme

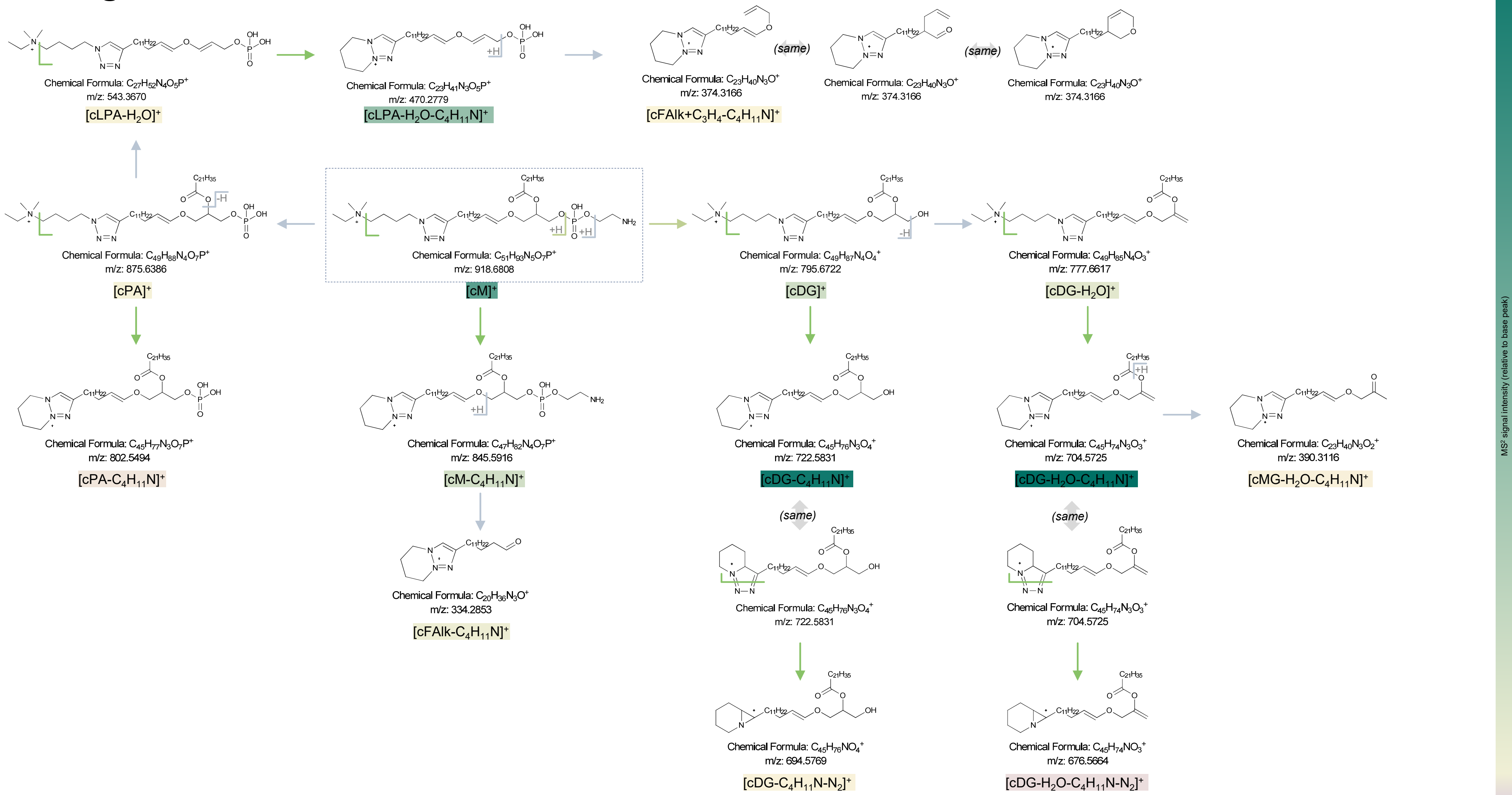

◂ Back to Content

PE P-16:0;C171_22:4 459.8440 – $C_{51}H_{94}N_5O_7P^{2+}$
proposed fragmentation scheme

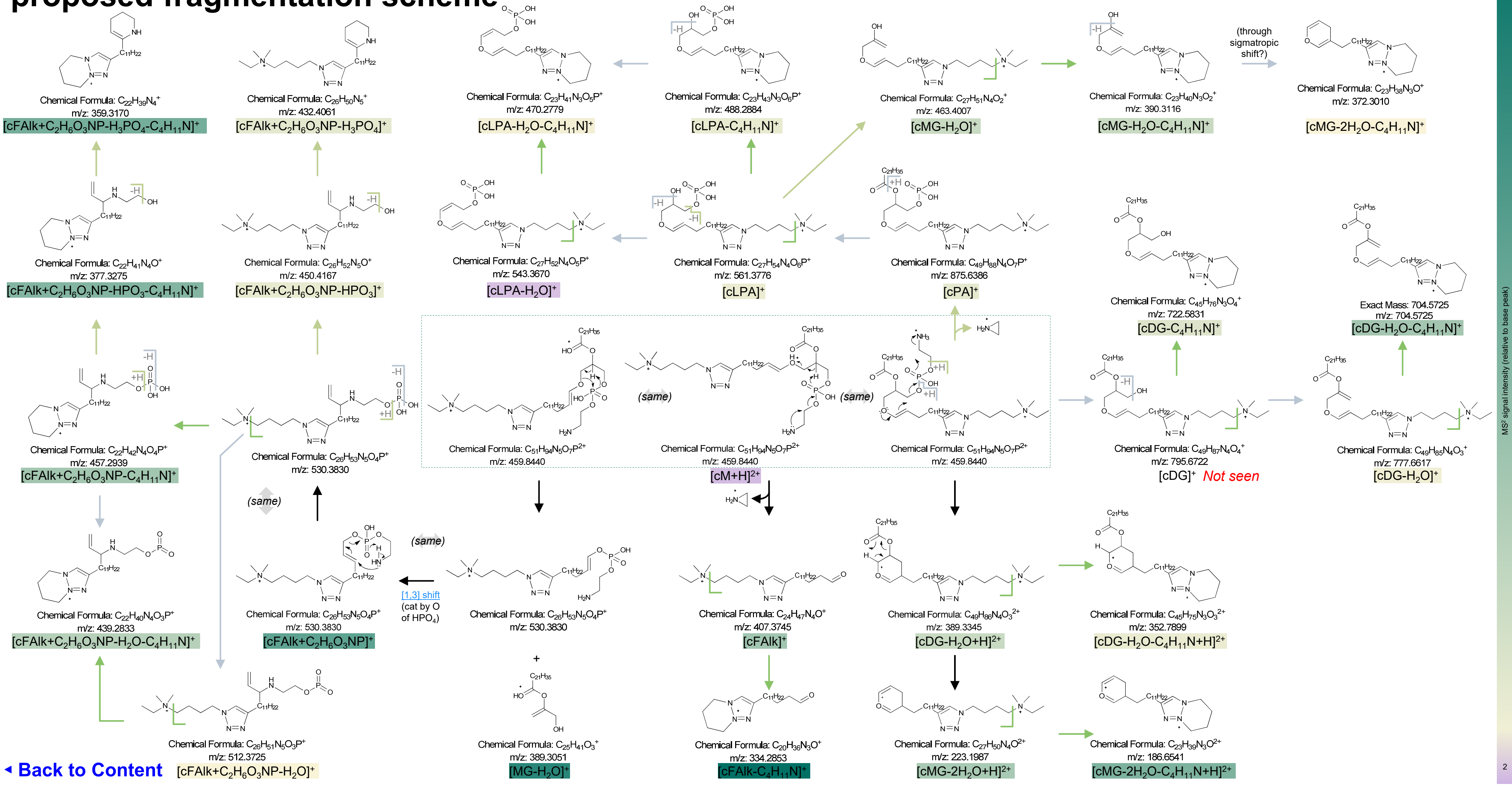

◂ Back to Content

PE P-16:0_18:1;C171 868.6651 – $C_{47}H_{91}N_5O_7P^+$ / 434.8362 – $C_{47}H_{92}N_5O_7P^{2+}$

Extracted Ion Chromatogram

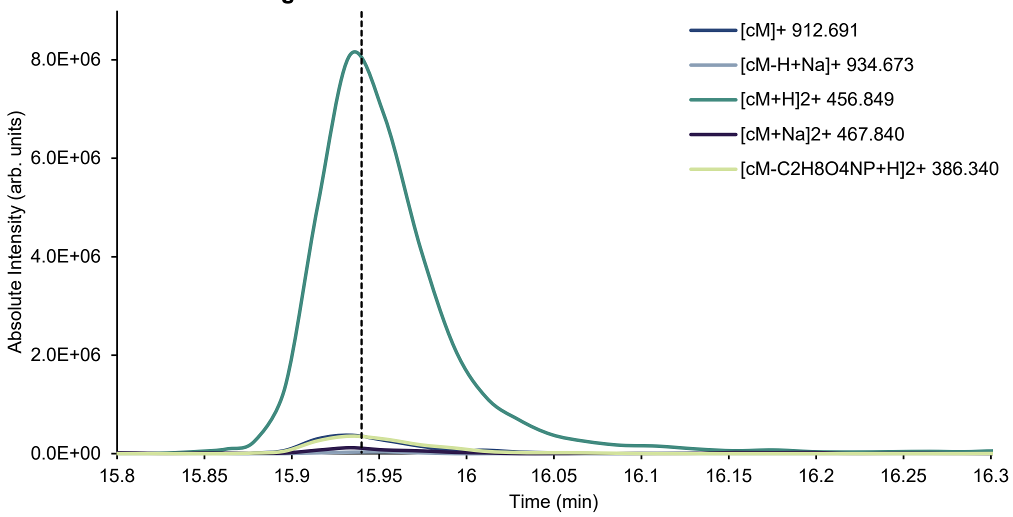

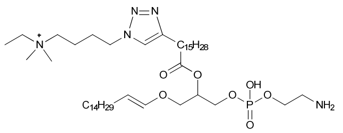

Chemical Formula: $C_{47}H_{91}N_5O_7P^+$
m/z: 868.6651

[cM]$^+$

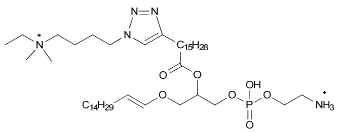

Chemical Formula: $C_{47}H_{92}N_5O_7P^{2+}$
m/z: 434.8362

[cM+H]$^{2+}$

MS² [cM]$^+$

30_40_50 #4846 RT: 15.93 AV: 1 NL: 1.66E5
T: FTMS + p ESI d Full ms2 868.6642@hcd40.00 [90.6650-906.6495]

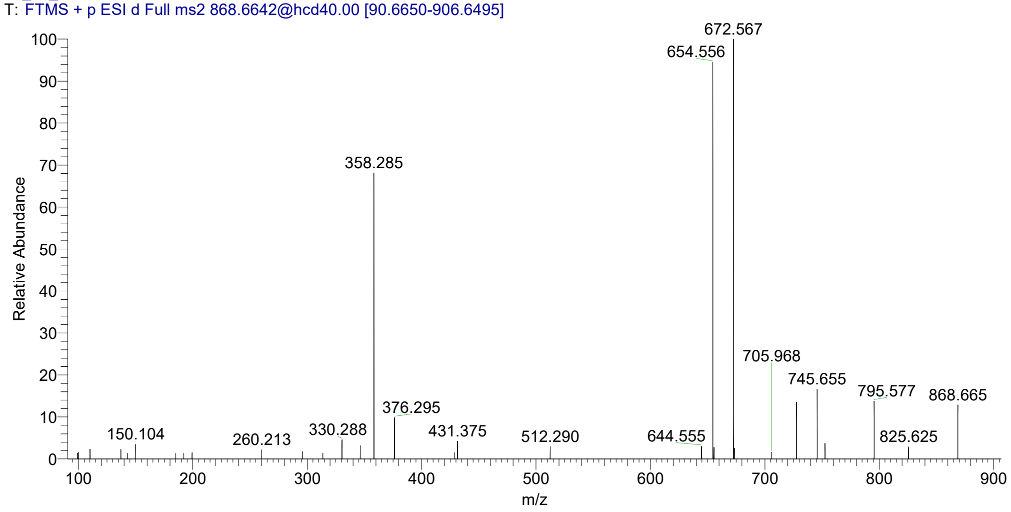

MS² [cM+H]$^{2+}$

25_35_60 #4819 RT: 15.94 AV: 1 NL: 1.64E6
T: FTMS + p ESI d Full ms2 434.8356@hcd40.00 [90.8289-908.2887]

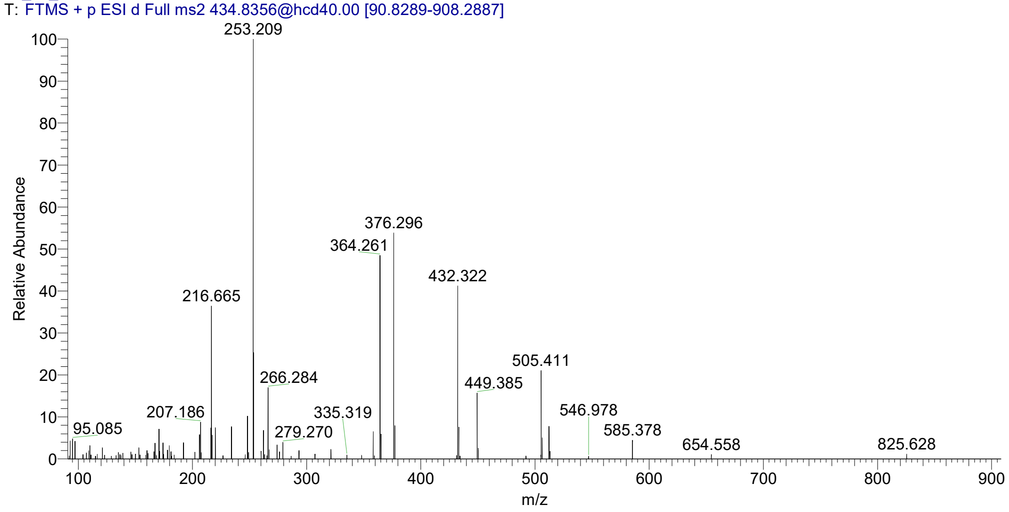

◂ Back to Content

PE P-16:0_18:1;C171 868.6651 – $C_{47}H_{91}N_5O_7P^+$
proposed fragmentation scheme

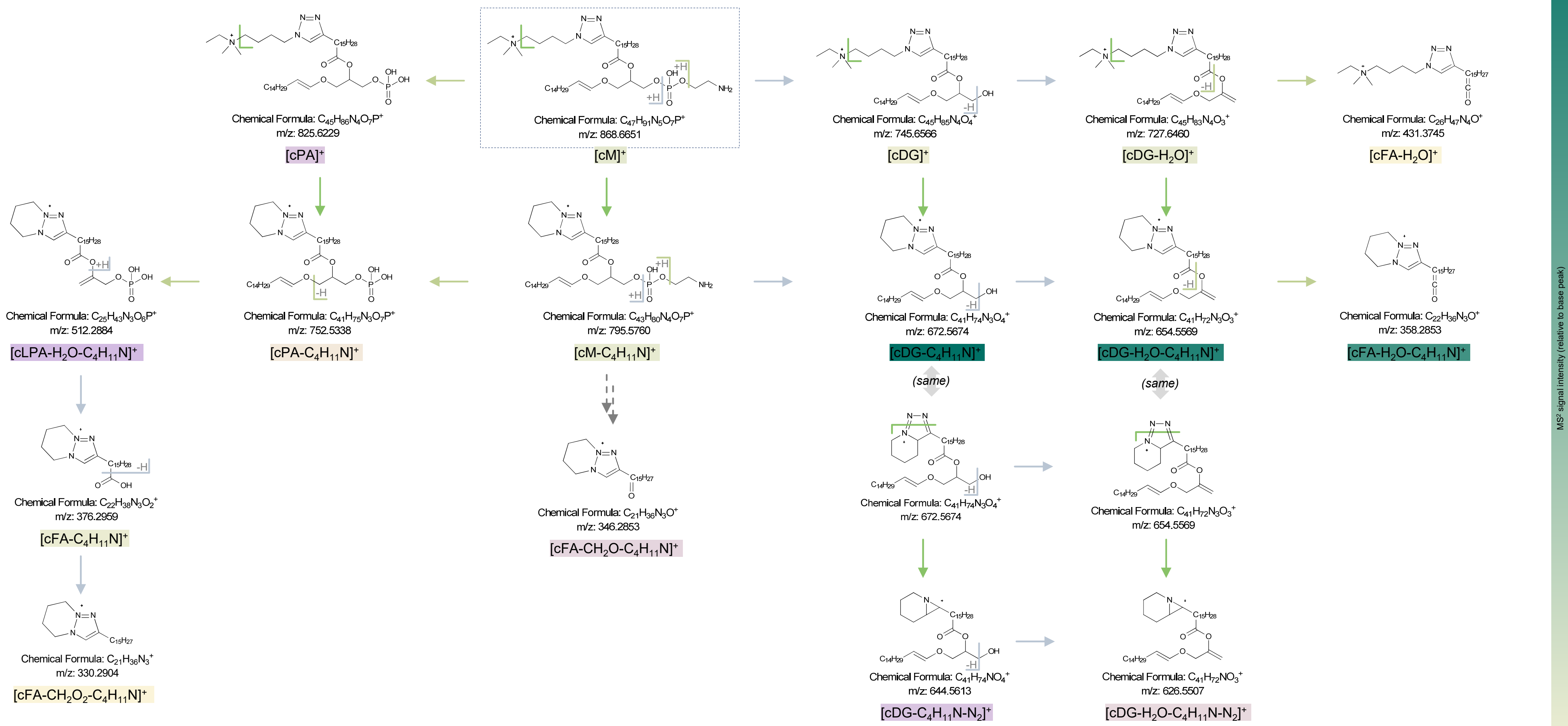

◂ Back to Content

PE P-16:0_18:1;C171 434.8362 – $C_{47}H_{92}N_5O_7P^{2+}$
proposed fragmentation scheme

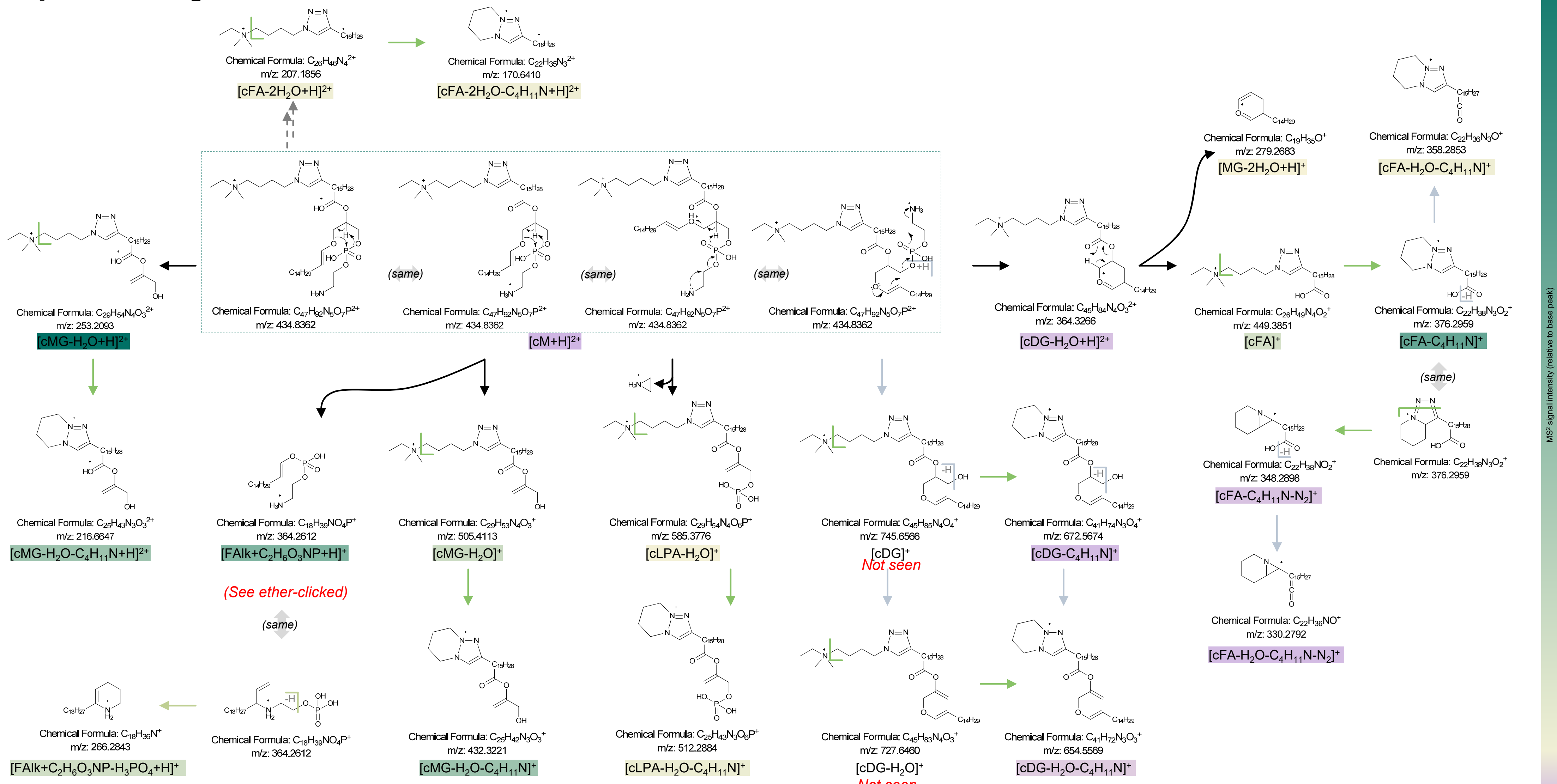

◂ Back to Content

PI 18:1;C171_16:0 1003.6706 – $C_{51}H_{96}N_4O_{13}P^+$

Extracted Ion Chromatogram

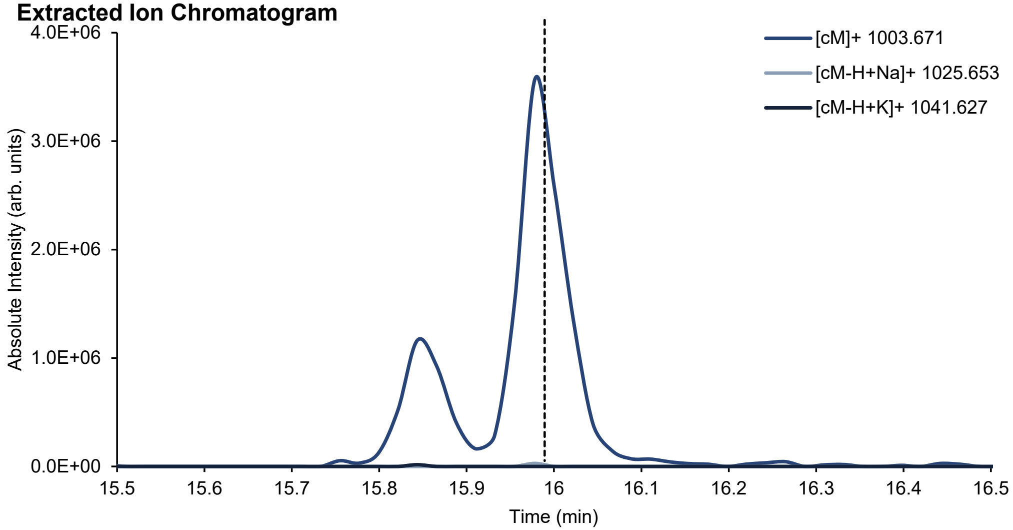

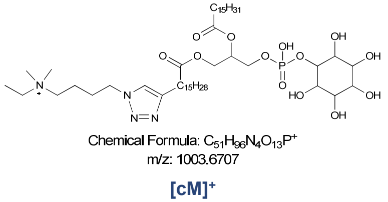

MS² [cM]⁺

Ex_23_50_PN02 #10384 RT: 15.98 AV: 1 NL: 1.43E6
T: FTMS + p ESI d Full ms2 1003.6696@hcd35.00 [104.4355-1044.3550]

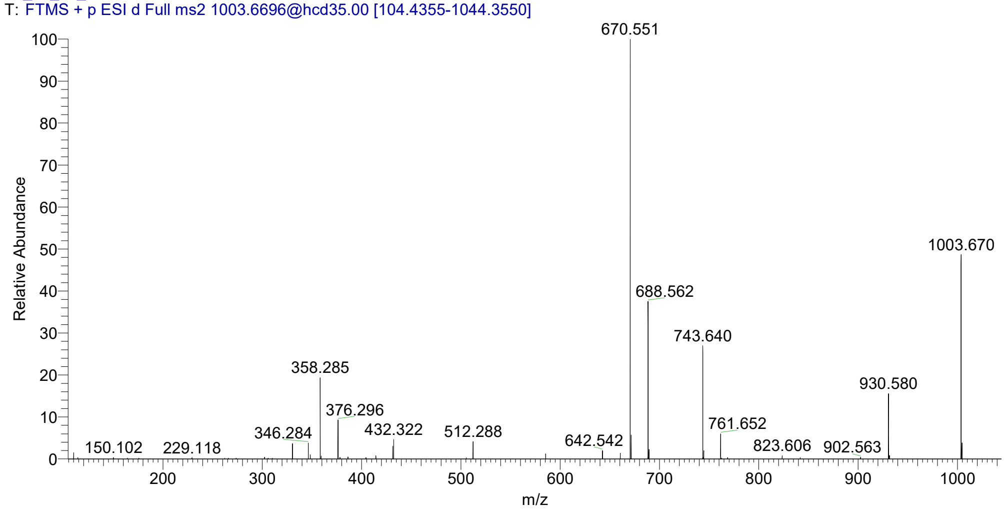

◂ Back to Content

PI 18:1;C171_16:0 1003.6706 – $C_{51}H_{96}N_4O_{13}P^+$ proposed fragmentation scheme

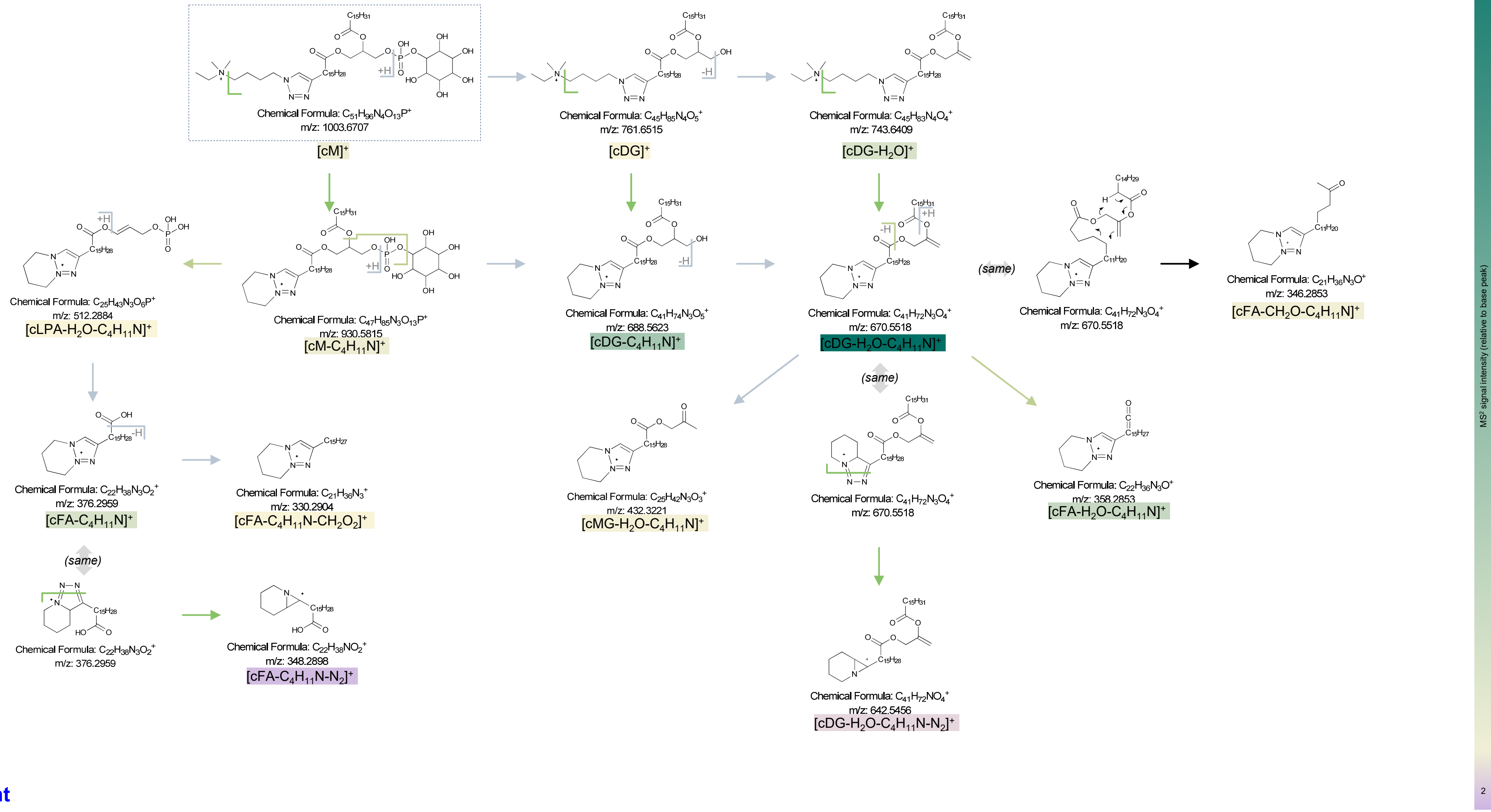

◂ Back to Content

PS 18:1;C171_18:0 956.6811 – $C_{50}H_{95}N_5O_{10}P^+$ / 478.8442 – $C_{50}H_{96}N_5O_{10}P^{2+}$

Extracted Ion Chromatogram

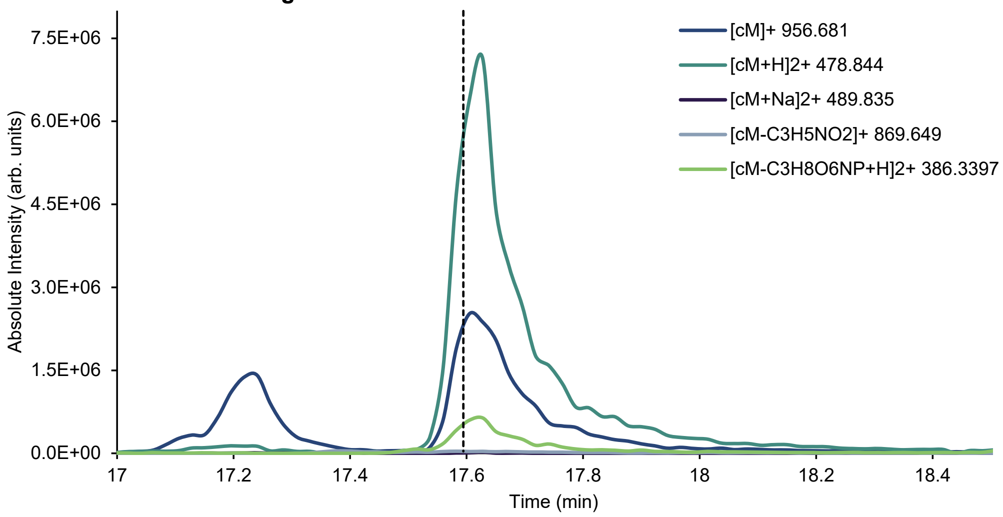

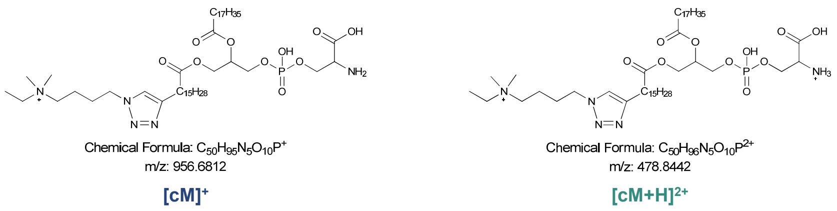

MS² [cM]⁺

6h_O_r1 #11153 RT: 17.59 AV: 1 NL: 1.10E6
T: FTMS + p ESI d Full ms2 956.6812@hcd37.00 [99.6427-996.4269]

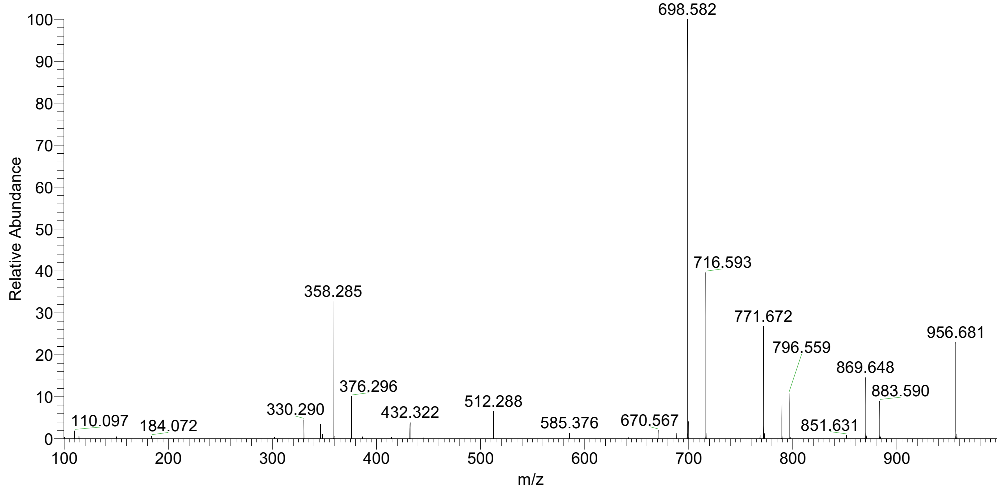

◂ Back to Content

PS 18:1;C171_18:0 956.6811 – $C_{50}H_{95}N_5O_{10}P^+$
proposed fragmentation scheme

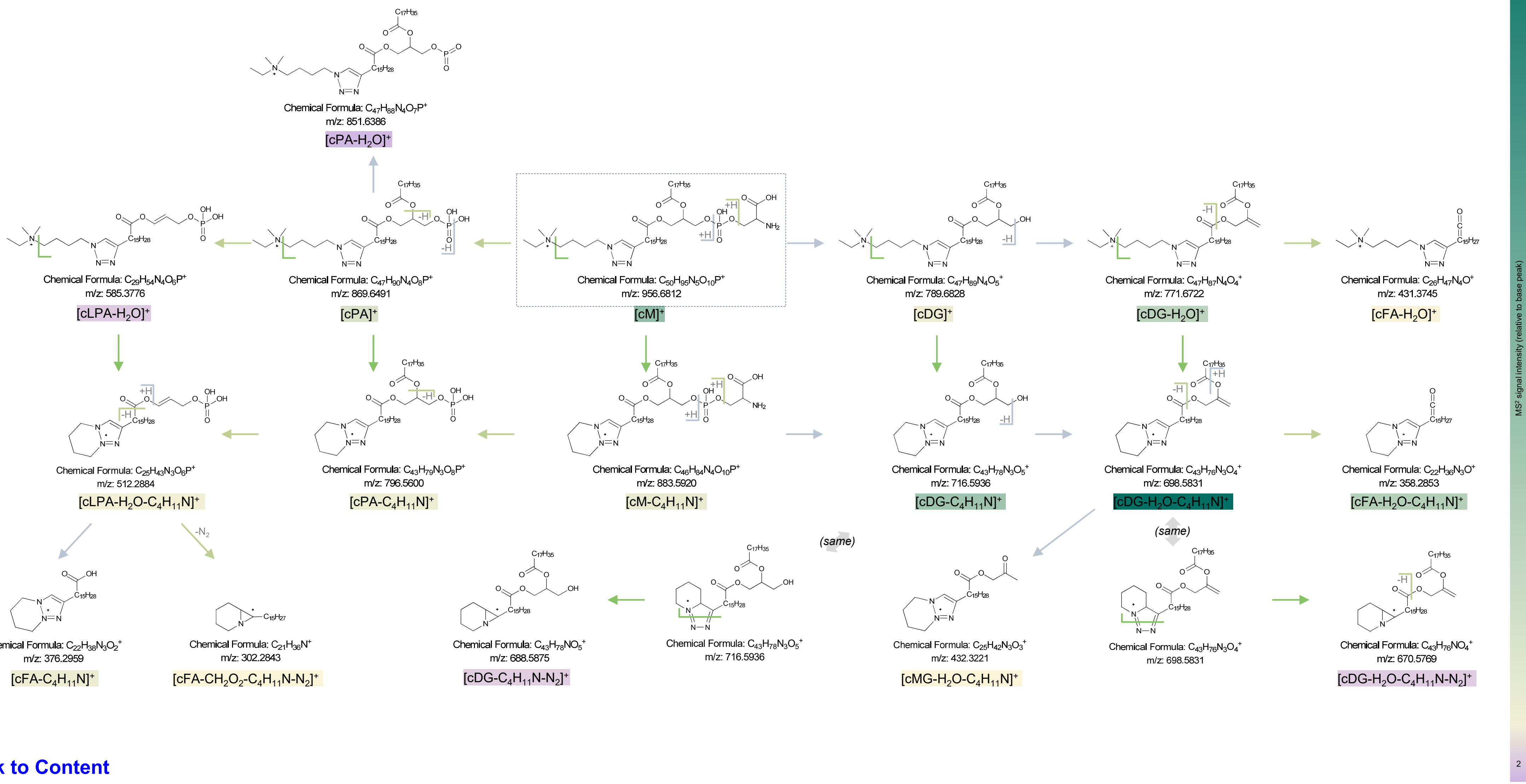

◂ Back to Content

PG 18:1;C171_18:1 941.6702 – $C_{50}H_{94}O_{10}N_4P^+$

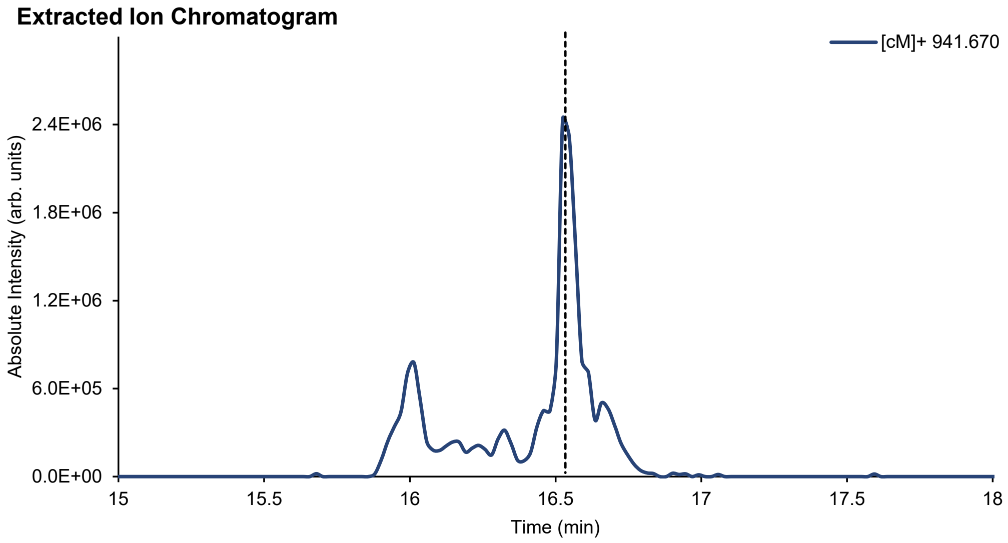

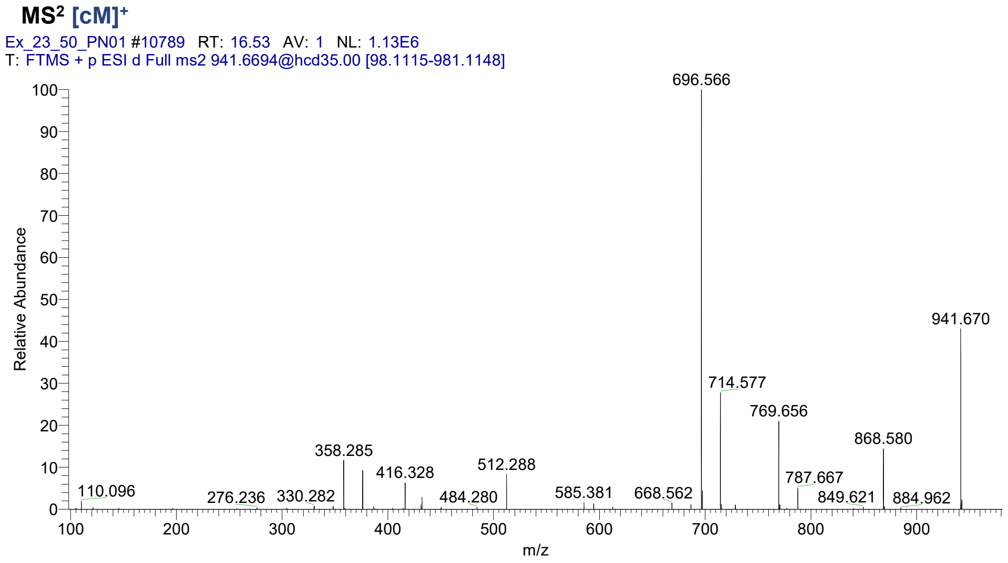

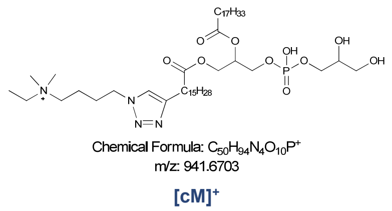

◂ Back to Content

PG 18:1;C171_18:1 941.6702 – $C_{50}H_{94}N_4O_{10}P^+$
proposed fragmentation scheme

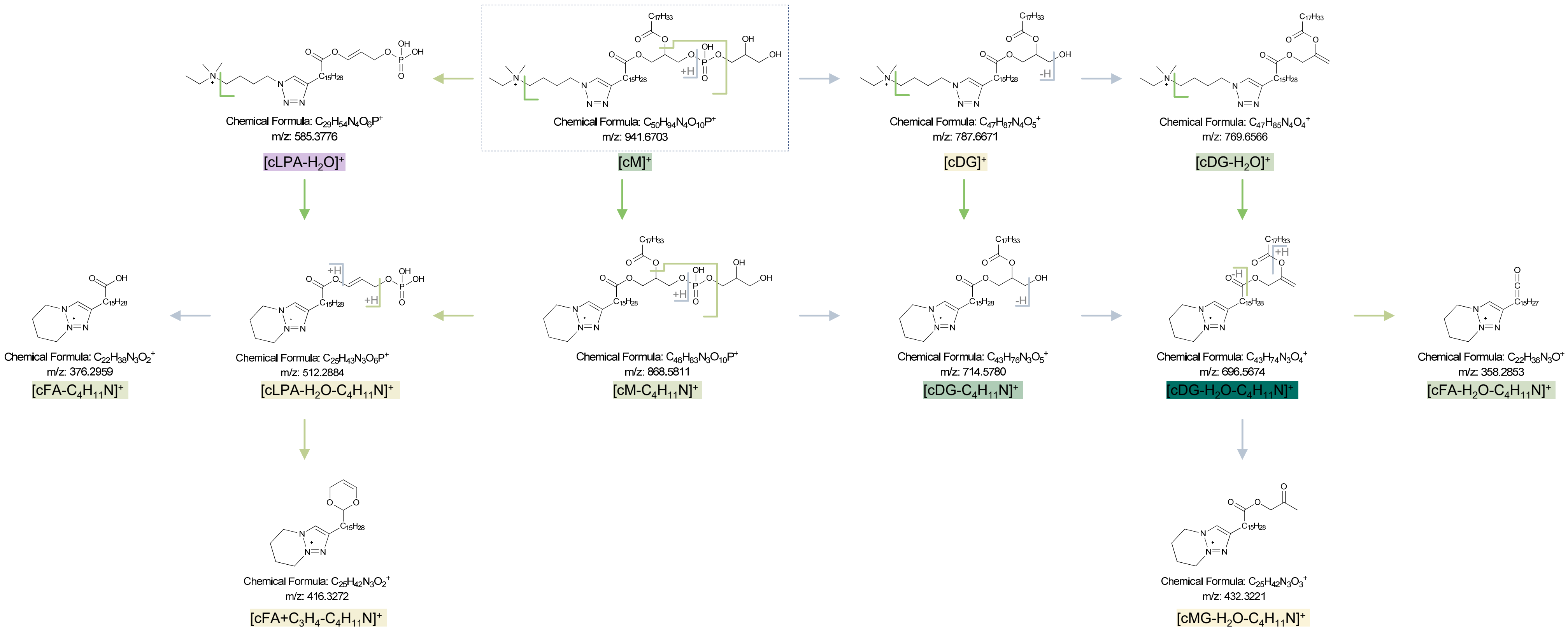

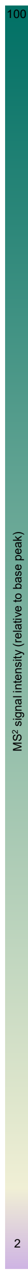

◂ Back to Content

BMP 18:1;C171_18:1 941.6702 – $C_{50}H_{94}O_{10}N_4P^+$

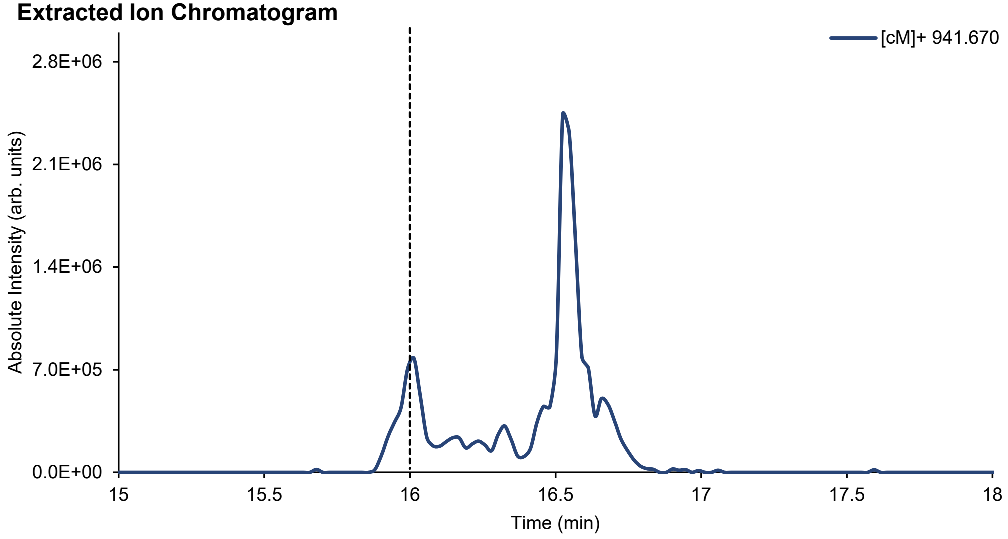

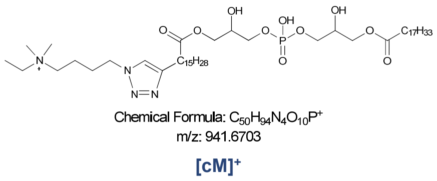

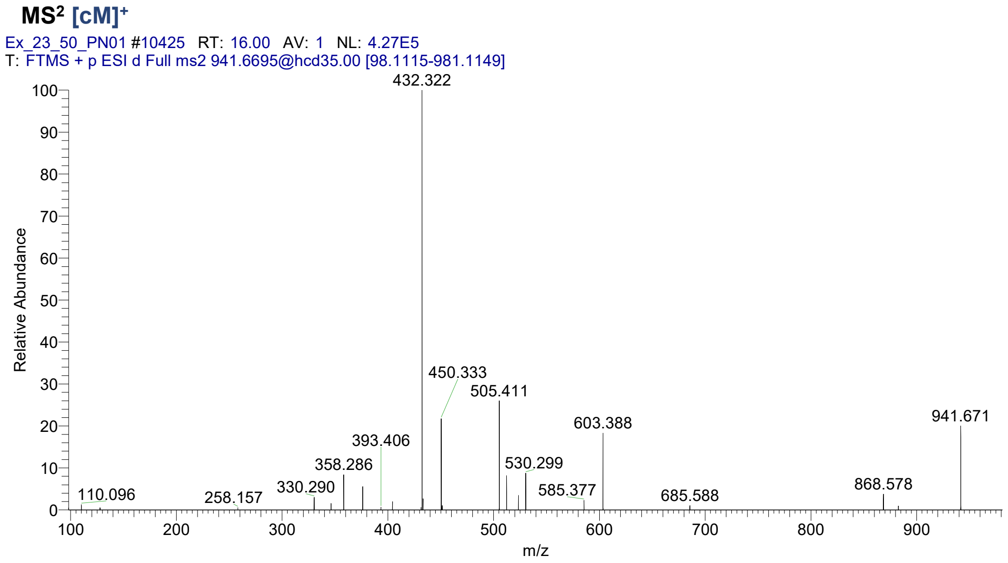

◂ Back to Content

BMP 18:1;C171_18:1 941.6702 – $C_{50}H_{94}N_4O_{10}P^+$
proposed fragmentation scheme

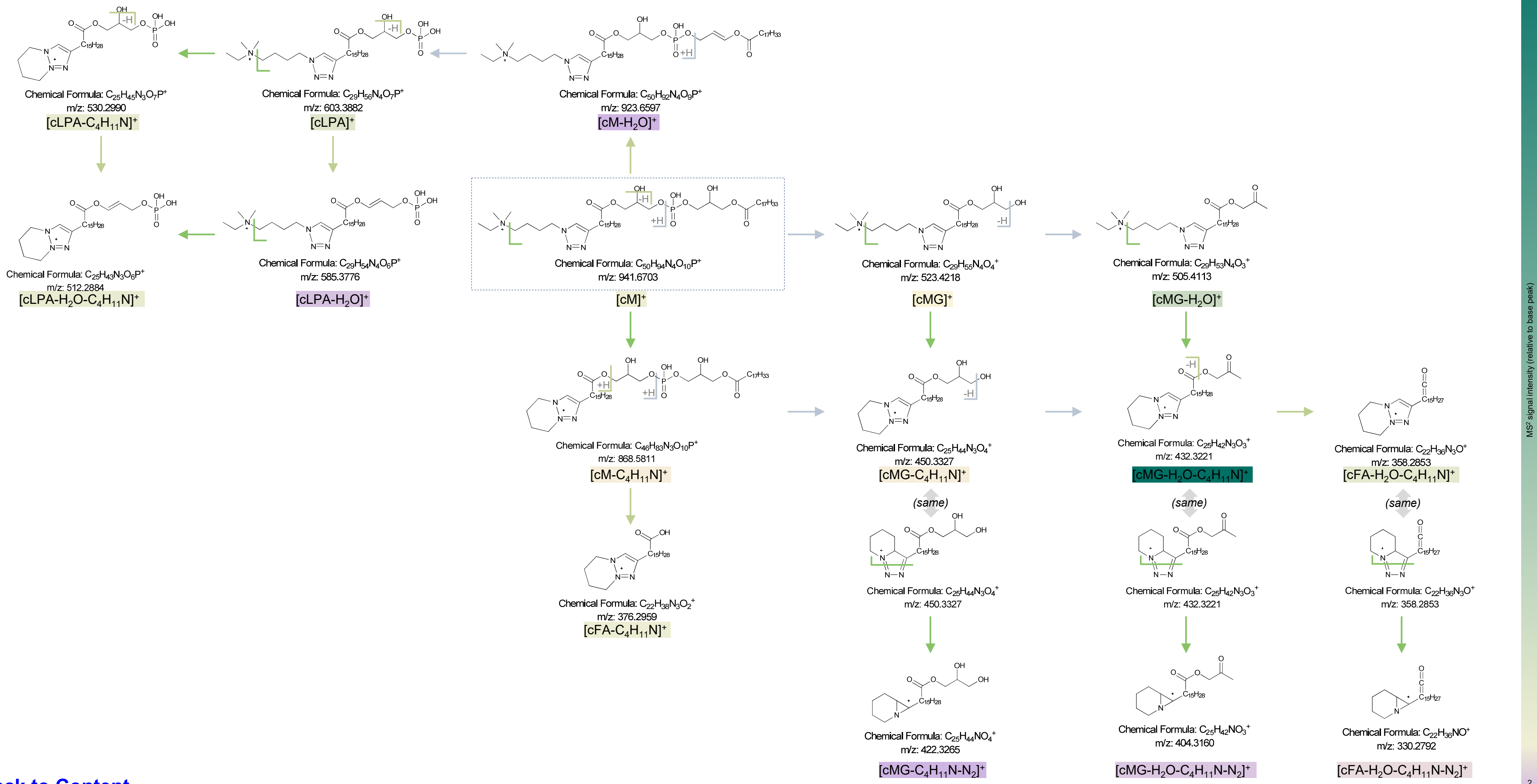

◂ Back to Content

CL 18:1;C171_18:1_16:1_18:1 1596.1326 – $C_{87}H_{161}O_{17}N_4P_2^+$ / 798.5700 – $C_{87}H_{162}O_{17}N_4P_2^{2+}$

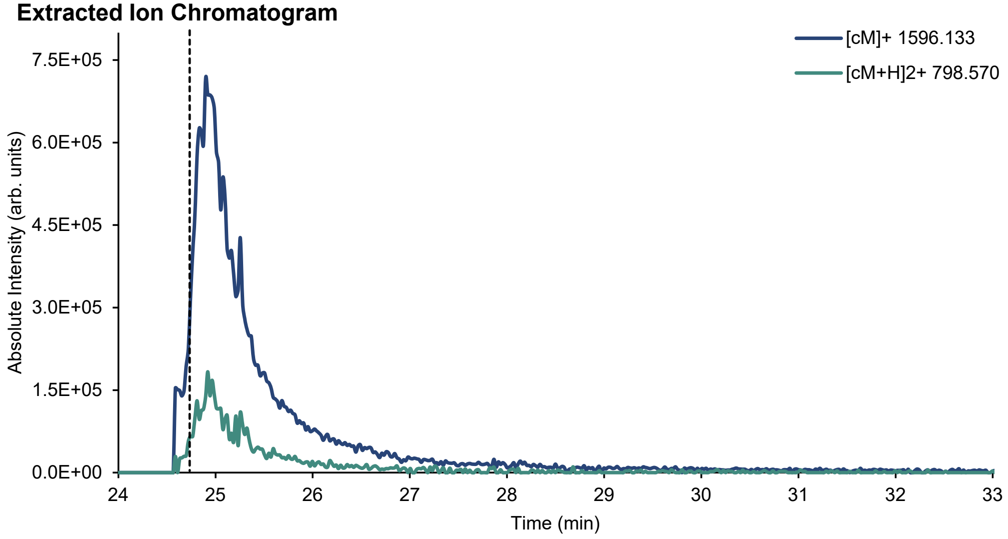

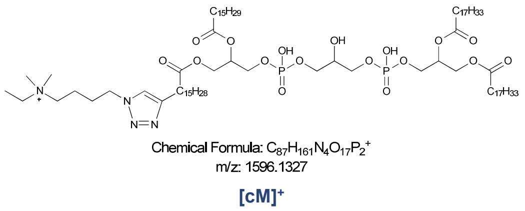

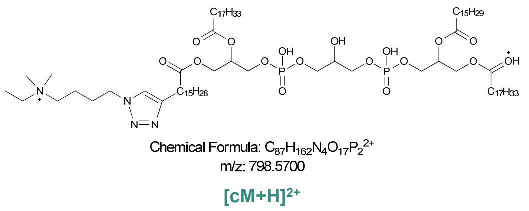

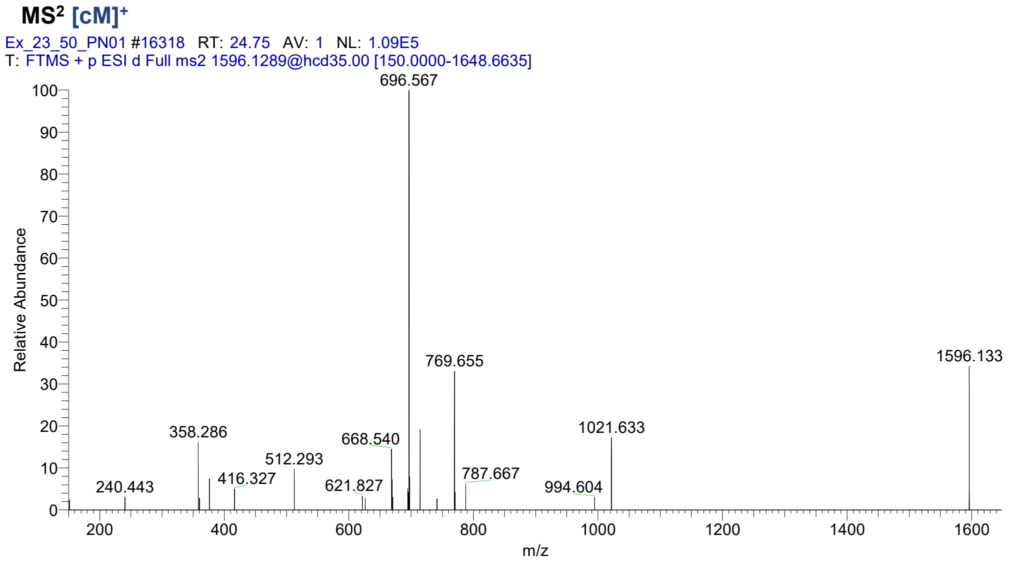

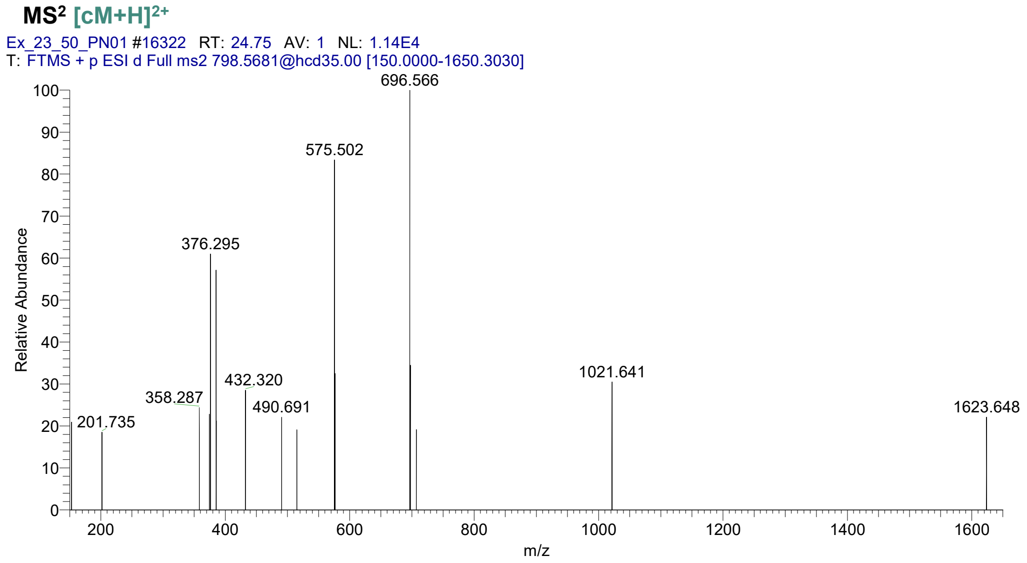

◂ Back to Content

CL 18:1;C171_18:1_16:1_18:1 1596.1326 – $C_{89}H_{165}O_{17}N_4P_2^+$
proposed fragmentation scheme

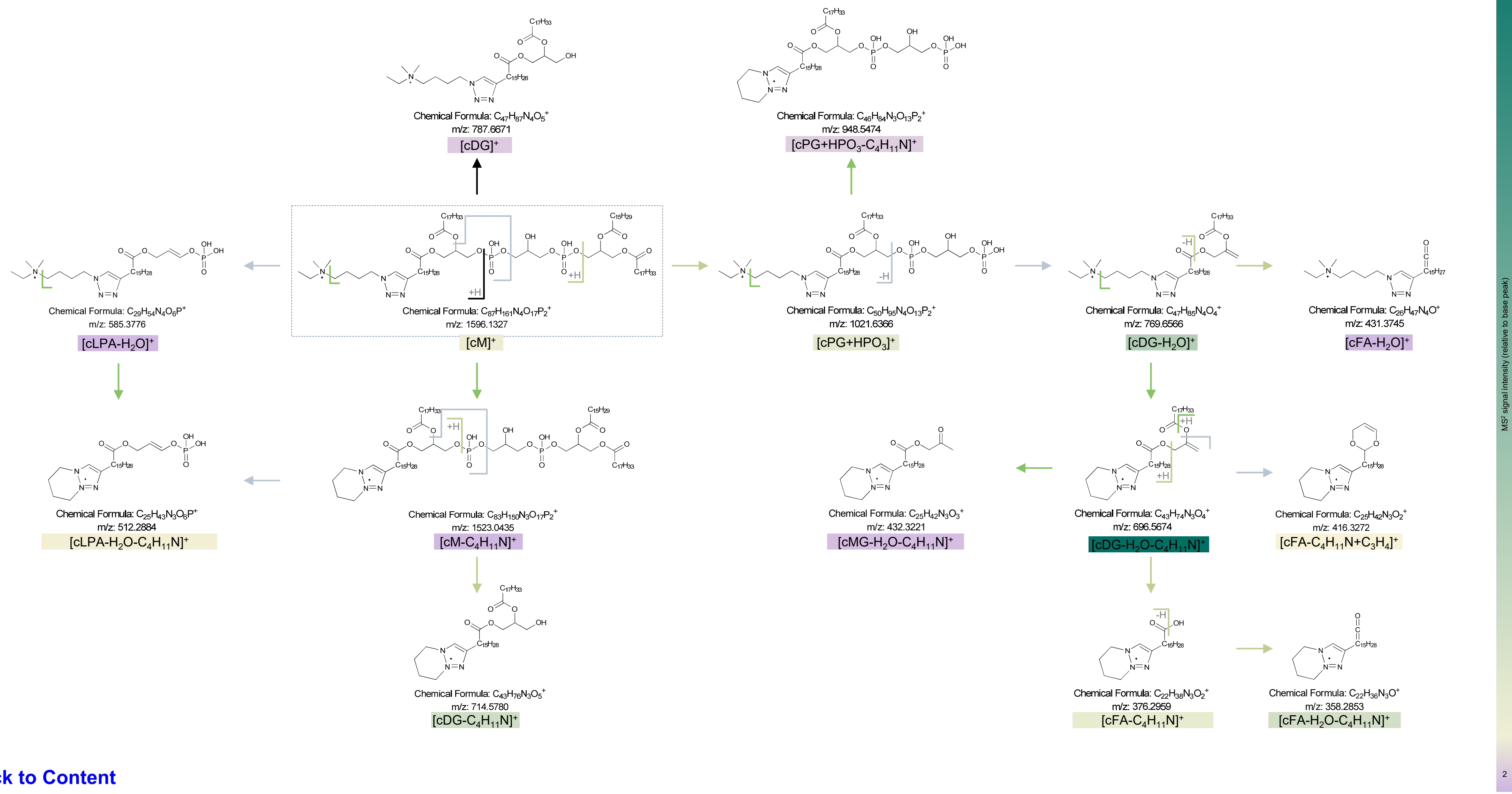

◂ Back to Content

CL 18:1;C171_18:1_16:1_18:1 798.5700 – $C_{87}H_{162}O_{17}N_4P_2^{2+}$
proposed fragmentation scheme

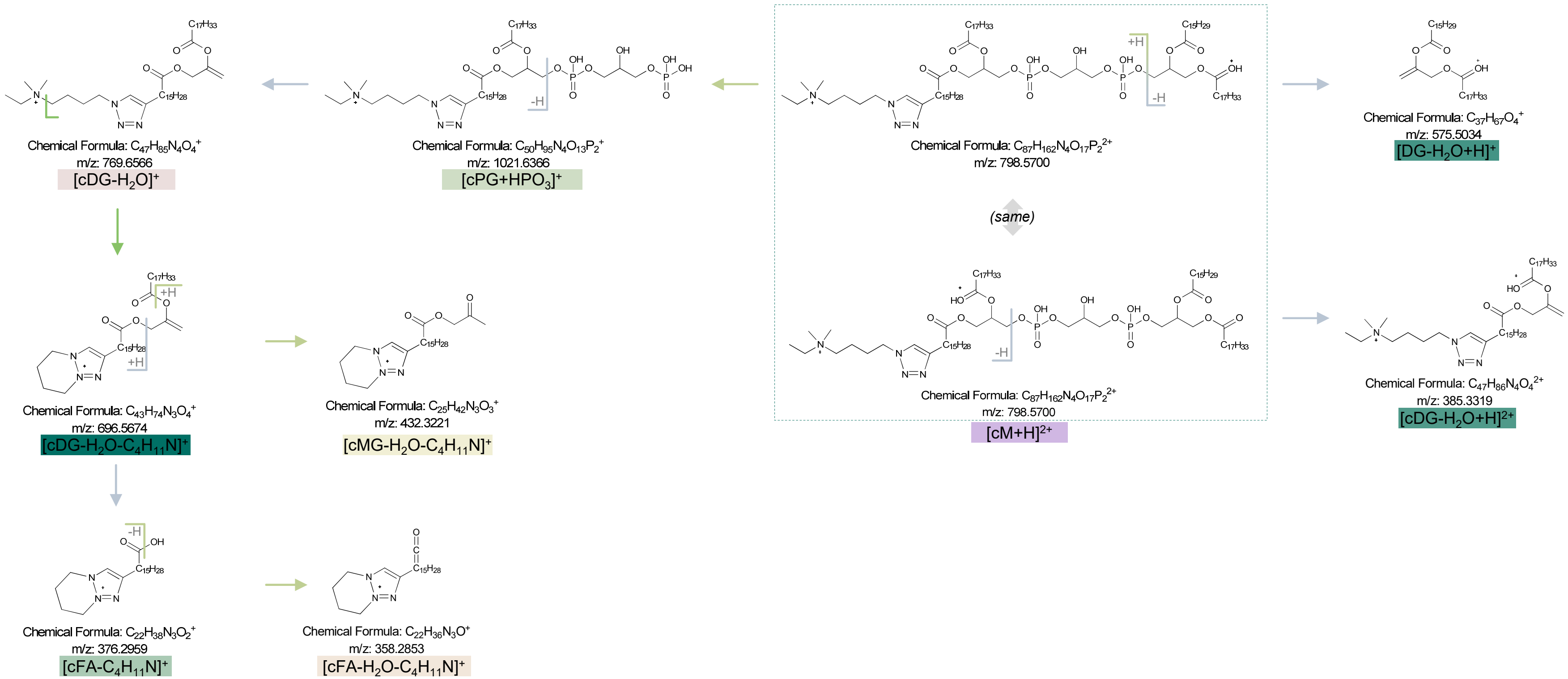

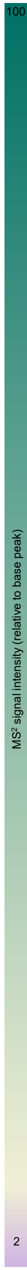

◂ Back to Content

Cer 18:0;O2/24:1;C171 816.7658 – $C_{50}H_{98}O_3N_5^+$ / 408.8868 – $C_{50}H_{99}O_3N_5^{2+}$

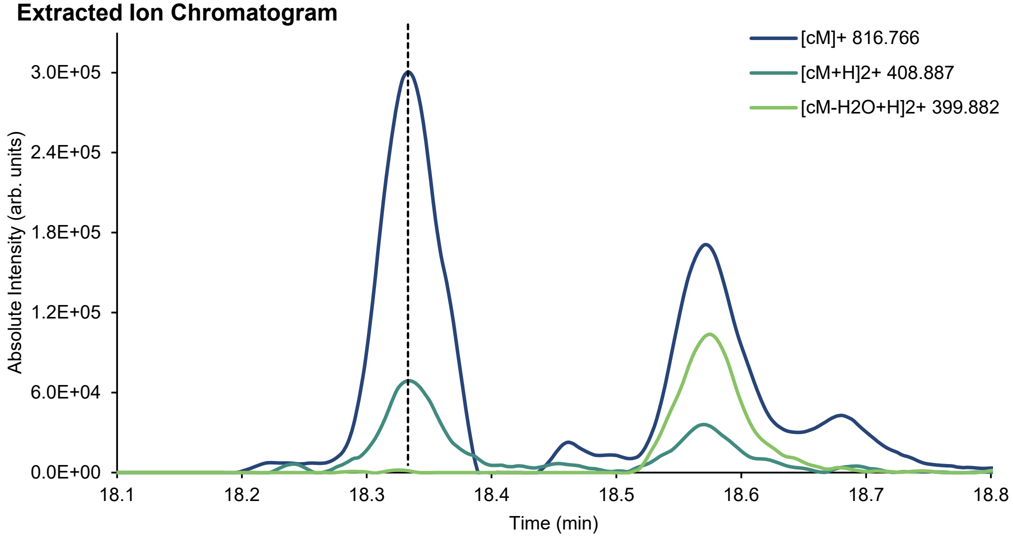

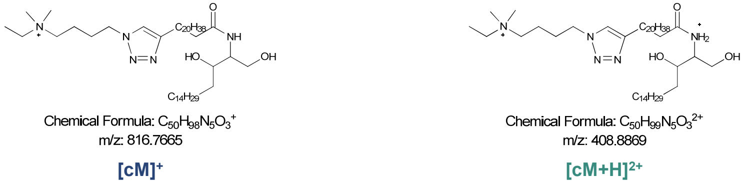

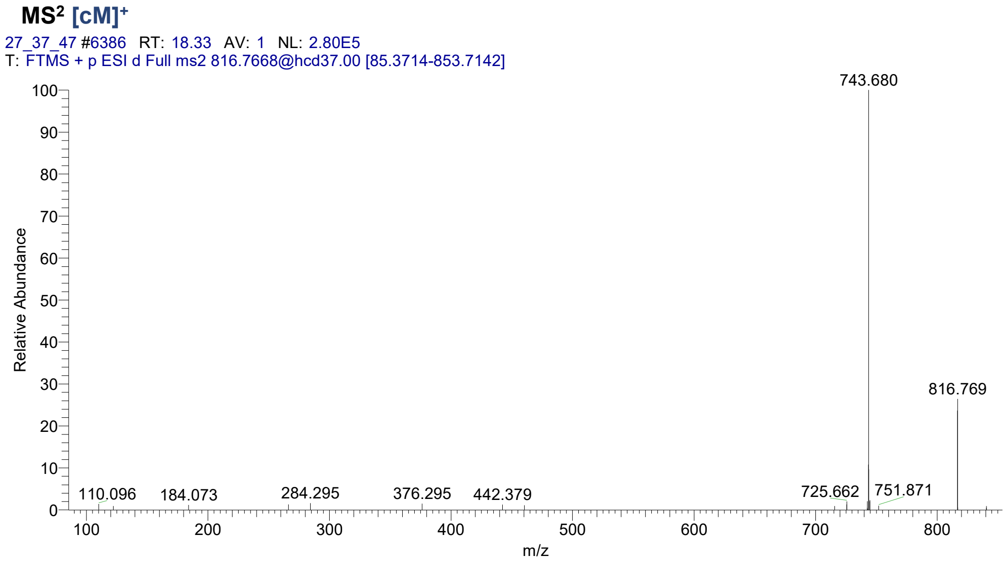

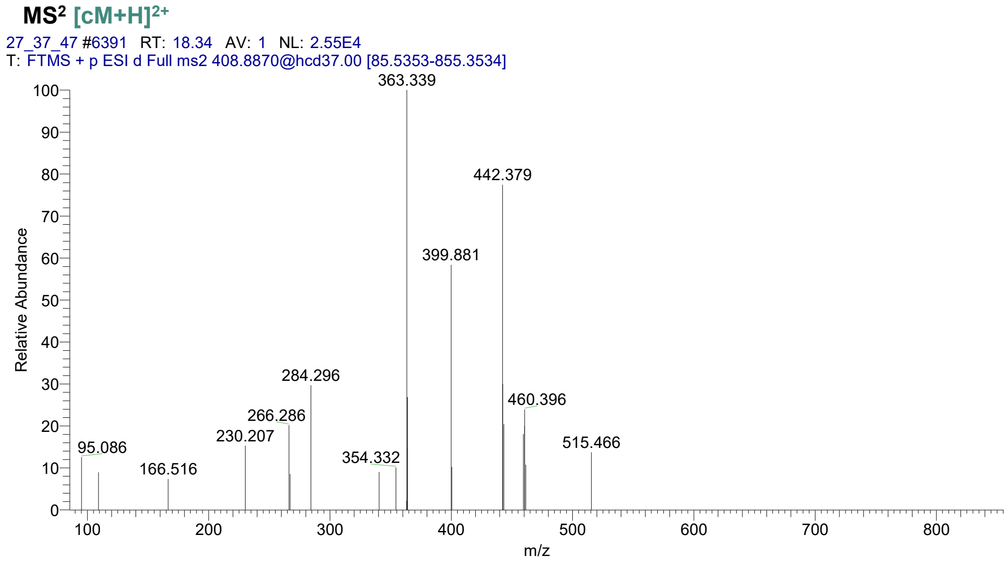

◂ Back to Content

Cer 18:0;O2/24:1;C171 816.7658 – $C_{50}H_{98}O_3N_5^+$
proposed fragmentation scheme

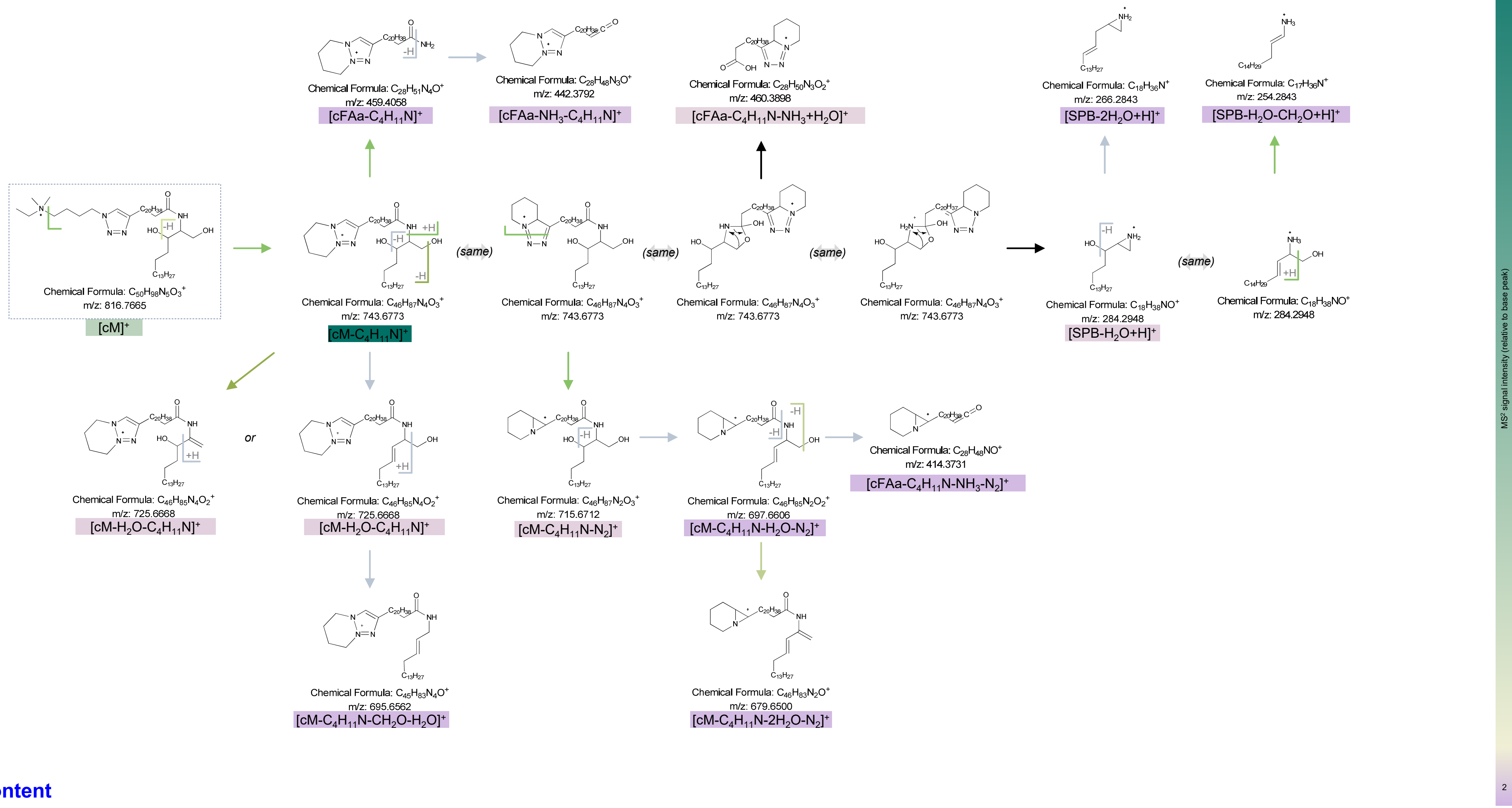

◂ Back to Content

Cer 18:0;O2/24:1;C171 408.8868 – $C_{50}H_{99}O_3N_5^{2+}$ proposed fragmentation scheme

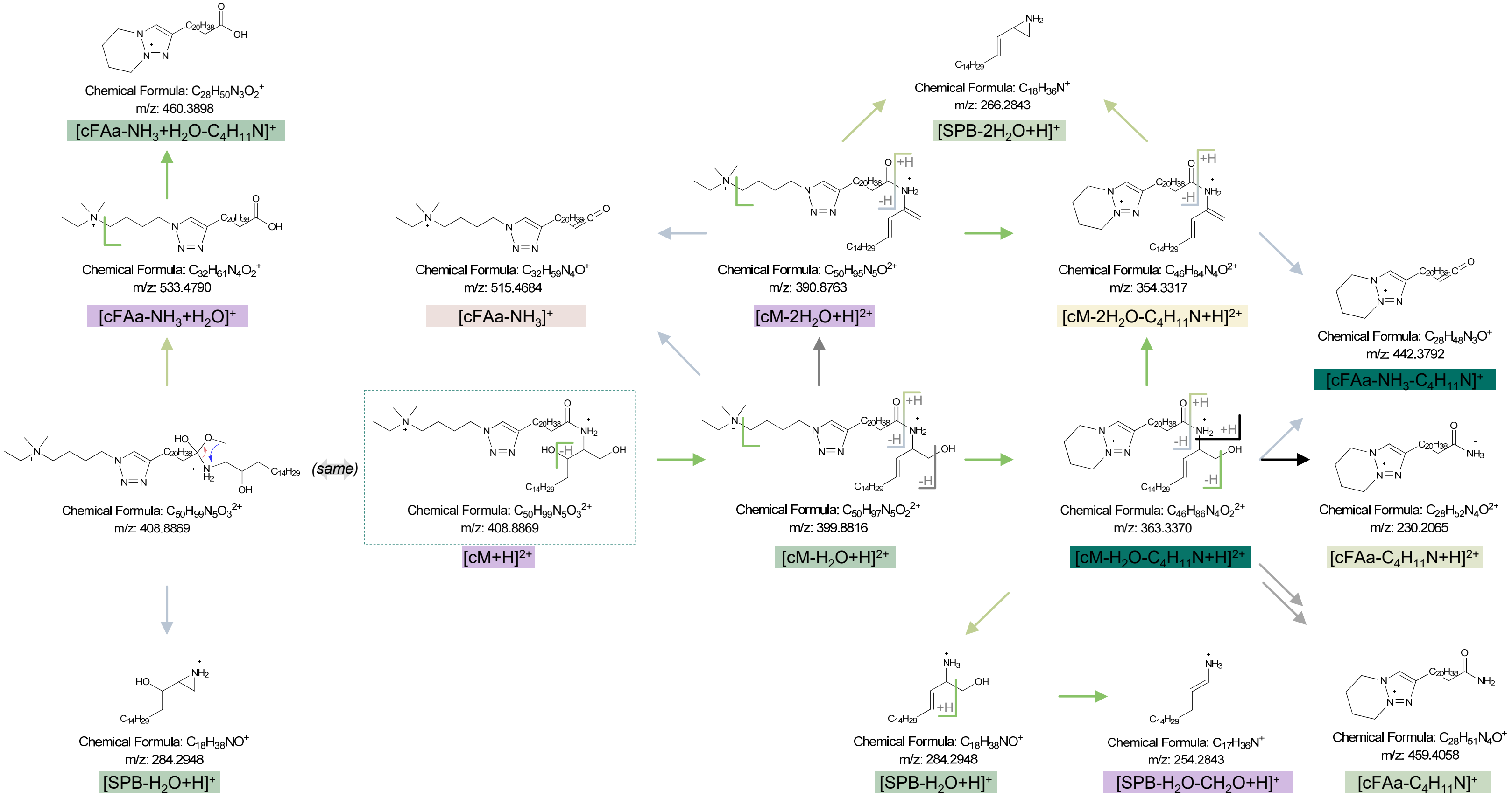

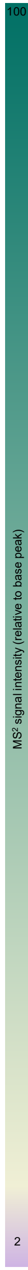

◂ Back to Content

Cer 18:1;O2/24:1;C171 814.7508 – $C_{50}H_{96}O_3N_5^+$ / 407.8790 – $C_{50}H_{97}O_3N_5^{2+}$

Extracted Ion Chromatogram

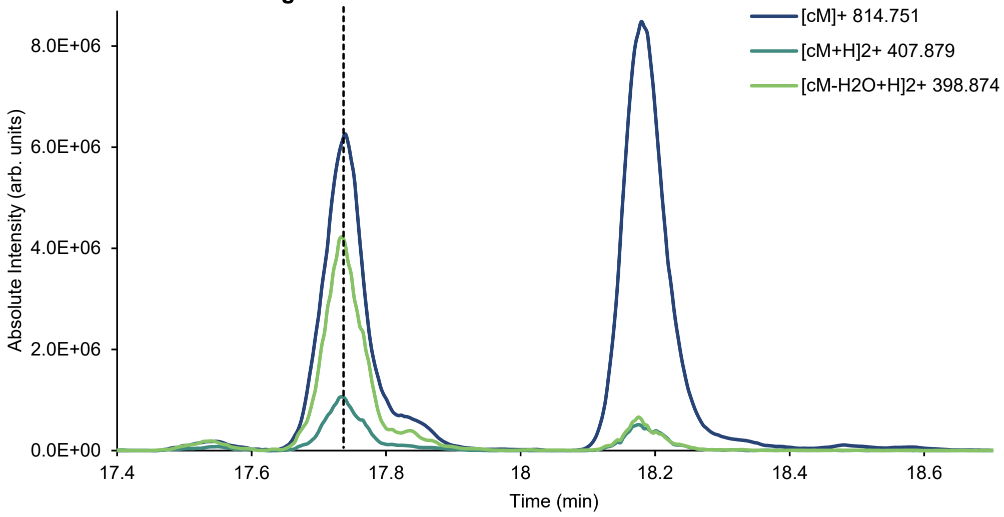

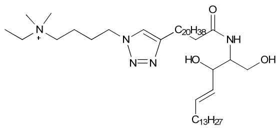

Chemical Formula: $C_{50}H_{96}N_5O_3^+$
m/z: 814.7508
$[cM]^+$

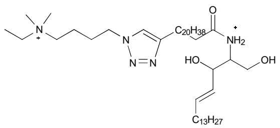

Chemical Formula: $C_{50}H_{97}N_5O_3^{2+}$
m/z: 407.8790
$[cM+H]^{2+}$

MS^2 $[cM]^+$

Ex_24_32_PN29_S9_aO_DDA_1 #11690 RT: 17.74 AV: 1 NL: 9.37E6
T: FTMS + p ESI d Full ms2 814.7507@hcd37.00 [85.1658-851.6577]

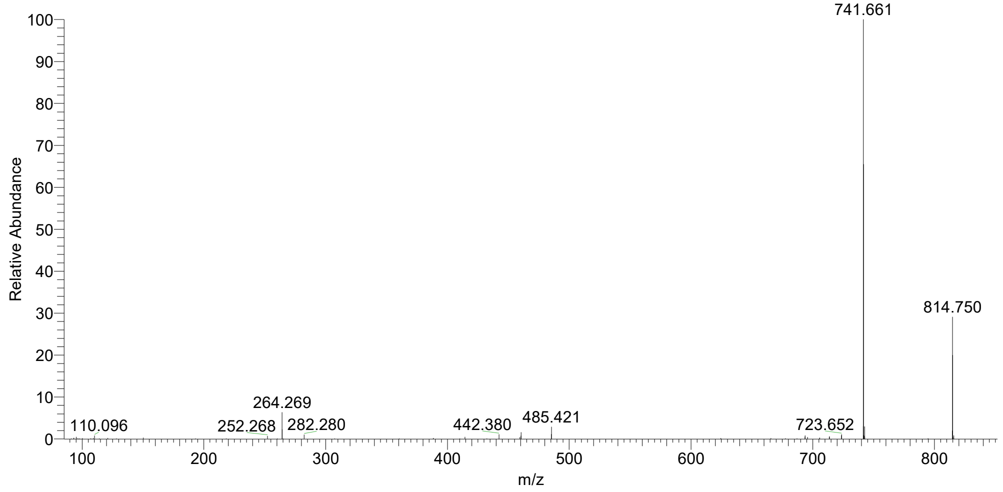

MS^2 $[cM+H]^{2+}$

Ex_24_32_PN29_S9_aO_DDA_1 #11692 RT: 17.75 AV: 1 NL: 9.54E5
T: FTMS + p ESI d Full ms2 407.8790@hcd37.00 [85.3297-853.2971]

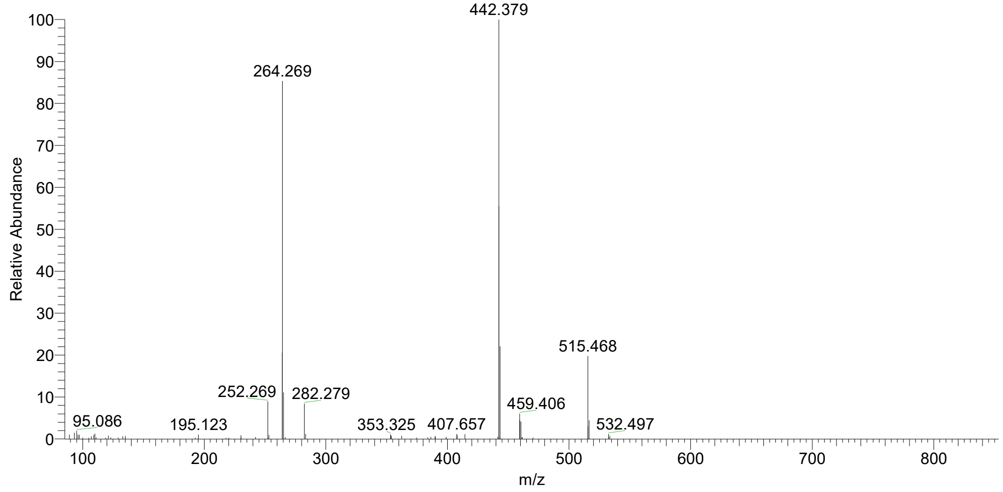

◂ Back to Content

Cer 18:1;O2/24:1;C171 814.7508 – $C_{50}H_{96}O_3N_5^+$ proposed fragmentation scheme

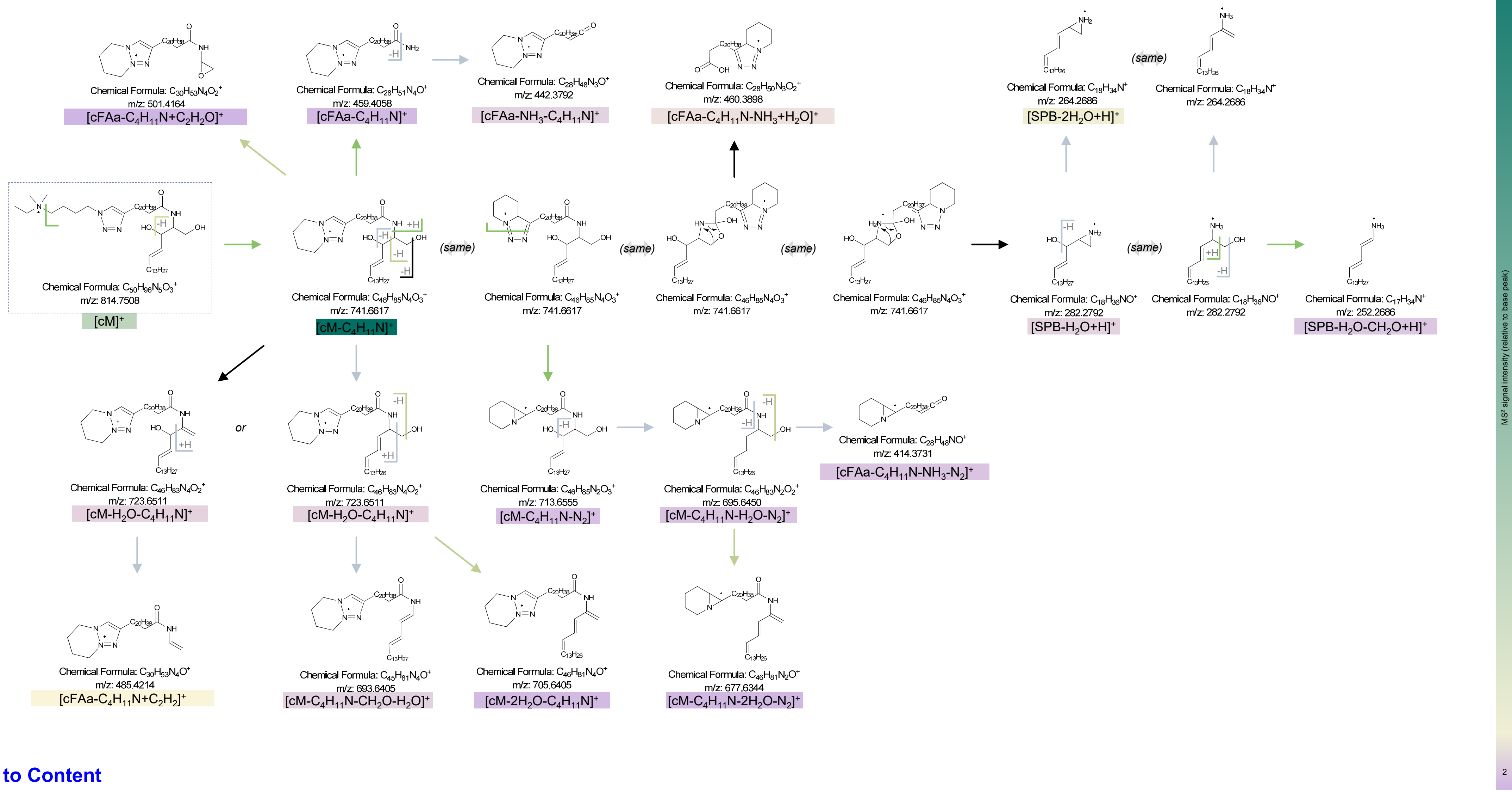

◂ Back to Content

Cer 18:1;O2/24:1;C171 407.8790 – $C_{50}H_{97}O_3N_5^{2+}$
proposed fragmentation scheme

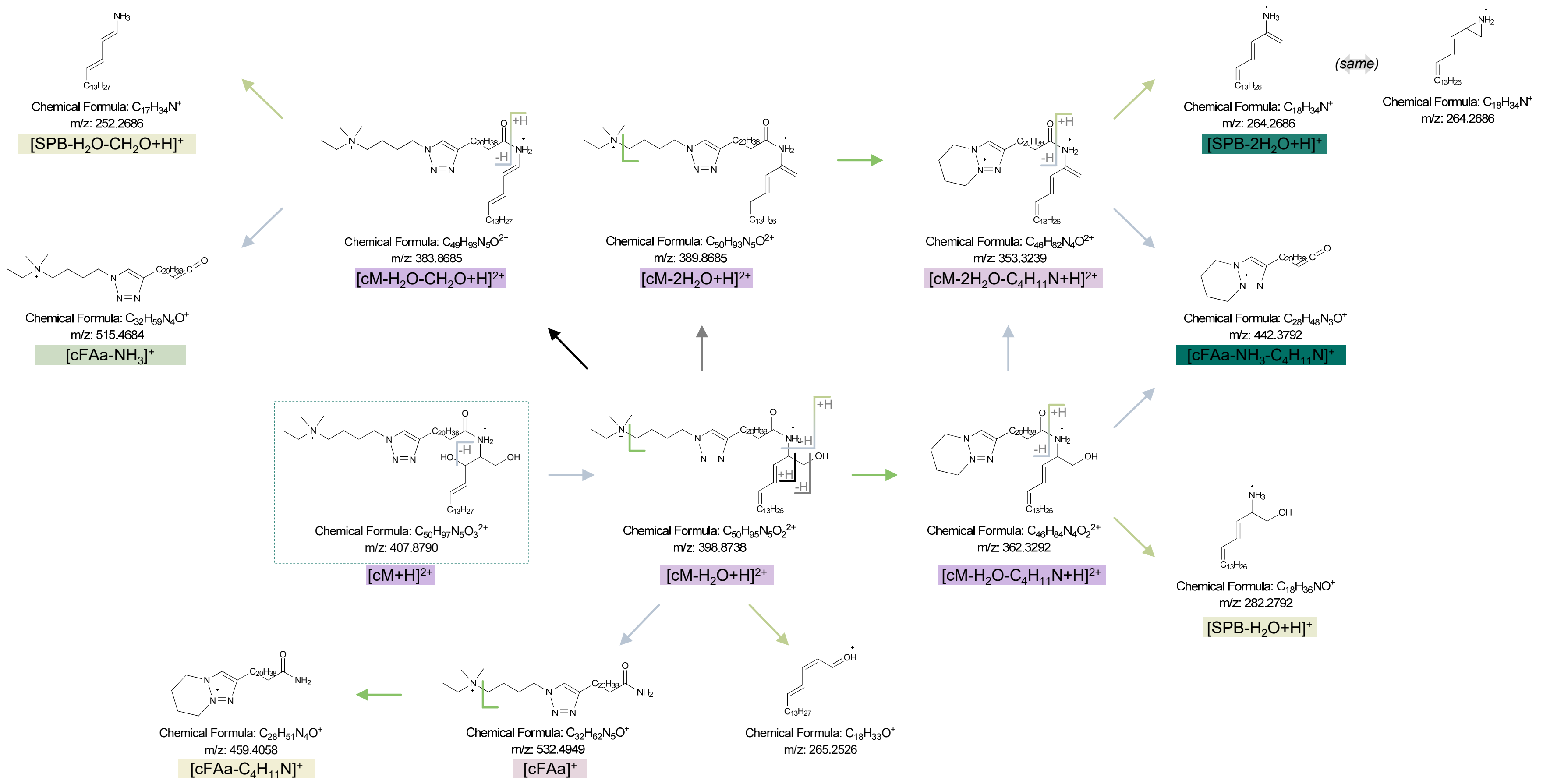

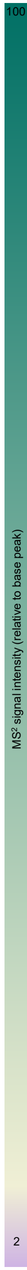

◂ Back to Content

Cer 18:1;O2,C171/24:1 814.7508 – $C_{50}H_{96}O_3N_5^+$ / 407.8790 – $C_{50}H_{97}O_3N_5^{2+}$

Extracted Ion Chromatogram

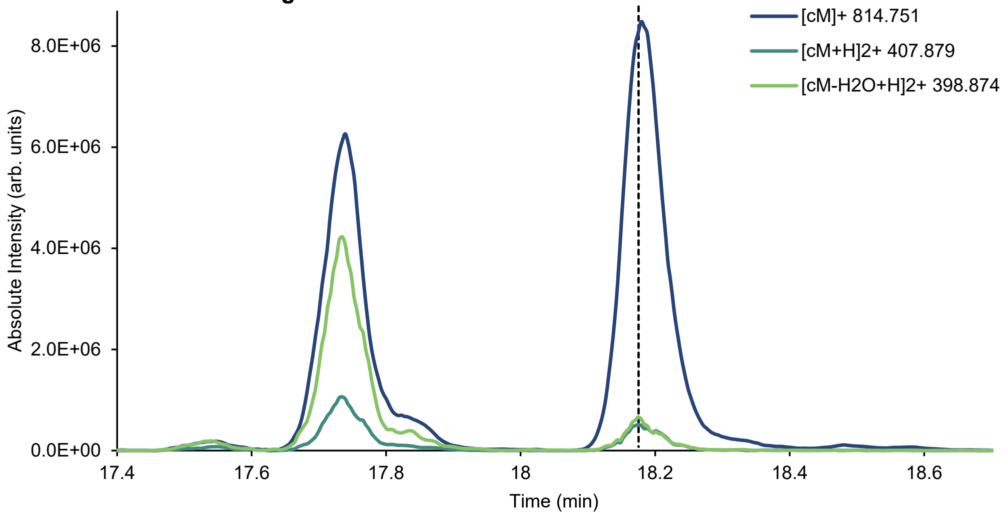

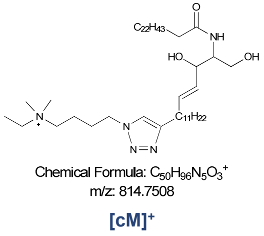

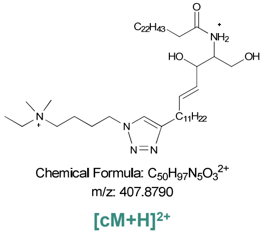

MS² [cM]⁺

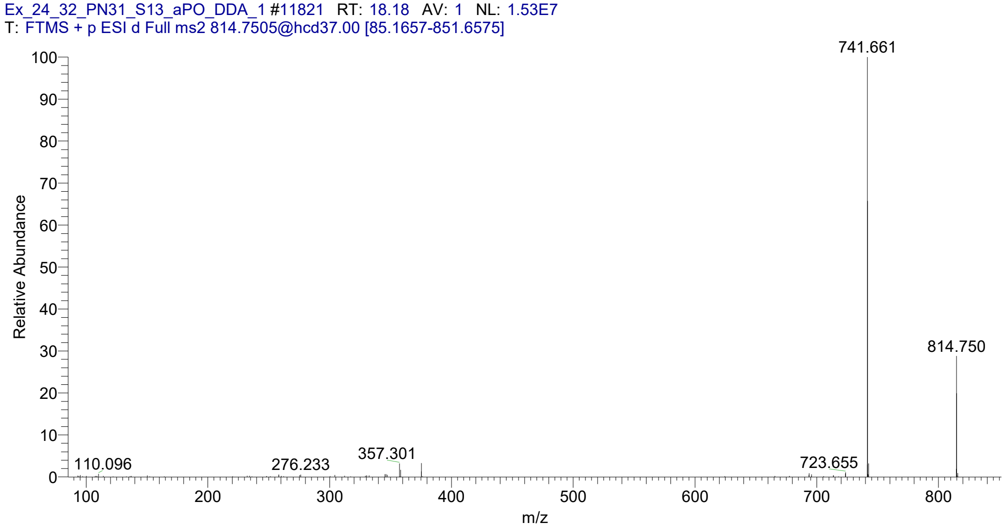

MS² [cM+H]²⁺

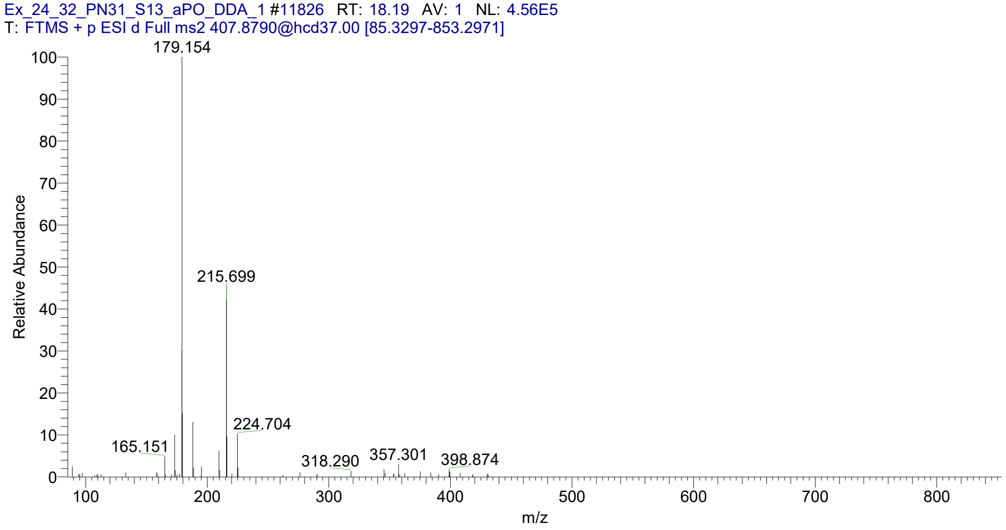

◂ Back to Content

Cer 18:1;O2,C171/24:1 814.7508 – $C_{50}H_{96}O_3N_5^+$ proposed fragmentation scheme

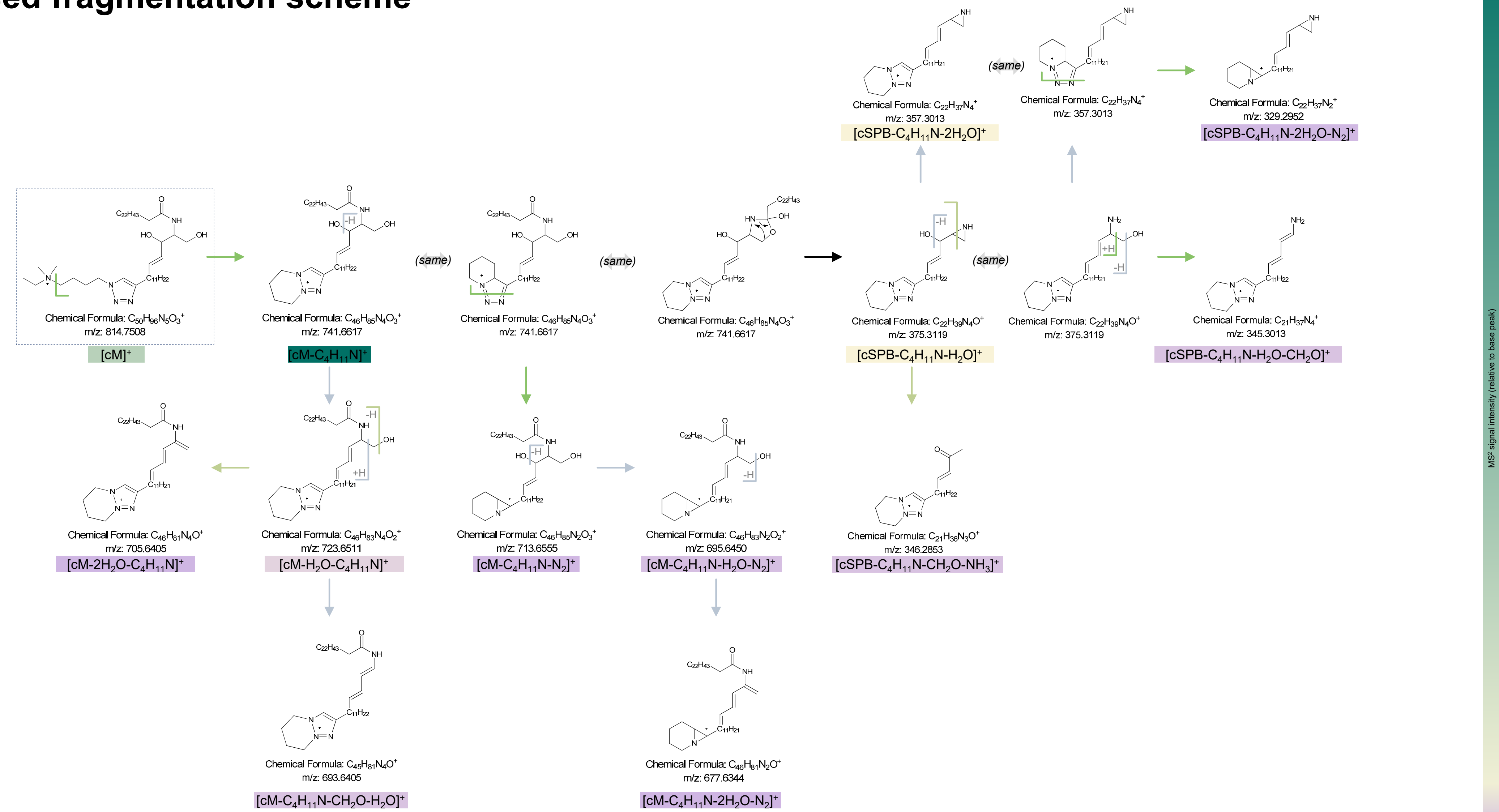

◂ Back to Content

Cer 18:1;O2,C171/24:1 407.8790 – $C_{50}H_{97}O_3N_5^{2+}$
proposed fragmentation scheme

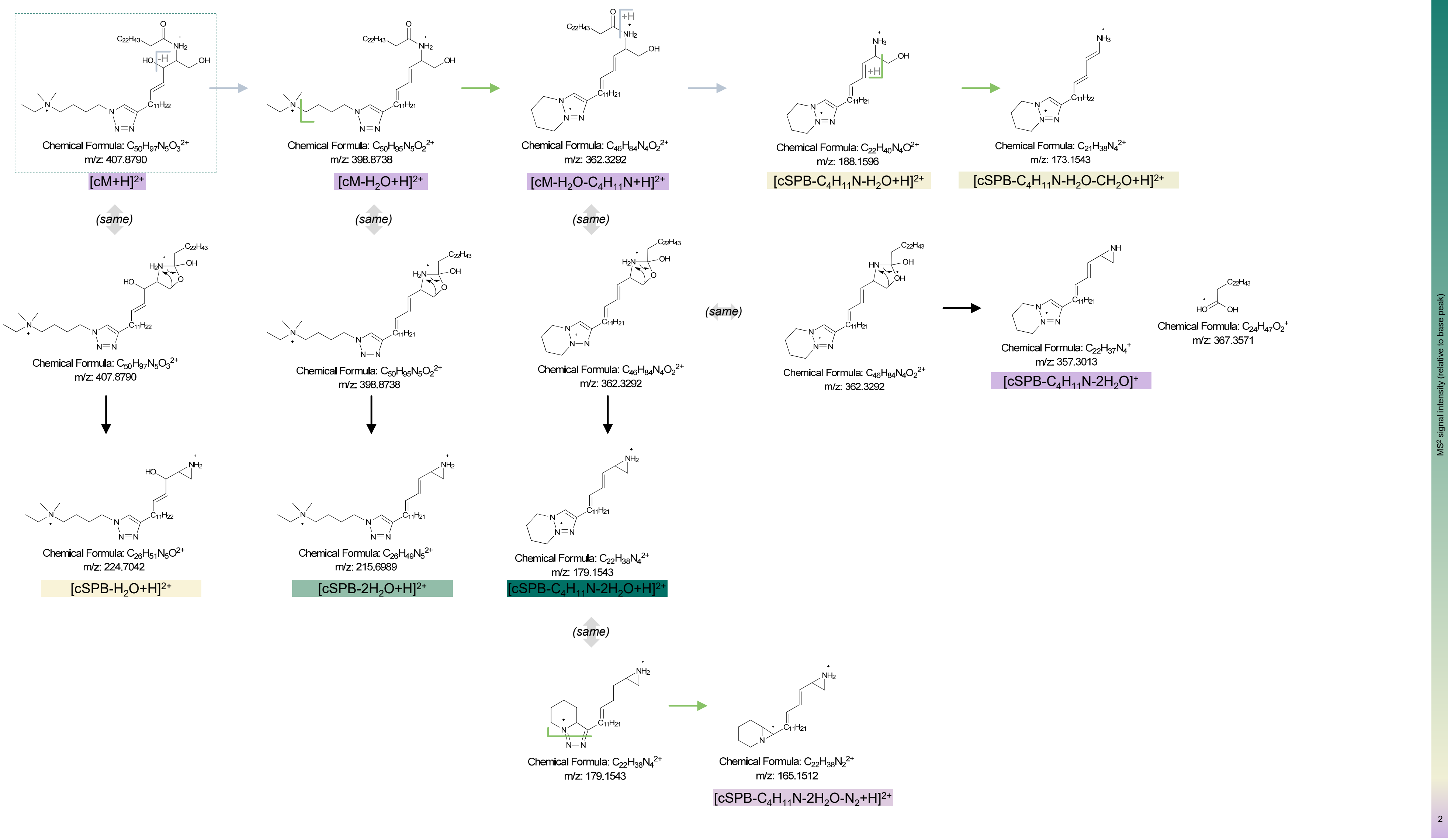

◂ Back to Content

Cer 18:1;O2,C171/24:0;C171 491.9478 – $C_{58}H_{113}O_3N_9^{2+}$

Extracted Ion Chromatogram

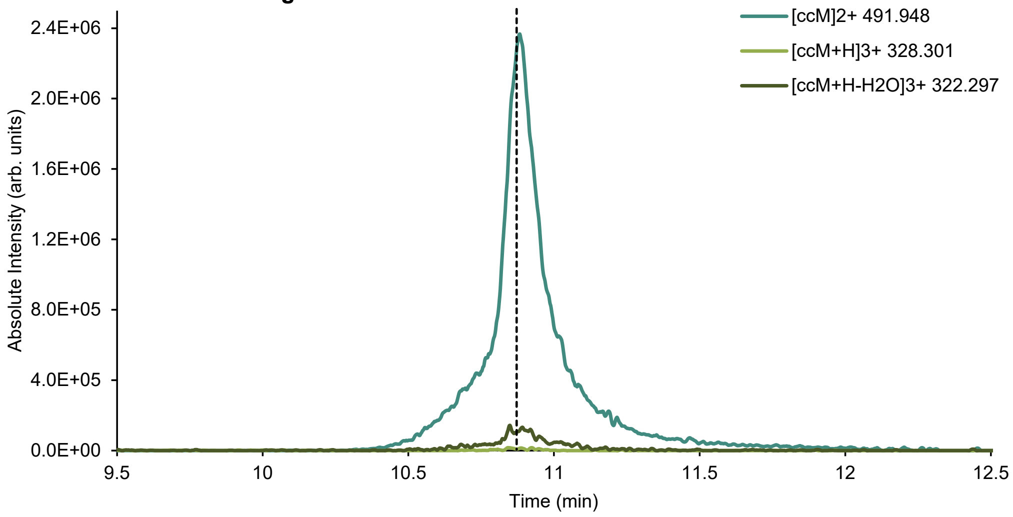

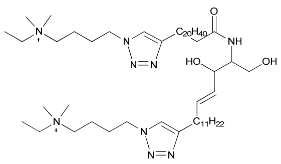

MS² [ccM]²⁺

Ex_24_32_PN27_S3_aP_DDA_1 #6920 RT: 10.87 AV: 1 NL: 2.12E6
T: FTMS + p ESI d Full ms2 491.9474@hcd37.00 [102.4797-1024.7968]

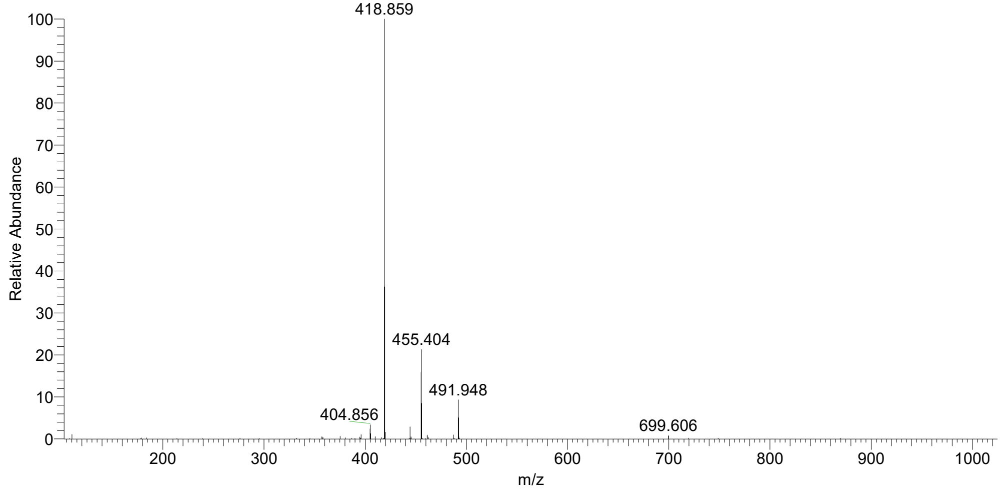

◂ Back to Content

Cer 18:1;O2,C171/24:0;C171 491.9478 – $C_{58}H_{113}O_3N_9^{2+}$ proposed fragmentation scheme

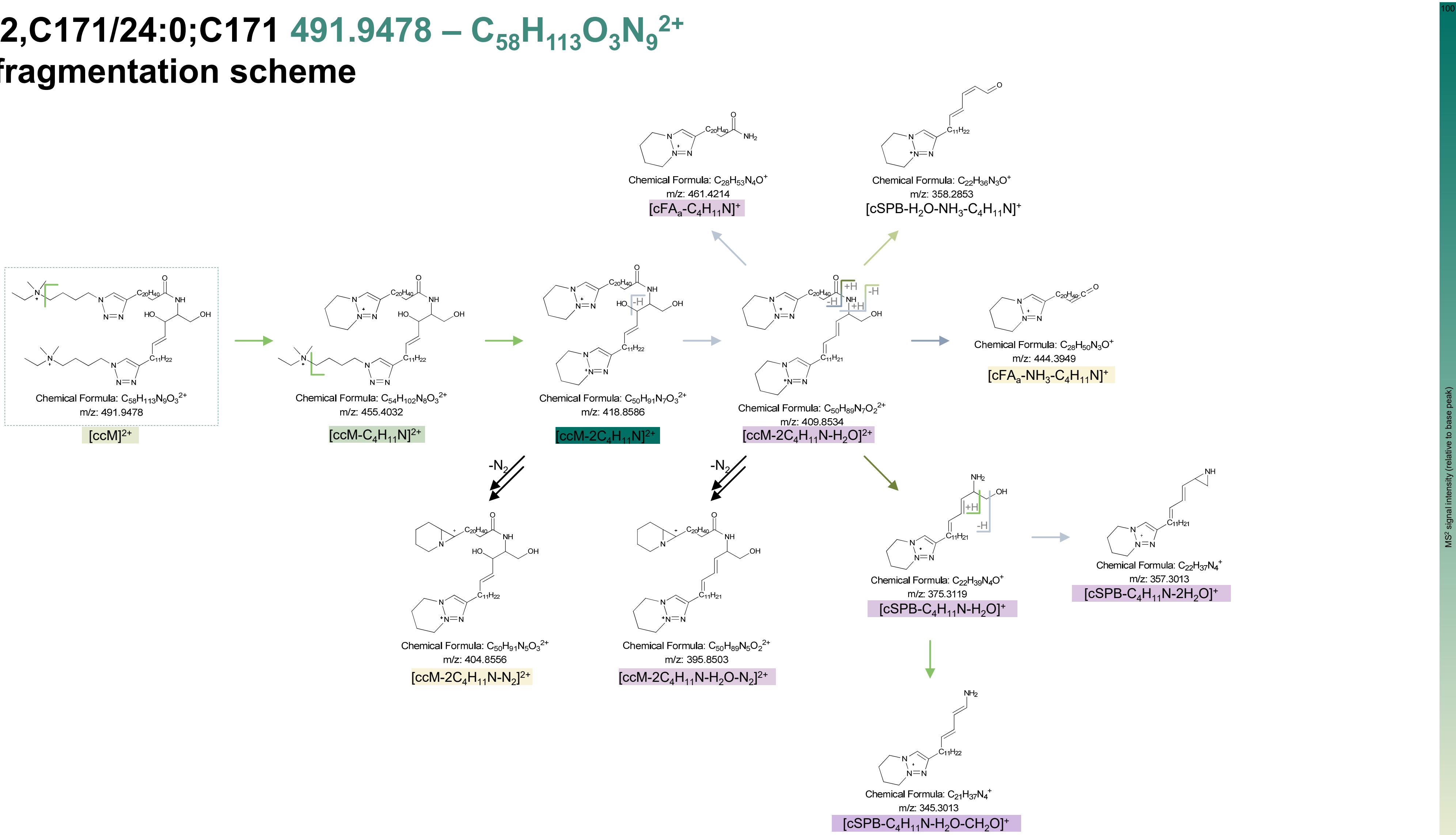

◂ Back to Content

HexCer 18:1;O2/24:1;C171 976.8036 – $C_{56}H_{106}O_8N_5^+$ / 488.9054 – $C_{56}H_{107}O_8N_5^{2+}$

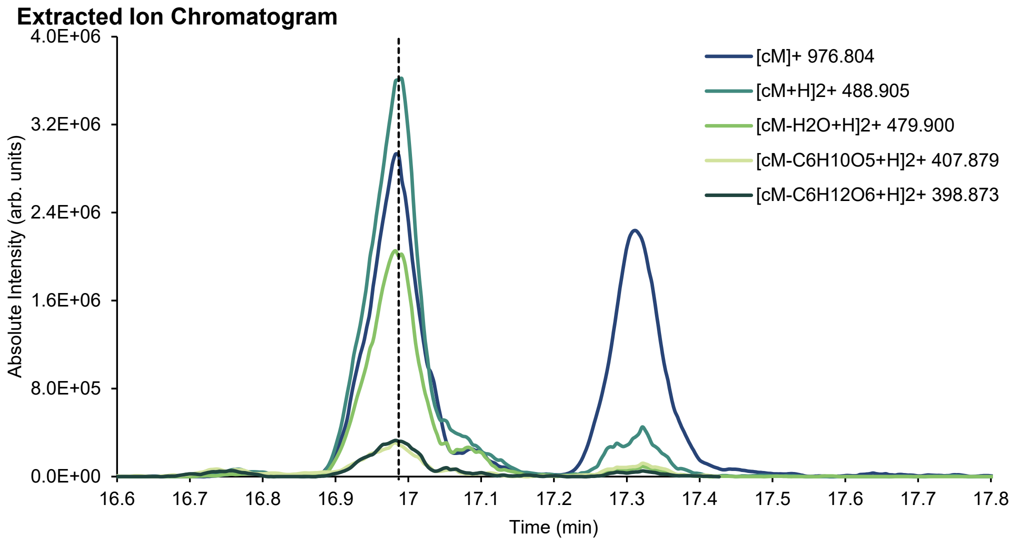

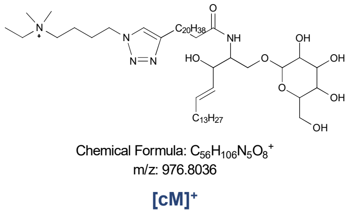

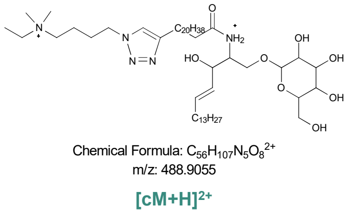

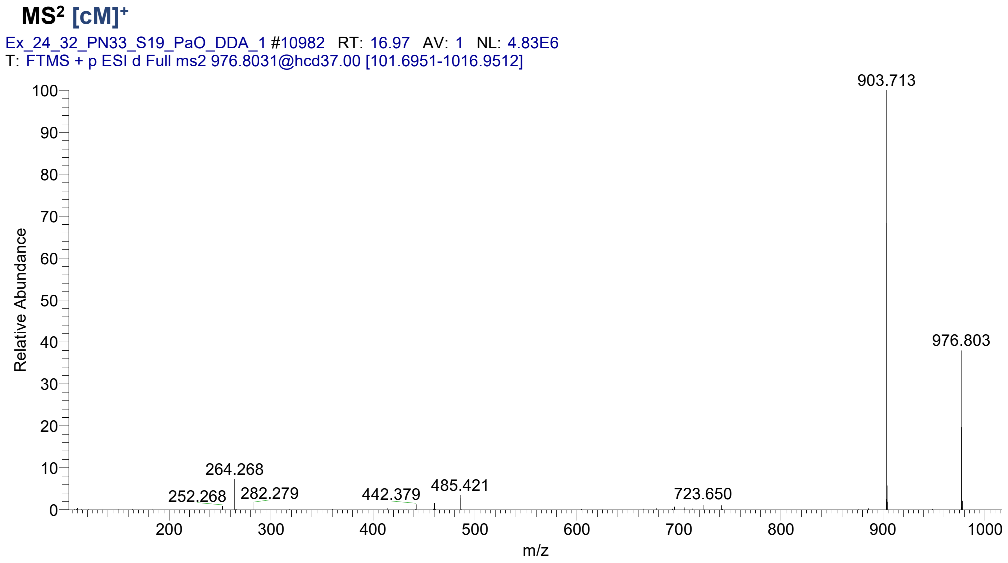

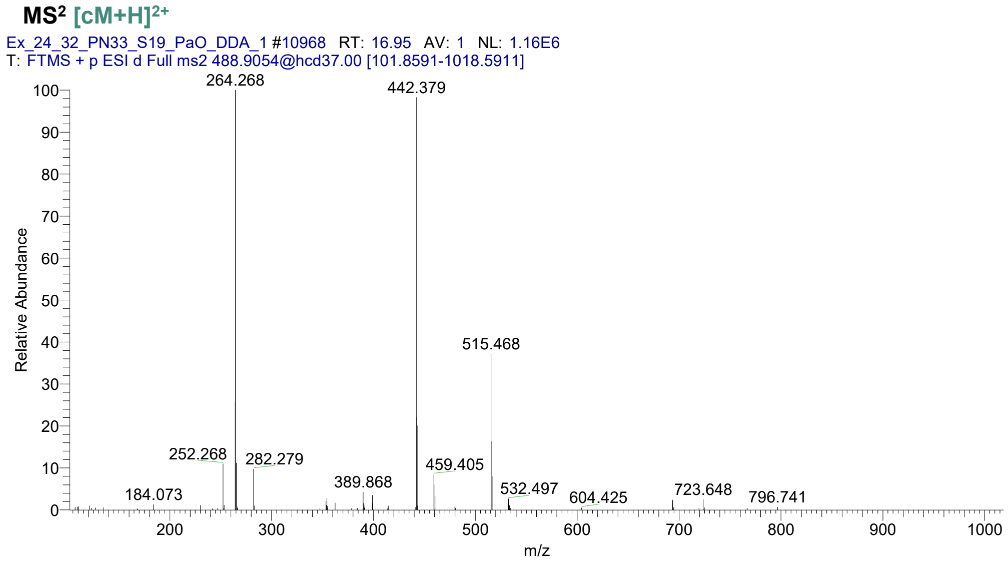

◂ Back to Content

HexCer 18:1;O2/24:1;C171 976.8036 – $C_{56}H_{106}O_8N_5^+$ proposed fragmentation scheme

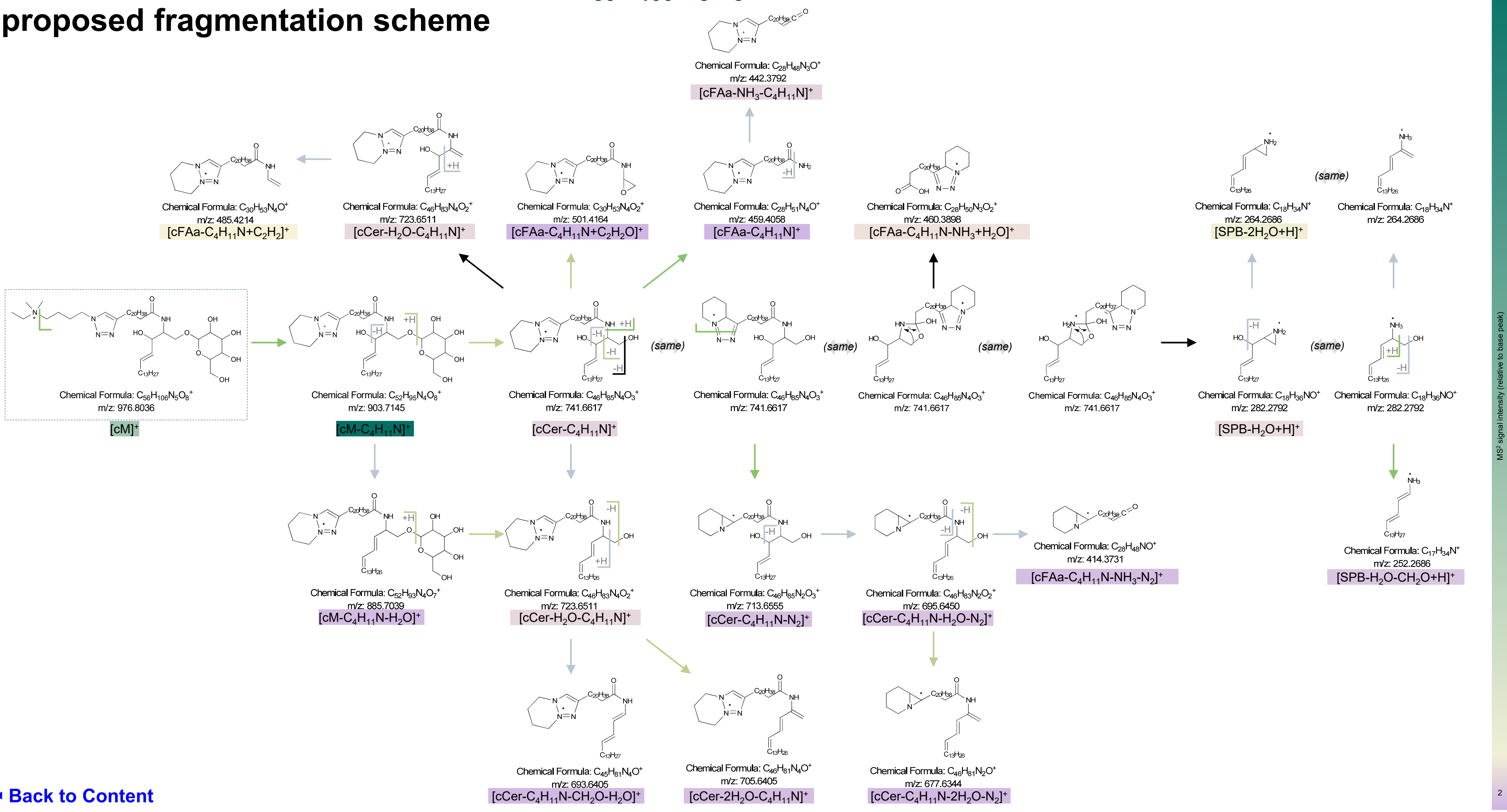

◂ Back to Content

HexCer 18:1;O2/24:1;C171 488.9054 – $C_{56}H_{107}O_8N_5^{2+}$ proposed fragmentation scheme

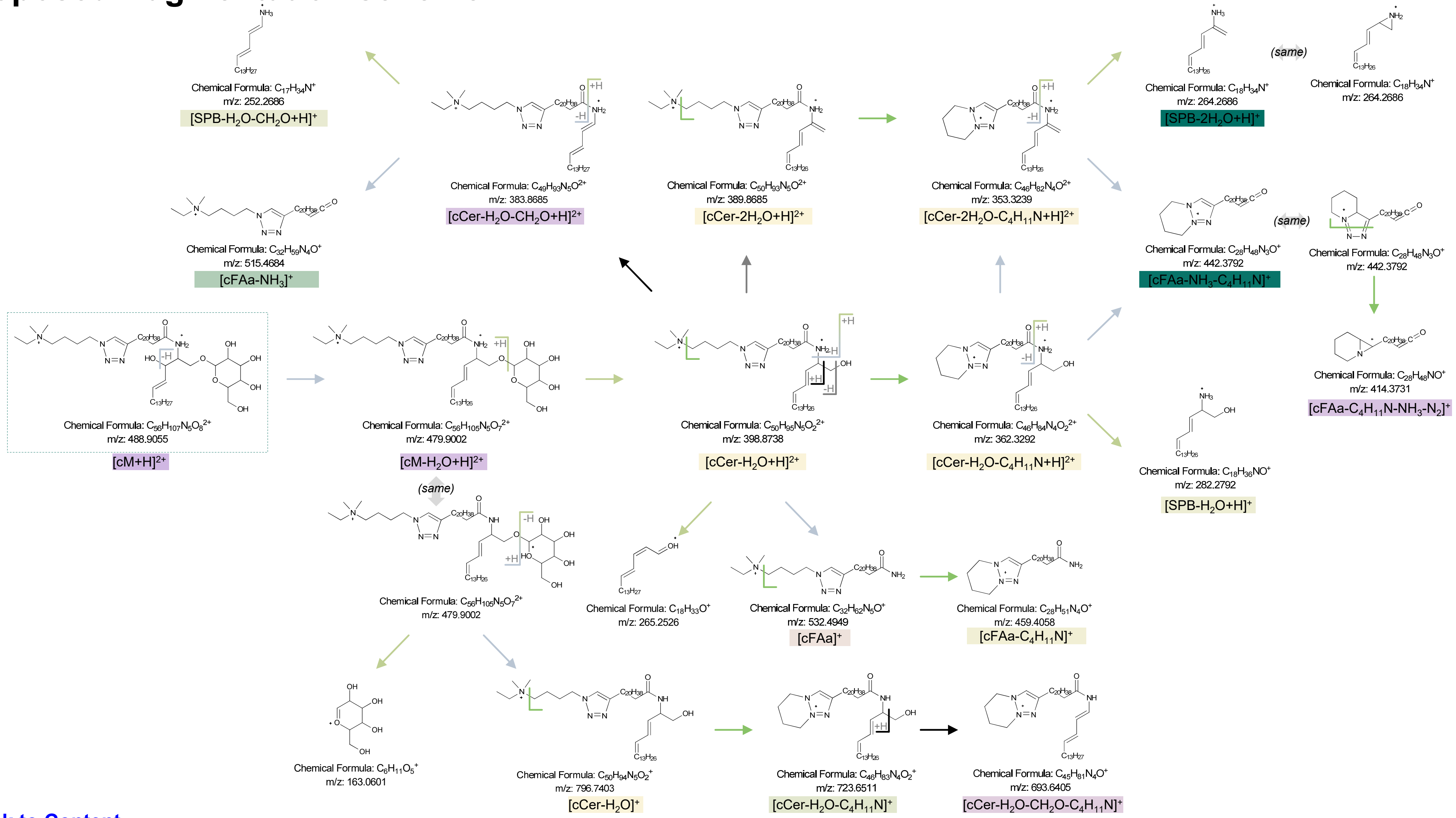

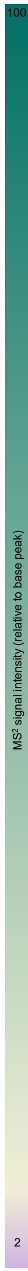

◂ Back to Content

HexCer 18:1;O2,C171/24:1 976.8036 – $C_{56}H_{106}O_8N_5^+$ / 488.9054 – $C_{56}H_{107}O_8N_5^{2+}$

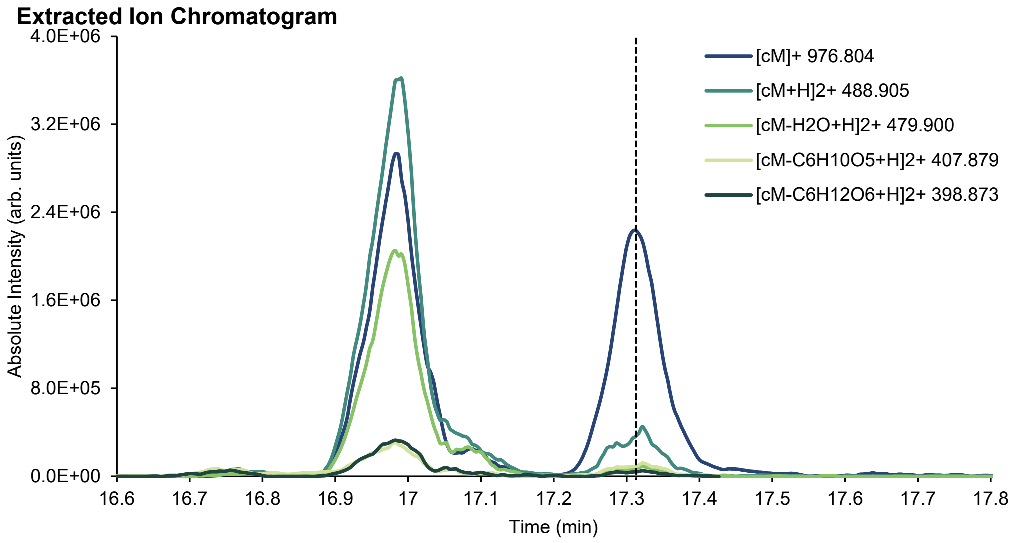

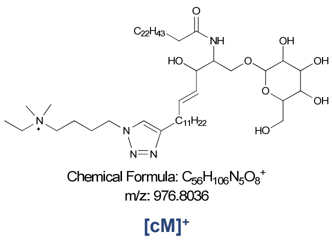

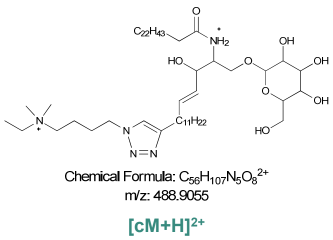

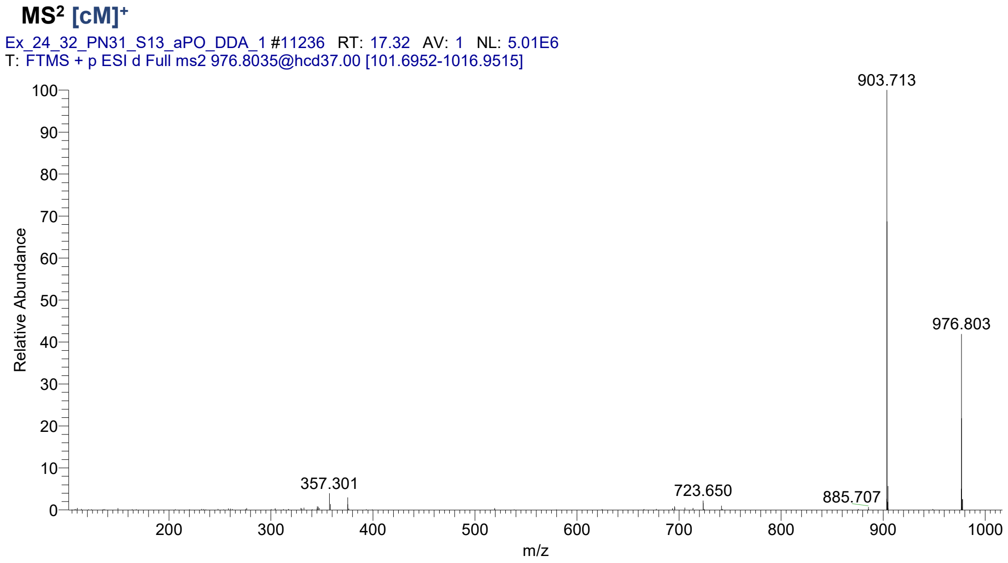

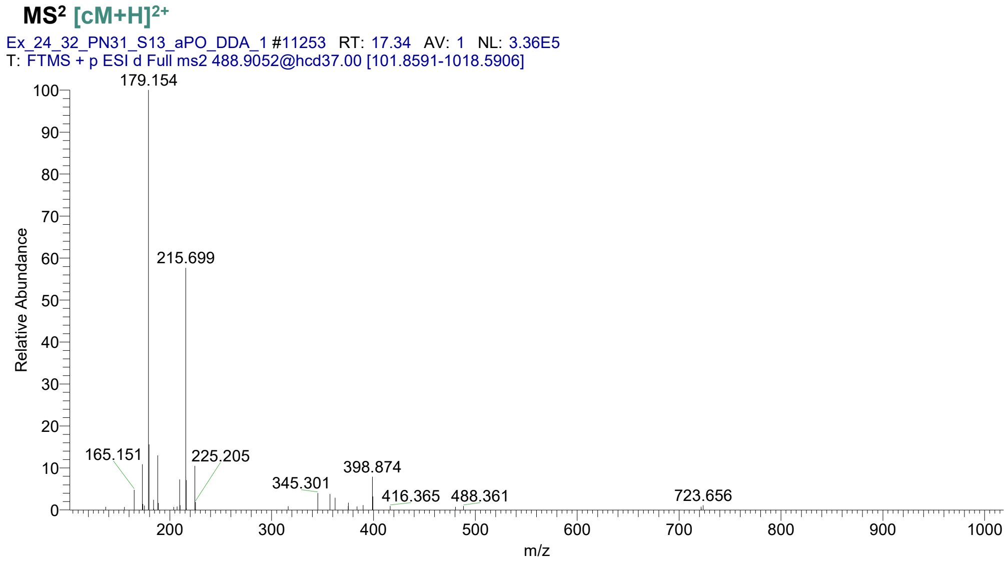

◂ Back to Content

HexCer 18:1;O2,C171/24:1 976.8036 – $C_{56}H_{106}O_8N_5^+$ proposed fragmentation scheme

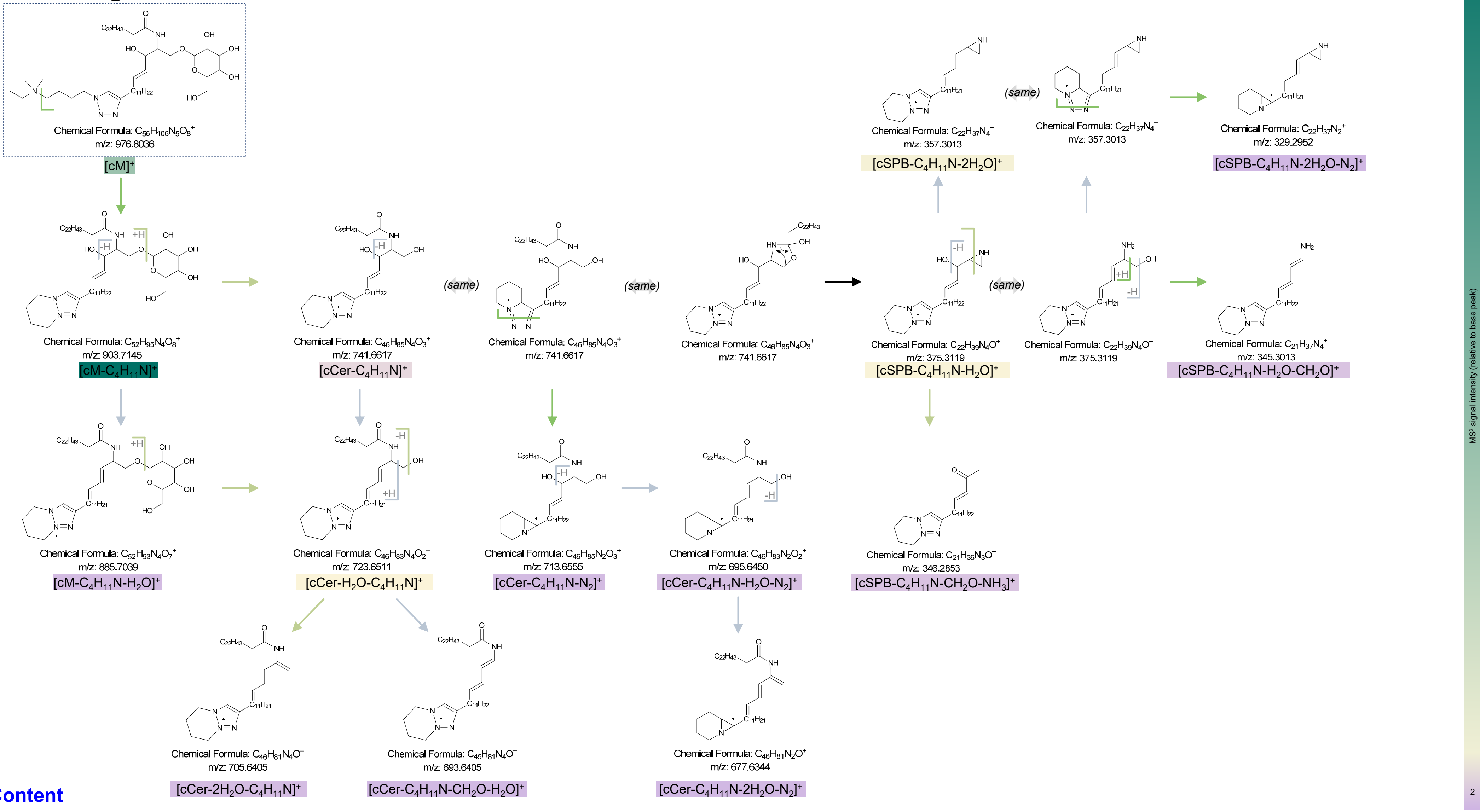

◂ Back to Content

HexCer 18:1;O2,C171/24:1 488.9054 – $C_{56}H_{107}O_8N_5^{2+}$ proposed fragmentation scheme

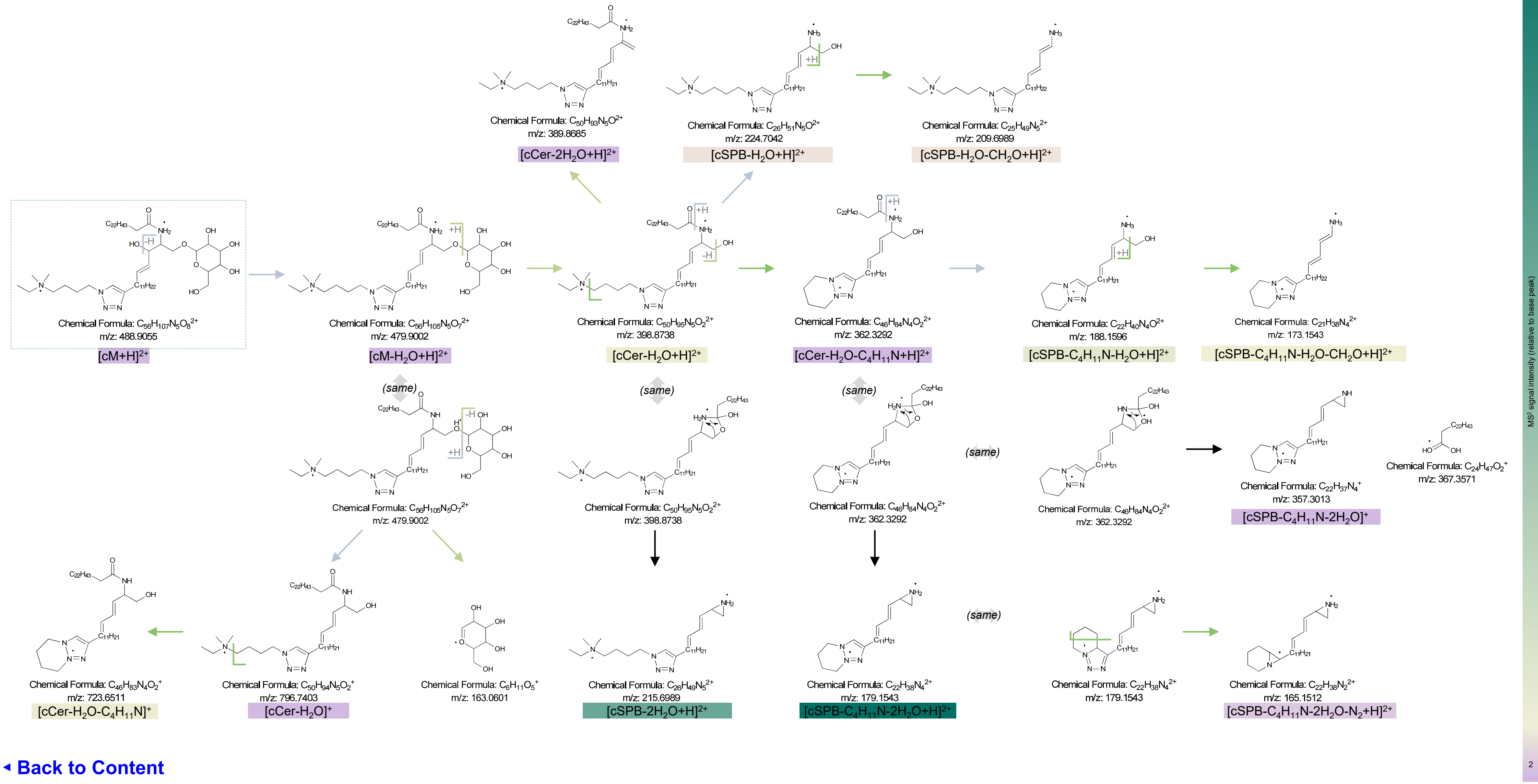

◂ Back to Content

HexCer 18:1;O2,C171/24:0;C171 572.9742 – $C_{64}H_{123}O_8N_9^{2+}$

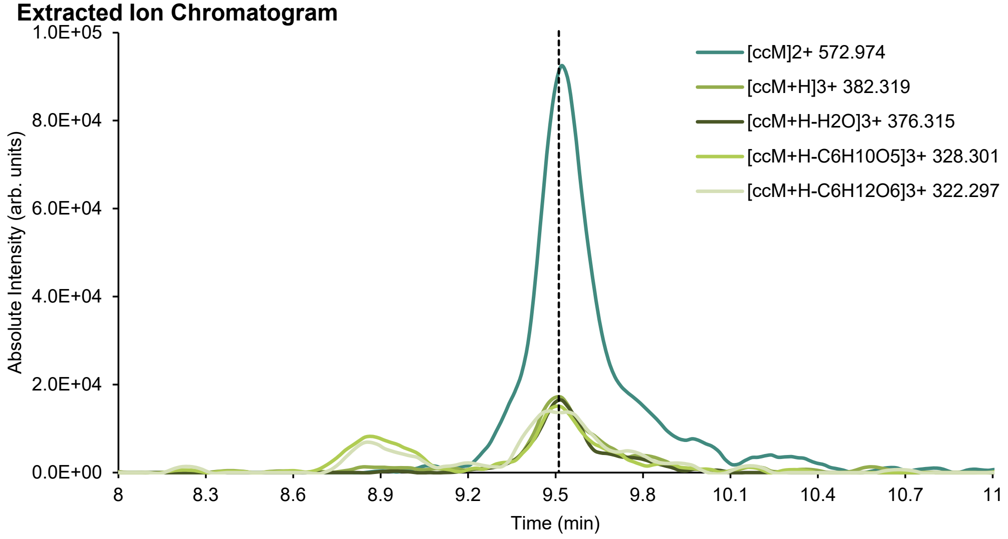

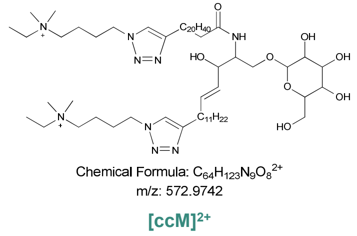

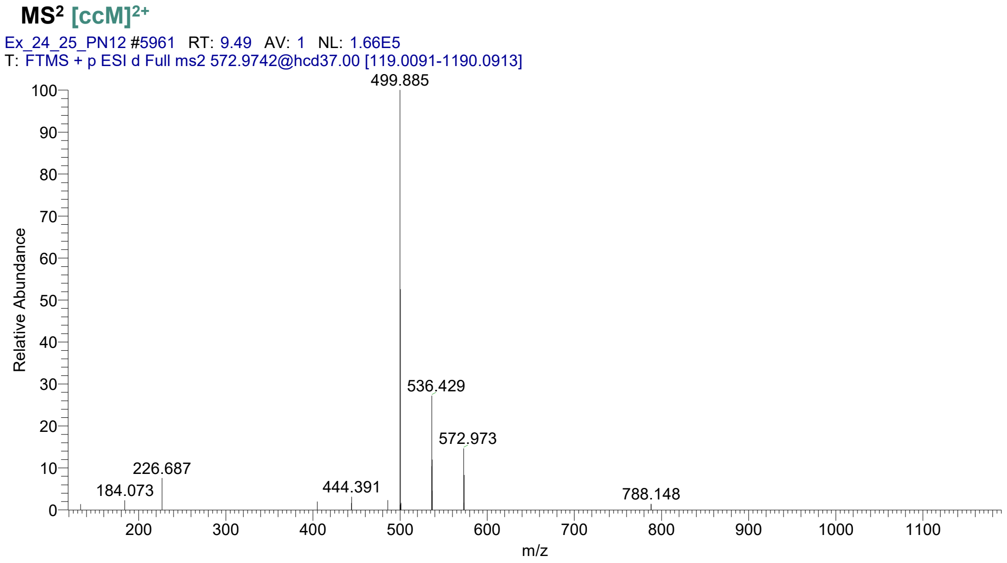

◂ Back to Content

HexCer 18:1;O2,C171/24:0;C171 572.9742 – $C_{64}H_{123}O_8N_9^{2+}$ proposed fragmentation scheme

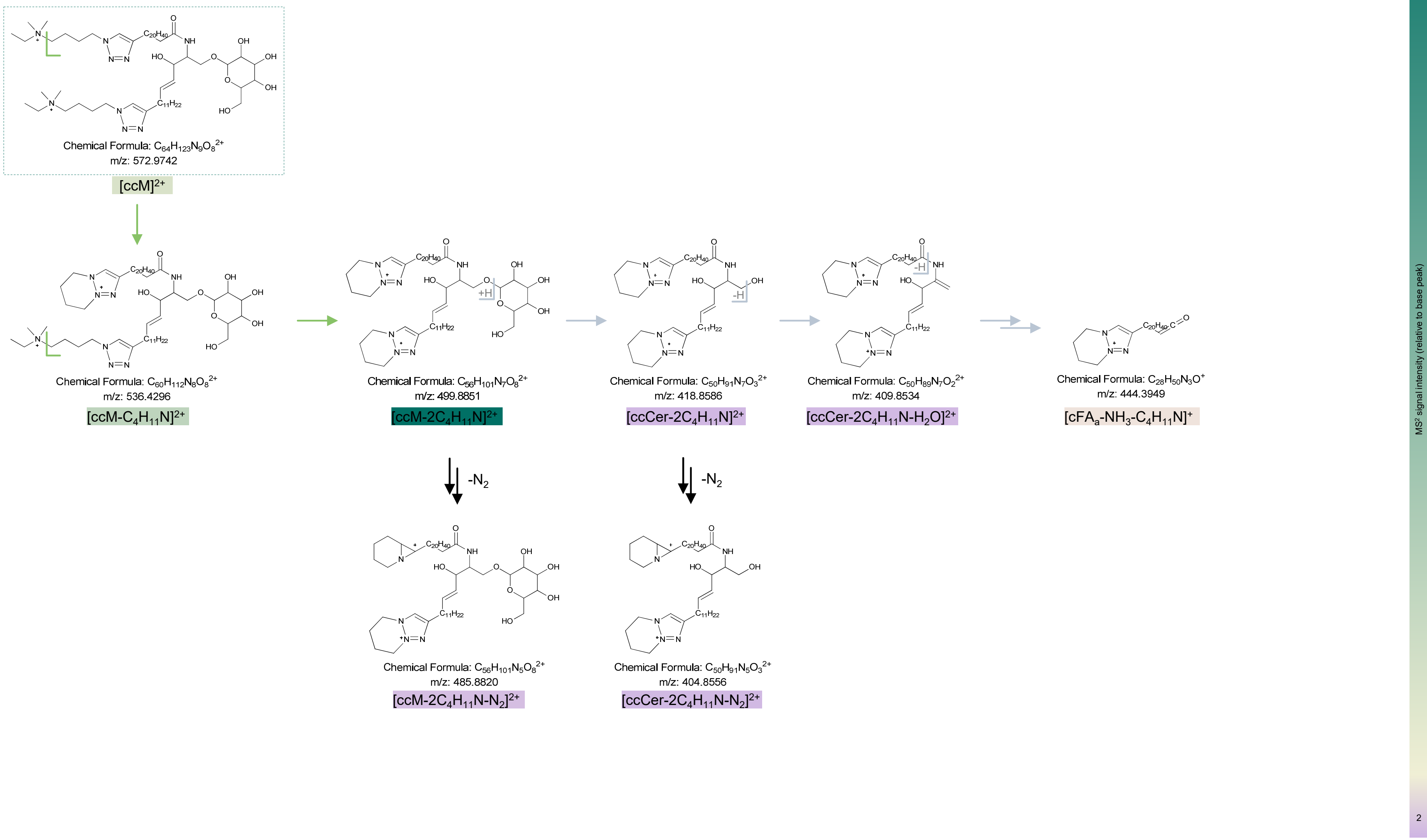

◂ Back to Content

Hex2Cer 18:1;O2/24:1;C171 1138.8564 – $C_{62}H_{116}O_{13}N_5^+$ / 569.9318 – $C_{62}H_{117}O_{13}N_5^{2+}$

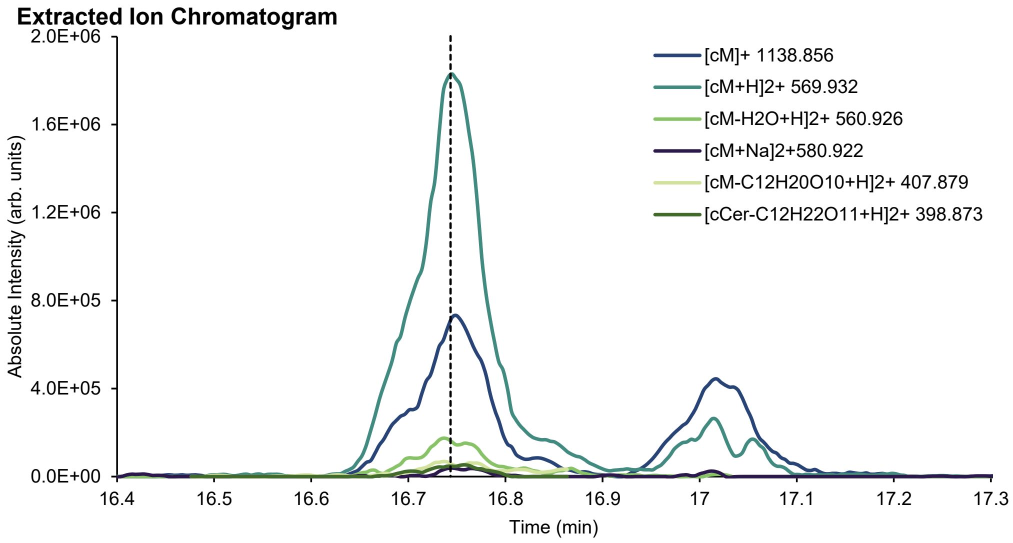

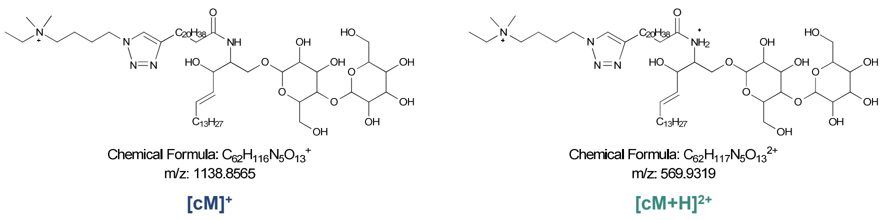

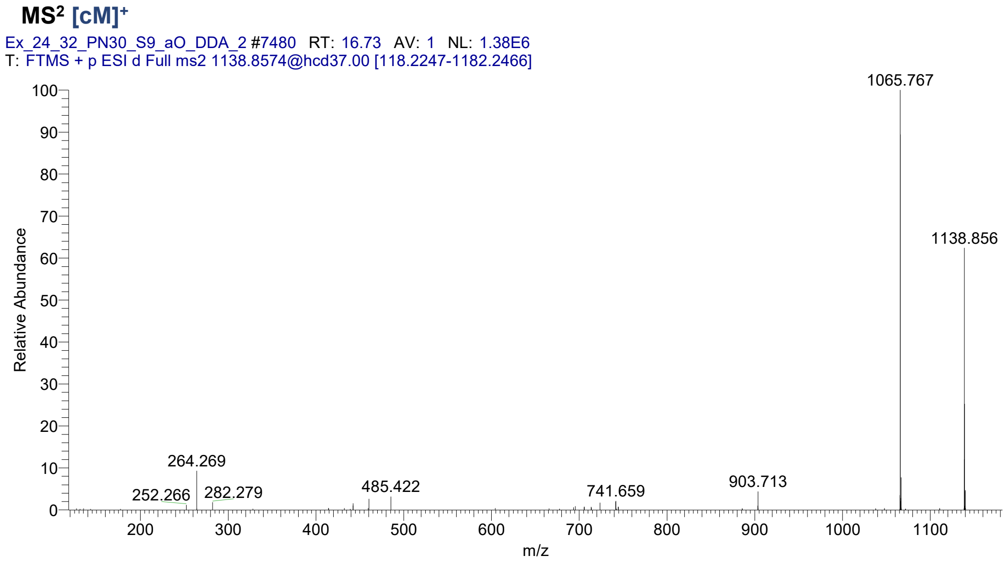

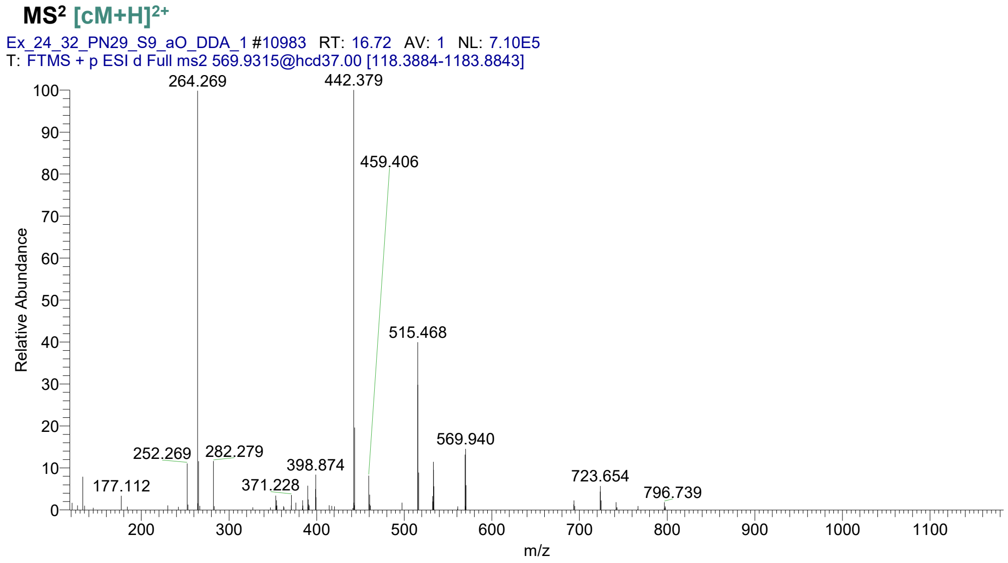

◂ Back to Content

Hex2Cer 18:1;O2/24:1;C171 1138.8564 – $C_{62}H_{116}O_{13}N_5^+$ proposed fragmentation scheme

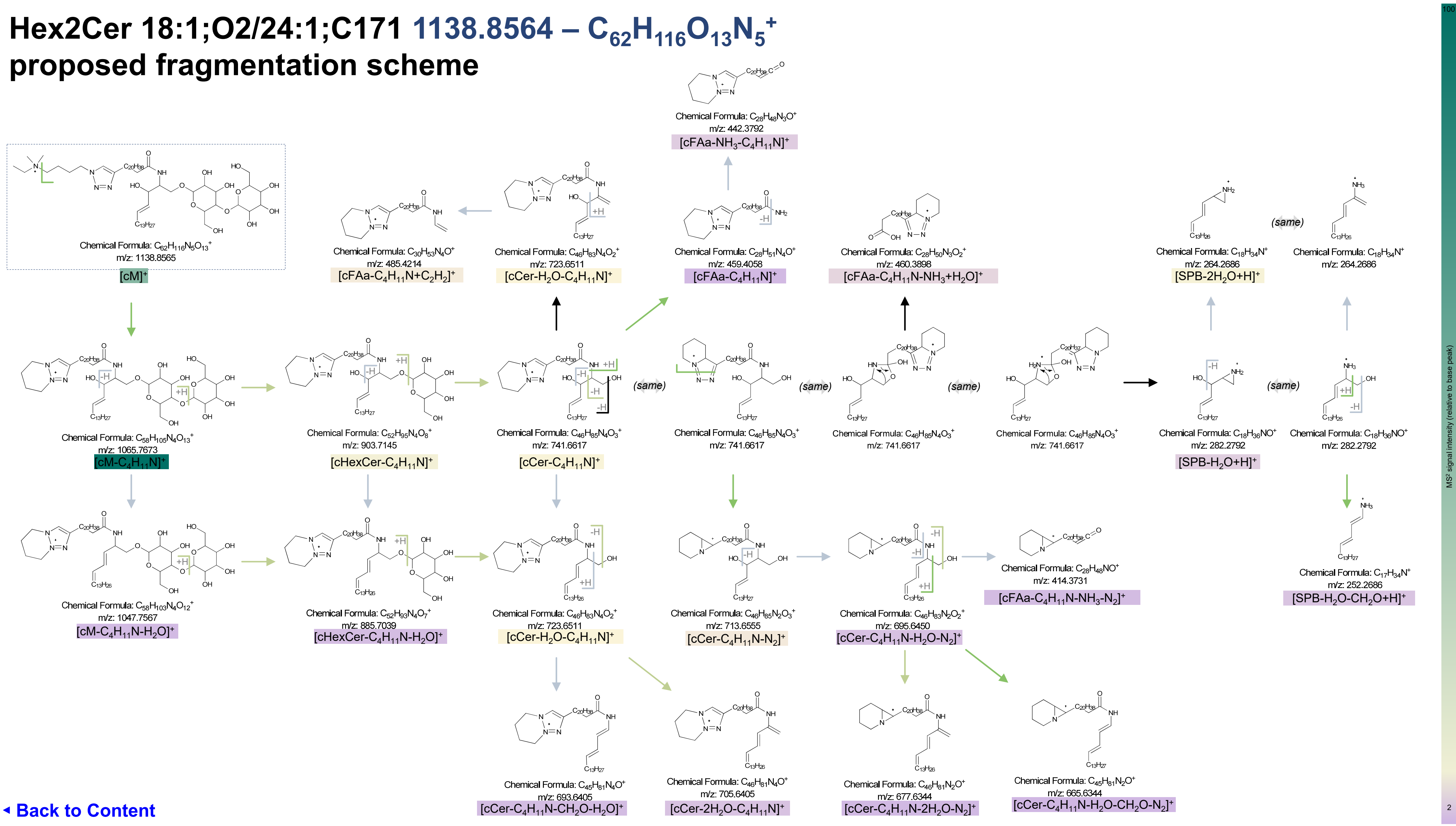

◂ Back to Content

Hex2Cer 18:1;O2/24:1;C171 569.9318 – $C_{62}H_{117}O_{13}N_5^{2+}$ proposed fragmentation scheme

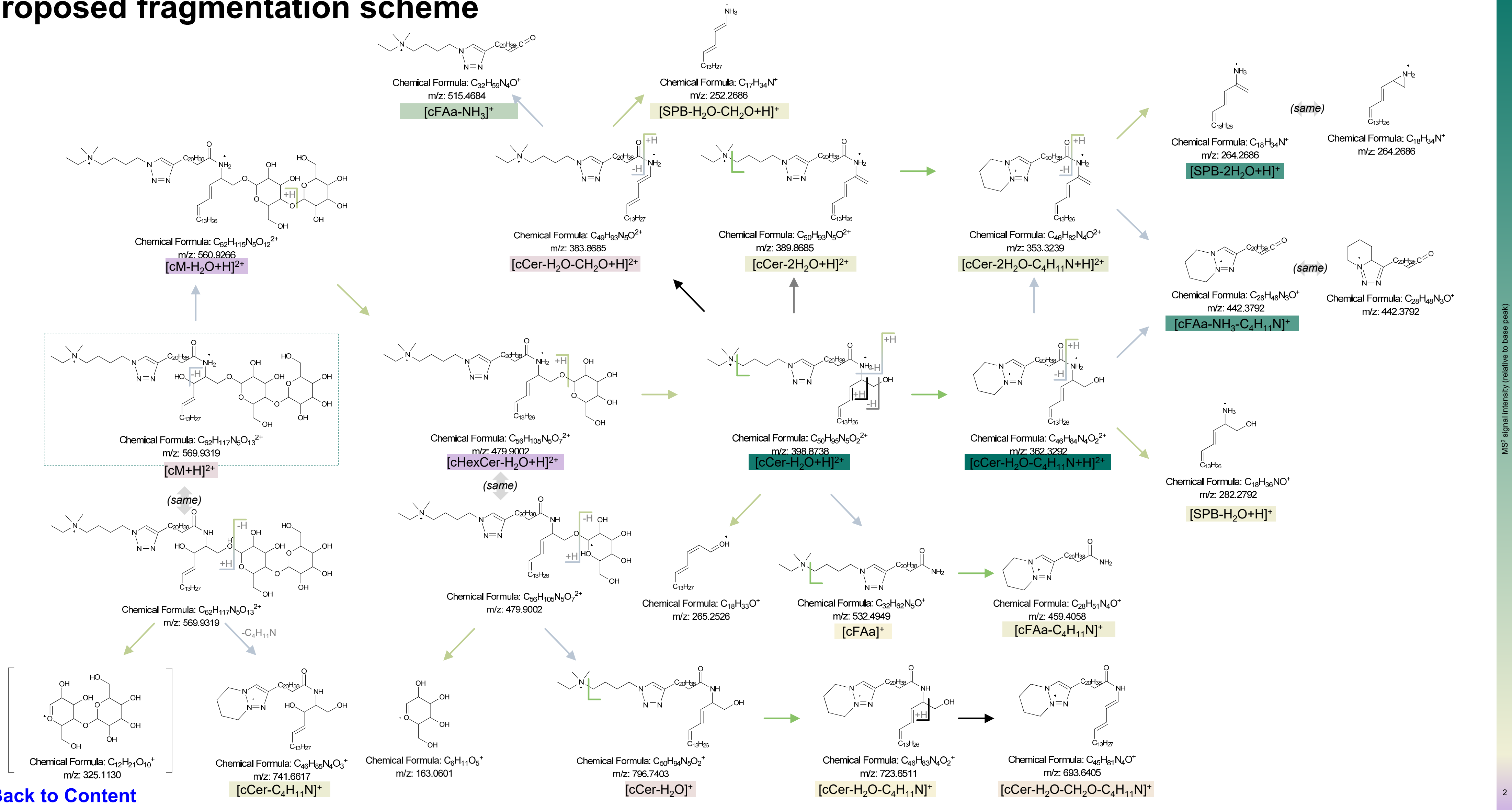

◂ Back to Content

Hex2Cer 18:1;O2,C171/24:1 1138.8564 – $C_{62}H_{116}O_{13}N_5^{+}$ / 569.9318 – $C_{62}H_{117}O_{13}N_5^{2+}$

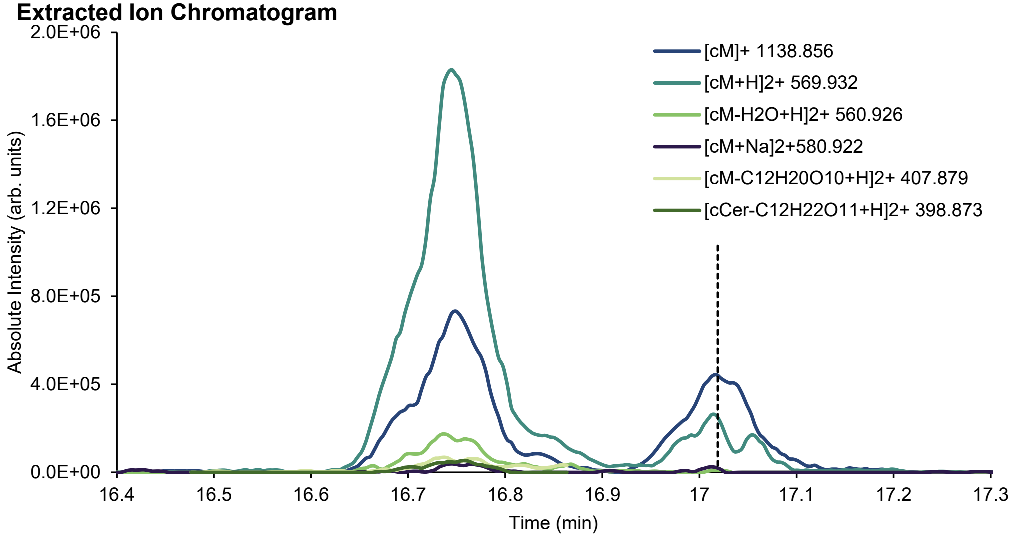

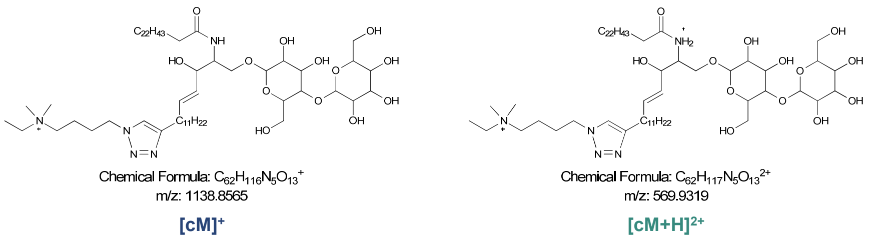

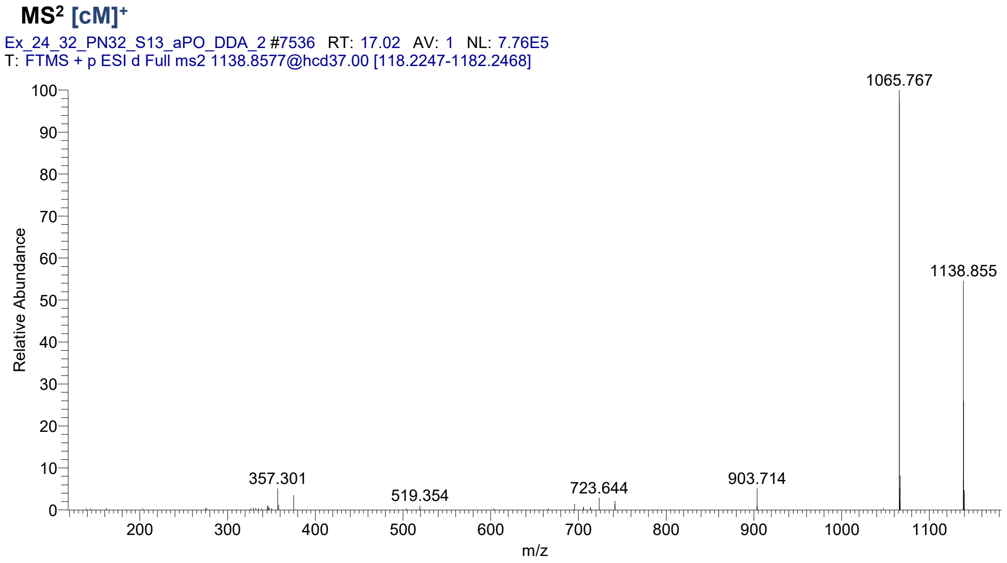

◂ Back to Content

Hex2Cer 18:1;O2,C171/24:1 1138.8564 – $C_{62}H_{116}O_{13}N_5^+$ proposed fragmentation scheme

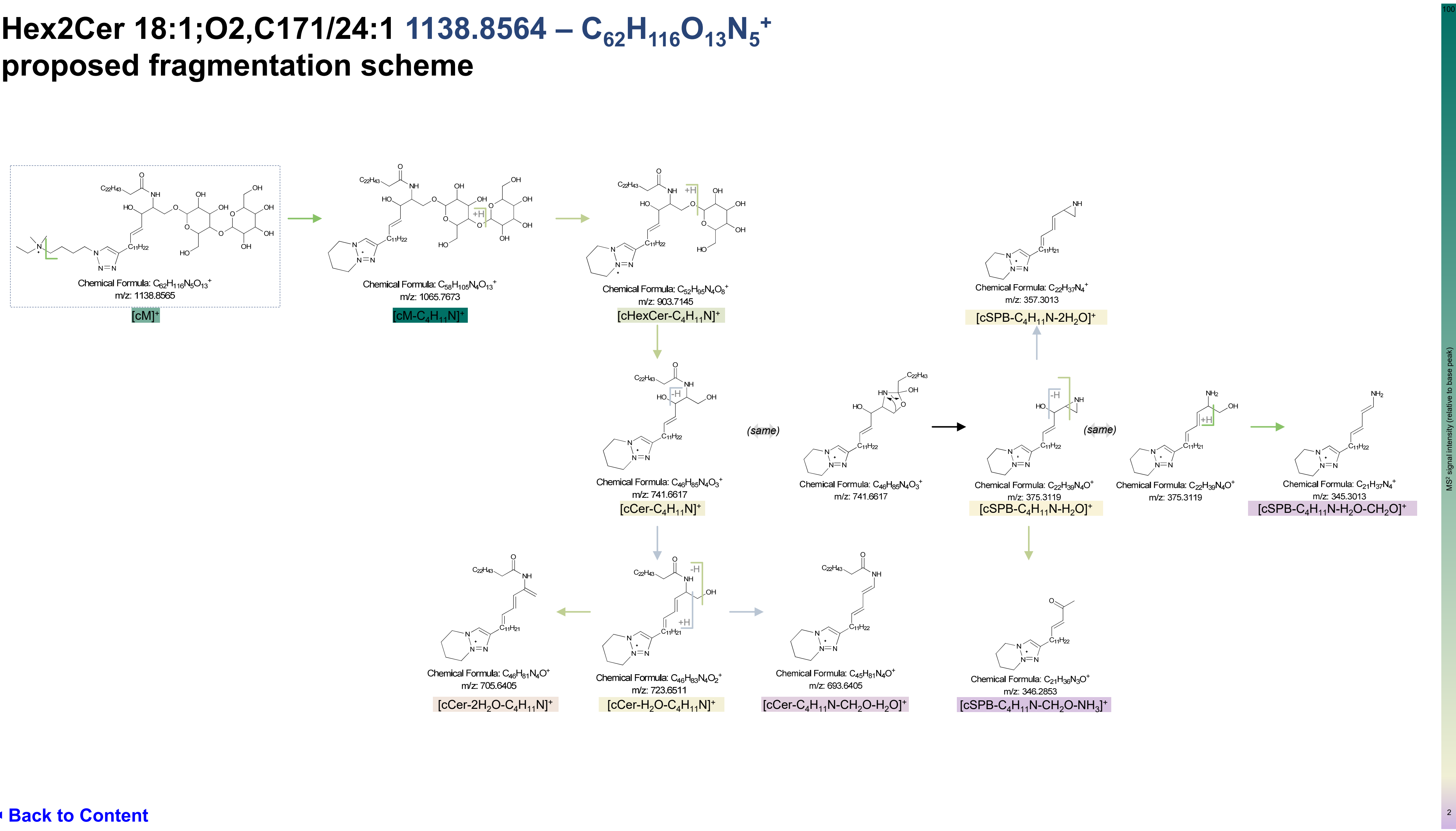

◂ Back to Content

Hex3Cer 18:1;O2/24:1;C171 1300.9093 – $C_{68}H_{126}O_{18}N_5^+$ / 650.9583 – $C_{68}H_{127}O_{18}N_5^{2+}$

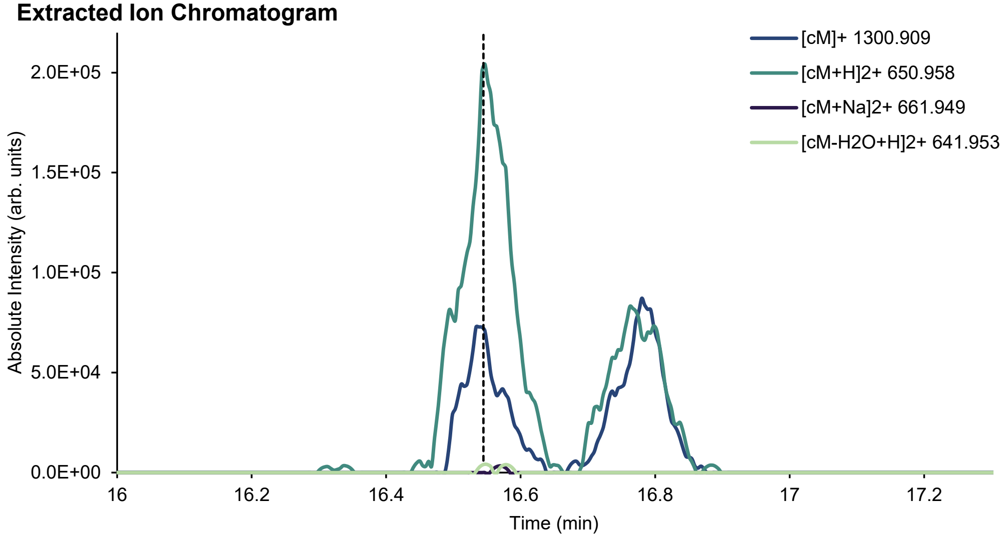

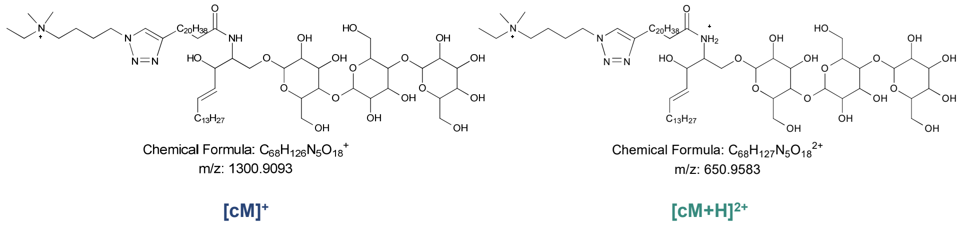

MS² [cM]⁺

Ex_24_32_PN30_S9_aO_DDA_2 #7355 RT: 16.54 AV: 1 NL: 9.73E4
T: FTMS + p ESI d Full ms2 1300.9113@hcd37.00 [134.7542-1347.5415]

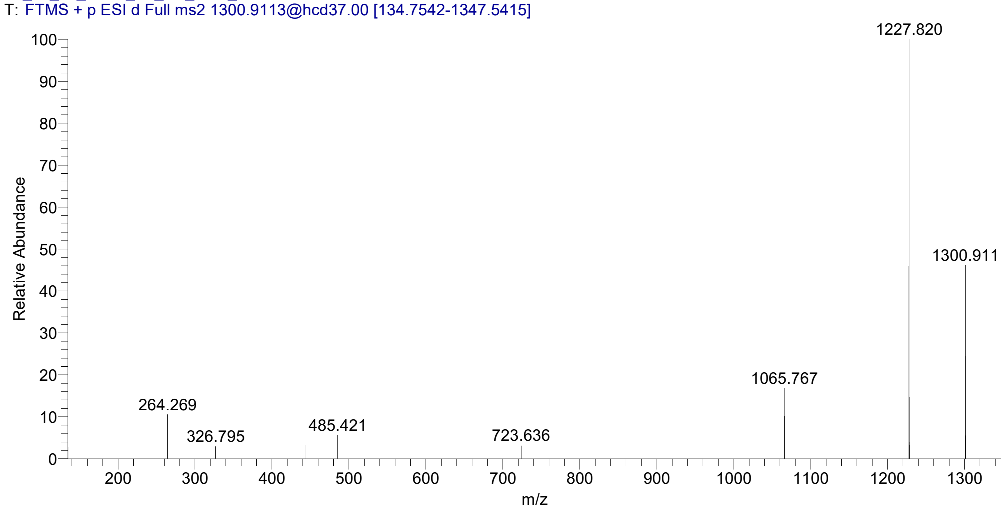

MS² [cM+H]²⁺

Ex_24_32_PN29_S9_aO_DDA_1 #10896 RT: 16.59 AV: 1 NL: 5.63E4
T: FTMS + p ESI d Full ms2 650.9581@hcd37.00 [134.9178-1349.1786]

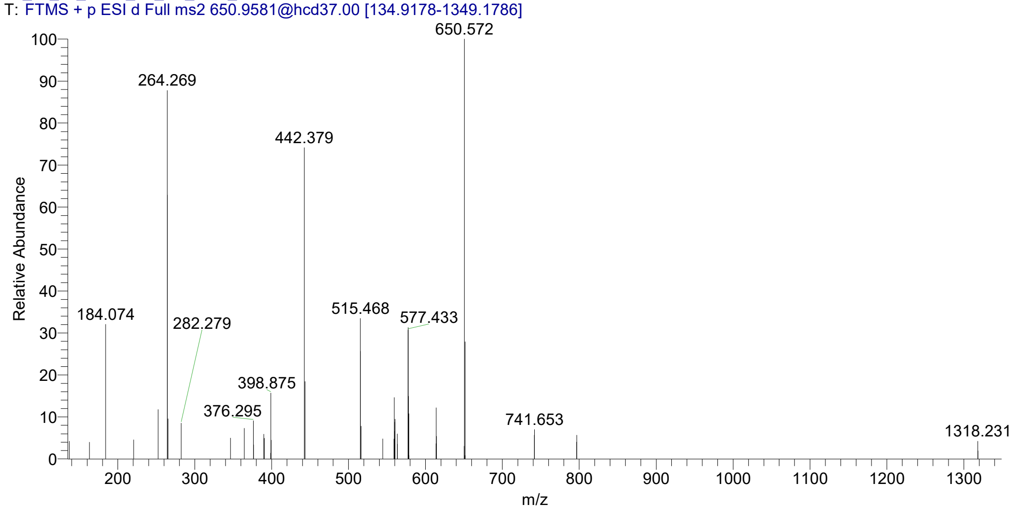

◂ Back to Content

Hex3Cer 18:1;O2/24:1;C171 1300.9093 – $C_{68}H_{126}O_{18}N_5^+$ proposed fragmentation scheme

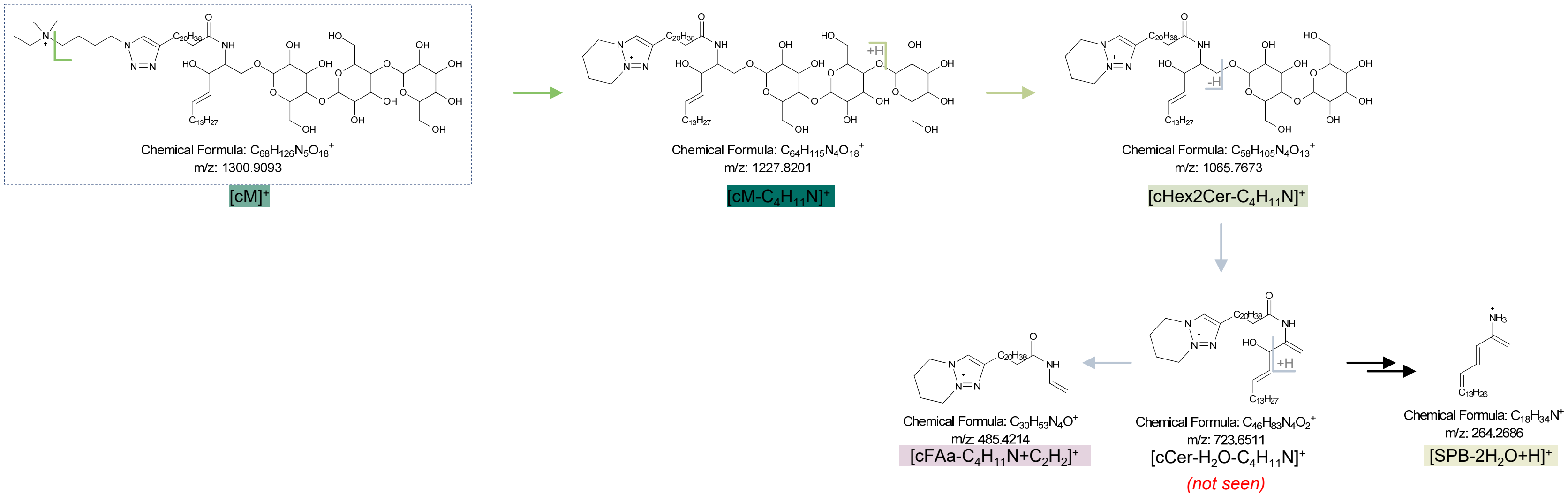

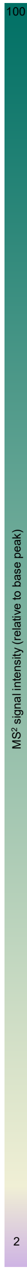

◂ Back to Content

Hex3Cer 18:1;O2/24:1;C171 650.9583 – $C_{68}H_{127}O_{18}N_5^{2+}$ proposed fragmentation scheme

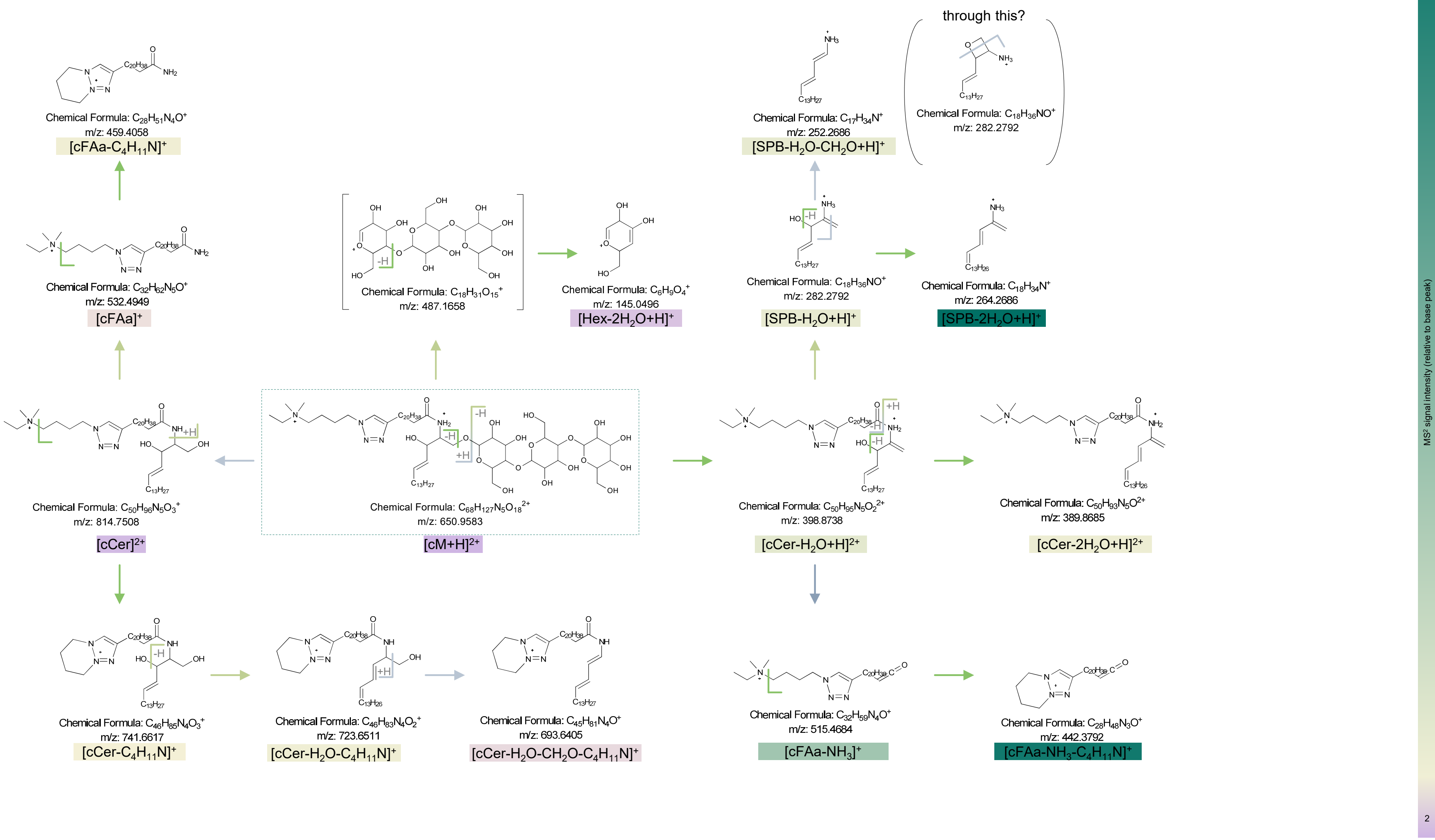

◂ Back to Content

Hex3Cer 18:1;O2,C171/24:1 1300.9093 – $C_{68}H_{126}O_{18}N_5^+$ / 650.9583 – $C_{68}H_{127}O_{18}N_5^{2+}$

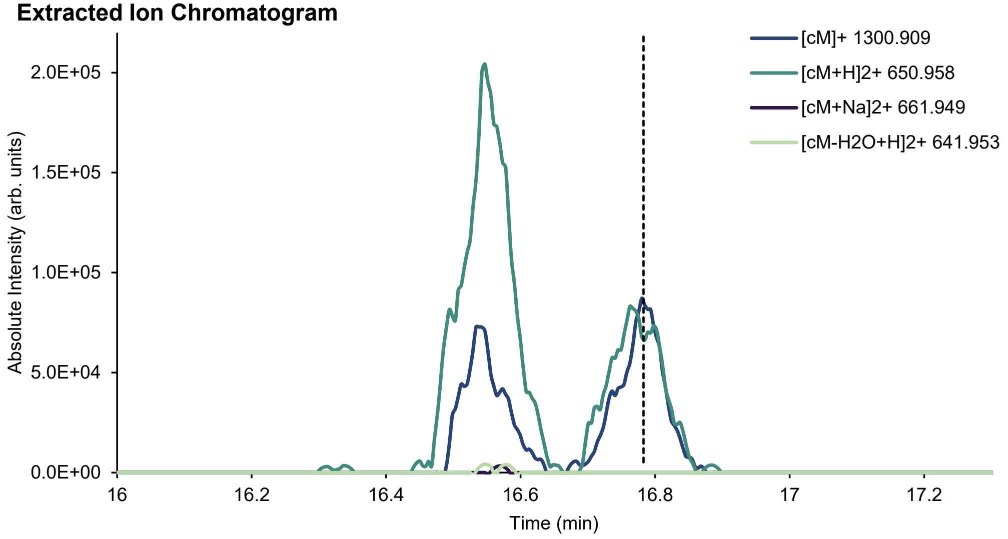

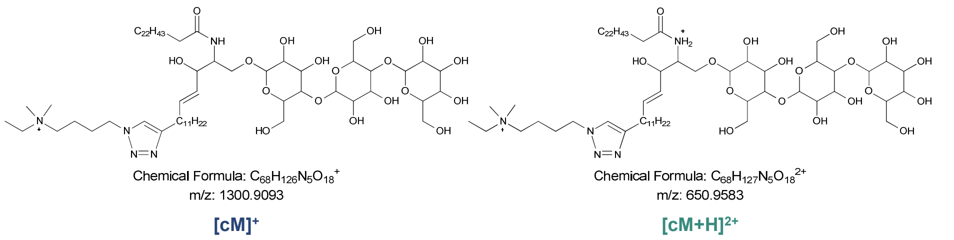

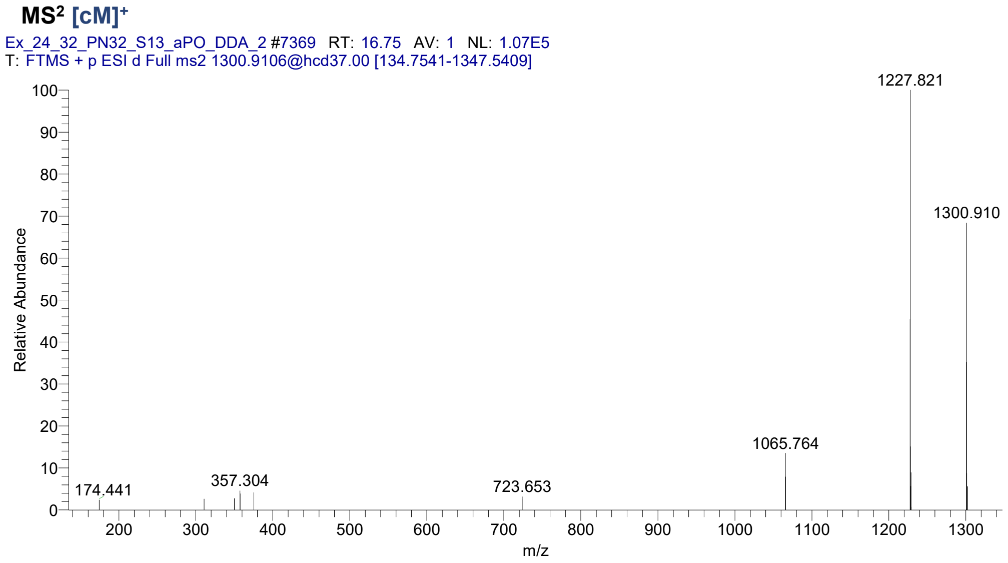

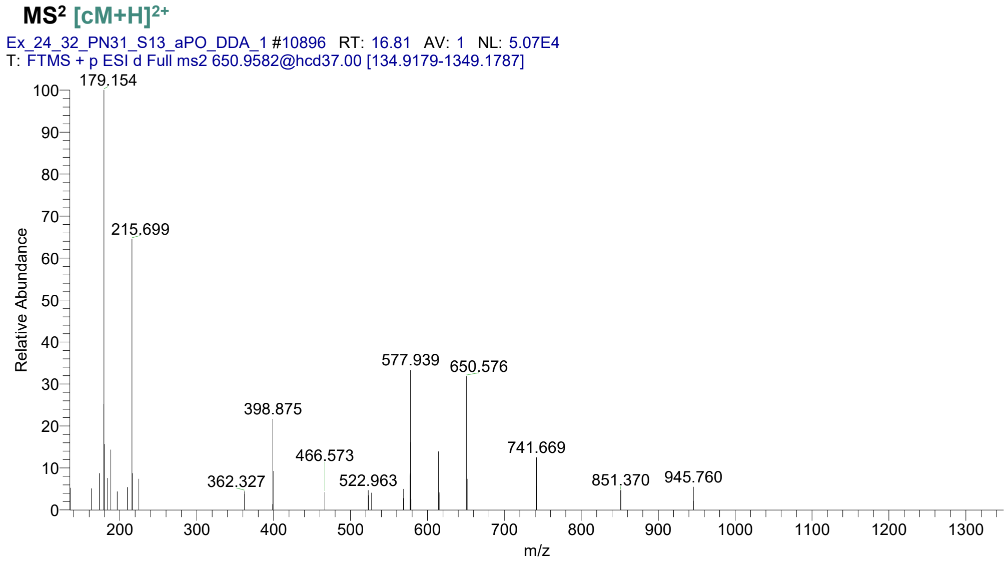

◂ Back to Content

Hex3Cer 18:1;O2,C171/24:1 1300.9093 – $C_{68}H_{126}O_{18}N_5^+$
proposed fragmentation scheme

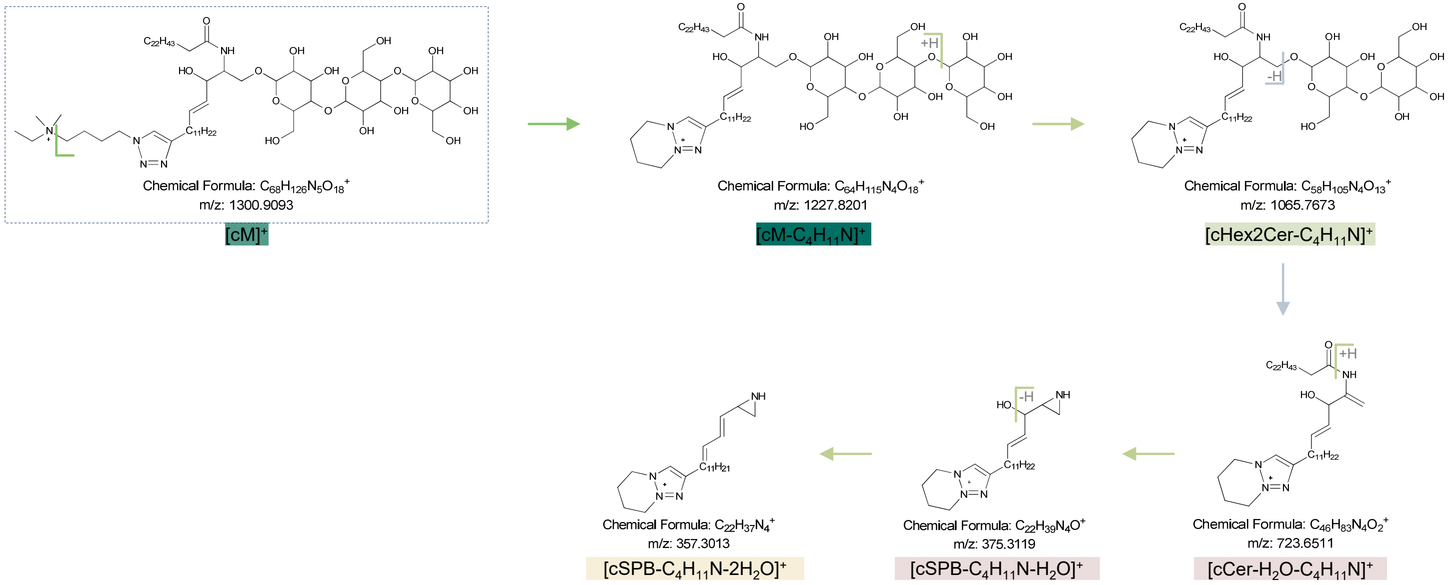

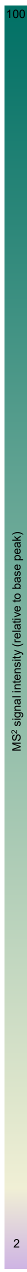

◂ Back to Content

Hex3Cer 18:1;O2,C171/24:1 650.9583 – $C_{68}H_{127}O_{18}N_5^{2+}$
proposed fragmentation scheme

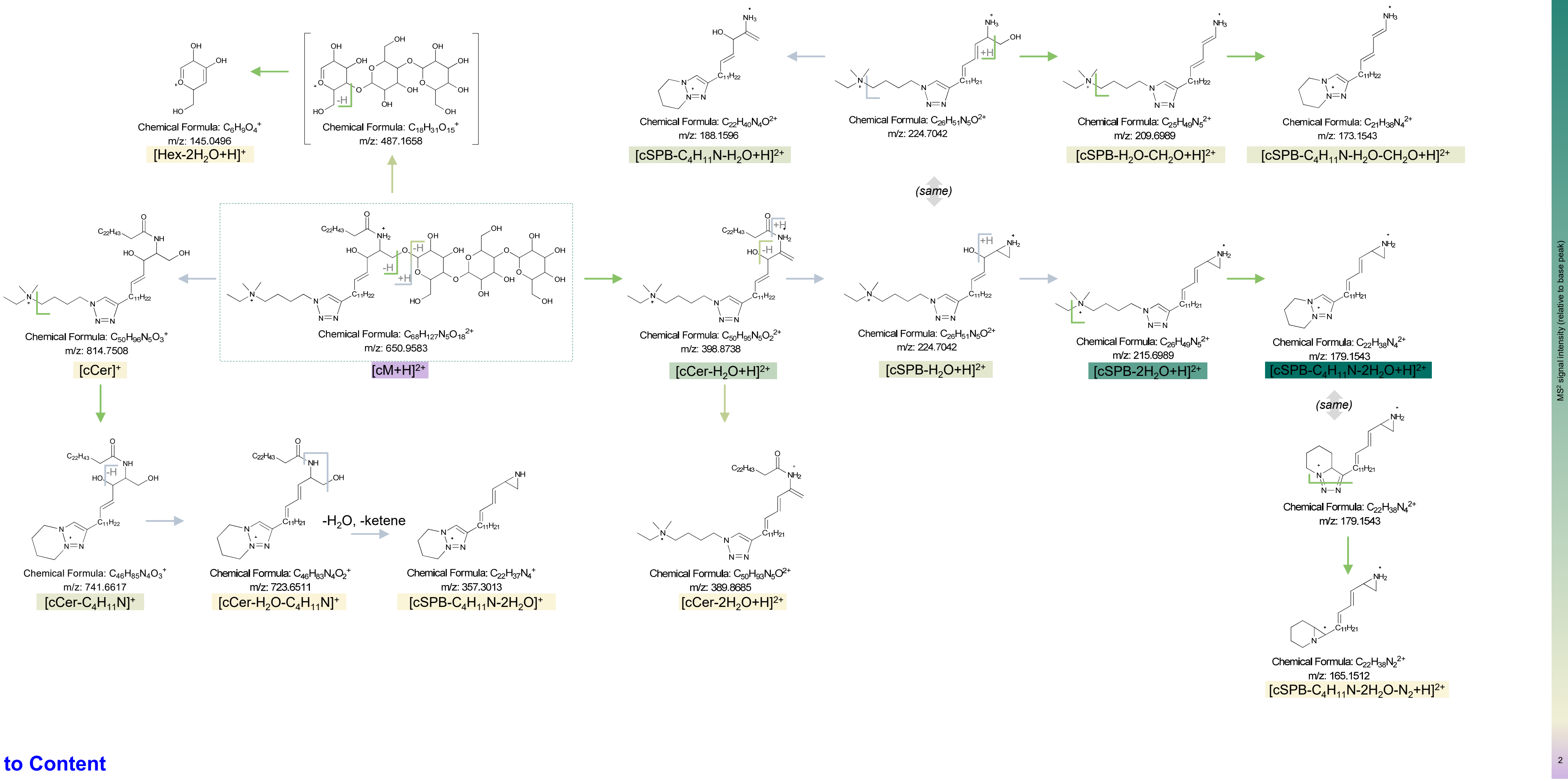

◂ Back to Content

HexNAc-Hex3Cer 18:1;O2/24:1;C171 1503.9886 – $C_{76}H_{139}O_{23}N_6^{+}$ / 752.4980 – $C_{76}H_{140}O_{23}N_6^{2+}$

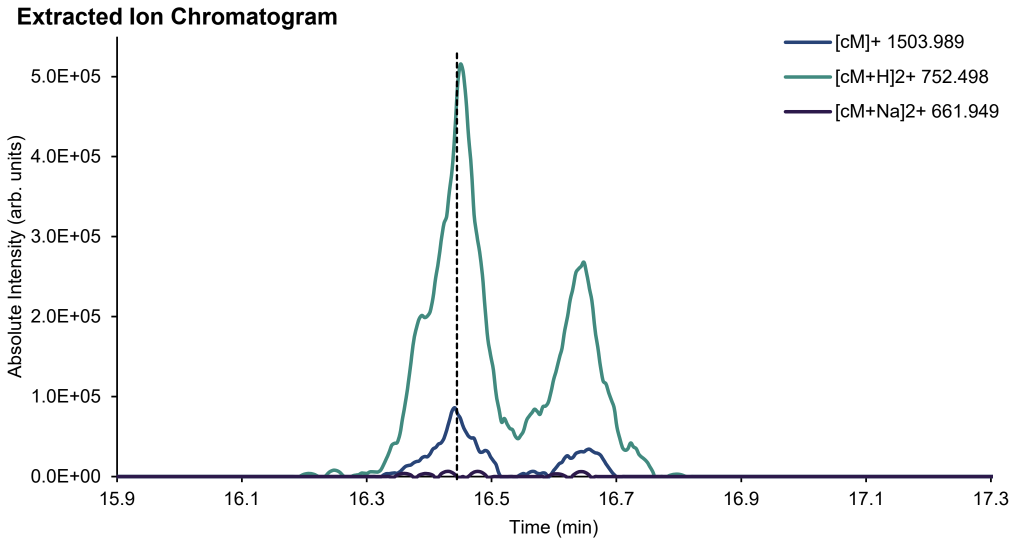

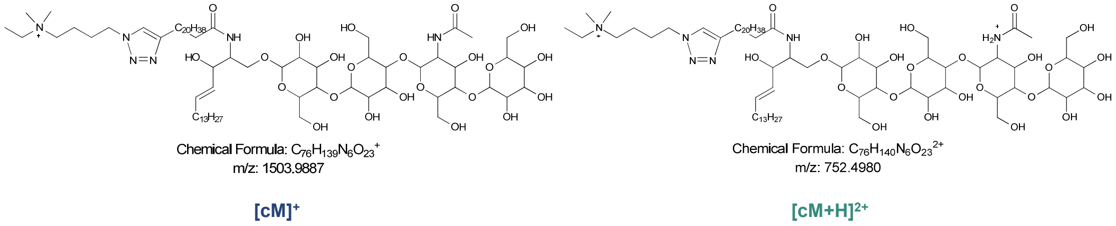

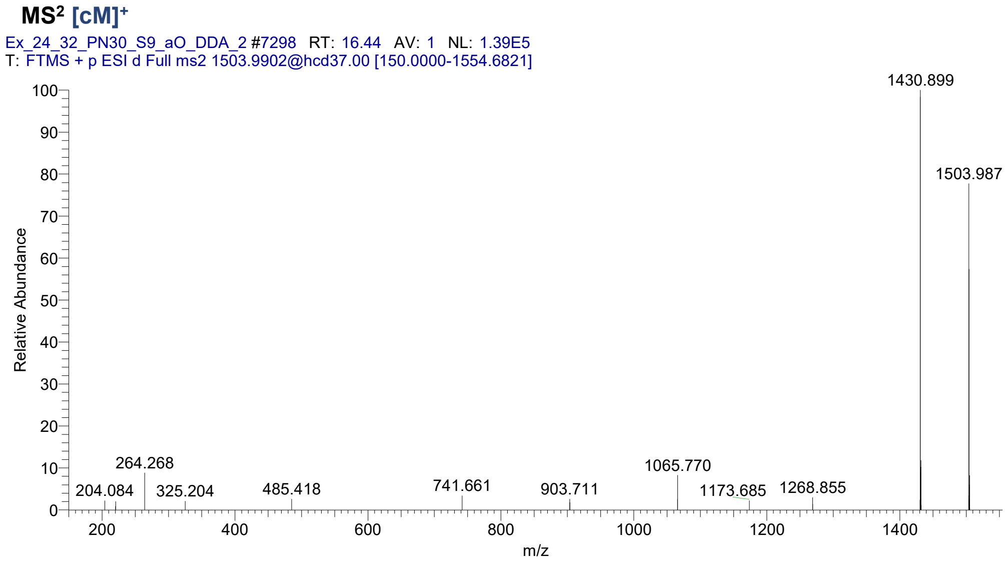

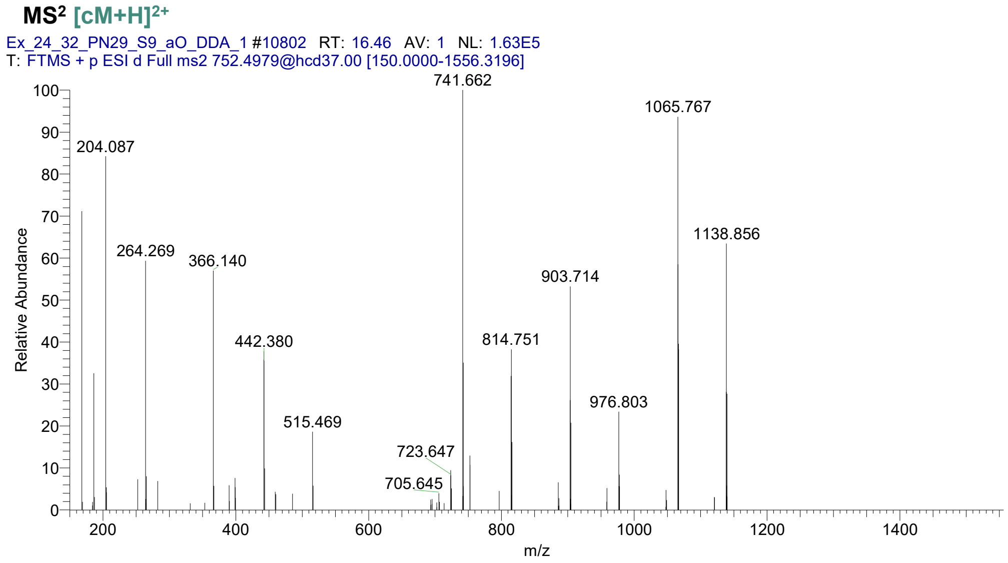

◂ Back to Content

HexNAc-Hex3Cer 18:1;O2/24:1;C171 1503.9886 – $C_{76}H_{139}O_{23}N_6^+$ proposed fragmentation scheme

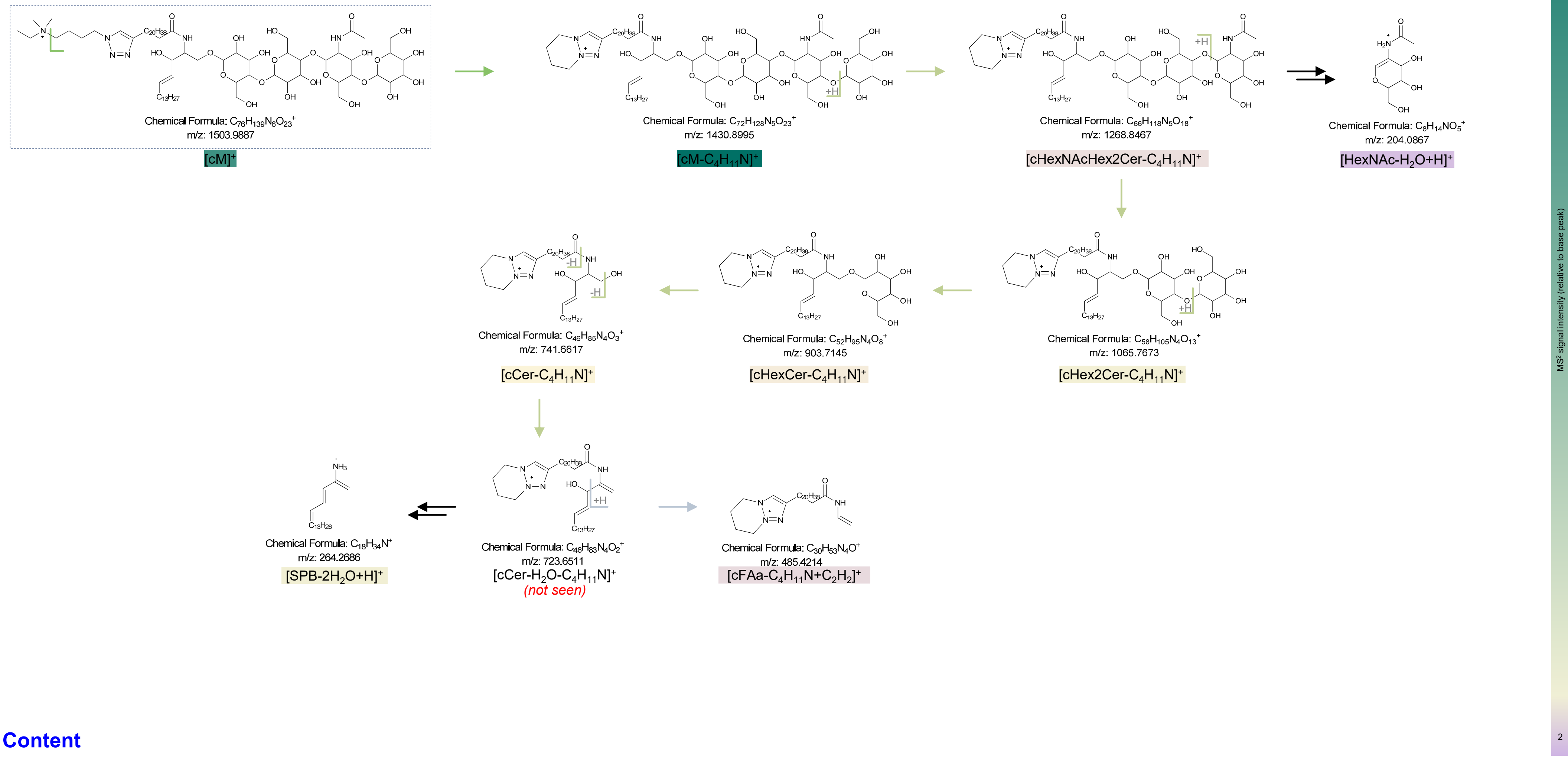

◂ Back to Content

HexNAc-Hex3Cer 18:1;O2/24:1;C171 752.4980 – $C_{76}H_{140}O_{23}N_6^{2+}$ proposed fragmentation scheme

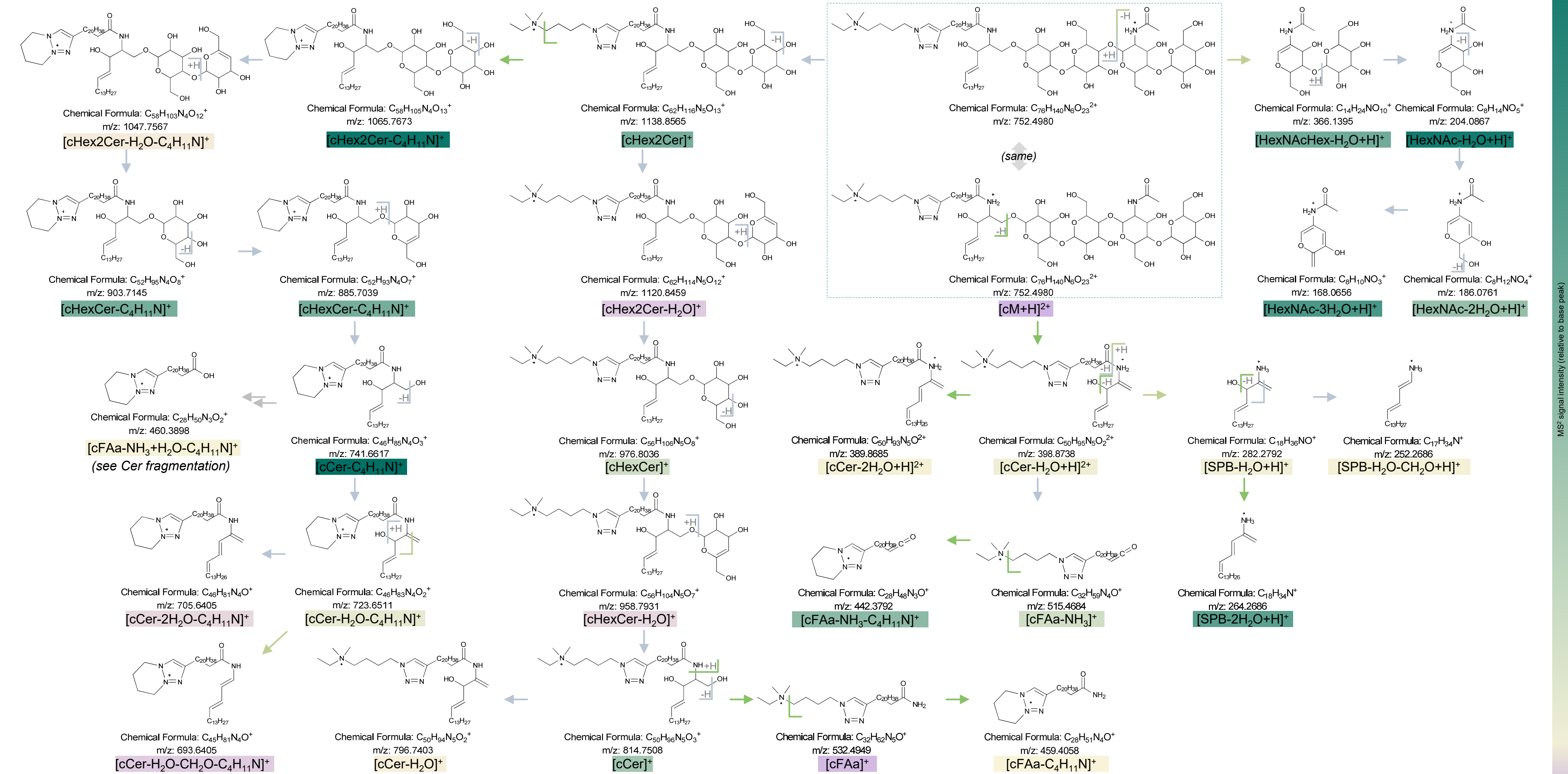

◂ Back to Content

HexNAc-Hex3Cer 18:1;O2,C171/24:1 1503.9886 – $C_{76}H_{139}O_{23}N_6^+$ / 752.4980 – $C_{76}H_{140}O_{23}N_6^{2+}$

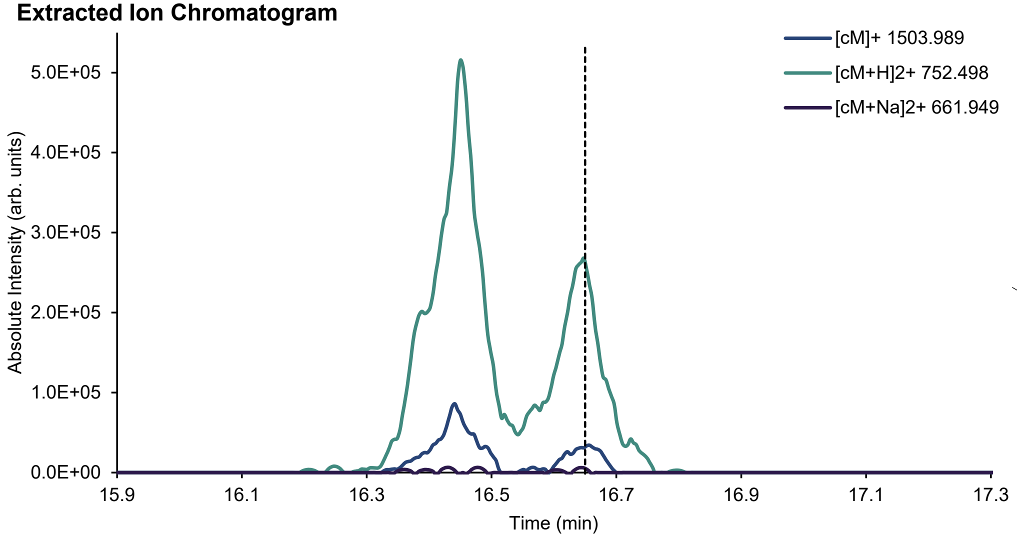

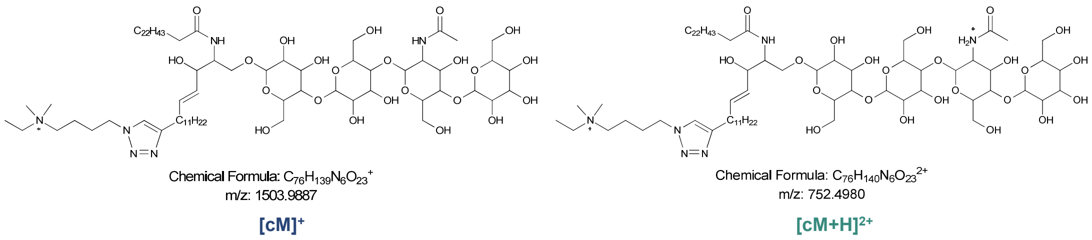

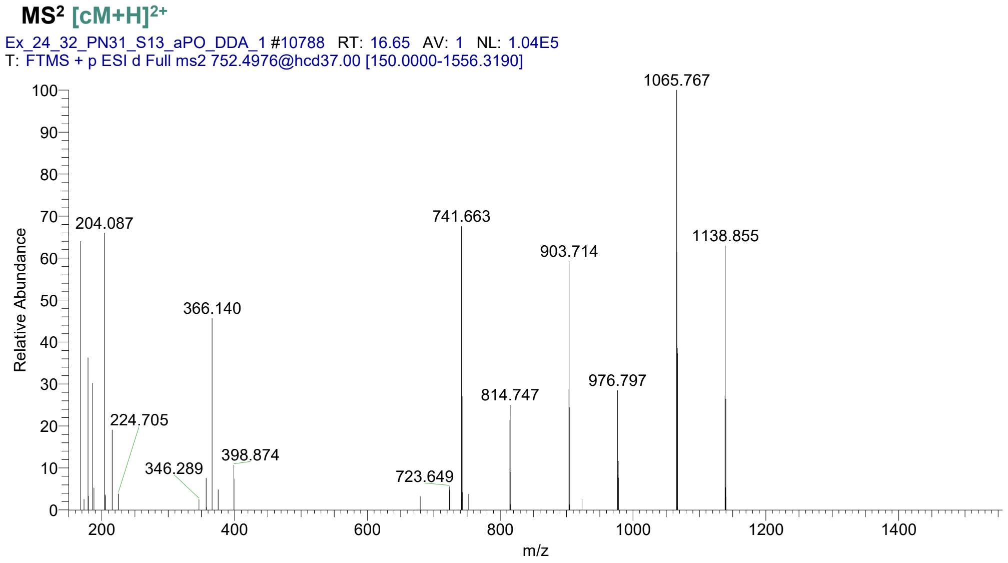

◂ Back to Content

HexNAc-Hex3Cer 18:1;O2,C171/24:1 752.4980 – $C_{76}H_{140}O_{23}N_6^{2+}$
proposed fragmentation scheme

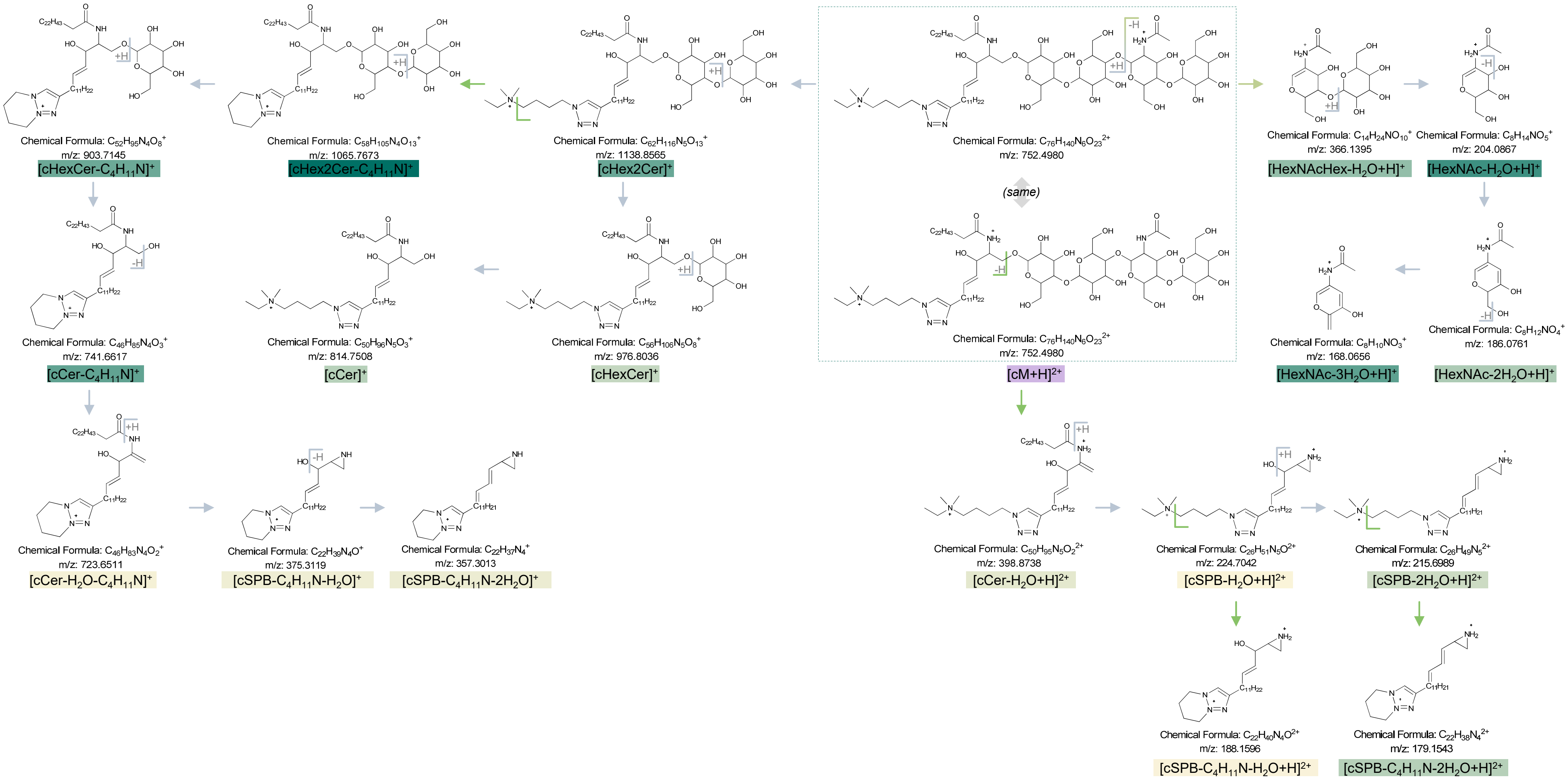

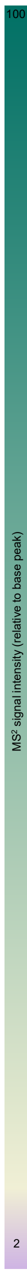

◂ Back to Content

SM 18:1;O2/24:1;C171 979.8062 – $C_{55}H_{108}O_6N_6P^+$ / 490.4068 – $C_{55}H_{109}O_6N_6P^{2+}$

Extracted Ion Chromatogram

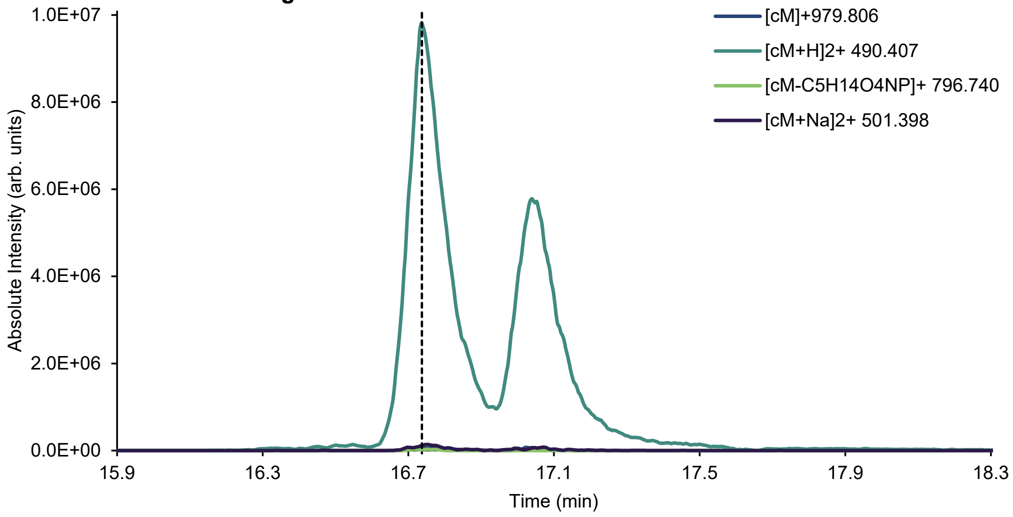

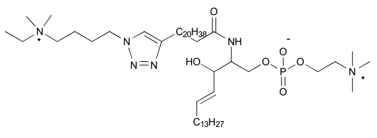

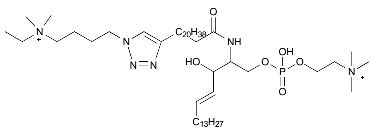

MS² [cM]⁺

Ex_24_32_PN29_S9_aO_DDA_1 #11009 RT: 16.76 AV: 1 NL: 1.18E5
T: FTMS + p ESI d Full ms2 979.8063@hcd37.00 [102.0014-1020.0145]

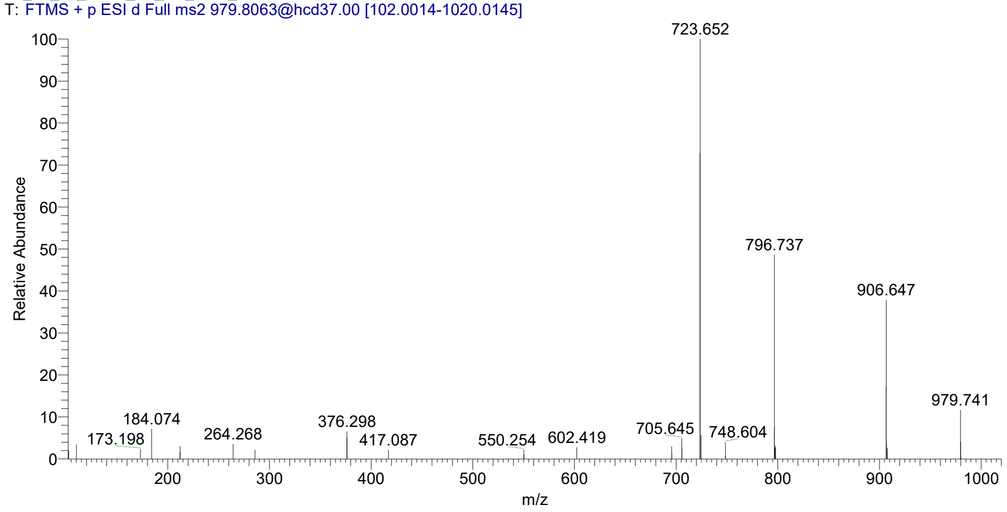

MS² [cM+H]²⁺

Ex_24_32_PN29_S9_aO_DDA_1 #11074 RT: 16.85 AV: 1 NL: 3.38E6
T: FTMS + p ESI d Full ms2 490.4065@hcd37.00 [102.1653-1021.6533]

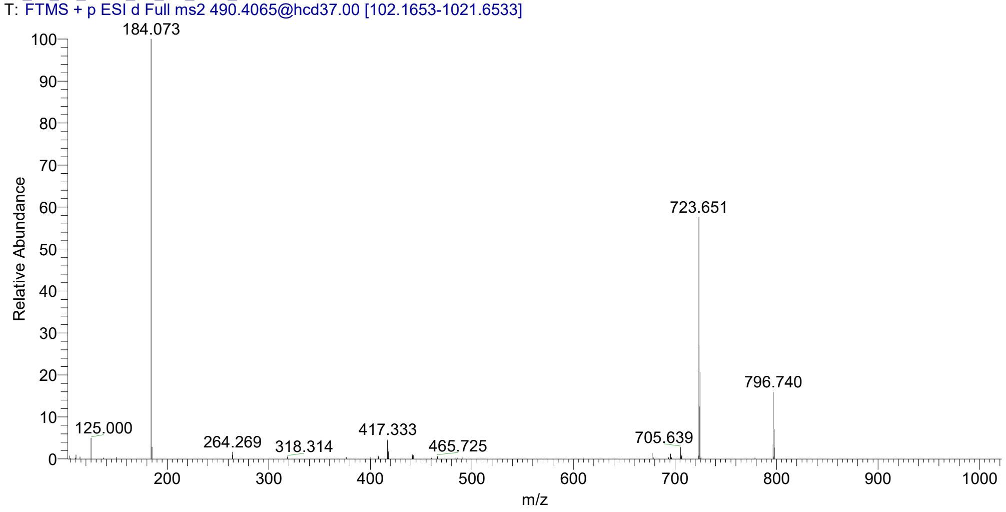

◂ Back to Content

SM 18:1;O2/24:1;C171 979.8062 – $C_{55}H_{108}O_6N_6P^+$ proposed fragmentation scheme

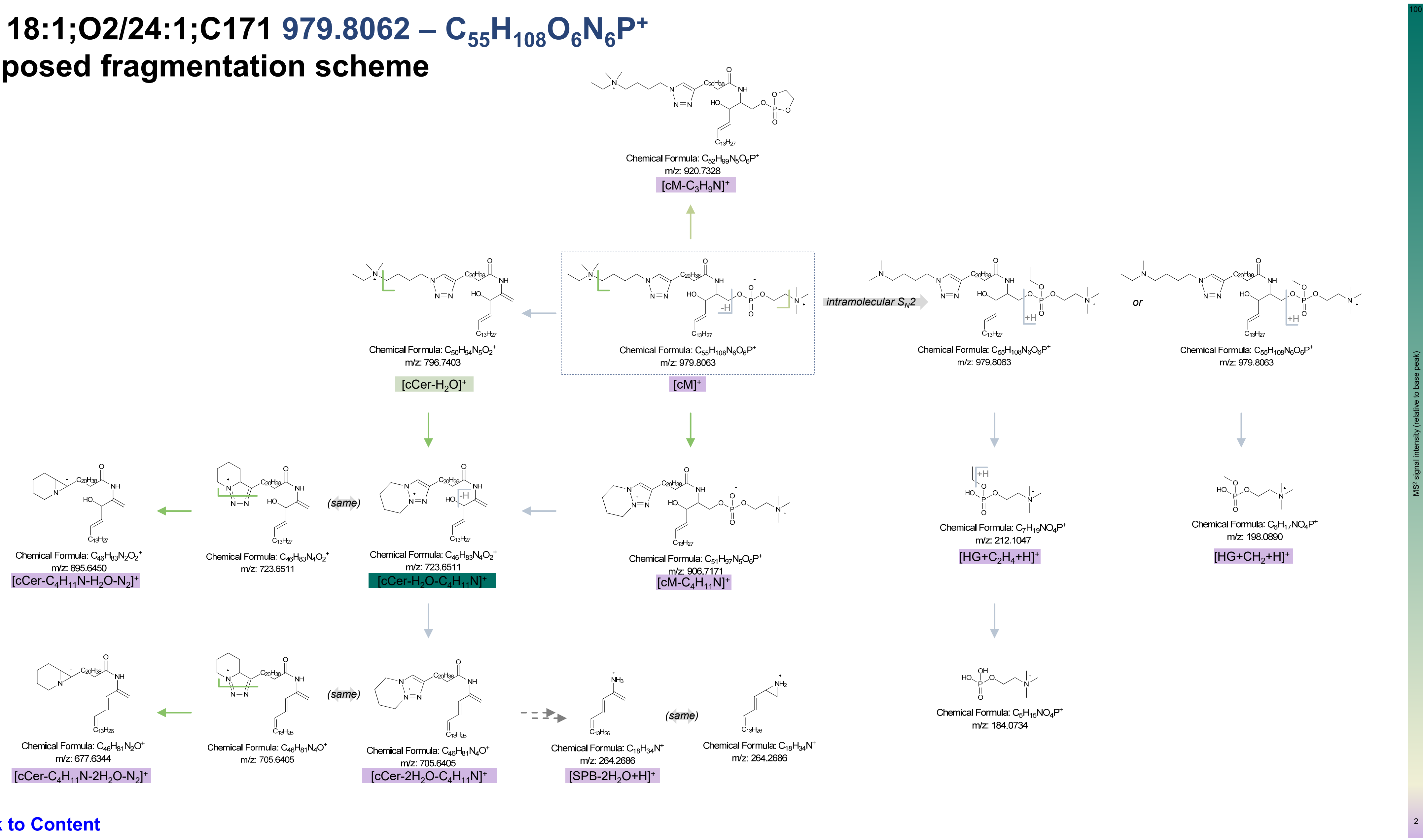

◂ Back to Content

SM 18:1;O2/24:1;C171 490.4068 – $C_{55}H_{109}O_6N_6P^{2+}$
proposed fragmentation scheme

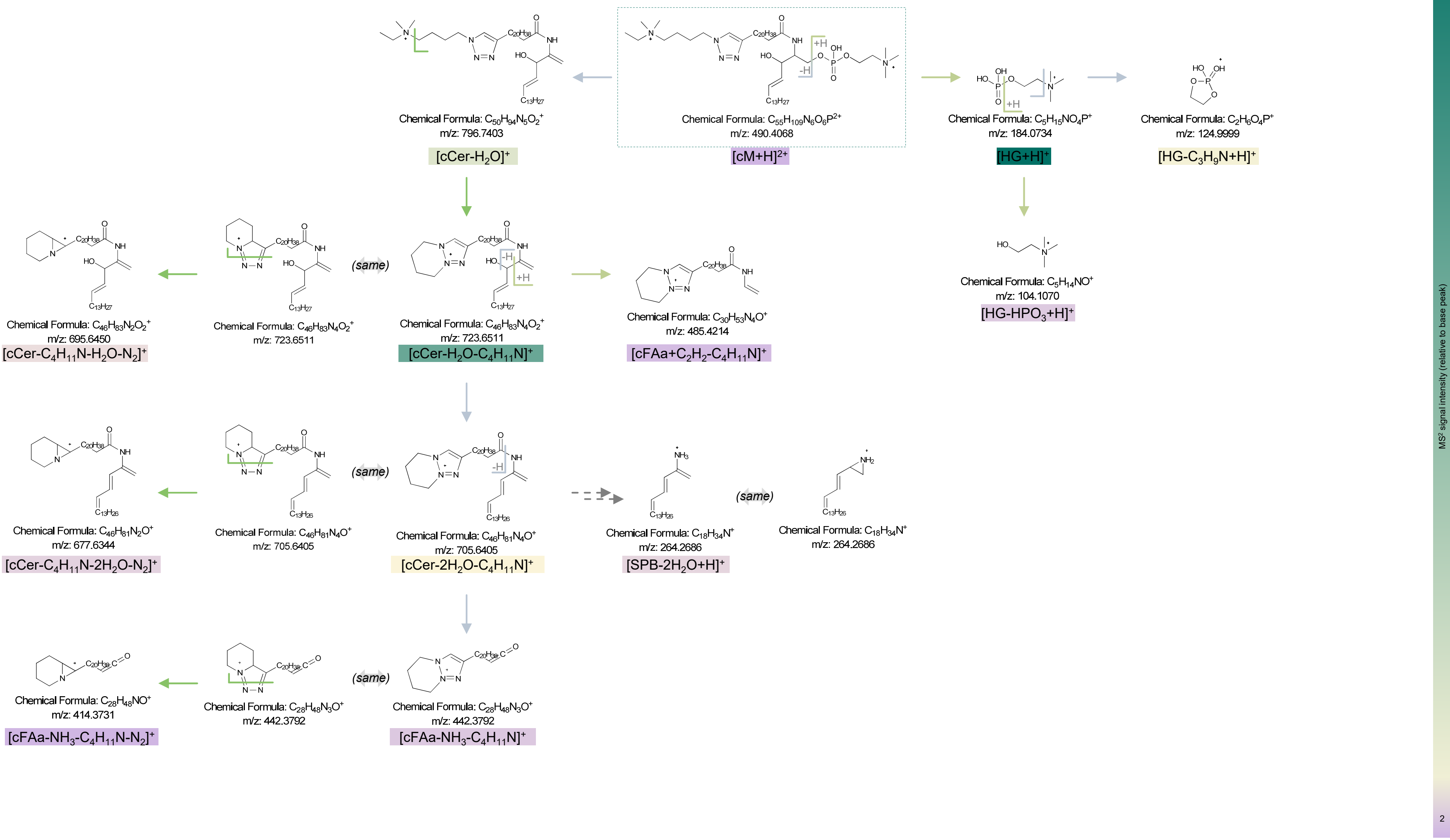

◂ Back to Content

SM 18:1;O2,C171/24:1 979.8062 – $C_{55}H_{108}O_6N_6P^+$ / 490.4068 – $C_{55}H_{109}O_6N_6P^{2+}$

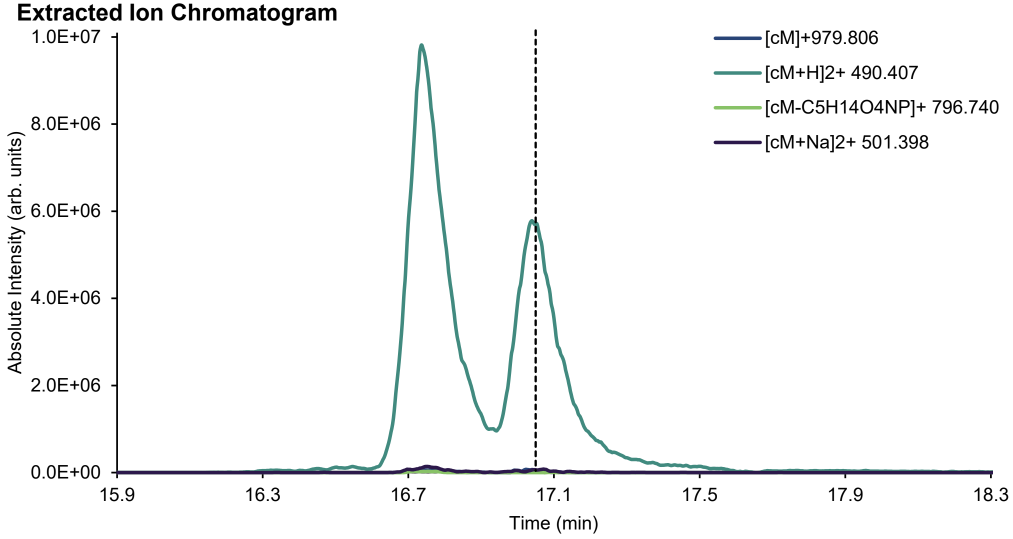

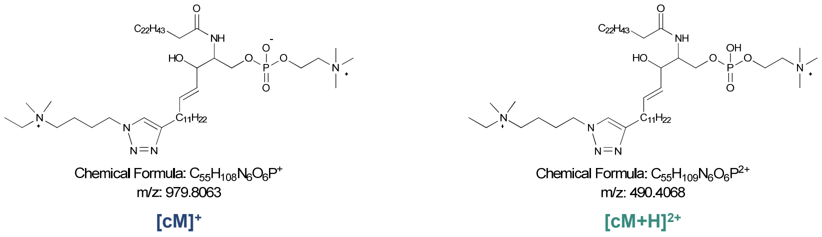

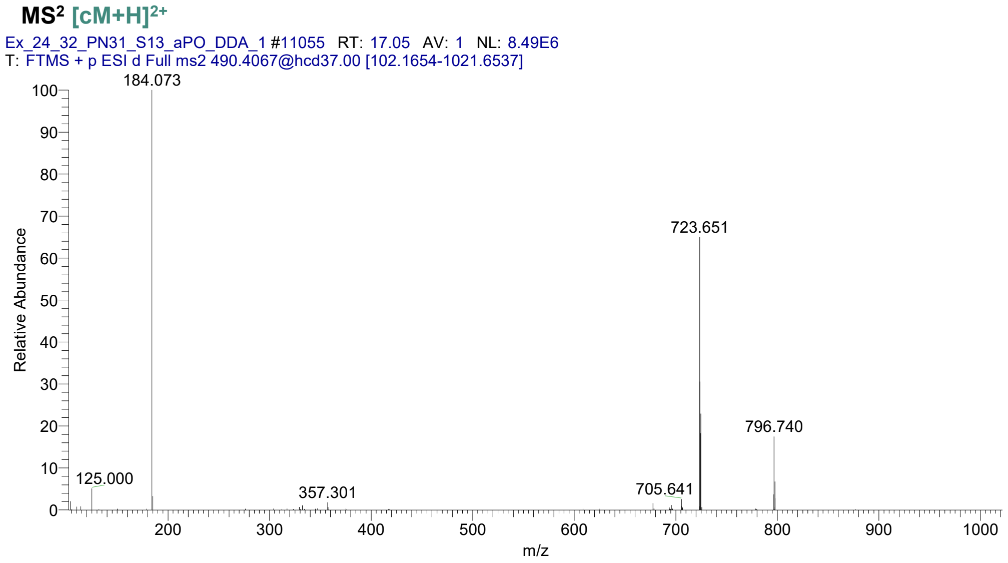

◂ Back to Content

SM 18:1;O2,C171/24:1 490.4068 – $C_{55}H_{109}O_6N_6P^{2+}$ proposed fragmentation scheme

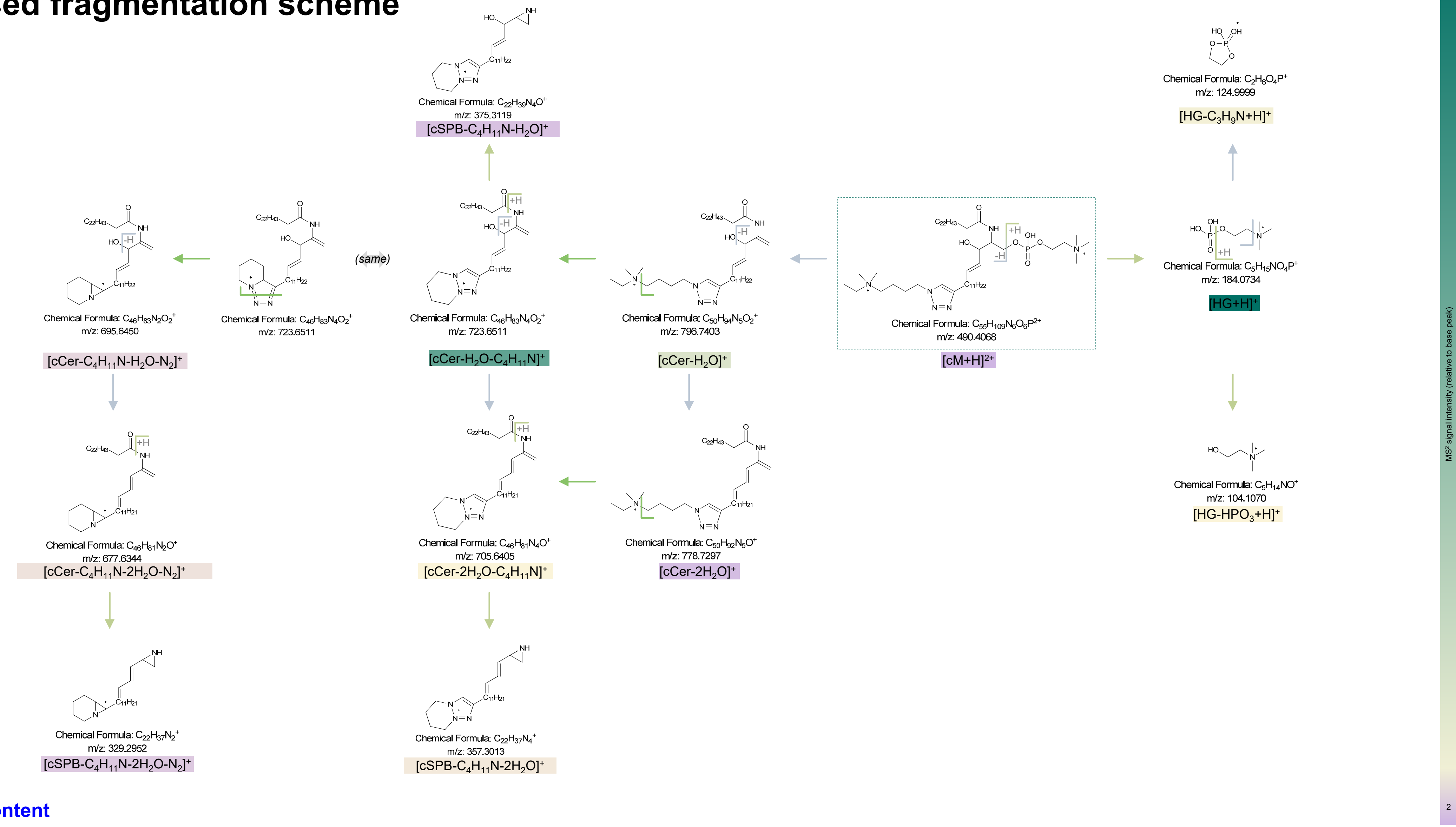

◂ Back to Content

FA 18:1;C171 449.3850 – $C_{26}H_{49}N_4O_2^+$

Extracted Ion Chromatogram

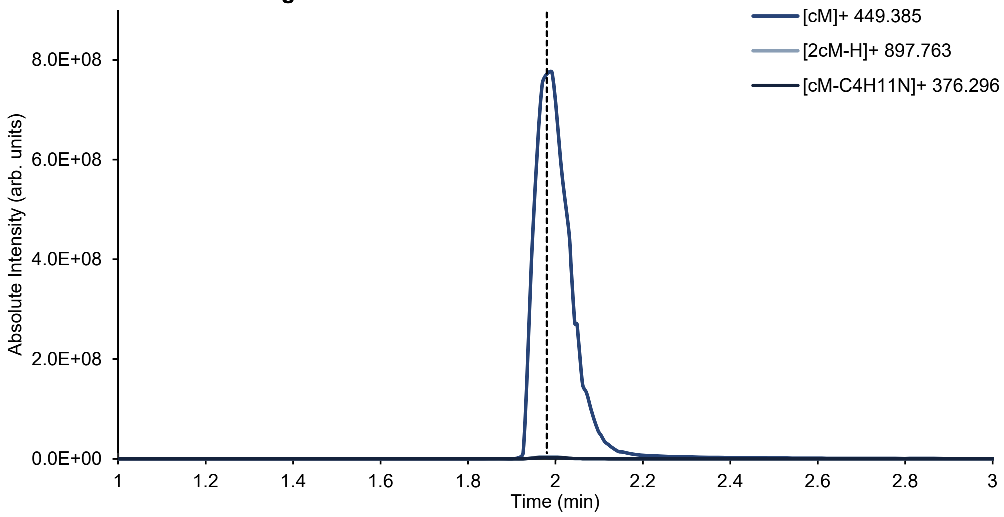

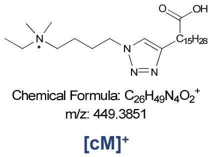

MS² [cM]⁺

Ex_23_24_PN16 #1219 RT: 1.95 AV: 1 NL: 4.06E8
T: FTMS + p ESI d Full ms2 449.3842@hcd30.00 [50.0000-478.9839]

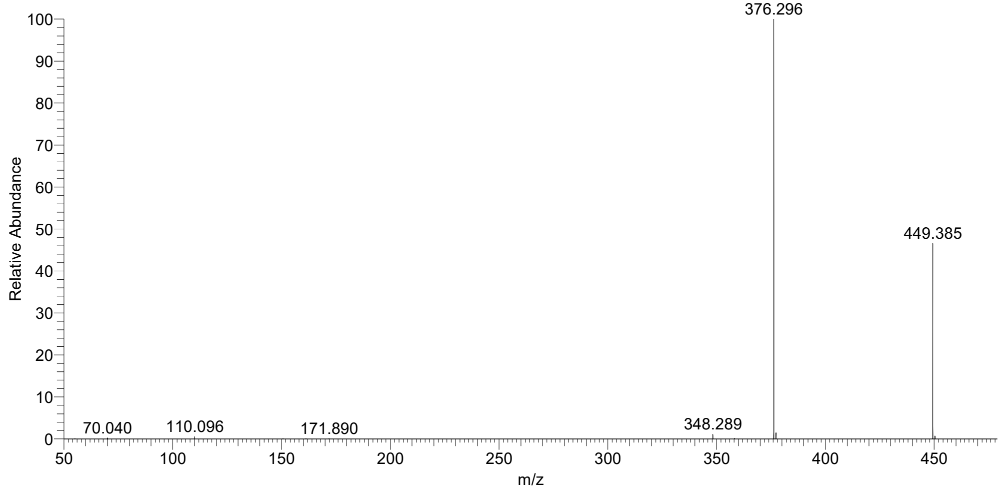

◂ Back to Content

FA 18:1;C171 449.3850 – $C_{26}H_{49}N_4O_2^+$ proposed fragmentation scheme

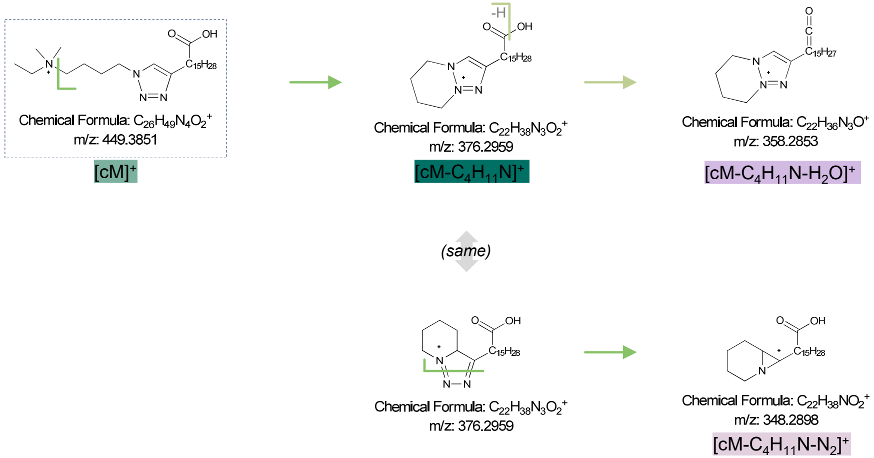

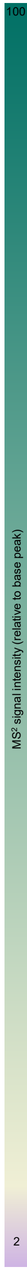

◂ Back to Content

MG 18:1;C171 523.4218 – $C_{29}H_{55}N_4O_4^+$

Extracted Ion Chromatogram

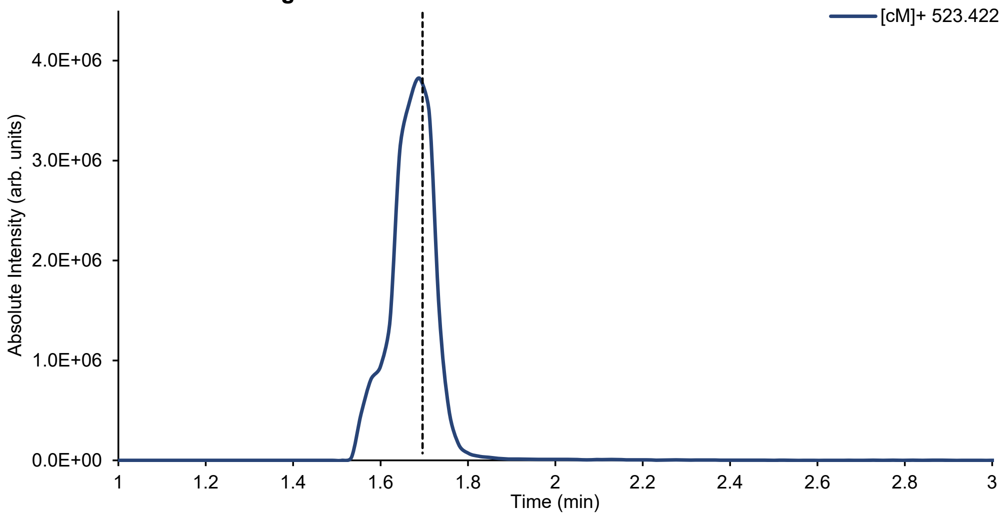

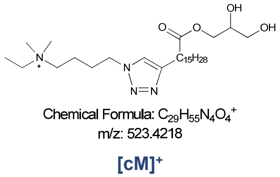

MS² [cM]⁺

Ex_23_50_PN01 #1090 RT: 1.72 AV: 1 NL: 2.17E6
T: FTMS + p ESI d Full ms2 523.4211@hcd35.00 [55.4502-554.5016]

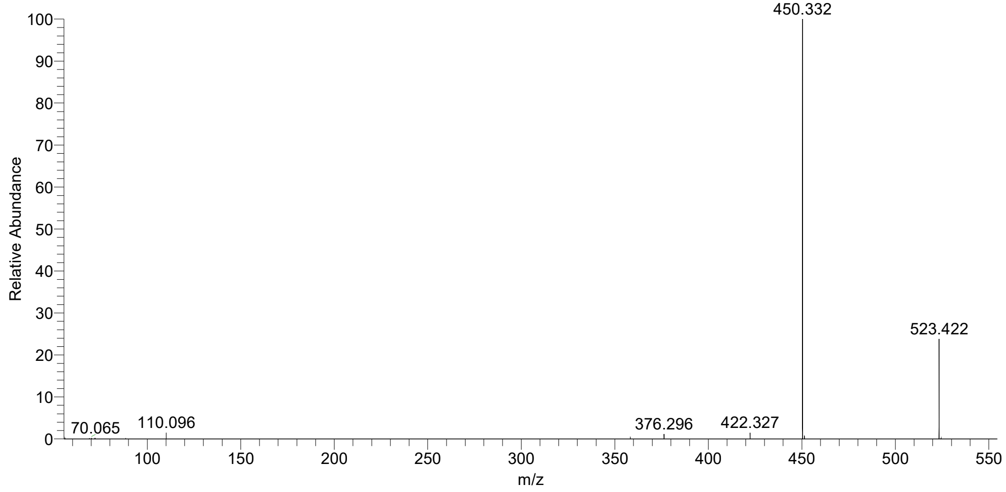

◂ Back to Content

MG 18:1;C171 523.4218 – $C_{29}H_{55}N_4O_4^+$ proposed fragmentation scheme

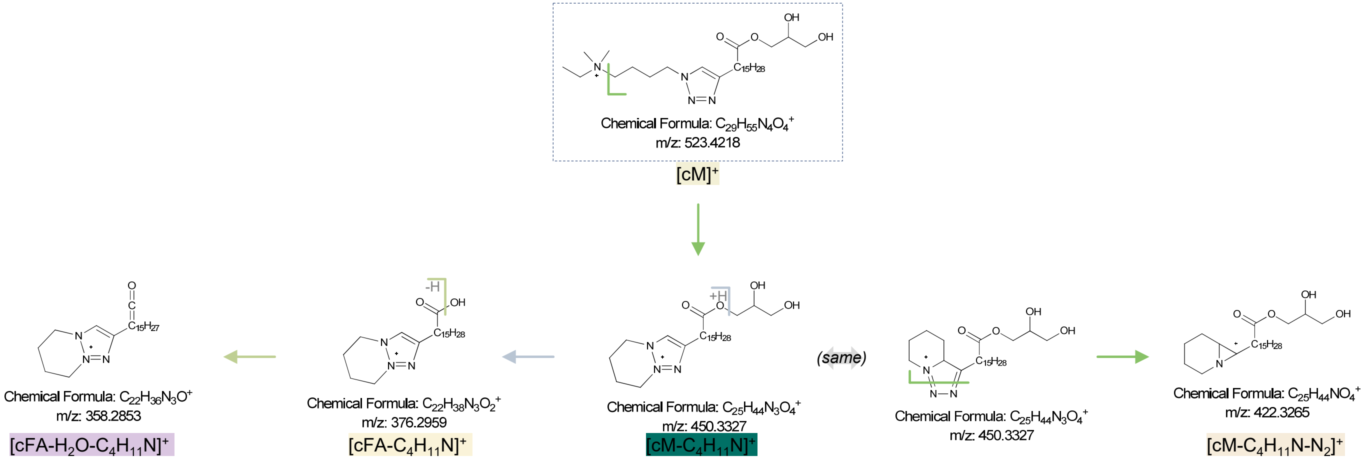

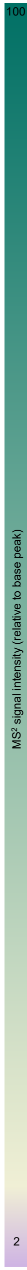

◂ Back to Content

DG 18:1;C171_18:1 787.6670 – $C_{47}H_{87}N_4O_5^+$

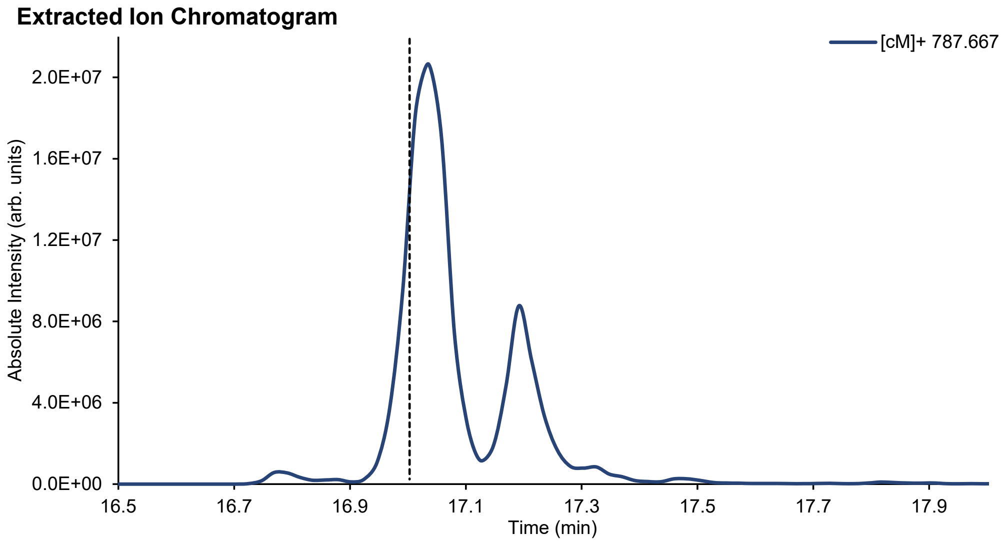

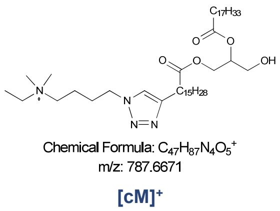

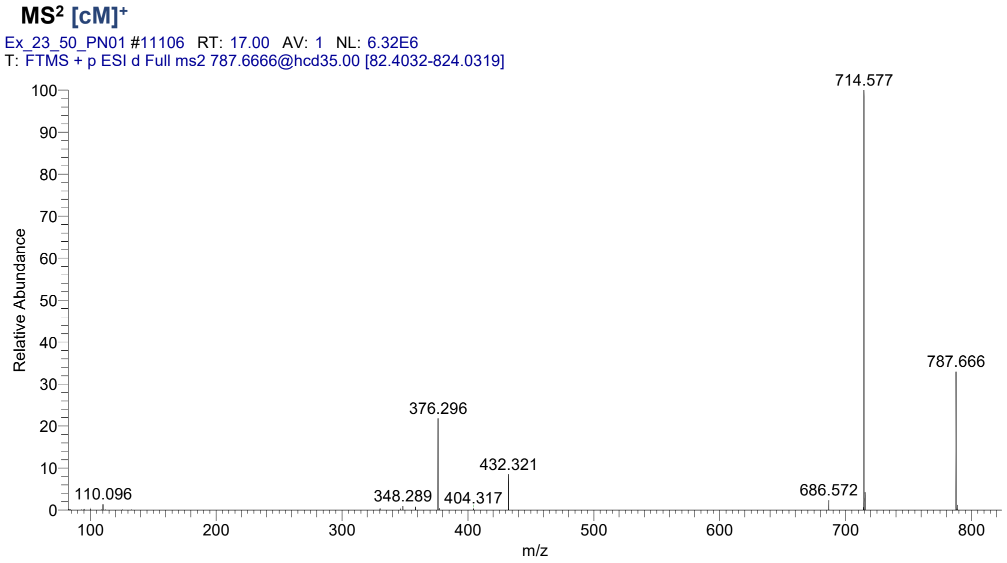

◂ Back to Content

DG 18:1;C171_18:1 787.6670 – $C_{47}H_{87}N_4O_5^+$
proposed fragmentation scheme

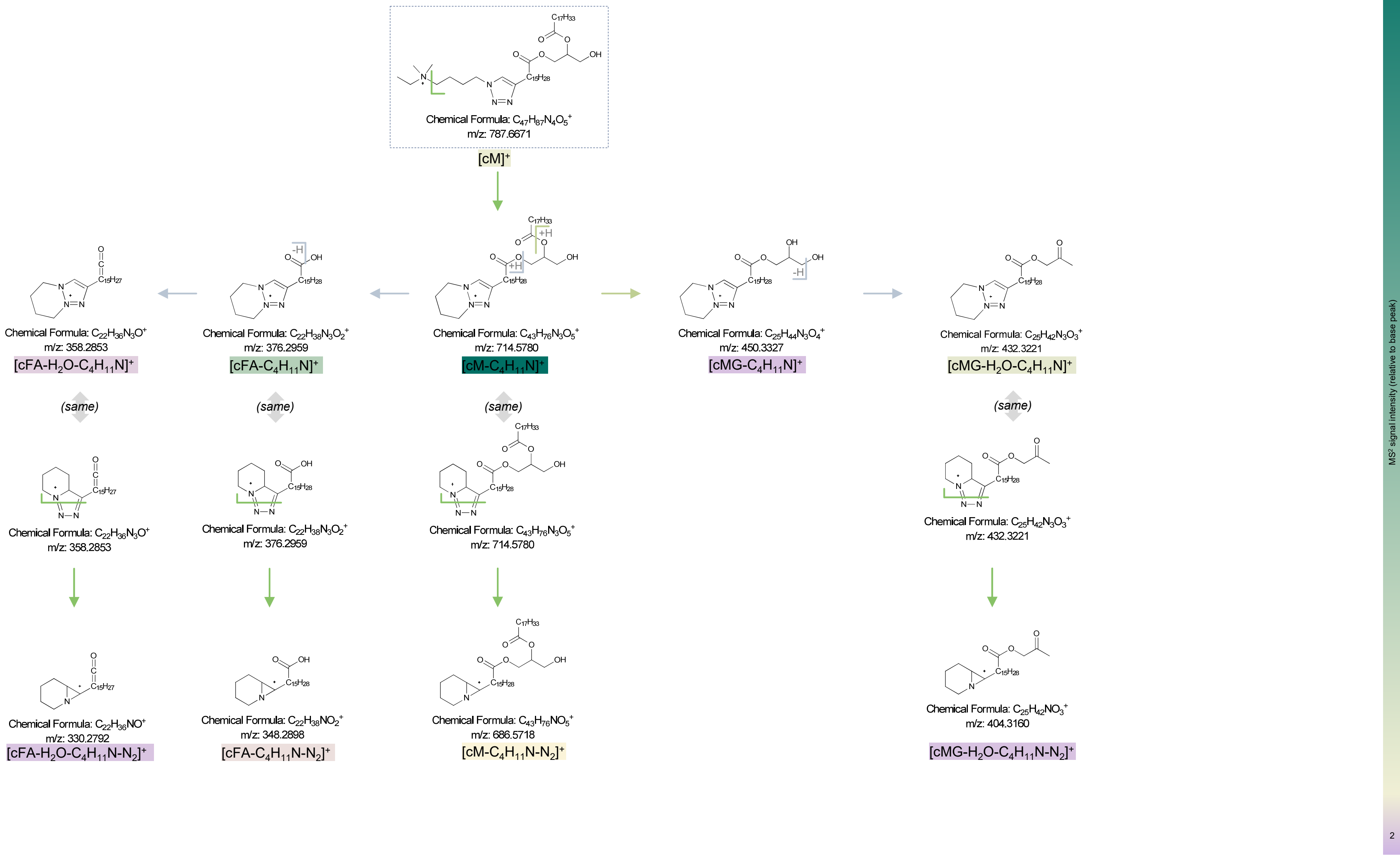

◂ Back to Content

TG 18:1;C171_18:1_16:0 1025.8968 – $C_{63}H_{117}N_4O_6^+$

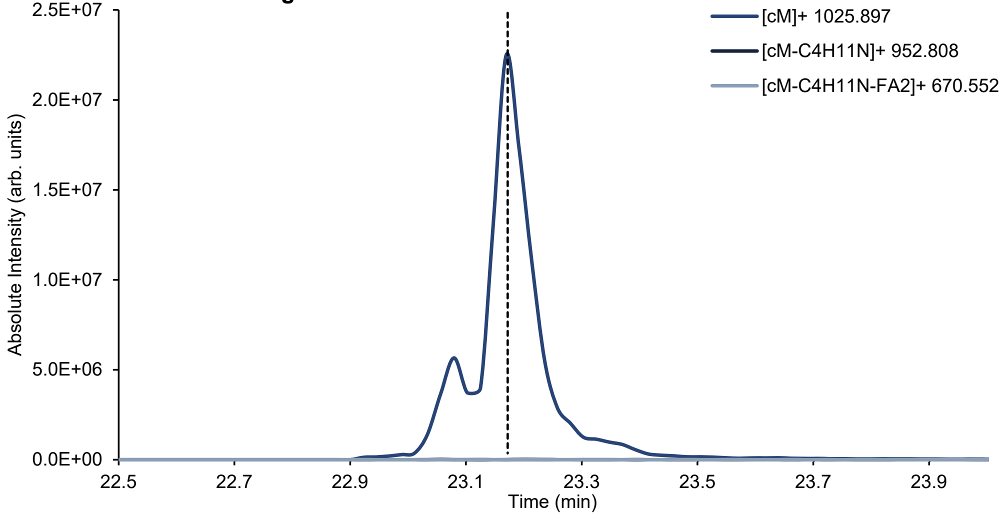

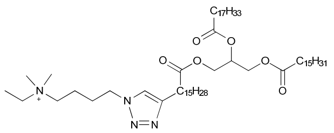

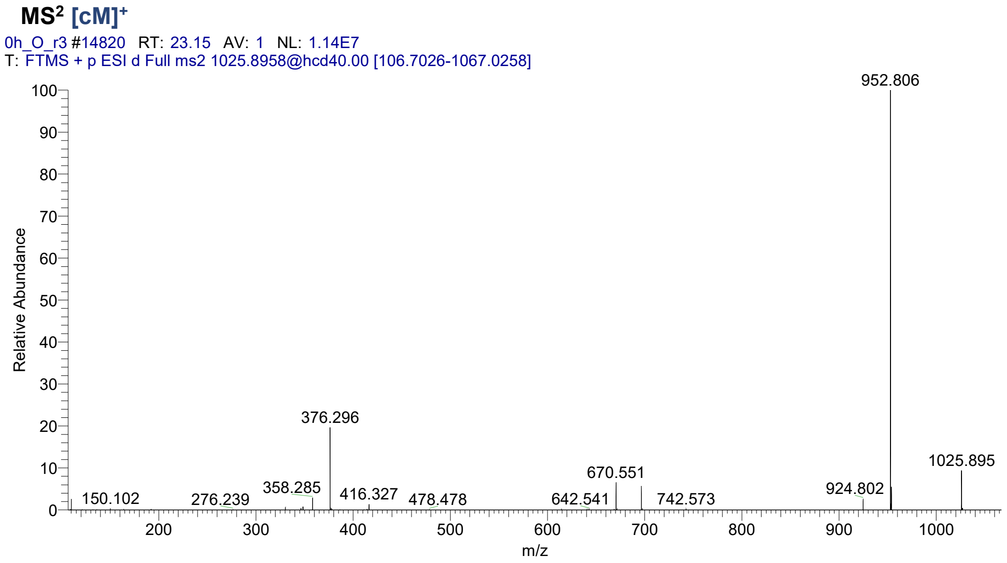

◂ Back to Content

TG 18:1;C171_18:1_16:0 1025.8968 – $C_{63}H_{117}N_4O_6^+$
proposed fragmentation scheme

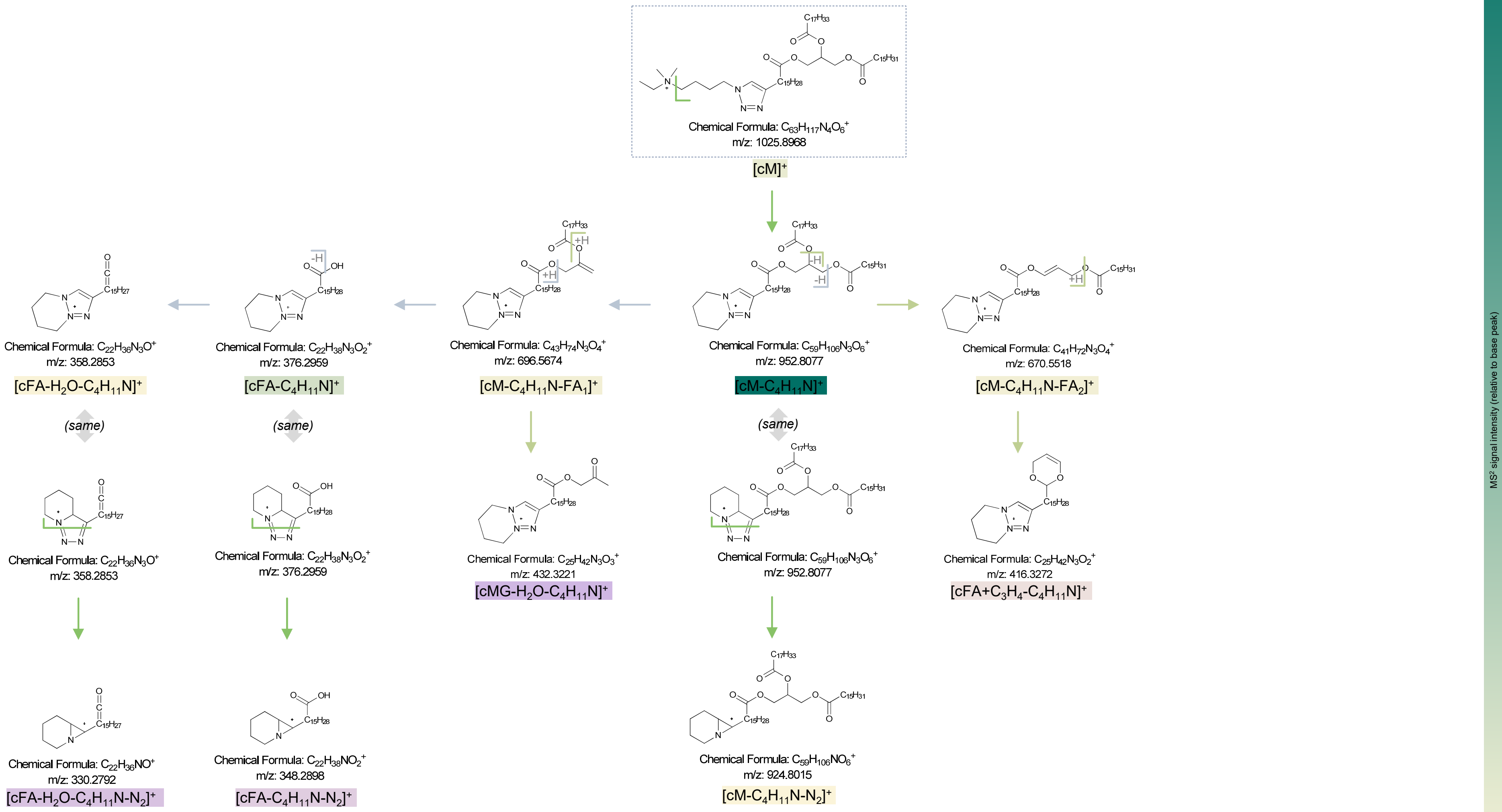

◂ Back to Content

CE 18:1;C171 817.7293 – $C_{53}H_{93}N_4O_2^+$

Extracted Ion Chromatogram

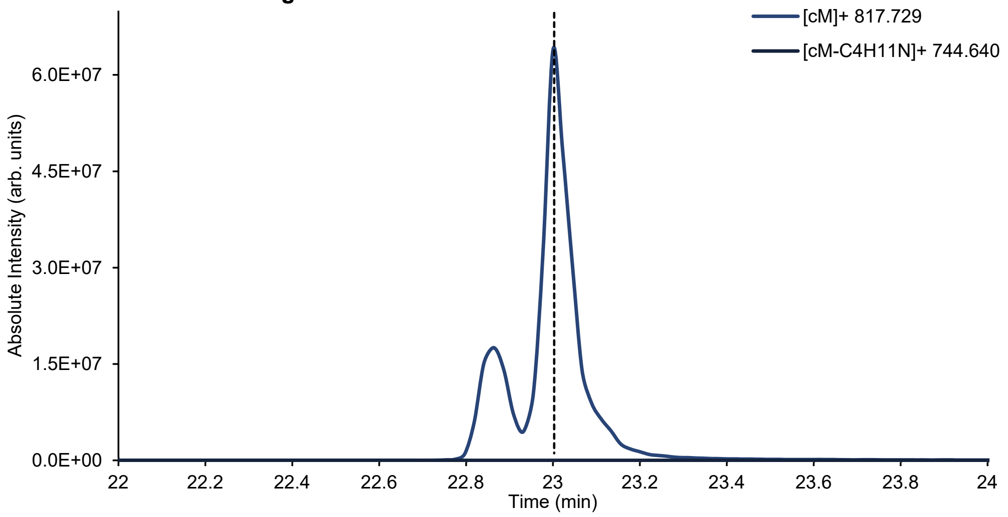

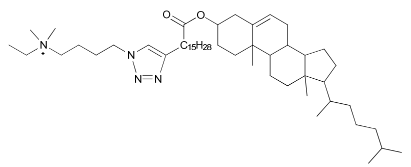

MS² [cM]⁺

0h_O_r1 #14742 RT: 23.02 AV: 1 NL: 1.70E7
T: FTMS + p ESI d Full ms2 817.7285@hcd40.00 [85.4695-854.6951]

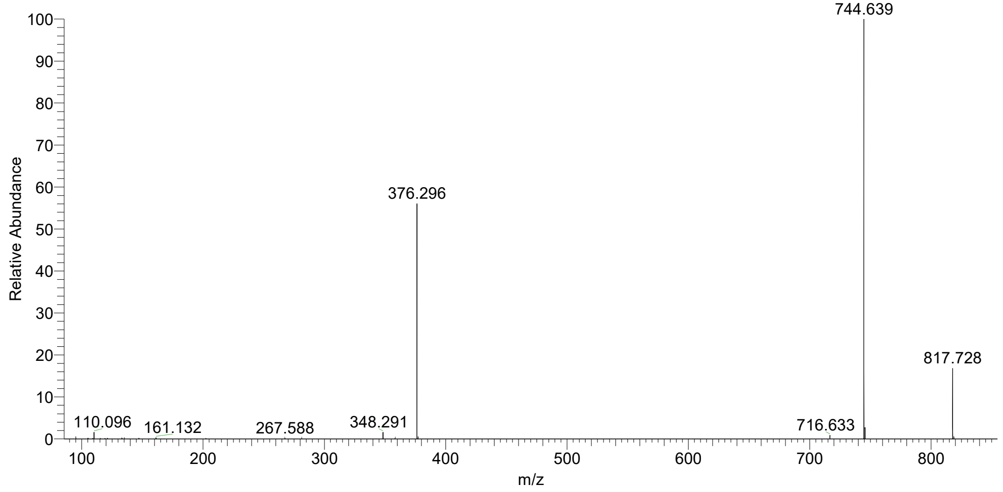

◂ Back to Content

CE 18:1;C171 817.7293 – $C_{53}H_{93}N_4O_2^+$ proposed fragmentation scheme

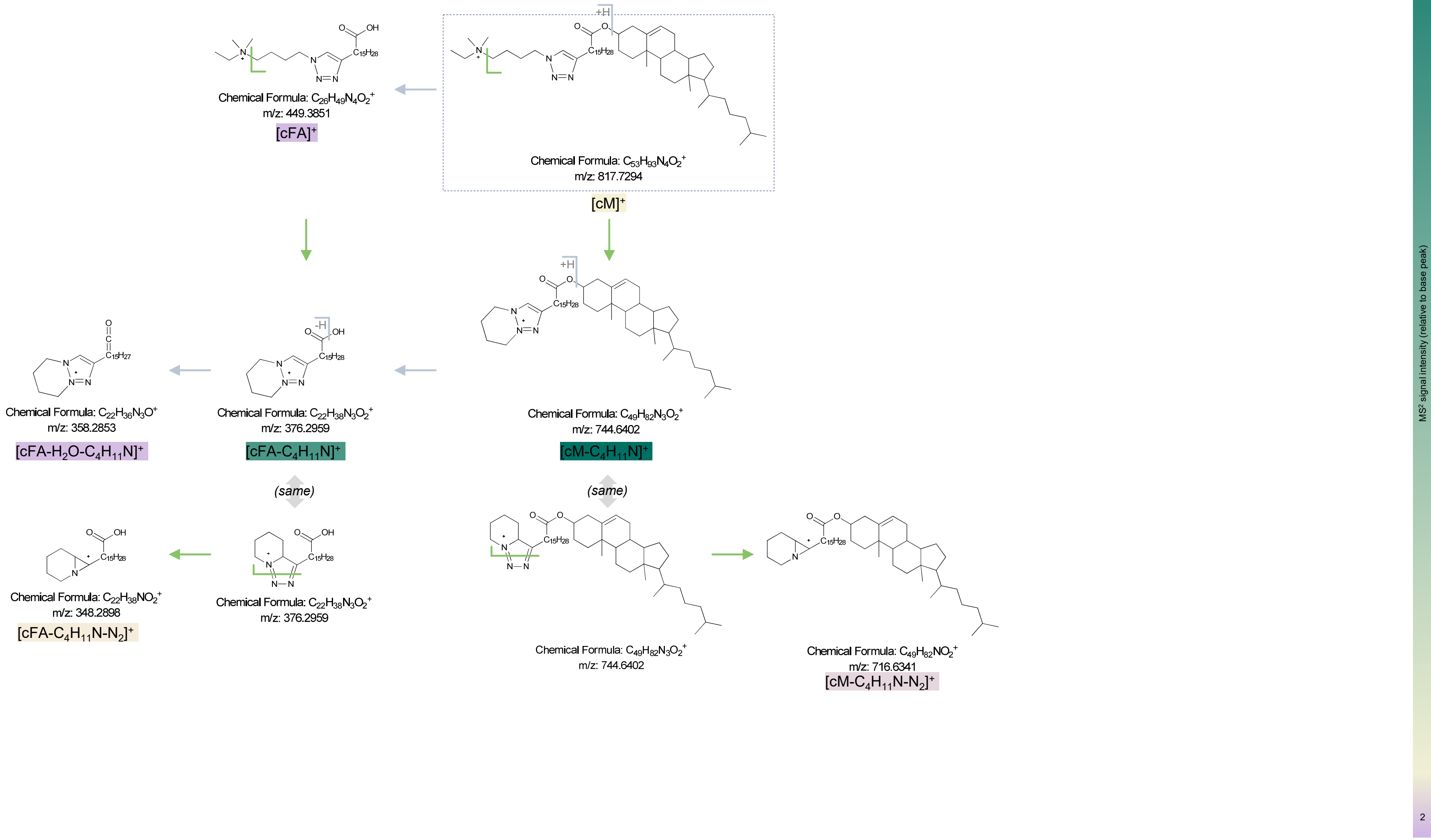

◂ Back to Content
